# Transcriptomic profile induced in bone marrow mesenchymal stromal cells after interaction with multiple myeloma cells: implications in myeloma progression and myeloma bone disease

**DOI:** 10.18632/oncotarget.2058

**Published:** 2014-06-04

**Authors:** Antonio Garcia-Gomez, Javier De Las Rivas, Enrique M. Ocio, Elena Díaz-Rodríguez, Juan C. Montero, Montserrat Martín, Juan F. Blanco, Fermín M. Sanchez-Guijo, Atanasio Pandiella, Jesús F. San Miguel, Mercedes Garayoa

**Affiliations:** ^1^ Centro de Investigación del Cáncer, IBMCC (Universidad de Salamanca-CSIC), Salamanca, Spain; ^2^ Hospital Universitario de Salamanca-IBSAL, Salamanca, Spain; ^3^ Centro en Red de Medicina Regenerativa y Terapia Celular de Castilla y León, Salamanca, Spain

**Keywords:** multiple myeloma, bone marrow mesenchymal stromal cells, tumor-stroma interactions, gene expression profiling, co-culture techniques, myeloma bone disease

## Abstract

Despite evidence about the implication of the bone marrow (BM) stromal microenvironment in multiple myeloma (MM) cell growth and survival, little is known about the effects of myelomatous cells on BM stromal cells. Mesenchymal stromal cells (MSCs) from healthy donors (dMSCs) or myeloma patients (pMSCs) were co-cultured with the myeloma cell line MM.1S, and the transcriptomic profile of MSCs induced by this interaction was analyzed. Deregulated genes after co-culture common to both d/pMSCs revealed functional involvement in tumor microenvironment cross-talk, myeloma growth induction and drug resistance, angiogenesis and signals for osteoclast activation and osteoblast inhibition. Additional genes induced by co-culture were exclusively deregulated in pMSCs and predominantly associated to RNA processing, the ubiquitine-proteasome pathway, cell cycle regulation, cellular stress and non-canonical Wnt signaling. The upregulated expression of five genes after co-culture (*CXCL1*, *CXCL5* and *CXCL6* in d/pMSCs, and *Neuregulin 3* and *Norrie disease protein* exclusively in pMSCs) was confirmed, and functional *in vitro* assays revealed putative roles in MM pathophysiology. The transcriptomic profile of pMSCs co-cultured with myeloma cells may better reflect that of MSCs in the BM of myeloma patients, and provides new molecular insights to the contribution of these cells to MM pathophysiology and to myeloma bone disease.

## INTRODUCTION

Multiple myeloma (MM) is a hematological malignancy characterized by the abnormal expansion of clonal plasma cells in the bone marrow (BM). It is widely admitted that the behavior of malignant plasma cells is not only dependent on their genomic abnormalities, but also, on the complex and reciprocal relationships of myeloma cells with their local BM niche [1, 2]. This BM microenvironment is a multifunctional complex network of extracellular matrix (ECM) proteins and non-hematopoietic cells, mainly of mesenchymal origin. Cross-talk of myeloma cells with this microenvironment is mediated by cell to cell, cell to ECM proteins or through soluble regulatory factors such as cytokines, chemokines and growth factors [3, 4]; protein, mRNA and miRNA interchange via MSC-derived exosomes has recently been reported as well [5]. These interactions have bidirectional consequences: on the one hand, myeloma cells perturb the BM homeostasis, causing anemia and immune suppression [6]. Further, these interactions may uncouple normal bone remodeling, reducing osteoblast (OB) differentiation and function and promoting osteoclast (OC) formation and resorption, and leading to the development of osteolytic bone lesions [7, 8]. On the other hand, interactions of myeloma cells mostly with BM stromal cells and OCs, activate pleiotropic cascades of proliferative, survival and migration signaling pathways in myeloma cells, and protect them from chemotherapy-induced apoptosis [1, 9].

Mesenchymal stromal cells (MSCs) are the progenitors of most of the stromal components of the BM, capable of self-renewing and differentiation to OBs, chondrocytes, reticular fibroblasts, adipocytes and muscle cells [4, 10]. Several studies have compared MSCs derived from the BM of myeloma patients (pMSCs) and those from healthy donors (dMSCs) (reviewed in [11]). Although pMSCs showed a similar immunophenotype to that of dMSCs and supported long-term hematopoietic growth in like fashion [12, 13]*,* they functionally and genetically differ from their healthy counterparts. Isolated and expanded pMSCs in culture showed non-recurrent genomic alterations [14], displayed a deficient proliferative capacity and replicative potential [15] and produced abnormally high amounts of certain cytokines [12, 13, 16] compared to dMSCs. As well, pMSCs showed a premature senescence profile [17] and presented reduced efficiency to inhibit T-cell proliferation [18] and to differentiate into the osteoblastic lineage [13], as compared to dMSCs. In addition, gene expression profile (GEP) analyses revealed differential expression of genes in pMSCs coding for tumor-supportive and angiogenic factors, as well as for factors contributing to bone disease [13]. Even a distinct transcriptional pattern was found associated to the occurrence of bone lesions in pMSCs [19]. Since these differences have been found for isolated dMSCs and pMSCs after *in vitro* expansion, they are influenced by *in vitro* growth culture conditions and long-term absence of myeloma interactions in pMSCs [13, 20]. Therefore these differences may only partially reflect true dissimilarities between pMSCs and dMSCs as occurring in the BM milieu of myeloma patients and healthy subjects. Although increasing number of studies are reporting on the expression of specific genes in myeloma-interacting MSCs [21-27], gene expression changes in co-cultured MSCs (with respect to mono-culture conditions) have not been done on a genome-wide basis.

Taking all this into consideration, in this work we have established co-cultures between BM derived MSCs and the MM.1S myeloma cell line, and performed GEP studies on the MSC population to determine those deregulated genes due to the co-culture condition with respect to MSCs in mono-culture. Both dMSCs and pMSCs have been used and compared. Our data provide new insights in the understanding of the intercellular communication signals between myeloma cells and MSCs, and further delineate the pivotal role of MSCs in the pathophysiology of MM and that of myeloma bone disease (MBD).

## RESULTS

### Experimental setting and expression profiling of d/pMSCs after co-culture with the MM.1S myeloma cell line

**Figure 1:**
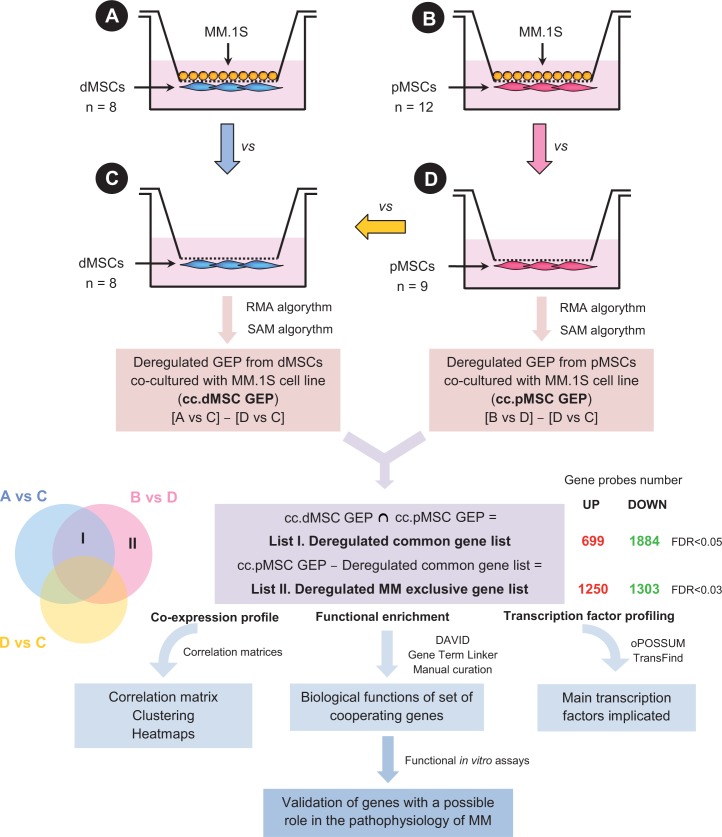
Experimental setting, microarray analysis workflow and subsequent analyses Graphical representation of the four experimental conditions under consideration: **(A)** dMSCs in co-culture with MM.1S, **(B)** pMSCs in co-culture with MM.1S, **(C)** dMSCs in mono-culture, and **(D)** pMSCs in mono-culture, which were used to obtain pure MSC populations after the 24 h co-culture. Microarray comparisons were performed to identify deregulated genes in the co-culture condition for dMSCs and pMSCs after exclusion of genes already differentially expressed in dMSCs and pMSCs in mono-culture ([A vs C] - [D vs C] = cc.dMSC GEP, and [B vs D] – [D vs C] = cc.pMSC GEP]). Next, two lists of differentially expressed genes in MSCs after co-culture with MM.1S cells were generated and represented in the Venn diagrams: (i) List I: deregulated genes in co-culture common to dMSCs and pMSCs, and (ii) List II: deregulated genes in co-culture exclusive of pMSCs. Numbers shown indicate genes with are differentially up- or down-regulated with respect to the mono-culture condition in both lists. FDR cut-off value in List II was set up to 0.03 in order to obtain a number of differentially expressed genes equivalent to List I. Both lists of genes (I and II) were the starting point for subsequent bioinformatic analyses as well as functional assays for selected genes outlined below.

Four experimental conditions using transwell chambers were established as depicted in Fig. 1: (A) dMSCs in co-culture with MM.1S cells; (B) pMSCs in co-culture with MM.1S cells; (C) dMSCs cultured in the same manner but without MM.1S cells; and (D) pMSCs also cultured without MM.1S cells. Characteristics of MM patients and healthy donors are detailed in Supp. Table S1. After a 24 hour co-culture period, RNA was isolated from separated MSC populations and used to hybridize oligonucleotide microarrays. First, we identified differentially expressed genes when comparing d/pMSC samples in co-culture with d/pMSCs from the same origin in mono-culture. Next, in order to identify differentially expressed genes in d/pMSCs only due to the co-culture condition, intrinsic differences between dMSCs and pMSCs were excluded from the respective gene signatures in the co-cultured condition, both for dMSCs and pMSCs. Finally, by determining differentially deregulated genes common to both dMSCs and pMSCs after co-culture, we generated a deregulated “common list” of significant genes [FDR (false discovery rate) < 0.05] (List I in Fig. 1), including 2583 genes, 699 upregulated and 1884 downregulated from mono-culture (Supp. Table S2). The remaining differentially expressed genes observed in co-cultured pMSCs but not present in the previous common list were considered exclusive of pMSCs. This “exclusive list” (List II in Fig.1) was set up with a FDR < 0.03, to allow a similar number of genes as in List I (2553 genes: 1250 upregulated and 1303 downregulated with respect to the mono-culture condition; Supp. Table S3).

### Co-expression and cluster analyses of gene signatures obtained for d/pMSCs co-cultured with myelomatous cells

**Figure 2:**
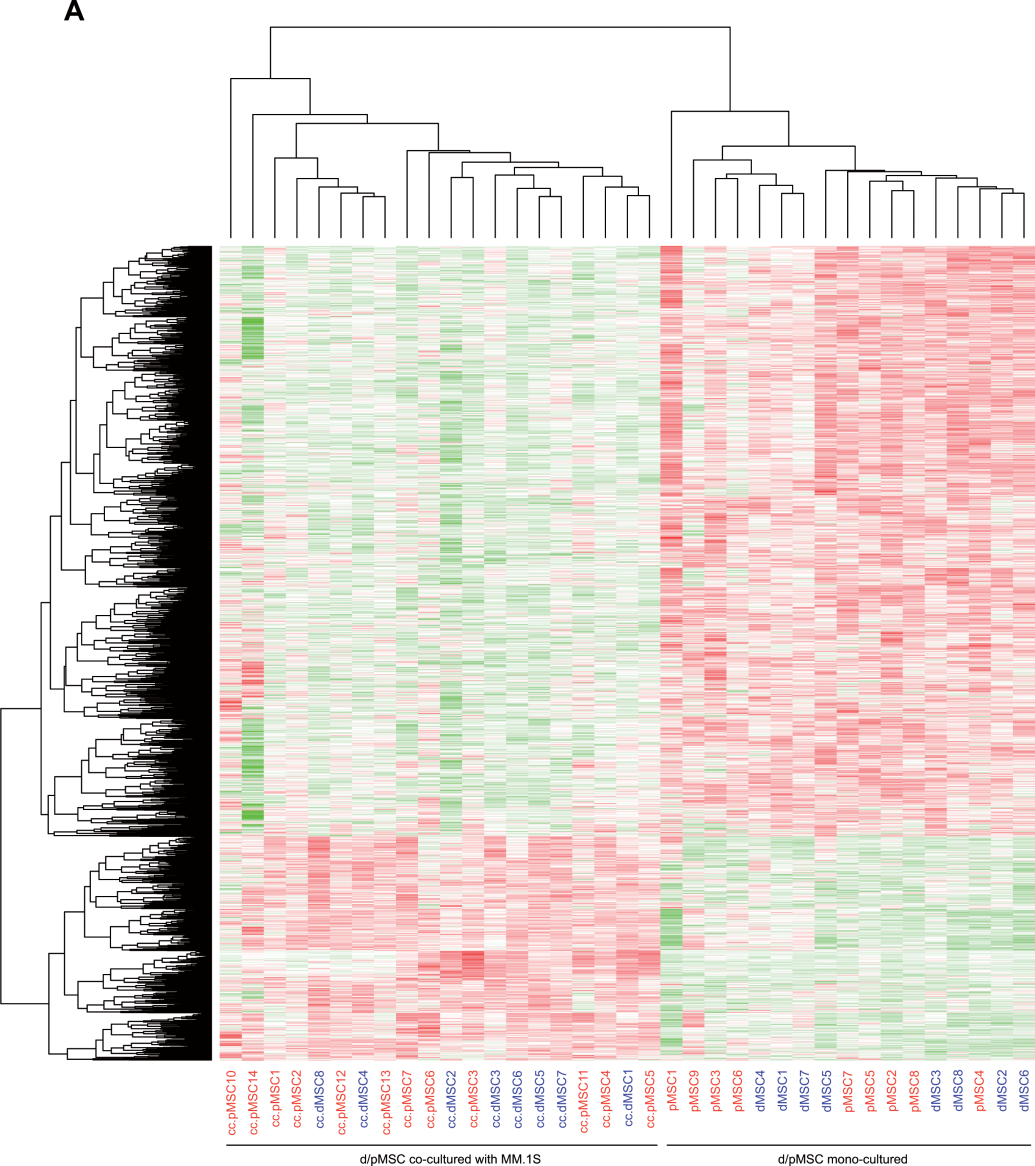

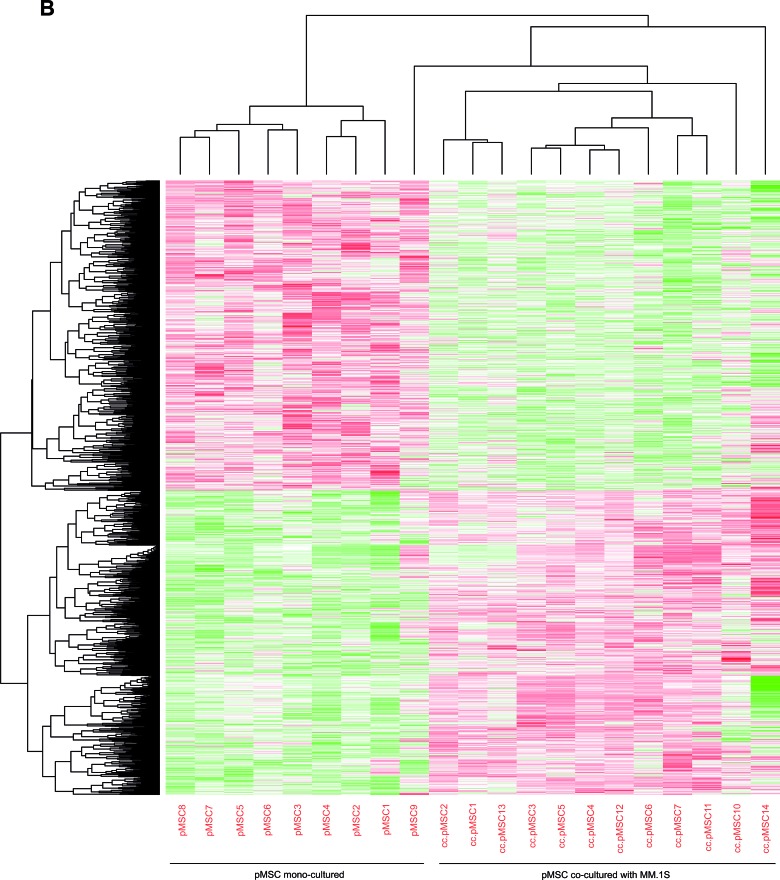
Hierarchical clustering of deregulated genes in MSCs due to co-culture with the MM.1S cell line **A**, Hierarchical clustering of deregulated genes in co-culture, common to dMSCs and pMSCs (List I, FDR < 0.03). Rows represent individual genes (2583: 699 upregulated and 1884 downregulated), whereas columns refer to each sample (a total of 37 MSC samples from donor or patient origin). The intensity of color saturation in each gene box (ranging from 2 to 14 in a log_2_ scale) depicts quantitative estimation of its expression level. Red color denotes high expression, increasing in brightness with higher values; green color denotes low expression, increasing brightness with lower values. White color denotes unchanged expression relative to the median expression value for each *probeset*. Samples are numerated in blue color for dMSCs and red color for pMSCs. **B**, Hierarchical clustering of deregulated genes in co-culture, exclusive of pMSCs (List II, FDR < 0.02). Rows represent individual genes (2553: 1250 upregulated and 1303 downregulated), whereas columns refer to each sample (a total of 21 pMSC samples, 9 in monoculture and 12 in co-culture). Color scale is the same as in A.

The expression heatmap from common deregulated genes in co-culture (List I) discriminated two main branches in the samples: one corresponding to the co-culture condition, whereas the other corresponded to mono-cultured MSCs. Confirming the semi-supervised character of the analysis, MSCs from donor or patient origin did not segregate within each branch, since genes from intrinsic differences between dMSCs and pMSCs in mono-culture were excluded (Fig. 2A). Similarly, hierarchical clustering of data from deregulated genes in co-culture exclusive of pMSCs (List II), showed a clear segregation of samples from mono-culture and the co-culture condition; however, we could not identify any further clusterization of pMSC samples relative to the occurrence of bone lesions, disease stage or other patient characteristics (Fig. 2B).

### Functional signatures linked to dMSCs and pMSCs co-cultured with MM.1S cells

By using *DAVID* (*http://david.abcc.ncifcrf.gov/*) enrichment tool, the most statistically significant functional categories associated to common upregulated genes in co-culture were relative to chemokine response, immune-inflammatory response, angiogenesis, regulation of proliferation and apoptosis, extracellular matrix remodelation and bone biology (Table 1). Similar associated functions were identified for metagroups after use of GeneTerm Linker (*http://gtlinker.dacya.ucm.es/*): chemokine response, cell communication, cell-matrix adhesion and regulation of proliferation and apoptosis (Table 1). The functional signature on downregulated genes of List I had lower statistical significance and did not reveal different functions to those in Table I. Nevertheless, some downregulated genes from List I were manually curated because of a reported effect on OB differentiation and/or function (e.g. *BMPR1A*, *CD276*, *EFNB2*, *FZD4*, *HMOX1*, *IL6ST*, *BMP7*, *SMAD2)*.

**Table 1:** Functional signatures of upregulated genes due to co-culture with MM.1S and common to d/pMSCs (List I, FDR < 0.03). Specific functional categories were assigned by DAVID Bioinformatics Resources 6.7, GeneTerm Linker or manual curation, with values of statistical significance, silhouette width (when applicable), together with a list of the differentially and significatively expressed loci associated to each function. Genes marked with an asterisk (*) refer to genes not initially included in the functional enrichment analysis due to a FDR > 0.03, but subsequently incorporated because of their potential role in myeloma.

DAVID Bioinformatics Resources 6.7				
***Term***	***Genes***	***Adjusted p-value (Benjamini)***	***Count /total list***	***Population hits/total***
**Chemokine response (25 different genes)**				
Chemotaxis	CCL2, CCL20, CCL3, CCL4, CCL7, CMKLR1, CXCL1, CXCL2, CXCL3, CXCL5, CXCL6, CXCR4, DOCK2, FGF2, IL16, IL1B, IL6, IL8, ITGAM, PLAUR, PRKCA, RAC2, RNASE2, ROBO1	7,29E-06	24/464	160/13528
Chemokine activity	CCL2, CCL20, CCL3, CCL4, CCL7, CXCL1, CXCL2, CXCL3, CXCL5, CXCL6, IL8	1,79E-03	11/443	46/12983
Leukocyte migration chemotaxis	CCL2, CXCL3, DOCK2, IL16, IL1B, IL6, IL8, ITGAM, PRKCA, VCAM1	1,46E-02	10/482	57/14116
**Immune / inflammatory response (45 different genes)**				
Inflammatory response	ATRN, C3, CCL2, CCL20, CCL3, CCL4, CCL7, CD44, CHST1, CR1, CXCL1, CXCL2, CXCL3, CXCL6, CXCR4, FN1, GPR68, IL1B, IL6, IL8, IRAK2, PDPN, S100A8, SERPINA1, SPP1, TLR8, VNN1	3,67E-03	27/482	325/14116
Positive regulation of T cell activation / proliferation	AP3B1, BLM, CD83, IL1B, IL6, PDCD1LG2, PTPRC, SOCS5, VCAM1, VNN1	1,08E-03	10/482	76/14116
Antigen processing and presentation of peptide or polysaccharide antigen via MHC class II	FCER1G, HLA-DPA1, HLA-DQA1, HLA-DQB1, HLA-DRA, HLA-DRB1, HLA-DRB4, HLA-DRB5, IFI30	6,99E-03	8/482	33/14116
**Angiogenesis (31 different genes)**				
Vasculature development / blood vessel morphogenesis	ANGPT2, ANGPTL4, ANPEP, ARHGAP22, BGN, CD44, COL1A2, CXCR4, ECGF1, EREG, FGF2, GLMN, IL1B, IL8, ITGAV, MMP19, NRP1, PDPN, PTEN, PTK2, RASA1, ROBO1, TIPARP, VEGF	8,72E-03	25/482	211/14116
NA genes to angiogenesis function: ADAMTS6, HGF, MMP1, MMP3, MMP9, MMP12, MMP13, POSTN, SPP1				
**Proliferation and apoptosis (48 different genes)**				
Positive regulation of apoptosis	AHRR, BNIP3, BRCA1, CD44, CUL1, DYRK2, ETS1, GCH1, HIPK2, IL1B, KLF10, NR3C1, PDCD5, PLAGL1, PPP3R1, PREX1, PRKCA, PTEN, PTGS2, PTPRC, SMAD3, SOS1, TIAL1, TIAM1, TOP2A, TRIO, VDR	6,19E-02	29/464	430/13528
Regulation of mitosis	BIRC6, BUB1, C21ORF45, CCNK, CCNH, CDCA2, CDCA5, CDCA8, CDK2, ESPL1, HGF, HMGA2, KIF22, KIFC1, OIP5, SEPT2, TREX2, TXNL4B, UBE2C, ZWINT	4,90E-02	18/482	295/14116
Regulation of osteoclast differentiation	CA2, GNAS, KLF10	(2,22E-01)*	3/482	6/14116
**Extracellular matrix remodelation (31 different genes)**				
ECM-receptor interaction	CD44, COL1A2, COL4A2, COL6A3, FN1, IBSP, ITGA2, ITGAV, ITGB1, ITGB3, LAMC1, SPP1, TNC	1,08E-02	13/221	84/5085
Collagen	COL1A2, COL4A2, COL6A3, COL7A1, COL12A1, COL16A1	8,47E-02	6/499	35/15908
Metal-thiolate cluster	MT2A, MT1F, MT1G, MT1H, MT1M, MT1X, MT4	1,46E-04	6/210	12/19235
Collagen degradation	MMP1, MMP3, MMP9, MMP13, MMP19	1,20E-02	5/460	25/19235
NA genes to ECM remodelation function: CTSS, MMP12, PLAUR				
**Bone biology (22 different genes)**				
Skeletal system development	ACP5, ANKH, CHST11, CMKLR1, COL12A1, COL1A2, DLX1, EXT1, GNAS, HOXB2, IBSP, KLF10, POSTN, PRKCA, PTGS2, SMAD3, SPP1, TIPARP, TNFRSF11A, VDR, NT5A	4,88E-02	23/482	319/14116

Gene Term Linker					
***Silhouette width***	***Term***		***Genes***	***Adjusted p-value (FDR)***	***Count / total list***
**Chemokine response related function (34 different genes)**					
0.606	Chemotaxis Cytokine-cytokine receptor interaction Chemokine signaling pathway Chemokine activity Small chemokine C-X-C / Interleukin 8 NOD-like receptor signaling pathway		BIRC3, CCL2, CCL20, CCL3, CCL4, CCL7, CLCF1, CMKLR1, CXCL1, CXCL2, CXCL3, CXCL5, CXCL6, CXCR4, DOCK2, FGF2, GDF5, HGF, IFNGR2, IL16, IL1B, IL6, IL8, LIF, PDGFC, PLAUR, RAC2, RAF1, RNASE2, ROBO1, S100A8, SOS1, TNFRSF11A, TNFRSF21	3.98E-12	34/556
**Cell interaction/communication related function (51 different genes)**					
0.270	External side of plasma membrane Cell-matrix adhesion Integrin-mediated signaling pathway		ADAMDEC1, ADAMTS1, ANPEP, APAF1, BGN, BIRC3, CD44, CD83, CDC42, CDK2, CHI3L1, COL12A1, COL16A1, COL1A2, COL4A2, COL6A3, COL7A1, CR1, CTSB, DDX26B, DST, FGF2, FN1, HGF, IBSP, ITGA2, ITGAM, ITGAV, ITGB, ITGB1BP1, ITGB3, LAMC1, PARVA, PDGFC, PIK3R5, PLEK, PRKCA, PTEN, PTGS2, PTK2, PTPRC, RAC2, RAF1, RAPH1, SOS1, SPP1, TFPI2, TIAM1, TNC, VCAM1, XRCC5	3.16E-19	51/556
**Proliferation/apoptosis related function (37 different genes)**					
0.137	Regulation of apoptosis Response to estradiol stimulus		APAF1, BIRC3, BRCA1, CAPN3, CCL2, CD44, CD82, CDCA8, CDK2, DAPP1, DUSP6, GADD45B, GCH1, GNAS, GRIA3, IL1B, ITGA2, LAMC1, PARVA, PDGFC, PIK3R5, POLS, PPP2R2A, PRKCA, PTEN, PTGS2, PTK2, PTPN12, PTPN2, PTPRC, PTPRE, RRM2, SERPINA1, SOD2, SRP54, TOP2A, TRIB1	1.30E-14	38/556
**Other functions not annotated**					
***Function***		***Genes***			
MM plasma cell proliferation / drug resistance		CCL2, CCL3, CD44, EXT1, FN1, HAS2, HGF, IL1B, IL6, IL8, IL16			
Osteoclast formation and resorption		CCL2, CCL3, CCL4, CCL20, CHSY1, IL1B, IL6, IL32, LIF			
Inhibition of osteoblast differentiation and function		CCL3, EREG			

In addition, differentially upregulated genes in co-culture exclusive to pMSCs, included genes linked with statistical significance to RNA processing and splicing, activation of the ubiquitine-proteasome pathway, regulation of cell cycle, cellular response to stress as well as to the Wnt signaling pathway (Table 2). Enrichment analysis was also performed on downregulated genes of List II, again not revealing additional functional annotations to the ones observed with upregulated genes. Diminished expression of certain genes in List II (such as *CST3, FZD5*, *WNT3, C/EBPB*, and *GLI1)* could be associated to inhibition of OB function due to their reported osteogenic roles.

Manual curation was also used to reinforce coverage of bioinformatic tools and to add not annotated functions for genes with a reported role in myeloma pathogenesis (end of Tables 1 and 2).

**Table 2:** Functional signatures of upregulated genes due to co-culture with MM.1S and exclusive to pMSCs (List II, FDR < 0.02). Specific functional categories were assigned by DAVID Bioinformatics Resources 6.7, GeneTerm Linker or manual curation, with values of statistical significance, silhouette width (when applicable), together with a list of the differentially expressed loci associated with each function. Genes marked with an asterisk (*) refer to genes not initially included in the functional enrichment analysis due to a FDR > 0.02, but subsequently incorporated because of their potential role in myeloma.

DAVID Bioinformatics Resources 6.7					
***Term***	***Genes***	***Adjusted p-value (Benjamini)***		***Count / total list***	***Population hits/total***
**RNA processing (44 different genes)**					
RNA processing / RNA splicing	C1D, CCAR1, CPSF6, CSTF2, DHX9, DKC1, EXOSC8, FBL, FRG1, GRSF1, GTF2F2, KHDRBS1, NPM3, POLR2G, PPIH, PPIL1, PPP4R2, PTBP2, PUS1, QTRTD1, RBM17, RBM22, RBM3, RPL35A, RPP40, SF3B14, SF4, SFRS11, SFRS3, SLU7, SNRPB, SNRPB2, SNRPC, SR140, STRAP, SYNCRIP, TFB2M, U2AF1, UTP14A, WDR3, ZNF638	6,96E-07		42/371	547/14116
Ribonucleoprotein complex biogenesis	C1D, DKC1, EXOSC8, FBL, FRG1, GNL2, GTPBP4, NPM3, RPL35A, SLU7, SNRPB, SNRPC, TFB2M, UTP14A, WDR3	2,26E-02		15/371	180/14116
**Proliferation (53 different genes)**					
Cell cycle regulation	AKAP8, APC, ATM, BTRC, CCAR1, CEP72, CETN3, CUL2, DMC1, DMTF1, DTYMK, E2F3, ESCO2, EVI5, KHDRBS1, KPNA2, LIG4, MAPK13, MCTS1, MDC1, MDM2, MDM4, MTBP, NEK4, NIPBL, PDCD6IP, PPP1CB, PPP1CC, PPP3CB, PSMB1, PSMC6, RANBP1, RBBP8, RPS27A, SH3BP4, SUGT1, UHMK1, USH1C	1,99E-03	42/371		776/14116
NA genes to proliferation function: BUB1B*, BUB3*, CCNA2*, CCNB2*, CCNC*, CCNF*, CCNT2*, CDC2*, CDC20*, CDC45L*, CDC5L*, CDC6*, CDCA1*, CDCA7L*, CDK7*, CDK8*, CENPE*, CENPF*, KNTC1*, MAD2L1*, ZW10*, ZWILCH*					
**Ubiquitin proteasome pathway activation (35 different genes)**					
Cellular response to stress	APC, ATG10, ATM, CASP3, CIDEB, DCLRE1C, DHX9, ESCO2, EYA3, FGD4, HDAC2, HMGB1, JUN, LIG4, MCTS1, MDC1, PPP1CB, PRKDC, RBBP8, RBX1, RFC4, SLK, UBE2N, UBE2V2, WRN	3,16E-02	30/371		566/14116
Protein ubiquitination	CAND1, CBL, CBLL1, MDM2, MIB1, RBBP6, RPS27A, UBE2D2, UBE2N, UBE2V2, UBE3A	2,24E-02	12/371		119/14116

**Gene Term Linker**					
***Silhouette width***	***Term***		***Genes***	***Adjusted p-value (FDR)***	***Count / total list***
**RNA processing (15 different genes)**					
0.612		Spliceosomal complex Spliceosome	CCDC12, FRG1, PPIH, PPIL1, RBM22, SF4, SFRS3, SLU7, SNRPB, SNRPB2, SNRPC, SR140, STRAP, SYNCRIP, U2AF1	2.97E-09	17/464
**Ubiquitin proteasome pathway activation and oncogenic events (34 different genes)**					
0.232		Protein ubiquitination Ubiquitin-protein ligase activity Ubiquitin mediated proteolysis Chronic myeloid leukemia Ubiquitin-dependent protein catabolic process Endocytosis Prostate cancer Cell cycle	ATM, BTRC, CAND1, CBL, CBLL1, CHMP5, CHUK, CREB3, CUL2, E2F3, HDAC2, MDM2, MIB1, PDCD6IP, PIAS2, PRKDC, PSMC6, RBBP6, RBX1, STAT5B, TCEB1, TCF7, TGFBR2, UBE2D2, UBE2J1, UBE2N, UBE2V2, UBE3A, USP13, USP24, USP4, VPS25, VPS37C, YWHAQ	9.05E-12	36/464
**Wnt signaling pathway (11 different genes)**					
0.584		Wnt signaling pathway	APC, FBXW1, JUN, NFATC2, PPP3CB, PRICKLE2, RBX1, ROCK1, TCF7, WNT16, WNT7A	3.08E-05	11/464

NA genes to Wnt signaling: NDP	
**Other functions not annotated**	
***Function***	***Genes***
MM plasma cell proliferation / drug resistance	ANXA1*, ANXA2P1, ANXA2P2, ANXA2P3, NRG3, WNT16
MSC immunomodulation	PTGER2*, PTGES3*
MSC survival/pro-inflammation	BIRC5*, HMMR*
Osteoblast inhibition	HES1, CALD1, NFATc2
Various	ABCC4*, NDP, WNT16

### Putative roles of CXCL1, CXCL5 and CXCL6 chemokines in myeloma pathophysiology

**Figure 3:**
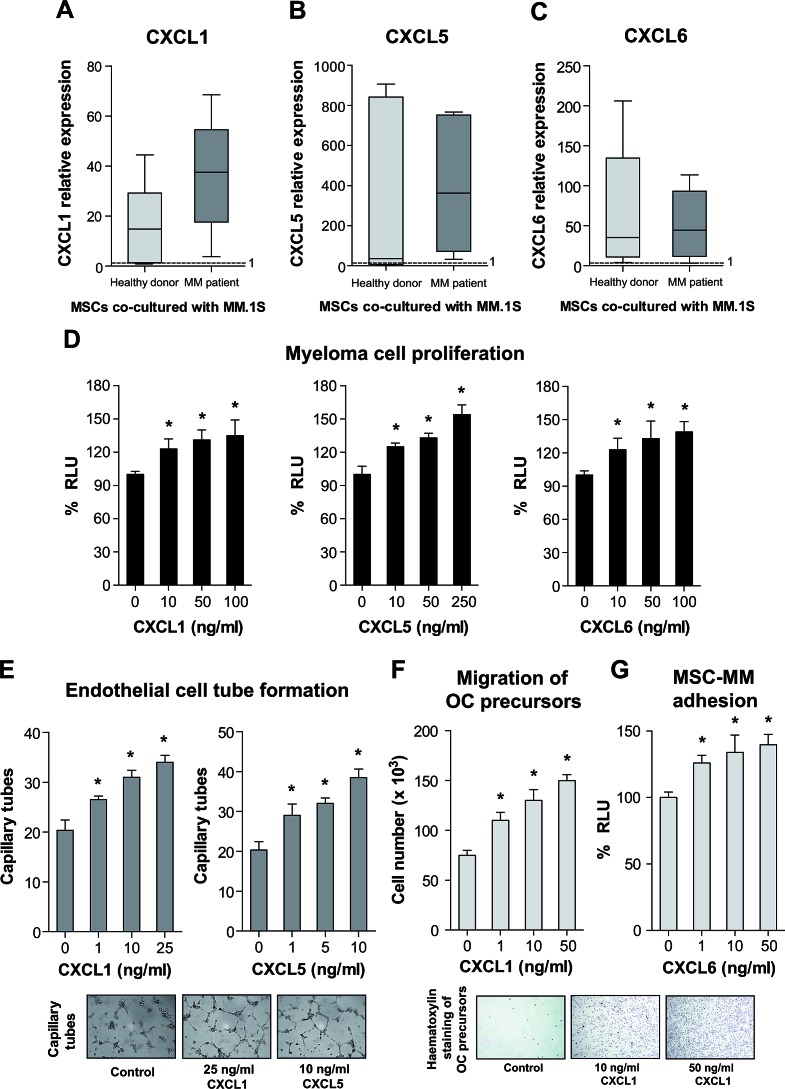
Expression of *CXCL1*, *CXCL5* and *CXCL6* by real-time PCR (A-C) and functional activities on myeloma cell proliferation (D), endothelial tube formation (E), migration of OC precursors (F) and MSC-MM cell adhesion (G) **A-C**, Expression levels for each gene were evaluated in dMSCs and pMSCs both in mono-culture or after 24 hour co-culture with the MM.1S cell line (n=7 for each type of MSC and culture condition), and normalized to GAPDH levels for each sample. Box plots represent fold-induction of gene expression in the co-culture condition relative to gene expression in mono-culture. **D**, MM.1S-luc cells were grown in 0.1% FBS containing medium in the presence or absence of specified concentrations of rh CXCL1, CXCL5 and CXCL6, and bioluminescence was measured after 3 days of culture. **E**, BMEC-1 cells were seeded on a Matrigel surface in medium containing 0.1% FBS and the specified chemokine concentrations for 5 hours. Tubule-like structures were counted under the microscope with the aid of a grid and micrographs are representative of maximal effects observed for each chemokine. **F**, Migration assays were performed by placing OC precursors in serum-limited conditions in the upper chamber and specified concentrations of rh CXCL1 diluted in the same medium in the lower chamber. Micrographs show representative CXCL1-mediated chemotaxis of OC precursors to the lower chamber after a 6 hour incubation time. **G**, MM.1S-luc cells were seeded on a monolayer of d/pMSCs in serum-free medium; after 3.5 hours, non-attached cells were removed by gentle PBS washes and bioluminescence signal measured. *p < 0.05.

The high level of expression and significance of three C-X-C motif chemokines (*CXCL1*/*GROα*, *CXCL5*/*ENA-78* and *CXCL6*/*GCP-2*) selected from List I was particularly interesting due to the known function of chemokines as regulators of multiple physiological and pathological processes. In agreement with our microarray data, *CXCL1*, *CXCL5* and *CXCL6* mRNA levels were shown to be highly upregulated in MSCs from both origins after co-culture (Fig. 3A-C).

The signaling receptor for CXCL1 and CXCL5 is CXCR2, while CXCL6 signals both through CXCR1 and CXCR2 [28]. Previous studies have demonstrated the presence of CXCR1 and CXCR2 receptors on MM cell lines and plasma cells from MM patients [29], suggesting that our selected chemokines may act as paracrine factors upon neoplastic plasma cells. We first tested the effect of recombinant human (rh) CXCL1, CXCL5 and CXCL6 on proliferation of the multiple myeloma MM.1S-luc cell line. As shown in Fig. 3D, the three chemokines dose-dependently and significantly increased the proliferation rate of MM.1S-luc cells by maximal effects of 39%, 54% and 35%, respectively.

A well-stablished role for several members of the CXC chemokine family is the promotion of angiogenesis [30]. When we tested the putative angiogenic potential of the three chemokines, an increasing dose-response in the tubule formation assay was found for CXCL1 and CXCL5, even at lower concentrations than those required to induce myeloma growth (Fig. 3E). Since IL8 has been reported as an angiogenic factor in MM [31], is highly overexpressed by d/pMSCs after co-culture (Supp. Table S2) and also signals through CXCR1 and CXCR2 [28], we explored the possibility of CXCL1 and CXCL5 presenting a synergistic angiogenic effect with IL8. Combination of sub-maximal concentrations of CXCL1 and CXCL5 with IL8 seemed to render at least an additive effect in the endothelial tube formation assay (see Supp. Fig. S1).

MBD is characterized by a double component of increased OC formation and resorption, whereas OB formation and function are impaired [7, 8]. Although we did not find any significative effect of CXCL1, CXCL5 or CXCL6 on the osteogenic or osteoclastogenic processes (data not shown), we observed that CXCL1 functioned as a chemoattractant for OC precursors (Fig. 3F), which express CXCR1 and CXCR2 receptors [32]. In the myeloma setting, this may favour their accumulation at sites of myeloma growth, where they could eventually differentiate to functional OCs. Finally, only CXCL6 moderately, but significatively, increased the adhesion of the MM.1S cell line to either d/pMSCs (Fig. 3G).

### Neuregulin-3

**Figure 4:**
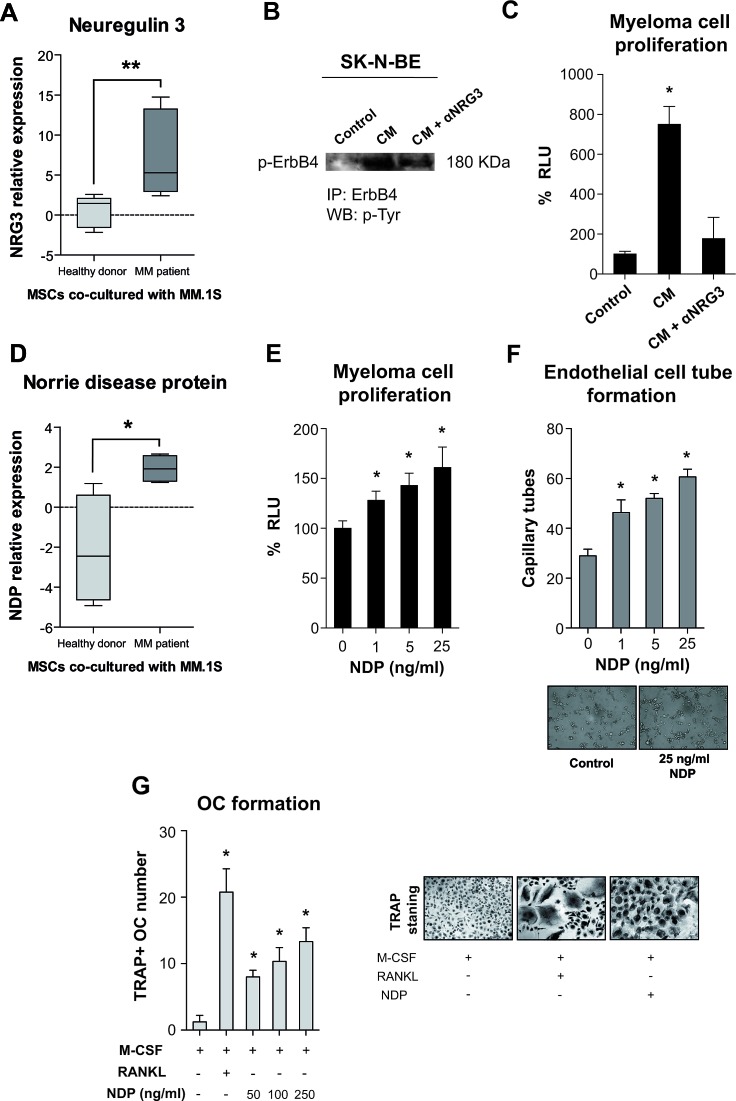
Expression of *NRG3* and NDP (A, D) and functional activities of NRG3 (B, C) and NDP (E-G) in relation to myeloma pathophysiology **A, D,** Expression of NRG3 and NDP in dMSCs and pMSCs co-cultured for 24 hours with the MM.1S cell line relative to that in mono-culture as assessed by real-time PCR and normalized to GAPDH levels for each sample (n=7 for each type of MSC and culture condition) *p < 0.05 **p < 0.01 between dMSCs and pMSCs. B, Secreted NRG3 from MM.1S and pMSC direct co-cultures was able to activate the ErbB4 receptor in the SK-N-BE neuroblastoma cell line. Conditioned media (CM) from MM.1S-pMSC direct co-cultures was concentrated ~ 20 fold and added to overnight serum-deprived SK-N-BE cells in the presence or absence of neutralizing anti-NRG3 antibody. After 10 min, protein extracts were immunoprecipitated with the anti-ErbB4 antibody and immunoblotted with an antibody specific for p-Tyr. C, NRG3 shows activity as a myeloma growth factor augmenting the proliferation of the RPMI8226-luc myeloma cell line. The multiple myeloma cell line RPMI8226-luc was cultured in serum-limiting medium (0.1% FBS), half supplemented with concentrated CM from MM.1S-pMSC co-culture with or without neutralizing anti-NRG3 antibody as specified, and bioluminescence measured after 72 hours. **E**, Recombinant NDP dose-dependently promoted the growth of the RPMI8226-luc cell line grown in serum-limiting conditions (0.1% FBS) for 72 hours. **F**, NDP induced the formation of tubule-like structures on the BMEC-1 cell line. BMEC-1 cells were seeded on a Matrigel-coated surface in serum-limiting conditions in the presence of specific concentrations of recombinant NDP; after 5 hours, formation of tubule-like structures was assessed. **G**, PBMCs from healthy donors were cultured in osteoclastogenic medium for 21 days containing M-CSF only, M-CSF and RANKL, or M-CSF and different NDP concentrations; OC formation was quantified by TRAP+ staining of multinucleated (≥ 3 nuclei) cells. *p < 0.05. Representative micrographs are shown.

The expression and/or activation of the epidermal growth factor (EGF) family of tyrosine kinase receptors, such as ErbB receptors, has been shown to be implicated in normal plasma cell differentiation and in myeloma biology [33, 34]. Neuregulin-3 (NRG3), a member of the EGF family ligands, was selected from List II and its increased expression after co-culture only in pMSCs was validated by real-time PCR (Fig. 4A). To test the specific capacity of NRG3 to activate its unique ErbB4 receptor and to function as a myeloma growth factor, we obtained the conditioned medium (CM) of MM.1S and pMSCs direct co-cultures. SK-N-BE neuroblastoma cells, which show high expression of the ErbB4 receptor, were exposed to this CM and NRG3-specific ErbB4 activation was observed after appropriate inmmunoprecipitation and immunoblotting procedures (Fig. 4B). This CM was also able to increase the growth rate of the RPMI8226-luc cell line, being its proliferating activity in great part dependent on NRG3 (Fig. 4C).

### Norrie Disease Protein

The Norrie disease protein (NDP) is a secreted protein recently identified as a non-conventional Wnt ligand capable of activating the canonical Wnt/β-catenin signaling pathway, via specific binding to frizzled receptor FZD4 and low density lipoprotein related LRP5/6 co-receptor [35]. In accordance with our data in List II, real-time PCR showed NDP to be highly overexpressed in co-cultured pMSCs, whereas it was even repressed in dMSCs after co-culture (Fig. 4D). In this study, we show that rh NDP was able to promote the growth of the RPMI8226-luc cell line (Fig. 4E) and to induce the formation of capillary tubes in the BMEC-1 cell line (see Fig. 4F) in a significative and dose-dependent manner. Moreover, NDP was able to induce osteoclastogenesis from peripheral blood mononuclear cells of healthy subjects in a dose-dependent manner in the presence of M-CSF (macrophage colony-stimulating factor) and absence of RANKL (receptor activator of nuclear factor κ B ligand) (Fig. 4G).

### Co-culture of bone marrow d/pMSCs with other myeloma cells and leukemia cell lines

**Figure 5:**
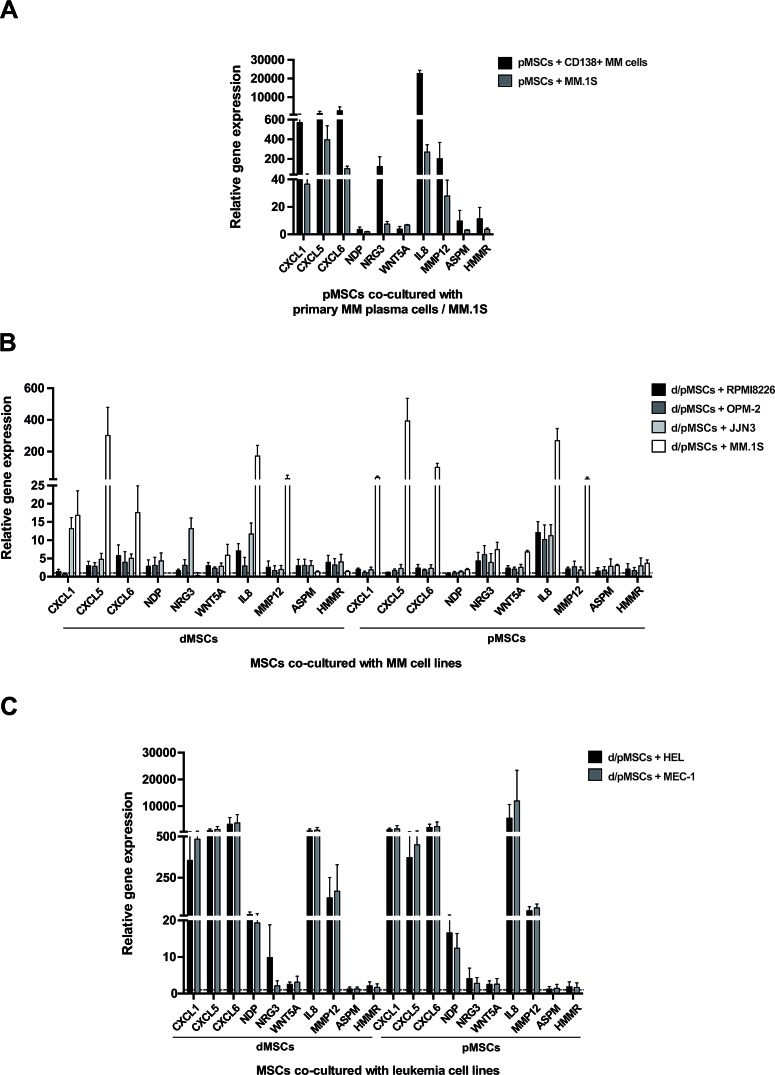
Expression of *CXCL1*, *CXCL5*, *CXCL6*, *WNT5A*, *IL8*, *MMP12* (from List I), and *NDP*, *NRG3*, *ASPM*, *HMMR* (from List II) by real-time PCR in d/pMSCs after co-culture with MM cells and leukemia cell lines **A**, Co-cultures of pMSCs (n=3) and primary CD138^+^ myeloma cells (n=2) were established following the same experimental settings as those with the MM.1S cell line. Expression values for each sample were normalized to GAPDH levels, and the relative expression of the selected genes in pMSCs in the co-culture condition was referred to that in mono-culture arbitrarily set as 1. **B**, Co-cultures of dMSCs (n=3) and pMSCs (n = 3) with the RPMI-8226, OPM-2 and JJN3 myeloma cell lines were performed in the same manner as those previously established with the MM.1S cell line. After normalization to GAPDH levels, fold-induction in MSCs in the co-culture condition was represented relative to gene expression in mono-culture set as 1 (dashed line). C, In a similar manner, relative expression of the set of genes in MSCs was obtained after co-cultures of dMSCs (n=3) and pMSCs (n=3) with the HEL (erythroleukemia) and MEC-1 (chronic B lymphocytic leukemia) human cell lines. Bars in all graphs illustrate mean values ± SEM.

In order to get a cue of whether the observed GEPs in d/pMSCs after interaction with the MM.1S cell line were representative of transcriptional changes in MSCs in the myeloma context, co-cultures were performed with primary CD138^+^ myeloma cells and with other established MM cell lines (i. e. RPMI8226, OPM-2 and JJN3). The expression levels of the 5 selected genes in our study (i.e. *CXCL1*, *CXCL5*, *CXCL6*, *NRG3* and *NDP*) together with the expression of 5 additional deregulated genes from our analyses [*WNT^5^A*, *IL8*, *MMP12* (matrix metallopeptidase 12) -from List I-; and *ASPM* (abnormal spindle homolog, microcephaly associated) and *HMMR* (hyaluronan-mediated motility receptor) -from List II-], were evaluated by real-time PCR (Figs. 5A, B). As observed in Fig. 5A, an upregulated expression of the 10 genes under study was observed in pMSCs when they were co-cultured with CD138^+^ myeloma cells, with even higher fold changes in co-culture than with the MM.1S cell line. A more heterogeneous response was observed, however, in d/pMSCs co-cultured with the RPMI8226, OPM-2 or the JJN3 cell lines (Fig. 5B). Although increased expression levels were evident for most of the genes under study, fold changes varied relative to co-culture with the MM.1S cell line. Also, the “pMSC exclusive” condition of genes from List II (*NDP*, *NRG3*, *ASPM* and *HMMR*) was lost in co-cultures with these other MM cell lines, since increased expression was observed on both dMSCs and pMSCs.

To test whether the gene expression changes in BM MSCs also occurred after interaction with other types of tumor cells, the relative expression levels of our set of genes was evaluated in d/pMSCs after co-culture with HEL (erythroleukemia) and MEC-1 (chronic B lymphocytic leukemia) human cell lines (Fig. 5C). These two cell lines were selected as representatives of acute myeloid leukemia (AML) and chronic lymphocytic leukemia (CLL) tumor cells, since similarly to MM, these two hematologic malignancies course with accumulation of tumor cells in the BM microenvironment. Of interest, an exacerbated expression of some of the evaluated genes (i. e. *CXCL1, CXCL5, CXCL6, IL8*) and strong expression of others (i. e. *NDP, NRG3, MMP12*) was observed in d/pMSCs after co-culture with the leukemic cell lines, which is suggestive of a specific role for these molecules in the biology of these hematological tumors. Other genes from List II (i. e. ASPM and HMMR) did not greatly vary their expression in co-culture with HEL or MEC-1cell lines with respect to mono-culture conditions, perhaps insinuating a more exclusive role of these molecules in MM pathophysiology.

### Transcription factor profile analysis

The analysis of putative transcription factor binding sites (TFBS) in the promoter regions of our deregulated genes in MSCs after co-culture (Lists I and II), allowed to infer the main TFs which might be regulating the observed changes in gene expression after co-culture with the MM.1S cell line (Supp. Table S4A). TFs with the highest number of TFBS and gene hits were Elk1 [36] and Gfi1 [37] in List I; and Elk4 [36] and GABPA [38] in List II. Interestingly, in the myeloma context, Gfi1 has recently been pointed out as a new transcriptional repressor of Runx2, blocking OB differentiation and being increased both in MSCs from MM patients and MM-bearing mice [24]. Among the multiple target genes regulated by these four transcription factors, many have been reported to be critical for MM pathogenesis (Supp. Table S4B).

**Figure 6:**
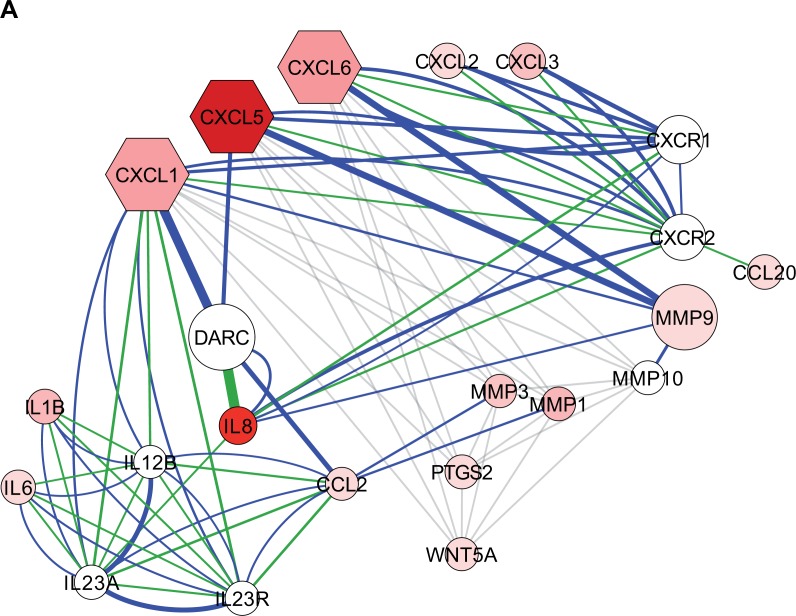

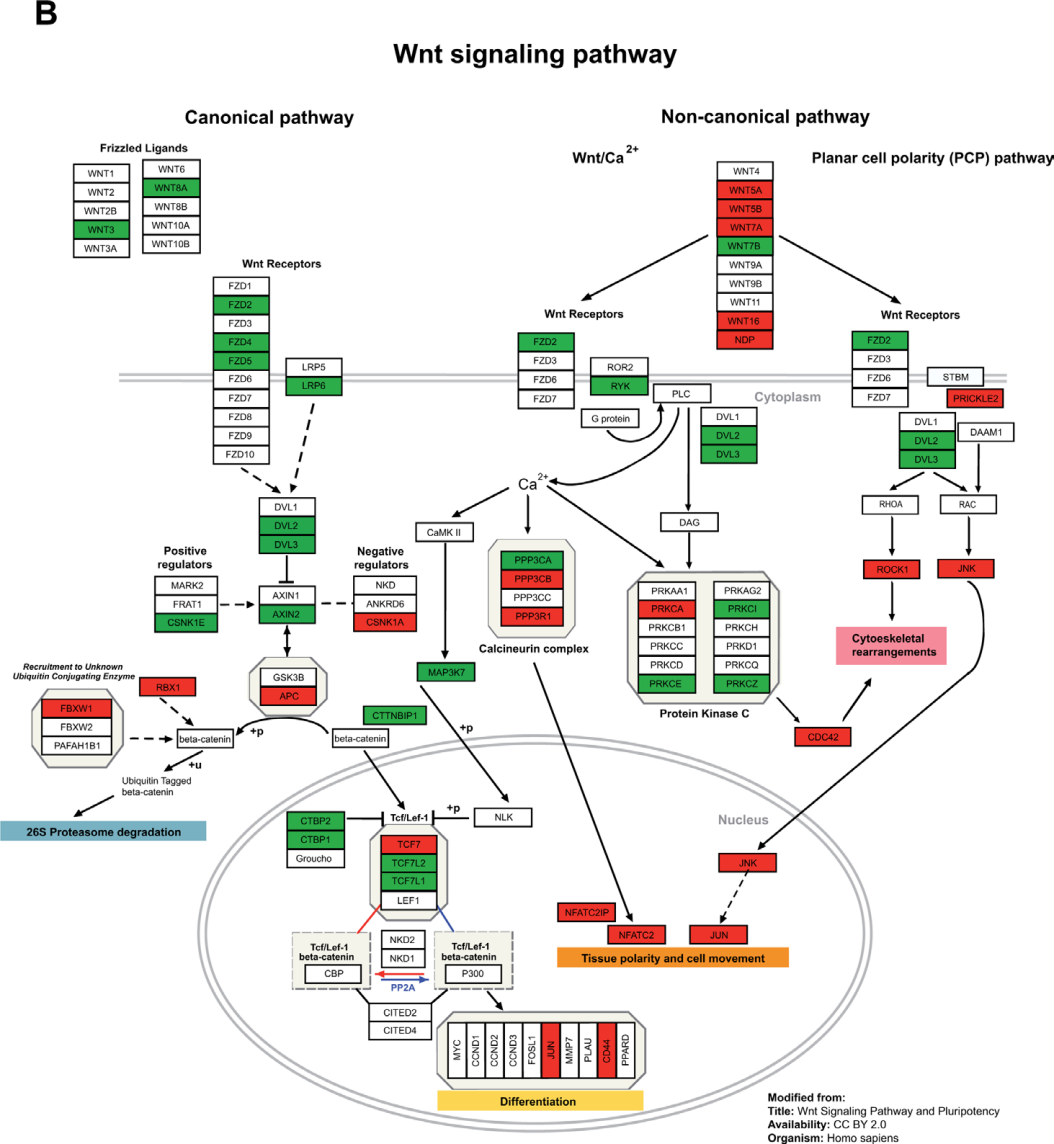
Schematic representations for deregulated genes in MSCs after interaction with MM.1S cells **A**, Predicted network when entering CXCL1, CXCL5 and CXCL6 genes from List I into the GeneMANIA and Cytoscape plugin allowing physical, signaling pathway and co-expression interactions. Node shape is hexagonal for the three query genes and circular for the rest of the predicted nodes of the network, whereas node color is proportional to its average fold change (FC) after co-culture with the MM.1S cell line (brighter red: higher FC after co-culture with respect to monoculture; white: absent in List I). Size of the nodes is indicative of its significance in the predicted network as determined by type and number of interactions. Physical interactions are depicted in blue, signaling pathway interactions in green and co-expression interactions in light gray; the strength of these interactions being indicated by line width. **B**, Schematic representation of Wnt canonical and non-canonical signaling, modified from www.Wikipathways.org. Upregulated genes on Lists I and II are shown in red, whereas downregulated genes on Lists I and II are coloured in green.

## DISCUSSION

During the last two decades, the importance of the tumor microenvironment for the acquisition of cancer capabilities by neoplasic cells and thus for cancer progression, has become increasingly clear [39, 40]. In the case of MM, the well-organized cellular and functional architecture of the BM is converted into a “privileged” tumor microenvironment greatly supporting the growth, progression, survival and drug resistance of myelomatous cells [3]. Therefore, the design of better and more effective anti-myeloma treatments partly relies on a better understanding of the reciprocal interactions of myeloma and tumor microenvironment cells in the BM. Being MSCs a relevant component of the tumor microenvironment in MM, in this study we have specifically focused on the “not so much studied” gene expression changes in MSCs induced by interaction with myeloma cells; these changes in MSCs further contribute to myeloma growth, survival and progression and compromise normal MSC function (e. g. contributing to the reduced pMSC osteogenic potential). It should be noted that the ability to induce gene expression changes in BM derived-MSCs is not exclusive of myeloma tumor cells. In fact, specific gene expression changes were observed for a subset of 10 selected genes from our study when d/pMSCs were co-cultured with acute myeloid and chronic lymphocytic leukemica cell lines (see Fig. 5C). Our piece of data not only gives further evidence of the important role of the BM stromal microenvironment in these hematological malignancies [41-44], but also opens the door for future research based on genome-wide expression studies in BM stromal cells interacting with leukemia cells.

In our study, we specifically illustrate a comprehensive transcriptomic analysis of BM-derived MSCs (from healthy donors and MM patients) after establishment of both cellular and molecular interactions with the MM.1S myeloma plasma cell line. We believe that the GEP of MSCs after interaction with the MM.1S cell line may be representative of gene expression changes as occurring in pMSCs in the BM of myeloma patients. In fact, a similar relative expression for the commented 10 selected genes from our analyses was observed in pMSCs after co-culture with either the MM.1S cell line or primary myeloma cells (Fig. 5A). However, it should also be acknowledged that some differences in gene expression changes for the same subset of genes were observed in d/pMSCs co-cultured with different MM cell lines (Fig. 5B). This may be indicative of qualitative and/or time-course differences in gene expression changes induced in MSCs when interacting with different types of myeloma cells.

Apart from a better understanding of the complex interactions between myeloma cells and MSCs, our analyses intended to identify deregulated pathways in MSCs due to these interactions which may influence the progression, therapeutic resistance, dissemination of MM and/or development of MBD. We also aimed to gain some insight into the evolution of the disease by determining which myeloma-induced transcripts were common to dMSCs and pMSCs and which were deregulated exclusively in pMSCs.

### Deregulated genes after co-culture common to dMSCs and pMSCs (List I)

The differentially upregulated genes in List I were functionally linked to: i) a chemokine/cytokine response, since these molecules have effectively been implicated in several aspects of MM biology (myeloma cell proliferation and survival, homing and/or drug resistance, angiogenesis, OC precursor recruitment and bone destruction) [1, 2, 28]; ii) an immune and inflammatory response, which could be related to the reported diminished immunomodulatory potential of pMSCs [18]; iii) angiogenesis (e. g. *ANGPTL4, FGF2*) [13, 31]; iv) microenvironment cross-talk, including cell-matrix adhesion (e. g. *COL12A1*, *ITGB1*) or integrin-mediated and extracellular matrix remodelation (e. g. *ITGA2, ITGAM*, *VCAM1, MMP1*, *MMP3*, *MMP9*, *MMP12*) [3, 45, 46]; and v) bone biology and skeletal development [(e. g. *SPP1* (osteopontin) [47]]. Manual curation based on the literature, further revealed functions of these deregulated genes related to myeloma growth and drug resistance (e. g. *IL6*, *CCL3*, *HGF*, *FN1*) [3, 20, 48], OC formation and/or activation (e. g. *CCL3*, *CCL20, LIF*) [26, 48, 49] and inhibition of OB differentiation and function (e. g. *CCL3*, *EREG*) [50, 51]. Other molecules identified as promoters of OB differentiation and/or function in myeloma (e. g. *EFNB2*, *HMOX1*) [52, 53] and in other contexts (e. g. *CD276,* BMP7, *IL6ST*) [54-56], showed a downregulated expression after co-culture (Supp. Table S2).

As observed in our functional *in vitro* assays, the three selected highly upregulated chemokines in co-cultured d/pMSCs (i. e. CXCL1, CXCL5 and CXCL6) supported important roles in myeloma growth, angiogenesis, recruitment of OC precursors and myeloma-cell adhesion. In fact, serum levels of CXCL1 and CXCL5 chemokines have been found to be significantly elevated in MM patients, and in the case of CXCL1, to increase with disease stage [57]. Serum concentrations of CXCL1 and CXCL5 in myeloma patients were below the concentrations used in our *in vitro* studies (186.5 ± 129.1 pg/ml and 765 572.1 pg/ml, respectively [57]). However, higher physiological concentrations of these cytokines are expected to be found in the BM at sites of MSC-myeloma cell interactions, which would support their angiogenic and myeloma growth activities. When we entered these three genes as a query in the GeneMANIA-Cytoscape tool, an association network of 22 genes was represented (Fig. 6A). Importantly, 16/22 of the nodules appearing in the network were included in List I. Some of the nodules not present in List I (e. g. CXCR1, CXCR2 and DARC) corresponded to common receptors for the three cytokines, suggesting a paracrine activity of the secreted CXCL chemokines on myeloma cells or other cells in the BM microenvironment. Represented interactions of these chemokines and matrix metalloproteinases (MMP9, MMP1 and MMP3) may be related to ECM remodeling associated to chemokine activity; alternatively, these interactions may respond to posttranslational cleavage of the chemokines to modify their biological potency [58]. Other nodules in the network correspond to cytokines with known function in myeloma pathophysiology and/or in osteolytic lesions (IL6, IL1β, CCL2, IL8 and CCL20) [8, 28, 59]. Specifically, *IL8*, being one of the most highly overexpressed genes in co-cultured d/pMSCs in our study, has been found to be elevated in serum of MM patients [31]. IL8 expression in myeloma has been related not only to increased angiogenesis [31], but also to proliferation and chemotaxis of myeloma cells [60] and stimulation of osteoclastogenesis and bone resorption [61]. Another upregulated gene in co-cultured MSCs and shown in the network is that corresponding to the non-canonical Wnt ligand *Wnt5A*. Wnt5a has been identified by GEP analyses as a myeloma growth factor expressed in myeloma cells and OCs from myeloma patients [20]. Although Wnt5a has been reported to promote osteoblastogenesis of human MSCs through Fzd receptors and Ror2 [62], it has been found that myeloma cells in co-culture with pre-OBs inhibit Ror2 expression in the latter, thus impairing osteogenic differentiation and contributing to OB suppression [63]. Interestingly, increased osteoclastogenesis has been found to be mediated by signaling between Wnt5a (secreted by OB-lineage cells) and the membrane receptor Ror2 (expressed in OC precursors) [64], which suggests that the Wnt5a-Ror2 axis may contribute to increased OC formation and bone resorption in MBD.

### Deregulated genes after co-culture exclusive to pMSCs (List II)

Two of the major functions associated to deregulated genes in List II were RNA processing and activation of the ubiquitin-proteasome pathway (UPP). These functions probably reflect the response of pMSCs to cope with the high transcriptional and protein secretory load after myeloma cell interaction. Upregulated expression of genes implicated in mRNA maturation and disposal of misfolded proteins would likely be necessary for pMSCs to maintain an adequate cellular function. Interestingly, upregulated expression of transcripts from the UPP were also found in the transcriptional signature of myeloma cells in the presence of stromal cells [65].

Wnt signaling is a critical pathway for OB differentiation and bone metabolism [66, 67] which is also deregulated in pMSCs after co-culture. Commitment of MSCs to osteogenic differentiation and to promotion of bone formation has majorly been associated to activation of the Wnt canonical pathway [66, 68]; however, mounting evidence is arising for non-canonical Wnt signaling also regulating OB differentiation and function [69-72]. Altogether, our analyses seem to point out an inhibition of the canonical Wnt signaling in co-cultured pMSCs (see Fig. 6B), both because of upregulated expression of several non-canonical Wnt ligands (*WNT5A, WNT5B* -from List I-, *WNT7A* and *WNT16* -from List II-), and because of upregulated expression of negative regulators of β-catenin (*APC*, *RBX1* and *FBXW1*) which would promote β-catenin ubiquitination for proteasome degradation [73]. Besides, the upregulated expression of some members of the non-canonical Wnt/Ca^2+^ and Planar Cell Polarity pathways (e. g. *CDC42* and *ROCK1*), may perhaps indicate a possible enhancement of the migration and invasiveness properties of co-cultured pMSCs as some authors have described in other cell types [74].

Both d/pMSCs after co-culture show a deregulated expression of genes involved in proliferation and apoptosis. More specifically, pMSCs after interaction with MM.1S cells also present upregulated expression of genes implicated in cell cycle regulation (including numerous mitotic spindle components as well as interphase and mitosis checkpoints; see Tables 1 and 2, and Supp. Tables S2 and S3 [75]). Besides, deregulated genes in co-cultured pMSCs present functional association with cellular response to stress (including genes transduction to oxidative-stress stimuli, endoplasmic reticulum stress and DNA damage response; Table 2). Taken together, interactions with myeloma cells seem to greatly influence the proliferative potential of MSCs, which may be related to the reduced growth rate of pMSCs and/or the premature onset of senescence observed in pMSCs when grown *in vitro* as compared to dMSCs [15, 17]. Further gene expression and functional studies on MSCs with longer co-culture times are warranted to shed light onto these issues.

Other genes exclusively upregulated in pMSCs and not directly included in the commented functions (Table 2, “Other functions not annotated”), might be implicated in several aspects of myeloma pathophysiology, such as myeloma cell adhesion and growth (e. g. *ANXA2P1*, *ANXA2P2* [23]), or inhibition of OB differentiation (e. g. *GFI1* [24]). The downregulated expression of several genes in pMSCs after co-culture (e. g. *CST3* [76], *FZD5* [72], *WNT3* [77], *C/EBPB* [78], and *GLI1* [79]; see Supp. Table S3) may also contribute to the reported impairment of OB function in MM.

The putative role of the upregulated expression of *NRG3* and *NDP* from List II was also explored.** NRG3 and its receptor have been found to be expressed in myeloma cells but not in their normal counterparts [34]. Besides, NRG3 has been reported to be significantly overexpressed by myeloma cells as compared to other cells in the BM microenvironment [20].** In this study, we show that pMSCs co-cultured with myeloma cells also secrete NRG3, which is able of activating ErbB4 and to promote myeloma proliferation, thereby creating a paracrine amplification loop for myeloma growth. On the other hand, our experiments showed that rh NDP increased the growth of a MM cell line and induced the formation of capillary tubes, being the latter capability in line with the reported function of NDP in the control of formation of retinal capillaries [35]. Interestingly, we also showed that NDP increased OC formation in the presence of M-CSF and absence of RANKL. Since as far as we know NDP has not been related to myeloma, our data suggest a putative role for this factor in the pathophysiology of the disease and in the development of ostelytic lesions.

Some authors have proposed the idea of a co-evolution of MSCs and myeloma cells along the course of the disease [3, 11]. In this sense, dMSCs would progressively convert to pMSCs, being the interactions with myelomatous cells determinant for those changes. Consistent with this hypothesis, common deregulated genes in MSCs after 24 hours of co-culture with MM.1S cells may daringly be considered as gene expression changes occurring in MSCs at initial phases of myeloma (i. e. production of myeloma growth and angiogenic factors, chemokines, chemoattraction of OC precursors, OC activation and OB inhibition). Following the same line of reasoning, genes deregulated after co-culture exclusively found in pMSCs, may perhaps be considered as expression changes predominantly affecting proper MSCs at latter stages of myeloma disease (i. e. changes in MSCs to cope with enhanced protein secretion, cellular response to stress, and regulation of Wnt signaling).

In summary, we have shown that the transcriptomic profile of pMSCs co-cultured with myeloma cells greatly differs from that of MSCs in mono-culture, may better reflect the expression signature of MSCs in the BM of myeloma patients, and provides new insights to the contribution of interacting MSCs to the pathophysiology of MM and MBD. Our data also bring up the issue that due to interactions with myeloma cells, MSCs are modified from a dMSC phenotype to one bearing the prototypic characteristics of pMSCs. Further, our studies support that therapeutic targeting of myeloma-stromal cell interactions would benefit from both an anti-myeloma effect and from maintenance of appropriate MSC function.

## METHODS

### Samples and Ethical statements

BM samples from 12 healthy donors and 19 newly diagnosed MM patients were obtained after written informed consent of participants, and used for MSC isolation and expansion (d/pMSCs) or for isolation of CD138^+^ primary plasma cells.

Research was conducted in accordance to ethical standards and principles expressed in the Declaration of Helsinki. The study was approved by the Institutional Review Board from the Centro de Investigación del Cáncer, IBMCC (University of Salamanca-CSIC, Spain).

### Reagents and immunochemicals

Recombinant human (rh) CXCL1, CXCL5, CXCL6, M-CSF and RANKL were purchased from PeproTech and rh NDP from R&D Systems. Primary antibodies were produced in-house (ErbB4) or purchased from Santa Cruz Biotechnology (p-Tyr, NRG3). Trypan Blue solution 0.4% was delivered by Sigma-Aldrich, cell culture media and supplements by Gibco (Life Technologies), and epidermal growth factor (EGF) and Matrigel from BD Biosciences.

### Cells and culture conditions

#### Cell lines

The human MM cell line MM.1S was provided by Dr ST Rosen (Northwestern University, Chicago, IL, USA), whereas the MM.1S-luc cell line was donated by Dr CS Mitsiades (Dana-Farber Cancer Institute, Boston, MA, USA). RPMI8226 cells purchased from the American Type Culture Collection (ATCC) were lentivirally transduced to stably express firefly luciferase as in Groen et al. [80]. Other established MM cell lines (OPM-2, JJN3), together with the erythroleukemia (HEL) and chronic B lymphocytic leukemia (MEC-1) cell lines were purchased from the Leibniz Institute DSMZ biosource center. All hematological tumor cell lines were periodically authentified by STR DNA profiling, and grown in RPMI 1640 medium or IMDM (MEC-1) supplemented with 10% heat-inactivated fetal bovine serum (FBS), 100 U/mL penicillin, 100 μg/mL streptomycin and 2 mM L-glutamine.

The BM endothelial cell line BMEC-1 was provided by Dr FJ Candal (Centers for Disease Control and Prevention, Atlanta, Georgia, USA). This cell line was grown in MCDB 131 medium containing 15% FBS, 2.5 μg/ml amphotericin B, 1 μg/ml hydrocortisone, 10 ng/ml EGF and antibiotics. The human neuroblastoma cell line SK-N-BE was purchased from ATCC and grown in DMEM medium (4.5 g/l glucose) with 10% FBS and antibiotics.

#### Primary mesenchymal stromal cells, osteoblasts, macrophages and osteoclasts

Primary MSCs from BM samples of healthy donors (n=11) and MM patients (n=17) were isolated and expanded as described by Garayoa *et al*. [14]. At passage 2, d/pMSCs were used in co-culture experiments with the MM.1S cell line and subsequently subjected to GEP analyses (clinical characteristics of patients used are shown in Supp. Table S1). In addition, some d/pMSCs were co-cultured with leukemia cell lines or primary MM cells, or utilized in OB differentiation experiments.

Methods for *in vitro* OB (n=4) and OC (n=4) differentiation have been previously reported [81]. Briefly, OC precursors were generated from PBMCs of healthy donors in α-MEM with 10% FBS and antibiotics and supplemented with 25 ng/ml M-CSF for 4 days; OBs were differentiated from primary MSCs by culture in osteogenic medium containing β-glycerol phosphate, ascorbic acid and dexamethasone.

#### Primary CD138+ plasma cells

Two BM samples from untreated myeloma patients at diagnosis were used to isolate primary myeloma cells via CD138-positive selection using the AutoMACs separation system (Miltenyi-Biotec) with a final purity > 95%. These primary myeloma cells were subsequently used in co-culture studies with pMSCs.

#### Co-culture system

Co-cultures were performed using a 6-well format transwell system with 1 μm pore size membrane (BD Biosciences). Briefly, 1.2 x 10^5^ MSCs (passage 2) were first cultured attached to the lower side of the membrane, and when a confluency of ≅ 85% was reached, 1 x 10^6^ MM.1S cells were seeded on the upper side (see Supp Methods, co-culture system). After a 24 hour co-culture in RPMI 1640 medium supplemented with 10% FBS and antibiotics, d/pMSCs were recovered by trypsinization and processed for total RNA extraction (Qiagen).

### RNA isolation, cDNA synthesis and microarray hybridization

After assessment of RNA integrity (Agilent 2100 Bioanalyzer, Agilent), biotinylated complementary RNA was synthesized (Enzo) and hybridized to HG-U133 Plus 2.0 GeneChip oligonucleotide arrays (Affymetrix). Quantitation of fluorescence intensities of *probesets* was done using the GeneArray Scanner (Hewlett Packard). Microarray data have been deposited at GEO database (ref GSE46053).

### Microarray data analysis: normalization, signal calculation, differential gene expression and clustering

The RMA (robust multi-array average) algorithm was applied for background correction, intra- and inter-microarray normalization and expression signal calculation. The SAM (significance analysis of microarrays) algorithm was used to identify gene *probesets* displaying significant differential expression when comparing d/pMSCs after co-culture with MSCs from the same origin in mono-culture, using *p*-value cut-offs determined by the FDR method. The corresponding matrix of expression values was analyzed with the hierarchical cluster analysis using algorithm *hclust*. All these methods were applied using R (*http://www.r-project.org*) and Bioconductor (*http://www.bioconductor.org*).

### Gene functional enrichment analysis

Functional enrichment analysis was performed on selected sets of genes after differential expression analyses using two bioinformatics tools: DAVID Bioinformatics Resources 6.7 (*http://david.abcc.ncifcrf.gov/*) [82] and GeneTerm Linker (*http://gtlinker.dacya.ucm.es/*) [83]. The gene sets analyzed for functional enrichment were the most significant from List I (upregulated genes with FDR<0.03) and List II (upregulated genes with FDR<0.02). The downregulated significant genes were also analyzed in separated runs. For construction and analysis of gene functional networks we used the GeneMANIA resource (*http://www.genemania.org/*) and Cytoscape (*http://www.cytoscape.org/*).

### Transcription factor profile analysis

Prediction of transcriptional regulators for the differentially expressed genes was obtained using TransFind and oPOSSUM tools (*http://transfind.sys-bio.net/; http://burgundy.cmmt.ubc.ca/oPOSSUM/*), which look for enrichment in consensus transcription factor binding sites (TFBS) in promoters of specific gene sets using JASPAR and TRANSFAC databases.

### Real-time PCR analysis

TaqMan Gene Expression Assays (Applied Biosystems, Foster City, CA, USA) were used according to manufacturer's instructions. Assay IDs were: *CXCL1*, Hs00236937_m1; *CXCL5*, Hs00171085_m1; *CXCL6*, Hs00237017_m1; *NRG3*, Hs01377907_m1; *NDP*, Hs00181129_m1; *WNT5A*, Hs00998537_m1; *IL8*, Hs00174103_m1; *MMP-12*, Hs 00899662_m1; *ASPM*, Hs 00411505_m1; *HMMR*, Hs00234864_m1. Experiments were performed as previously described [81].

### Proliferation assay

MM.1S-luc cells were seeded in 96-well plates (1 x 10^4^ cells/well) in RPMI 1640 medium with 0.1% FBS and specified concentrations of each recombinant protein. After 72 hours, firefly luciferine was added and bioluminescence measured in a Xenogen IVIS 50 Bioluminiscent System (Caliper Life Sciences).

### Migration assay

We used a 24-well plate with transwells having 5.0 μm pore size membranes (Corning). Serial dilutions of recombinant proteins in α-MEM with 0.5% FBS were placed in the lower chamber, whereas macrophages (2 x 10^5^ cells/100 μl) were added to the upper chamber. After 3 hours, cells that had migrated and adhered to the bottom of the lower chamber were stained with haematoxylin and counted with Trypan Blue solution.

### Tube formation assay

BMEC-1 cells (5 x 10^3^ cells/well) were seeded onto Matrigel-coated 96-well plates in MCDB 131 medium supplemented with 0.1% FBS and indicated doses of the recombinant proteins. After 5 hours, tubule-like structures were counted using a 1 mm^2^ grid, and photographed in an Axiovert 135 (Zeiss) microscope.

### Adhesion assay

Both d/pMSCs (1 x 10^4^ cells/well) were incubated in 96-well plates for 24 hours and then MM.1S-luc cells (1 x 10^5^ cells/well) were added to the monolayer in serum-free medium with specified doses of recombinant proteins. After 3.5 hour incubation, unbound MM.1S-luc cells were removed with three gentle PBS washes and adhered cells quantified by bioluminescence.

### Mineralization and OC formation assays

To evaluate the effect of the recombinant proteins on OB/OC differentiation and function, matrix mineralization and osteoclastogenesis were quantitatively evaluated as previously described [81].

### Statistical analyses

Each condition was analyzed in triplicate or quadruplicate and data were presented as the mean ± SD or SEM (as specified) of at least 3 independent experiments. Statistical comparisons were performed using the Mann-Whitney U test and considered significant for *p*<0.05 (SPSS Statistics 15.0).

## SUPPLEMENTARY MATERIAL, FIGURE AND TABLES


