# Supplementary material for: Transcriptomic profile induced in bone marrow mesenchymal stromal cells after interaction with multiple myeloma cells: implications in myeloma progression and myeloma bone disease
Source: Oncotarget. 2014 Jun 4;5(18):8284–305. doi: 10.18632/oncotarget.2058 (PMC4226683; doi:10.18632/oncotarget.2058)
Supplement: Supplementary file 1 [file oncotarget-05-8284-s001.pdf]

Transcriptomic profile induced in bone marrow mesenchymal stromal cells after interaction with multiple myeloma cells: implications in myeloma progression and myeloma bone disease

Supplementary Methods

Co-culture system

Myeloma plasma cells strongly adhere to bone marrow MSCs, and recovery of separate pure populations after co-culture was possible using a special transwell system with 1 µm diameter pore membrane, which besides molecular interactions, also allows at least partial cellular contact between cells [84]. Specifically, MSCs were cultured attached to the lower side of the insert transwell membrane, whereas myeloma MM.1S cells were cultured in the upper chamber of inserts (BD Biosciences, Paisley, UK). To achieve this, transwell inserts were flipped upside down and placed on a sterile dish; approximately 1.2 x 10^5 MSCs at passage 2 in 450 µl of culture medium were distributed over the surface of the membrane of each insert and incubated for 1 hour at 37ºC to allow cells to adhere. After that time, inserts were flipped back in a 6-well plate and allowed to grow in standard MSC medium. When MSCs reached around 85% confluence, medium was replaced by RPMI 1640 supplemented with 10% heat-inactivated FBS and antibiotics, and 1 x 10^6 MM.1S were added to the upper side of each transwell insert for 24 hours. At the end of the co-culture period, both populations were separated using 0.25% Trypsin-EDTA. MSCs were washed with PBS and collected in RLT buffer (Qiagen), which was then kept at -80ºC for subsequent RNA extraction. For each subject´s MSC sample, six transwells were used for the co-culture condition, and additional four to six wells with MSCs without MM.1S cells were used as a control for the mono-culture condition.

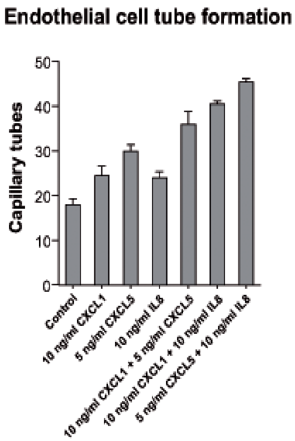

Supplementary Figure 1: Combined activity of CXCL1, CXCL5 and IL8 chemokines in the endothelial cell tube formation assay. Concentrations of CXCL1 and CXCL5 chemokines with submaximal effect were co-administered with that of IL8 to test their combined angiogenic activity. BMEC-1 cells seeded on a Matrigel surface in medium containing 0.1% FBS were incubated with the specified chemokine concentrations, and tubule-like structures were counted under the microscope with the aid of a grid after 5 hours.

Supplementary Table 1, Garcia-Gomez et al.

	Healthy donors n = 8	***Multiple myeloma n = 14***
Age (year ± SD)	68 ± 17	72 ± 8
Sex (% male)	87.5	71.4
Disease stage<br>*ISS classification*		I = 3<br>II = 6<br>III = 5
Bone lesions		Absence = 5<br>Presence = 9

ProbesetID	Gene Symbol	Gene Description	Adjusted p-value (	FCd	Adjusted p-value (	FCp	Average FC
216598_s_at	**CCL2**	chemokine (C-C motif) ligand 2	0,0001069	9,106	0,0214687	6,503	7,805
202331_at	**BCKDHA**	branched chain keto acid dehydrogenase E1, alpha polypeptide (maple syrup urine disease)	0,0003174	1,264	0,0354382	1,261	1,262
220173_at	**C14orf45**	chromosome 14 open reading frame 45	0,0009209	1,922	0,0151647	1,754	1,838
1559780_at	**DDAH1**	dimethylarginine dimethylaminohydrolase 1	0,0017230	1,971	0,0330502	3,341	2,656
213425_at	**WNT5A**	wingless-type MMTV integration site family, member 5A	0,0019462	2,836	0,0293233	2,360	2,598
219861_at	**DNAJC17**	DnaJ (Hsp40) homolog, subfamily C, member 17	0,0020335	1,475	0,0452763	1,216	1,346
209312_x_at	**HLA-DRB1**	major histocompatibility complex, class II, DR beta 1	0,0021284	5,067	0,0253269	7,127	6,097
236028_at	**IBSP**	integrin-binding sialoprotein (bone sialoprotein, bone sialoprotein II)	0,0022103	11,331	0,0125661	7,613	9,472
202862_at	**FAH**	fumarylacetoacetate hydrolase (fumarylacetoacetase)	0,0025083	1,377	0,0264770	1,462	1,420
206336_at	**CXCL6**	chemokine (C-X-C motif) ligand 6 (granulocyte chemotactic protein 2)	0,0026466	8,129	0,0421464	49,334	28,732
201721_s_at	**LAPTM5**	lysosomal associated multispanning membrane protein 5	0,0026466	11,237	0,0277977	7,591	9,414
209774_x_at	**CXCL2**	chemokine (C-X-C motif) ligand 2	0,0029103	7,844	0,0068729	11,755	9,799
204858_s_at	**ECGF1**	endothelial cell growth factor 1 (platelet-derived)	0,0029103	1,822	0,0391911	1,825	1,824
208894_at	**HLA-DRA**	major histocompatibility complex, class II, DR alpha	0,0036029	5,628	0,0231238	11,223	8,426
211990_at	**HLA-DPA1**	major histocompatibility complex, class II, DP alpha 1	0,0036477	3,694	0,0373700	5,102	4,398
206385_s_at	**ANK3**	ankyrin 3, node of Ranvier (ankyrin G)	0,0043882	2,150	0,0361412	2,835	2,493
230424_at	**C5orf13**	chromosome 5 open reading frame 13	0,0044881	2,436	0,0358009	3,084	2,760
210944_s_at	**CAPN3**	calpain 3, (p94)	0,0047146	1,517	0,0114695	1,279	1,398
201438_at	**COL6A3**	collagen, type VI, alpha 3	0,0051017	1,296	0,0219563	1,354	1,325
227560_at	**SFXN2**	sideroflexin 2	0,0051713	1,384	0,0467125	1,550	1,467
202393_s_at	**KLF10**	Kruppel-like factor 10	0,0064782	1,432	0,0006372	1,592	1,512
223786_at	**CHST6**	carbohydrate (N-acetylglucosamine 6-O) sulfotransferase 6	0,0065428	1,834	0,0407390	1,409	1,622
231777_at	**LY6G5B**	lymphocyte antigen 6 complex, locus G5B	0,0075724	1,522	0,0413222	1,271	1,397
218777_at	**C8orf20**	chromosome 8 open reading frame 20	0,0076819	1,562	0,0240891	1,503	1,532
202888_s_at	**ANPEP**	alanyl (membrane) aminopeptidase (aminopeptidase N, aminopeptidase M, microsomal aminopeptidase, CD13, p150)	0,0077999	2,527	0,0277908	2,805	2,666
201422_at	**IFI30**	interferon, gamma-inducible protein 30	0,0079461	2,737	0,0414287	2,231	2,484
232797_at	**ITGAV**	integrin, alpha V (vitronectin receptor, alpha polypeptide, antigen CD51)	0,0082231	2,223	0,0343200	2,494	2,359
226670_s_at	**C20orf119**	chromosome 20 open reading frame 119	0,0089277	1,548	0,0400370	1,480	1,514
204345_at	**COL16A1**	collagen, type XVI, alpha 1	0,0090280	1,703	0,0341366	1,754	1,728
204859_s_at	**APAF1**	apoptotic peptidase activating factor	0,0090360	1,808	0,0162097	1,867	1,838
205453_at	**HOXB2**	homeo box B2	0,0093026	1,369	0,0302891	1,271	1,320
201288_at	**ARHGDIB**	Rho GDP dissociation inhibitor (GDI) beta	0,0095299	2,914	0,0372581	2,875	2,894
204319_s_at	**RGS10**	regulator of G-protein signalling 10	0,0095581	1,470	0,0123074	1,210	1,340
49878_at	**PEX16**	peroxisomal biogenesis factor 16	0,0096396	1,173	0,0148603	1,353	1,263
1552502_s_at	**RHBDL2**	rhomboid, veinlet-like 2 (Drosophila)	0,0101613	1,806	0,0233693	2,183	1,995
202345_s_at	**FABP5**	fatty acid binding protein 5 (psoriasis-associated)	0,0102262	2,332	0,0295247	2,430	2,381
236599_at	**SERPINE2**	serpin peptidase inhibitor, clade E (nexin, plasminogen activator inhibitor type 1), member 2	0,0102418	2,978	0,0187907	3,330	3,154
242812_at	**HCG18**	HLA complex group 18	0,0108107	1,263	0,0152526	1,227	1,245
213068_at	**DPT**	dermatopontin	0,0108431	3,851	0,0112443	2,558	3,205
244356_at	**PTPN12**	protein tyrosine phosphatase, non-receptor type 12	0,0114542	1,277	0,0172274	1,837	1,557
227314_at	**ITGA2**	integrin, alpha 2 (CD49B, alpha 2 subunit of VLA-2 receptor)	0,0114774	3,851	0,0148603	6,714	5,283
202901_x_at	**CTSS**	cathepsin S	0,0115022	5,684	0,0362839	5,598	5,641
225681_at	**CTHRC1**	collagen triple helix repeat containing 1	0,0116078	1,618	0,0380027	2,105	1,861
224909_s_at	**PREX1**	NA	0,0116136	1,901	0,0214277	1,857	1,879
225883_at	**ATG16L2**	ATG16 autophagy related 16-like 2 (S. cerevisiae)	0,0118108	1,386	0,0043682	1,188	1,287
228105_at	**C11orf23**	chromosome 11 open reading frame 23	0,0118453	1,624	0,0130023	1,427	1,526
229055_at	**GPR68**	G protein-coupled receptor 68	0,0119591	2,449	0,0171176	4,001	3,225
1557578_at	**PHLDB2**	pleckstrin homology-like domain, family B, member 2	0,0120559	1,163	0,0463699	1,917	1,540
207719_x_at	**KIAA0470**	KIAA0470	0,0123352	1,381	0,0001600	1,540	1,461
202859_x_at	**IL8**	interleukin 8	0,0124165	64,480	0,0222056	74,959	69,719
209396_s_at	**CHI3L1**	chitinase 3-like 1 (cartilage glycoprotein-39)	0,0125059	6,977	0,0202159	3,383	5,180
212192_at	**KCTD12**	potassium channel tetramerisation domain containing 12	0,0129008	1,433	0,0461273	1,950	1,692

232633_at	**XRCC5**	c("X-ray repair complementing defective repair in Chinese hamster cells 5 (double-strand-break rejoining", " Ku autoantigen, 80kDa)")	0,0129458	1,233	0,0160292	1,140	1,187
228597_at	**C21orf45**	chromosome 21 open reading frame 45	0,0131524	1,886	0,0005687	2,220	2,053
219118_at	**FKBP11**	FK506 binding protein 11, 19 kDa	0,0132921	1,801	0,0301493	1,969	1,885
241473_at	**SULF1**	sulfatase 1	0,0133093	1,659	0,0408668	1,941	1,800
238688_at	**TPM1**	tropomyosin 1 (alpha)	0,0133383	1,843	0,0378446	1,157	1,500
232098_at	**DST**	dystonin	0,0133592	1,571	0,0057709	1,798	1,684
236249_at	**IKIP**	NA	0,0133592	1,686	0,0295170	2,077	1,881
215209_at	**SEC24D**	SEC24 related gene family, member D (S. cerevisiae)	0,0133592	1,734	0,0017270	1,633	1,684
202854_at	**HPRT1**	hypoxanthine phosphoribosyltransferase 1 (Lesch-Nyhan syndrome)	0,0133827	1,359	0,0427870	1,413	1,386
218443_s_at	**DAZAP1**	DAZ associated protein 1	0,0135334	1,425	0,0448179	1,418	1,421
220994_s_at	**STXBP6**	syntaxin binding protein 6 (amisyn)	0,0135746	2,378	0,0390259	2,407	2,393
215081_at	**KIAA1024**	NA	0,0136373	1,446	0,0111535	1,399	1,423
244777_at	**DCP2**	DCP2 decapping enzyme homolog (S. cerevisiae)	0,0137502	1,325	0,0164350	1,336	1,331
1557432_at	**RASAL2**	RAS protein activator like 2	0,0138995	1,661	0,0183939	1,371	1,516
235419_at	**ERRFI1**	ERBB receptor feedback inhibitor 1	0,0146227	1,768	0,0275438	1,505	1,637
228271_at	**SND1**	NA	0,0146555	1,415	0,0175803	1,196	1,306
211048_s_at	**PDIA4**	protein disulfide isomerase family A, member 4	0,0146905	1,832	0,0275191	1,346	1,589
210845_s_at	**PLAUR**	plasminogen activator, urokinase receptor	0,0146905	2,758	0,0384651	2,405	2,582
204614_at	**SERPINB2**	serpin peptidase inhibitor, clade B (ovalbumin), member 2	0,0146905	21,921	0,0200268	22,947	22,434
204670_x_at	**HLA-DRB5**	major histocompatibility complex, class II, DR beta 5	0,0147289	4,057	0,0439617	4,181	4,119
209875_s_at	**SPP1**	secreted phosphoprotein 1 (osteopontin, bone sialoprotein I, early T-lymphocyte activation 1)	0,0147289	14,388	0,0232869	11,143	12,765
227236_at	**TSPAN2**	tetraspanin 2	0,0147300	3,695	0,0204257	7,872	5,784
204748_at	**PTGS2**	prostaglandin-endoperoxide synthase 2 (prostaglandin G/H synthase and cyclooxygenase)	0,0147884	5,908	0,0038632	5,308	5,608
202508_s_at	**SNAP25**	synaptosomal-associated protein, 25kDa	0,0148678	1,594	0,0076786	1,228	1,411
1569477_at	**FOXO3A**	forkhead box O3A	0,0149341	1,397	0,0119939	1,267	1,332
237624_at	**COL4A2**	collagen, type IV, alpha 2	0,0150065	1,585	0,0044944	1,566	1,575
228937_at	**FLJ38725**	NA	0,0150065	2,555	0,0270675	4,007	3,281
235629_at	**FN1**	fibronectin 1	0,0150065	3,342	0,0382123	3,214	3,278
230636_s_at	**KLF9**	Kruppel-like factor 9	0,0150065	1,784	0,0133183	1,739	1,762
204575_s_at	**MMP19**	matrix metallopeptidase 19	0,0150065	1,627	0,0214277	1,419	1,523
221898_at	**PDPN**	podoplanin	0,0150065	3,781	0,0069289	3,549	3,665
225947_at	**MYOHD1**	myosin head domain containing 1	0,0150760	1,391	0,0354872	1,313	1,352
1558948_a_at	**OIP5**	Opa interacting protein 5	0,0150760	1,144	0,0340173	3,587	2,366
219920_s_at	**GMPPB**	GDP-mannose pyrophosphorylase B	0,0151438	1,406	0,0235591	1,478	1,442
202211_at	**ARFGAP3**	ADP-ribosylation factor GTPase activating protein 3	0,0152088	1,322	0,0004401	1,359	1,340
232174_at	**EXT1**	exostoses (multiple) 1	0,0152270	3,072	0,0047405	2,181	2,626
205266_at	**LIF**	leukemia inhibitory factor (cholinergic differentiation factor)	0,0152864	3,994	0,0269940	3,657	3,825
1553207_at	**ARL10A**	ADP-ribosylation factor-like 10A	0,0153307	1,239	0,0118424	1,213	1,226
203739_at	**ZNF217**	zinc finger protein 217	0,0153920	1,713	0,0340441	1,877	1,795
230748_at	**SLC16A6**	solute carrier family 16 (monocarboxylic acid transporters), member 6	0,0155854	6,665	0,0050498	3,157	4,911
213603_s_at	**RAC2**	ras-related C3 botulinum toxin substrate 2 (rho family, small GTP binding protein Rac2)	0,0155898	2,133	0,0248731	2,217	2,175
234465_at	**EME1**	essential meiotic endonuclease 1 homolog 1 (S. pombe)	0,0156589	1,240	0,0031122	2,301	1,771
215223_s_at	**SOD2**	superoxide dismutase 2, mitochondrial	0,0157061	3,522	0,0452766	5,721	4,622
205174_s_at	**QPCT**	glutaminyl-peptide cyclotransferase (glutaminyl cyclase)	0,0157434	1,829	0,0352772	2,091	1,960
1554899_s_at	**FCER1G**	c("Fc fragment of IgE, high affinity I, receptor for", " gamma polypeptide")	0,0157912	8,936	0,0450666	14,702	11,819
229354_at	**AHRR**	aryl-hydrocarbon receptor repressor	0,0158242	2,866	0,0254197	3,158	3,012
204136_at	**COL7A1**	collagen, type VII, alpha 1 (epidermolysis bullosa, dystrophic, dominant and recessive)	0,0158242	2,249	0,0200268	2,390	2,319
202855_s_at	**SLC16A3**	solute carrier family 16 (monocarboxylic acid transporters), member 3	0,0159243	2,790	0,0232869	7,980	5,385
215269_at	**TMEM1**	transmembrane protein 1	0,0161353	1,529	0,0370224	1,827	1,678
219634_at	**CHST11**	carbohydrate (chondroitin 4) sulfotransferase 11	0,0161958	1,813	0,0284760	2,383	2,098
203889_at	**SGNE1**	secretory granule, neuroendocrine protein 1 (7B2 protein)	0,0162196	4,066	0,0200268	8,230	6,148
222584_at	**MSTO1**	misato homolog 1 (Drosophila)	0,0163508	1,387	0,0267056	1,506	1,447
204470_at	**CXCL1**	chemokine (C-X-C motif) ligand 1 (melanoma growth stimulating activity, alpha)	0,0164075	15,749	0,0261493	38,361	27,055

217362_x_at	**HLA-DRB6**	major histocompatibility complex, class II, DR beta 6 (pseudogene)	0,0164611	2,274	0,0390073	3,662	2,968
230142_s_at	**CIRBP**	cold inducible RNA binding protein	0,0164741	1,497	0,0341417	1,470	1,483
220518_at	**ABI3BP**	ABI gene family, member 3 (NESH) binding protein	0,0165156	1,775	0,0464354	1,213	1,494
36711_at	**MAFF**	v-maf musculoaponeurotic fibrosarcoma oncogene homolog F (avian)	0,0165866	2,702	0,0058141	2,346	2,524
213359_at	**HNRPD**	heterogeneous nuclear ribonucleoprotein D (AU-rich element RNA binding protein 1, 37kDa)	0,0168060	1,967	0,0317752	2,007	1,987
46665_at	**SEMA4C**	sema domain, immunoglobulin domain (Ig), transmembrane domain (TM) and short cytoplasmic domain, (semaphorin) 4C	0,0168791	2,126	0,0174497	2,623	2,374
227410_at	**FAM43A**	family with sequence similarity 43, member A	0,0168916	1,952	0,0032003	2,556	2,254
222040_at	**HNRPA1**	heterogeneous nuclear ribonucleoprotein A1	0,0168916	2,488	0,0427028	2,086	2,287
203936_s_at	**MMP9**	matrix metallopeptidase 9 (gelatinase B, 92kDa gelatinase, 92kDa type IV collagenase)	0,0168916	19,412	0,0285061	14,548	16,980
226287_at	**NY-REN-41**	NA	0,0168916	1,889	0,0371674	1,841	1,865
214974_x_at	**CXCL5**	chemokine (C-X-C motif) ligand 5	0,0169643	82,878	0,0230671	183,967	133,422
212809_at	**NFATC2IP**	nuclear factor of activated T-cells, cytoplasmic, calcineurin-dependent 2 interacting protein	0,0169987	1,317	0,0259083	1,214	1,266
216450_x_at	**TRA1**	tumor rejection antigen (gp96) 1	0,0170431	1,412	0,0327265	1,907	1,659
234276_at	**USP6NL**	USP6 N-terminal like	0,0170431	1,131	0,0027915	1,460	1,295
241387_at	**PTK2**	PTK2 protein tyrosine kinase 2	0,0170508	1,266	0,0124139	1,292	1,279
204662_at	**CP110**	NA	0,0171490	1,401	0,0322053	1,280	1,341
1556006_s_at	**CSNK1A1**	casein kinase 1, alpha 1	0,0171569	1,593	0,0309596	1,541	1,567
242542_at	**PHACTR2**	phosphatase and actin regulator 2	0,0171569	1,262	0,0384583	1,116	1,189
218644_at	**PLEK2**	pleckstrin 2	0,0171569	1,541	0,0260543	1,414	1,478
205499_at	**SRPX2**	sushi-repeat-containing protein, X-linked 2	0,0171569	3,474	0,0043914	2,841	3,157
236462_at	**YEATS2**	YEATS domain containing 2	0,0171569	2,362	0,0141412	1,739	2,050
223203_at	**TMEM29**	transmembrane protein 29	0,0171630	1,491	0,0214687	1,461	1,476
213160_at	**DOCK2**	dedicator of cytokinesis 2	0,0172214	1,899	0,0374870	1,565	1,732
1556126_s_at	**GPATC2**	G patch domain containing 2	0,0172214	1,382	0,0327992	1,204	1,293
212665_at	**TIPARP**	TCDD-inducible poly(ADP-ribose) polymerase	0,0172761	2,590	0,0218822	3,547	3,069
233445_at	**BUB1**	BUB1 budding uninhibited by benzimidazoles 1 homolog (yeast)	0,0172846	1,197	0,0333673	7,094	4,146
207850_at	**CXCL3**	chemokine (C-X-C motif) ligand 3	0,0173821	12,328	0,0425036	24,194	18,261
212110_at	**SLC39A14**	solute carrier family 39 (zinc transporter), member 14	0,0173843	1,425	0,0234726	3,494	2,460
205959_at	**MMP13**	matrix metallopeptidase 13 (collagenase 3)	0,0174243	4,402	0,0269888	2,316	3,359
204440_at	**CD83**	CD83 antigen (activated B lymphocytes, immunoglobulin superfamily)	0,0175131	1,493	0,0042996	1,452	1,473
201430_s_at	**DPYSL3**	dihydropyrimidinase-like 3	0,0175318	1,943	0,0219563	2,691	2,317
241775_at	**SCFD1**	sec1 family domain containing 1	0,0176093	1,735	0,0255358	1,523	1,629
210385_s_at	**ARTS-1**	NA	0,0176321	1,523	0,0372135	1,559	1,541
201762_s_at	**PSME2**	proteasome (prosome, macropain) activator subunit 2 (PA28 beta)	0,0177701	1,723	0,0207825	1,931	1,827
36554_at	**ASMTL**	acetylserotonin O-methyltransferase-like	0,0177932	1,403	0,0227682	1,586	1,494
212020_s_at	**MKI67**	antigen identified by monoclonal antibody Ki-67	0,0177969	2,135	0,0354872	4,831	3,483
1555441_at	**FLJ10808**	NA	0,0178338	1,251	0,0255380	1,607	1,429
209284_s_at	**RAP140**	NA	0,0178528	1,285	0,0040071	1,323	1,304
225569_at	**EIF2C2**	eukaryotic translation initiation factor 2C, 2	0,0179127	1,353	0,0016655	1,468	1,410
204745_x_at	**MT1G**	metallothionein 1G	0,0179417	1,876	0,0167915	2,430	2,153
237411_at	**ADAMTS6**	ADAM metallopeptidase with thrombospondin type 1 motif, 6	0,0180081	3,248	0,0204257	3,522	3,385
219232_s_at	**EGLN3**	egl nine homolog 3 (C. elegans)	0,0180397	1,368	0,0023942	1,397	1,382
226569_s_at	**CHTF18**	CTF18, chromosome transmission fidelity factor 18 homolog (S. cerevisiae)	0,0180432	1,353	0,0416371	1,263	1,308
227517_s_at	**GAS5**	growth arrest-specific 5	0,0180486	1,641	0,0436930	2,229	1,935
230152_at	**WDR52**	WD repeat domain 52	0,0180877	1,293	0,0241750	1,340	1,317
236764_at	**POLR2B**	polymerase (RNA) II (DNA directed) polypeptide B, 140kDa	0,0180974	1,837	0,0113969	1,556	1,697
232472_at	**FNDC3B**	fibronectin type III domain containing 3B	0,0181516	1,503	0,0299505	2,045	1,774
219559_at	**C20orf59**	chromosome 20 open reading frame 59	0,0182821	1,912	0,0239741	1,770	1,841
226651_at	**HOMER1**	homer homolog 1 (Drosophila)	0,0182969	1,594	0,0113898	1,597	1,596
204521_at	**HSU79274**	NA	0,0183145	1,396	0,0050918	1,626	1,511
200754_x_at	**SFRS2**	splicing factor, arginine/serine-rich 2	0,0183179	1,408	0,0148603	1,971	1,690
201631_s_at	**IER3**	immediate early response 3	0,0183842	3,051	0,0230733	2,644	2,847
206472_s_at	**TLE3**	transducin-like enhancer of split 3 (E(sp1) homolog, Drosophila)	0,0183842	1,913	0,0231570	1,653	1,783
204400_at	**EFS**	embryonal Fyn-associated substrate	0,0185499	1,643	0,0427028	1,724	1,683
205844_at	**VNN1**	vanin 1	0,0186312	1,378	0,0267359	2,742	2,060
201489_at	**PPIF**	peptidylprolyl isomerase F (cyclophilin F)	0,0186694	1,317	0,0435003	1,747	1,532
219179_at	**DACT1**	dapper, antagonist of beta-catenin, homolog 1 (Xenopus laevis)	0,0186777	2,131	0,0365116	2,427	2,279
1555832_s_at	**KLF6**	Kruppel-like factor 6	0,0186777	1,604	0,0254560	1,358	1,481

224842_at	**SMG1**	NA	0,0186777	1,398	0,0009703	1,488	1,443
218673_s_at	**ATG7**	ATG7 autophagy related 7 homolog (S. cerevisiae)	0,0187398	1,190	0,0218822	1,230	1,210
217767_at	**C3**	complement component 3	0,0187632	3,268	0,0408668	3,939	3,604
39891_at	**DKFZp547K1113**	NA	0,0187632	1,186	0,0092280	1,135	1,161
225633_at	**DPY19L3**	dpy-19-like 3 (C. elegans)	0,0188067	1,297	0,0219392	1,254	1,276
204382_at	**NAT9**	N-acetyltransferase 9	0,0188971	1,218	0,0408574	1,219	1,219
213909_at	**LRRC15**	leucine rich repeat containing 15	0,0189241	3,070	0,0234030	6,295	4,683
215813_s_at	**PTGS1**	prostaglandin-endoperoxide synthase 1 (prostaglandin G/H synthase and cyclooxygenase)	0,0190822	2,186	0,0114590	2,832	2,509
232080_at	**HECW2**	HECT, C2 and WW domain containing E3 ubiquitin protein ligase 2	0,0190926	1,677	0,0160119	1,625	1,651
226722_at	**FAM20C**	family with sequence similarity 20, member C	0,0191181	1,529	0,0234158	1,423	1,476
215418_at	**PARVA**	parvin, alpha	0,0191181	1,640	0,0084115	1,389	1,514
238823_at	**FMNL3**	formin-like 3	0,0191887	1,964	0,0453032	2,038	2,001
206461_x_at	**MT1H**	metallothionein 1H	0,0191944	1,818	0,0235292	2,310	2,064
206614_at	**GDF5**	growth differentiation factor 5 (cartilage-derived morphogenetic protein-1)	0,0192810	2,185	0,0055894	2,334	2,260
227033_at	**ELL3**	elongation factor RNA polymerase II-like 3	0,0192918	1,258	0,0415978	1,295	1,277
211724_x_at	**FLJ20323**	NA	0,0192986	1,311	0,0130736	1,648	1,479
242725_at	**KDELR2**	KDEL (Lys-Asp-Glu-Leu) endoplasmic reticulum protein retention receptor 2	0,0193426	1,787	0,0350495	1,479	1,633
1552489_s_at	**MPP4**	membrane protein, palmitoylated 4 (MAGUK p55 subfamily member 4)	0,0193599	1,142	0,0148135	1,342	1,242
224629_at	**LMAN1**	lectin, mannose-binding, 1	0,0193676	1,290	0,0286355	2,400	1,845
226873_at	**FAM63B**	family with sequence similarity 63, member B	0,0194174	1,619	0,0325065	1,267	1,443
201014_s_at	**PAICS**	phosphoribosylaminoimidazole carboxylase, phosphoribosylaminoimidazole succinocarboxamide synthetase	0,0194565	1,447	0,0200268	2,358	1,903
213098_at	**RQCD1**	RCD1 required for cell differentiation1 homolog (S. pombe)	0,0195975	1,228	0,0218822	1,518	1,373
243184_at	**TJP1**	tight junction protein 1 (zona occludens 1)	0,0196260	1,258	0,0016499	1,361	1,309
242669_at	**UFM1**	ubiquitin-fold modifier 1	0,0196652	1,298	0,0194280	1,737	1,517
243012_at	**RAPH1**	Ras association (RalGDS/AF-6) and pleckstrin homology domains 1	0,0196938	1,464	0,0210759	1,411	1,437
223278_at	**GJB2**	gap junction protein, beta 2, 26kDa (connexin 26)	0,0198802	2,251	0,0165161	2,477	2,364
202437_s_at	**CYP1B1**	cytochrome P450, family 1, subfamily B, polypeptide 1	0,0199758	1,293	0,0444075	3,594	2,443
229095_s_at	**LIMS3**	LIM and senescent cell antigen-like domains 3	0,0199905	2,993	0,0008719	2,089	2,541
233109_at	**COL12A1**	collagen, type XII, alpha 1	0,0200854	1,972	0,0380027	2,761	2,367
206173_x_at	**GABPB2**	GA binding protein transcription factor, beta subunit 2	0,0200854	1,231	0,0365949	1,575	1,403
204103_at	**CCL4**	chemokine (C-C motif) ligand 4	0,0201770	4,691	0,0446544	8,621	6,656
37408_at	**MRC2**	mannose receptor, C type 2	0,0202558	1,414	0,0276269	1,243	1,329
232431_at	**NR3C1**	nuclear receptor subfamily 3, group C, member 1 (glucocorticoid receptor)	0,0203328	2,715	0,0016095	2,338	2,527
205207_at	**IL6**	interleukin 6 (interferon, beta 2)	0,0203563	4,210	0,0412945	4,078	4,144
216005_at	**TNC**	tenascin C (hexabrachion)	0,0204065	2,842	0,0204257	2,717	2,779
208619_at	**DDB1**	damage-specific DNA binding protein 1, 127kDa	0,0204174	1,184	0,0397478	1,267	1,225
235705_at	**TRIO**	triple functional domain (PTPRF interacting)	0,0205789	2,278	0,0414614	1,932	2,105
224753_at	**CDCA5**	cell division cycle associated 5	0,0206104	2,117	0,0291992	3,176	2,646
1565703_at	**SMAD4**	SMAD, mothers against DPP homolog 4 (Drosophila)	0,0206331	1,300	0,0382123	1,356	1,328
1555878_at	**RPS24**	ribosomal protein S24	0,0206496	1,789	0,0371744	1,711	1,750
209827_s_at	**IL16**	interleukin 16 (lymphocyte chemoattractant factor)	0,0207512	1,570	0,0093677	1,381	1,476
217523_at	**CD44**	CD44 antigen (homing function and Indian blood group system)	0,0207606	1,817	0,0267703	2,576	2,197
203044_at	**CHSY1**	carbohydrate (chondroitin) synthase 1	0,0207606	1,488	0,0253930	1,443	1,466
208892_s_at	**DUSP6**	dual specificity phosphatase 6	0,0207606	2,656	0,0053722	2,498	2,577
223276_at	**NID67**	NA	0,0207606	2,306	0,0056156	2,292	2,299
215611_at	**TCF12**	transcription factor 12 (HTF4, helix-loop-helix transcription factors 4)	0,0207606	1,168	0,0344502	1,062	1,115
202729_s_at	**LTBP1**	latent transforming growth factor beta binding protein 1	0,0207743	1,761	0,0123930	1,617	1,689
212723_at	**PTDSR**	phosphatidylserine receptor	0,0207743	1,714	0,0232869	1,642	1,678
204422_s_at	**FGF2**	fibroblast growth factor 2 (basic)	0,0207959	2,221	0,0347741	1,898	2,060
223822_at	**SUSD4**	sushi domain containing 4	0,0208123	1,184	0,0129125	1,281	1,232
210659_at	**CMKLR1**	chemokine-like receptor 1	0,0208304	1,820	0,0124777	1,765	1,792
219888_at	**SPAG4**	sperm associated antigen 4	0,0208304	2,584	0,0382265	1,745	2,164
225557_at	**AXUD1**	AXIN1 up-regulated 1	0,0208721	2,153	0,0120187	1,712	1,932
242052_at	**BICD1**	bicaudal D homolog 1 (Drosophila)	0,0208721	2,369	0,0011313	2,063	2,216

213629_x_at	**MT1F**	metallothionein 1F (functional)	0,0208721	2,114	0,0247191	3,481	2,797
202657_s_at	**SERTAD2**	SERTA domain containing 2	0,0208721	1,279	0,0313547	1,484	1,381
227415_at	**DGKH**	diacylglycerol kinase, eta	0,0208760	1,623	0,0003594	1,640	1,632
223767_at	**GPR84**	G protein-coupled receptor 84	0,0210369	5,309	0,0327572	7,350	6,330
205100_at	**GFPT2**	glutamine-fructose-6-phosphate transaminase 2	0,0210603	2,028	0,0418043	2,917	2,473
210512_s_at	**VEGF**	vascular endothelial growth factor	0,0210603	2,173	0,0224770	2,419	2,296
212820_at	**DMXL2**	Dmx-like 2	0,0211174	1,712	0,0164359	1,575	1,643
1556012_at	**FLJ38753**	NA	0,0212177	1,220	0,0417664	1,147	1,183
229638_at	**IRX3**	iroquois homeobox protein 3	0,0212425	1,633	0,0005796	1,907	1,770
204224_s_at	**GCH1**	GTP cyclohydrolase 1 (dopa-responsive dystonia)	0,0212457	3,301	0,0409130	5,112	4,206
203320_at	**LNK**	NA	0,0212457	1,422	0,0268001	1,464	1,443
243591_at	**LAMC1**	laminin, gamma 1 (formerly LAMB2)	0,0212466	1,662	0,0299632	1,869	1,765
209305_s_at	**GADD45B**	growth arrest and DNA-damage-inducible, beta	0,0212860	1,903	0,0041158	1,772	1,837
218544_s_at	**RCL1**	RNA terminal phosphate cyclase-like 1	0,0212947	1,216	0,0384583	1,405	1,311
218951_s_at	**PLCXD1**	phosphatidylinositol-specific phospholipase C, X domain containing 1	0,0213075	1,327	0,0236879	1,303	1,315
203062_s_at	**MDC1**	mediator of DNA damage checkpoint 1	0,0213254	1,373	0,0038432	1,346	1,359
205205_at	**RELB**	v-rel reticuloendotheliosis viral oncogene homolog B, nuclear factor of kappa light polypeptide gene enhancer in B-cells 3 (avian)	0,0213254	2,033	0,0295933	1,894	1,964
228933_at	**NHS**	Nance-Horan syndrome (congenital cataracts and dental anomalies)	0,0213726	1,794	0,0033400	1,909	1,852
222557_at	**STMN3**	stathmin-like 3	0,0214111	1,797	0,0287868	1,621	1,709
223343_at	**MS4A7**	membrane-spanning 4-domains, subfamily A, member 7	0,0214170	24,775	0,0318979	26,420	25,598
223484_at	**NMES1**	NA	0,0214360	23,597	0,0355597	21,129	22,363
210109_at	**NAG8**	NA	0,0214539	1,475	0,0073888	1,189	1,332
222537_s_at	**CDC42SE1**	CDC42 small effector 1	0,0214641	1,402	0,0058025	1,350	1,376
204627_s_at	**ITGB3**	integrin, beta 3 (platelet glycoprotein IIIa, antigen CD61)	0,0215692	2,530	0,0236325	3,108	2,819
232511_at	**GCC2**	GRIP and coiled-coil domain containing 2	0,0216183	1,204	0,0250050	1,257	1,231
210962_s_at	**AKAP9**	A kinase (PRKA) anchor protein (yotiao) 9	0,0216255	1,300	0,0214687	1,232	1,266
221489_s_at	**SPRY4**	sprouty homolog 4 (Drosophila)	0,0216391	1,692	0,0306878	1,329	1,511
47105_at	**DUS2L**	dihydrouridine synthase 2-like (SMM1, S. cerevisiae)	0,0218000	1,151	0,0246987	1,097	1,124
221290_s_at	**MUM1**	melanoma associated antigen (mutated) 1	0,0218000	1,169	0,0212047	1,198	1,183
218992_at	**C9orf46**	chromosome 9 open reading frame 46	0,0218648	1,366	0,0415828	1,469	1,418
203471_s_at	**PLEK**	pleckstrin	0,0218648	3,079	0,0275336	2,052	2,565
204775_at	**CHAF1B**	chromatin assembly factor 1, subunit B (p60)	0,0218708	1,275	0,0320467	1,316	1,295
33307_at	**CGI-96**	NA	0,0219417	1,341	0,0303651	1,330	1,336
227700_x_at	**ATAD3A**	ATPase family, AAA domain containing 3A	0,0219572	1,342	0,0374870	1,183	1,262
221802_s_at	**KIAA1598**	KIAA1598	0,0220023	1,624	0,0463940	1,889	1,756
221505_at	**ANP32E**	acidic (leucine-rich) nuclear phosphoprotein 32 family, member E	0,0220139	1,440	0,0023719	2,014	1,727
220976_s_at	**KRTAP1-1**	keratin associated protein 1-1	0,0222074	3,124	0,0349218	3,000	3,062
205088_at	**CXorf6**	chromosome X open reading frame 6	0,0222094	1,991	0,0251213	2,702	2,346
1557347_at	**MCPH1**	microcephaly, primary autosomal recessive 1	0,0223339	1,200	0,0316065	1,209	1,205
208581_x_at	**MT1X**	metallothionein 1X	0,0223743	1,647	0,0200268	2,338	1,993
1555673_at	**KRTAP2-1**	keratin associated protein 2-1	0,0223981	4,570	0,0278475	2,243	3,406
206432_at	**HAS2**	hyaluronan synthase 2	0,0225146	4,300	0,0256104	4,592	4,446
219500_at	**CLCF1**	cardiotrophin-like cytokine factor 1	0,0225824	1,548	0,0367936	1,472	1,510
203904_x_at	**CD82**	CD82 antigen	0,0225891	1,725	0,0417119	1,698	1,711
238243_at	**CCNH**	cyclin H	0,0226018	1,768	0,0112932	1,886	1,827
223188_at	**FLJ12886**	NA	0,0226570	1,182	0,0367058	1,152	1,167
212887_at	**SEC23A**	Sec23 homolog A (S. cerevisiae)	0,0226570	1,287	0,0275336	1,672	1,479
222579_at	**UBE1DC1**	ubiquitin-activating enzyme E1-domain containing 1	0,0226570	1,332	0,0200268	1,588	1,460
237183_at	**GALNT5**	UDP-N-acetyl-alpha-D-galactosamine:polypeptide N-acetylgalactosaminyltransferase 5 (GalNAc-T5)	0,0227111	1,679	0,0451026	2,035	1,857
227176_at	**FLJ40126**	NA	0,0228258	1,410	0,0354872	1,451	1,431
233314_at	**PTEN**	phosphatase and tensin homolog (mutated in multiple advanced cancers 1)	0,0228258	1,285	0,0174468	1,218	1,251
208488_s_at	**CR1**	complement component (3b/4b) receptor 1, including Knops blood group system	0,0228783	1,153	0,0088644	1,156	1,155
204078_at	**SC65**	NA	0,0228783	1,522	0,0337774	1,580	1,551
243286_at	**CUL1**	cullin 1	0,0228834	1,629	0,0322071	1,369	1,499
219134_at	**ELTD1**	EGF, latrophilin and seven transmembrane domain containing 1	0,0229325	1,853	0,0267816	2,915	2,384
218404_at	**SNX10**	sorting nexin 10	0,0229605	3,266	0,0118503	3,295	3,281
209984_at	**JMJD2C**	jumonji domain containing 2C	0,0229782	1,382	0,0270264	1,180	1,281
202620_s_at	**PLOD2**	procollagen-lysine, 2-oxoglutarate 5-dioxygenase 2	0,0229782	1,526	0,0288735	1,488	1,507

205567_at	**CHST1**	carbohydrate (keratan sulfate Gal-6) sulfotransferase 1	0,0230083	2,037	0,0425429	1,522	1,779
221029_s_at	**WNT5B**	wingless-type MMTV integration site family, member 5B	0,0230400	2,234	0,0416258	1,961	2,097
204679_at	**KCNK1**	potassium channel, subfamily K, member 1	0,0230741	1,376	0,0424829	1,750	1,563
213479_at	**NPTX2**	neuronal pentraxin II	0,0232234	5,313	0,0206995	1,633	3,473
201011_at	**RPN1**	ribophorin I	0,0232315	1,376	0,0311971	1,382	1,379
203201_at	**PMM2**	phosphomannomutase 2	0,0232807	1,409	0,0219563	1,353	1,381
219386_s_at	**SLAMF8**	SLAM family member 8	0,0233935	4,018	0,0225927	3,177	3,598
239597_at	**PAN3**	NA	0,0233968	1,775	0,0316787	1,340	1,558
211987_at	**TOP2B**	topoisomerase (DNA) II beta 180kDa	0,0234117	1,176	0,0006372	1,286	1,231
202655_at	**ARMET**	arginine-rich, mutated in early stage tumors	0,0234162	1,277	0,0319996	1,307	1,292
211852_s_at	**ATRN**	attractin	0,0234555	1,353	0,0304079	1,803	1,578
220088_at	**C5R1**	complement component 5 receptor 1 (C5a ligand)	0,0235601	7,447	0,0231412	4,498	5,973
204122_at	**TYROBP**	TYRO protein tyrosine kinase binding protein	0,0236576	5,429	0,0359588	6,050	5,740
239166_at	**MARK3**	MAP/microtubule affinity-regulating kinase 3	0,0236636	1,218	0,0106381	1,245	1,232
233540_s_at	**CDK5RAP2**	CDK5 regulatory subunit associated protein 2	0,0236643	1,701	0,0373700	1,845	1,773
212949_at	**BRRN1**	barren homolog (Drosophila)	0,0236914	1,796	0,0230733	2,703	2,250
202241_at	**TRIB1**	tribbles homolog 1 (Drosophila)	0,0237192	1,421	0,0118424	1,765	1,593
204915_s_at	**SOX11**	SRY (sex determining region Y)-box 11	0,0238045	1,651	0,0261493	4,275	2,963
1569464_at	**PPFIBP1**	PTPRF interacting protein, binding protein 1 (liprin beta 1)	0,0238590	1,272	0,0288199	1,685	1,478
212847_at	**FUBP1**	far upstream element (FUSE) binding protein 1	0,0238982	1,619	0,0148603	1,750	1,685
208025_s_at	**HMGA2**	high mobility group AT-hook 2	0,0238982	2,643	0,0023066	3,268	2,955
232287_at	**PGBD3**	piggyBac transposable element derived 3	0,0239948	2,453	0,0190392	1,537	1,995
205588_s_at	**FGFR1OP**	FGFR1 oncogene partner	0,0240662	1,427	0,0230479	1,357	1,392
215691_x_at	**C1orf41**	chromosome 1 open reading frame 41	0,0241752	1,525	0,0011824	1,690	1,607
228964_at	**PRDM1**	PR domain containing 1, with ZNF domain	0,0241752	2,303	0,0414990	1,271	1,787
204346_s_at	**RASSF1**	Ras association (RalGDS/AF-6) domain family 1	0,0241752	1,665	0,0328483	1,586	1,626
227972_at	**TOR2A**	torsin family 2, member A	0,0242145	1,171	0,0061755	1,219	1,195
203696_s_at	**RFC2**	replication factor C (activator 1) 2, 40kDa	0,0242456	1,456	0,0148603	1,930	1,693
206134_at	**ADAMDEC1**	ADAM-like, decysin 1	0,0242540	14,061	0,0360903	12,272	13,166
208075_s_at	**CCL7**	chemokine (C-C motif) ligand 7	0,0242750	4,384	0,0210357	9,169	6,777
228162_at	**ESD**	esterase D/formylglutathione hydrolase	0,0242791	1,196	0,0048297	1,167	1,181
239102_s_at	**PICALM**	phosphatidylinositol binding clathrin assembly protein	0,0242823	1,376	0,0257500	2,006	1,691
203805_s_at	**FANCA**	Fanconi anemia, complementation group A	0,0243408	1,405	0,0281366	1,256	1,331
1559502_s_at	**LRRC25**	leucine rich repeat containing 25	0,0243979	1,614	0,0412639	1,745	1,679
222774_s_at	**NETO2**	neuropilin (NRP) and tolloid (TLL)-like 2	0,0244102	3,254	0,0036068	5,646	4,450
224797_at	**ARRDC3**	arrestin domain containing 3	0,0244982	1,857	0,0135435	1,370	1,613
240527_at	**RNF170**	ring finger protein 170	0,0245828	1,169	0,0219838	1,213	1,191
207992_s_at	**AMPD3**	adenosine monophosphate deaminase (isoform E)	0,0246183	2,153	0,0348252	2,649	2,401
241965_at	**LARP5**	La ribonucleoprotein domain family, member 5	0,0247135	1,214	0,0223342	1,460	1,337
228846_at	**MXD1**	MAX dimerization protein 1	0,0247393	1,659	0,0298015	2,061	1,860
212685_s_at	**TBL2**	transducin (beta)-like 2	0,0248449	1,316	0,0393484	1,451	1,384
243410_at	**PTPN2**	protein tyrosine phosphatase, non-receptor type 2	0,0248629	1,330	0,0224770	1,282	1,306
218856_at	**TNFRSF21**	tumor necrosis factor receptor superfamily, member 21	0,0248704	2,074	0,0280563	2,694	2,384
221009_s_at	**ANGPTL4**	angiopoietin-like 4	0,0249197	1,998	0,0326676	1,885	1,941
202954_at	**UBE2C**	ubiquitin-conjugating enzyme E2C	0,0249308	2,692	0,0267961	5,245	3,969
242143_at	**RANBP9**	RAN binding protein 9	0,0249930	1,543	0,0256322	1,227	1,385
236816_at	**FLJ13089**	NA	0,0250391	1,332	0,0328483	1,470	1,401
222443_s_at	**RBM8A**	RNA binding motif protein 8A	0,0252500	1,243	0,0232395	1,410	1,327
205114_s_at	**CCL3**	chemokine (C-C motif) ligand 3	0,0252617	20,144	0,0354208	16,613	18,379
236924_at	**GLMN**	glomulin, FKBP associated protein	0,0252987	1,231	0,0086399	1,304	1,267
243524_at	**SLC30A7**	solute carrier family 30 (zinc transporter), member 7	0,0253956	1,710	0,0018180	1,610	1,660
235417_at	**SPOCD1**	SPOC domain containing 1	0,0254022	1,976	0,0053027	2,351	2,163
203401_at	**PRPS2**	phosphoribosyl pyrophosphate synthetase 2	0,0254071	1,432	0,0148603	1,560	1,496
241786_at	**PPP3R1**	protein phosphatase 3 (formerly 2B), regulatory subunit B, 19kDa, alpha isoform (calcineurin B, type I)	0,0254346	1,574	0,0006180	1,658	1,616
229014_at	**FLJ42709**	NA	0,0254395	1,473	0,0007873	2,134	1,803
210110_x_at	**HNRPH3**	heterogeneous nuclear ribonucleoprotein H3 (2H9)	0,0254732	1,298	0,0208800	1,568	1,433
201664_at	**SMC4L1**	SMC4 structural maintenance of chromosomes 4-like 1 (yeast)	0,0255370	1,551	0,0351794	2,596	2,073
243514_at	**WDFY2**	WD repeat and FYVE domain containing 2	0,0255370	1,705	0,0148163	1,272	1,488
201031_s_at	**HNRPH1**	heterogeneous nuclear ribonucleoprotein H1 (H)	0,0255493	1,517	0,0204257	3,017	2,267
205249_at	**EGR2**	early growth response 2 (Krox-20 homolog, Drosophila)	0,0255581	3,344	0,0037809	2,592	2,968

222759_at	**SUV420H1**	suppressor of variegation 4-20 homolog 1 (Drosophila)	0,0256293	1,602	0,0267961	1,942	1,772
222416_at	**ALDH18A1**	aldehyde dehydrogenase 18 family, member A1	0,0257167	1,359	0,0219392	1,579	1,469
229886_at	**FLJ32363**	NA	0,0257167	1,492	0,0300120	2,036	1,764
218913_s_at	**GMIP**	GEM interacting protein	0,0258109	1,638	0,0408482	1,319	1,478
243992_at	**ZNF198**	zinc finger protein 198	0,0259712	1,291	0,0214687	1,274	1,283
201890_at	**RRM2**	ribonucleotide reductase M2 polypeptide	0,0259967	3,180	0,0200268	11,381	7,280
229218_at	**COL1A2**	collagen, type I, alpha 2	0,0260651	1,451	0,0380027	1,323	1,387
225680_at	**DKFZp434K1815**	NA	0,0260675	1,319	0,0200268	1,443	1,381
210119_at	**KCNJ15**	potassium inwardly-rectifying channel, subfamily J, member 15	0,0260712	2,248	0,0097594	2,376	2,312
235959_at	**ARID4B**	AT rich interactive domain 4B (RBP1- like)	0,0261257	1,284	0,0369712	1,219	1,251
220011_at	**C1orf135**	chromosome 1 open reading frame 135	0,0261728	1,561	0,0098933	1,328	1,445
1553292_s_at	**FLJ25006**	NA	0,0261752	1,274	0,0455484	1,314	1,294
204580_at	**MMP12**	matrix metallopeptidase 12 (macrophage elastase)	0,0262438	9,117	0,0341341	11,175	10,146
205825_at	**PCSK1**	proprotein convertase subtilisin/kexin type 1	0,0262704	2,349	0,0235534	3,394	2,872
240656_at	**SIPA1L1**	signal-induced proliferation-associated 1 like 1	0,0262865	1,311	0,0148722	1,286	1,298
206730_at	**GRIA3**	glutamate receptor, ionotrophic, AMPA 3	0,0263467	1,249	0,0245690	2,533	1,891
205476_at	**CCL20**	chemokine (C-C motif) ligand 20	0,0264196	2,327	0,0194857	4,294	3,311
219572_at	**CADPS2**	Ca2+-dependent activator protein for secretion 2	0,0264671	1,670	0,0091542	1,497	1,583
239296_at	**ZFHX1B**	zinc finger homeobox 1b	0,0264672	1,459	0,0332986	1,342	1,401
233323_at	**VTI1A**	vesicle transport through interaction with t-SNAREs homolog 1A (yeast)	0,0265218	1,273	0,0155818	1,313	1,293
242688_at	**TRIP12**	thyroid hormone receptor interactor 12	0,0265383	1,640	0,0244751	1,438	1,539
222592_s_at	**ACSL5**	acyl-CoA synthetase long-chain family member 5	0,0266044	1,306	0,0459600	1,637	1,472
203298_s_at	**JARID2**	Jumonji, AT rich interactive domain 2	0,0266592	1,524	0,0275194	1,483	1,503
226743_at	**FLJ34922**	NA	0,0266733	1,662	0,0200268	1,543	1,603
232012_at	**CAPN1**	calpain 1, (mu/I) large subunit	0,0267208	1,299	0,0238741	1,263	1,281
213135_at	**TIAM1**	T-cell lymphoma invasion and metastasis 1	0,0267208	1,611	0,0273914	1,736	1,673
212687_at	**LIMS1**	LIM and senescent cell antigen-like domains 1	0,0268230	1,314	0,0047166	1,500	1,407
242281_at	**GLUL**	glutamate-ammonia ligase (glutamine synthase)	0,0268232	1,521	0,0090862	2,682	2,102
238534_at	**LRRFIP1**	leucine rich repeat (in FLII) interacting protein 1	0,0268487	1,472	0,0207848	1,165	1,319
204178_s_at	**RBM14**	RNA binding motif protein 14	0,0268561	1,256	0,0241866	1,195	1,226
1557350_at	**G3BP**	NA	0,0268774	1,213	0,0232691	1,508	1,360
203624_at	**DXYS155E**	NA	0,0268908	1,224	0,0093779	1,301	1,262
205786_s_at	**ITGAM**	c("integrin, alpha M (complement component receptor 3, alpha", " also known as CD11b (p170), macrophage antigen alpha polypeptide)")	0,0269066	2,702	0,0339959	1,669	2,186
227066_at	**MOBKL2C**	MOB1, Mps One Binder kinase activator-like 2C (yeast)	0,0269066	1,370	0,0406458	1,430	1,400
228840_at	**AMOTL1**	angiomotin like 1	0,0270666	1,526	0,0298015	1,885	1,705
226472_at	**PPIL4**	peptidylprolyl isomerase (cyclophilin)-like 4	0,0270789	1,319	0,0275012	1,264	1,292
219279_at	**DOCK10**	dedicator of cytokinesis 10	0,0270995	2,037	0,0023647	1,199	1,618
201642_at	**IFNGR2**	interferon gamma receptor 2 (interferon gamma transducer 1)	0,0271940	1,333	0,0342150	1,400	1,367
234973_at	**SLC38A5**	solute carrier family 38, member 5	0,0272018	2,648	0,0339466	3,524	3,086
203765_at	**GCA**	grancalcin, EF-hand calcium binding protein	0,0272314	1,410	0,0185535	1,574	1,492
205518_s_at	**CMAH**	cytidine monophosphate-N-acetylneuraminic acid hydroxylase (CMP-N-acetylneuraminate monooxygenase)	0,0272814	1,477	0,0404236	1,255	1,366
206003_at	**CEP4**	centrosomal protein 4	0,0273449	1,373	0,0411843	1,350	1,362
218070_s_at	**GMPPA**	GDP-mannose pyrophosphorylase A	0,0273691	1,449	0,0293233	1,540	1,495
219848_s_at	**ZNF432**	zinc finger protein 432	0,0274141	1,514	0,0034669	1,448	1,481
202472_at	**MPI**	mannose phosphate isomerase	0,0274169	1,254	0,0362828	1,235	1,244
236007_at	**AKAP10**	A kinase (PRKA) anchor protein 10	0,0275077	1,653	0,0041030	1,463	1,558
212539_at	**CHD1L**	chromodomain helicase DNA binding protein 1-like	0,0275077	1,710	0,0329950	1,864	1,787
242957_at	**FLJ32009**	NA	0,0275077	1,583	0,0023014	1,454	1,519
205828_at	**MMP3**	matrix metallopeptidase 3 (stromelysin 1, progelatinase)	0,0275077	12,763	0,0102449	21,794	17,278
217739_s_at	**PBEF1**	pre-B-cell colony enhancing factor 1	0,0275077	2,166	0,0262205	3,218	2,692
226419_s_at	**SFRS1**	splicing factor, arginine/serine-rich 1 (splicing factor 2, alternate splicing factor)	0,0275559	1,549	0,0200268	2,638	2,094
222939_s_at	**SLC16A10**	solute carrier family 16 (monocarboxylic acid transporters), member 10	0,0275559	5,352	0,0105117	5,281	5,316
241435_at	**ETS1**	v-ets erythroblastosis virus E26 oncogene homolog 1 (avian)	0,0276205	1,336	0,0041196	1,692	1,514

201037_at	**PFKP**	phosphofructokinase, platelet	0,0276297	1,795	0,0463213	1,583	1,689
233388_at	**CA12**	carbonic anhydrase XII	0,0277454	1,976	0,0252534	1,436	1,706
213274_s_at	**CTSB**	cathepsin B	0,0277599	1,655	0,0310055	1,678	1,666
238462_at	**STS-1**	NA	0,0278156	2,552	0,0332741	2,916	2,734
205767_at	**EREG**	epiregulin	0,0278365	2,174	0,0233835	2,048	2,111
227701_at	**C10orf118**	chromosome 10 open reading frame 118	0,0278592	1,405	0,0260278	1,264	1,334
1555970_at	**FBXO28**	F-box protein 28	0,0279451	1,486	0,0357973	1,412	1,449
222411_s_at	**SSR3**	signal sequence receptor, gamma (translocon-associated protein gamma)	0,0279776	1,558	0,0307178	1,641	1,599
239283_at	**TMED5**	transmembrane emp24 protein transport domain containing 5	0,0280456	1,180	0,0454006	1,312	1,246
202406_s_at	**TIAL1**	TIA1 cytotoxic granule-associated RNA binding protein-like 1	0,0281758	1,170	0,0214687	1,129	1,150
221520_s_at	**CDCA8**	cell division cycle associated 8	0,0282664	1,934	0,0079793	2,234	2,084
229264_at	**FLJ39739**	NA	0,0282892	1,519	0,0218822	1,412	1,466
212185_x_at	**MT2A**	metallothionein 2A	0,0283244	1,624	0,0396590	1,849	1,736
209301_at	**CA2**	carbonic anhydrase II	0,0284317	4,166	0,0328864	4,336	4,251
243851_at	**RAB3GAP2**	RAB3 GTPase activating protein subunit 2 (non-catalytic)	0,0284465	1,313	0,0150541	1,254	1,283
219510_at	**POLQ**	polymerase (DNA directed), theta	0,0285894	1,544	0,0380511	1,897	1,721
1560622_at	**TPARL**	NA	0,0286122	1,651	0,0240391	1,413	1,532
212588_at	**PTPRC**	protein tyrosine phosphatase, receptor type, C	0,0286735	8,696	0,0376245	11,297	9,997
1555628_a_at	**HAVCR2**	hepatitis A virus cellular receptor 2	0,0287614	1,224	0,0159805	1,322	1,273
1558783_at	**WTAP**	Wilms tumor 1 associated protein	0,0288160	1,514	0,0220778	1,655	1,585
219512_at	**C20orf172**	chromosome 20 open reading frame 172	0,0288623	1,388	0,0349218	1,572	1,480
218257_s_at	**UGCGL1**	UDP-glucose ceramide glucosyltransferase-like 1	0,0288849	1,284	0,0257500	1,407	1,345
210997_at	**HGF**	c("hepatocyte growth factor (hepapoietin A", " scatter factor)")	0,0289263	2,962	0,0000517	3,954	3,458
222282_at	**PAPD4**	PAP associated domain containing 4	0,0289378	1,478	0,0349203	1,539	1,508
209648_x_at	**SOCS5**	suppressor of cytokine signaling 5	0,0289709	1,306	0,0442596	1,432	1,369
216306_x_at	**PTBP1**	polypyrimidine tract binding protein 1	0,0289832	1,244	0,0218822	1,792	1,518
235821_at	**WISP1**	WNT1 inducible signaling pathway protein 1	0,0289913	1,374	0,0220778	2,444	1,909
214221_at	**ALMS1**	Alstrom syndrome 1	0,0290771	1,428	0,0064069	1,712	1,570
232476_at	**ITGB1BP1**	integrin beta 1 binding protein 1	0,0292001	1,262	0,0216908	1,215	1,238
212038_s_at	**VDAC1**	voltage-dependent anion channel 1	0,0292154	1,347	0,0246837	1,624	1,486
217787_s_at	**GALNT2**	UDP-N-acetyl-alpha-D-galactosamine:polypeptide N-acetylgalactosaminyltransferase 2 (GalNAc-T2)	0,0292566	1,473	0,0267961	1,867	1,670
201849_at	**BNIP3**	BCL2/adenovirus E1B 19kDa interacting protein 3	0,0293068	1,530	0,0398000	1,641	1,585
213194_at	**ROBO1**	roundabout, axon guidance receptor, homolog 1 (Drosophila)	0,0293688	1,417	0,0269841	1,716	1,566
215513_at	**HYMAI**	hydatidiform mole associated and imprinted	0,0293707	1,269	0,0395343	1,176	1,223
1559038_at	**SEPT2p**	septin 2	0,0293888	1,505	0,0248596	1,886	1,695
221768_at	**SFPQ**	splicing factor proline/glutamine-rich (polypyrimidine tract binding protein associated)	0,0294187	1,888	0,0011580	1,742	1,815
204549_at	**IKBKE**	inhibitor of kappa light polypeptide gene enhancer in B-cells, kinase epsilon	0,0294497	1,407	0,0313920	1,201	1,304
226661_at	**CDCA2**	cell division cycle associated 2	0,0294532	2,792	0,0274382	4,683	3,737
208897_s_at	**DDX18**	DEAD (Asp-Glu-Ala-Asp) box polypeptide 18	0,0294532	1,186	0,0044925	1,213	1,200
217028_at	**CXCR4**	chemokine (C-X-C motif) receptor 4	0,0294689	4,960	0,0466245	1,725	3,342
229070_at	**C6orf105**	chromosome 6 open reading frame 105	0,0295919	1,878	0,0438578	2,936	2,407
203828_s_at	**IL32**	interleukin 32	0,0295919	5,380	0,0277977	5,521	5,451
207002_s_at	**PLAGL1**	pleiomorphic adenoma gene-like 1	0,0296723	1,517	0,0065712	1,541	1,529
203439_s_at	**STC2**	stanniocalcin 2	0,0297351	1,914	0,0293084	2,370	2,142
231107_at	**RSU1**	Ras suppressor protein 1	0,0297772	1,457	0,0193268	1,193	1,325
209686_at	**S100B**	S100 calcium binding protein, beta (neural)	0,0297772	2,213	0,0074775	2,516	2,364
213975_s_at	**LILRB1**	leukocyte immunoglobulin-like receptor, subfamily B (with TM and ITIM domains), member 1	0,0298418	6,293	0,0204257	7,899	7,096
223969_s_at	**RETNLB**	resistin like beta	0,0299654	1,195	0,0070073	1,191	1,193
204026_s_at	**ZWINT**	ZW10 interactor	0,0300001	2,222	0,0247900	4,025	3,123
204254_s_at	**VDR**	vitamin D (1,25- dihydroxyvitamin D3) receptor	0,0300596	1,665	0,0233020	2,072	1,869
204299_at	**FUSIP1**	FUS interacting protein (serine/arginine-rich) 1	0,0300892	1,284	0,0042803	1,407	1,346
231779_at	**IRAK2**	interleukin-1 receptor-associated kinase 2	0,0300983	1,498	0,0269841	2,184	1,841
204531_s_at	**BRCA1**	breast cancer 1, early onset	0,0301793	1,704	0,0254363	1,254	1,479
227638_at	**KIAA1632**	KIAA1632	0,0301865	1,397	0,0408574	1,331	1,364
232270_at	**C9orf3**	chromosome 9 open reading frame 3	0,0301989	1,542	0,0347857	1,445	1,493
223097_at	**ADPRHL2**	ADP-ribosylhydrolase like 2	0,0303253	1,218	0,0277977	1,188	1,203
239489_at	**UBLCP1**	ubiquitin-like domain containing CTD phosphatase 1	0,0303253	1,129	0,0241266	1,309	1,219
213418_at	**HSPA6**	heat shock 70kDa protein 6 (HSP70B')	0,0303470	1,721	0,0133749	1,434	1,578

213256_at	**MARCH3p**	membrane-associated ring finger (C3HC4) 3	0,0303470	1,645	0,0440300	2,180	1,913
221031_s_at	**DKFZP434F0318**	NA	0,0303533	1,679	0,0255380	2,494	2,087
204475_at	**MMP1**	matrix metallopeptidase 1 (interstitial collagenase)	0,0303966	15,487	0,0417091	24,934	20,210
226452_at	**PDK1**	pyruvate dehydrogenase kinase, isoenzyme 1	0,0304618	2,240	0,0230671	2,100	2,170
219540_at	**ZNF267**	zinc finger protein 267	0,0304697	1,510	0,0171176	2,258	1,884
221923_s_at	**NPM1**	nucleophosmin (nucleolar phosphoprotein B23, numatrin)	0,0305628	1,248	0,0204257	1,490	1,369
226264_at	**SUSD1**	sushi domain containing 1	0,0305892	1,339	0,0380151	1,114	1,227
219203_at	**C14orf122**	chromosome 14 open reading frame 122	0,0305917	1,401	0,0288812	1,292	1,346
225024_at	**C20orf77**	chromosome 20 open reading frame 77	0,0305917	1,189	0,0403429	1,151	1,170
203312_x_at	**ARF6**	ADP-ribosylation factor 6	0,0306893	1,287	0,0148522	1,323	1,305
230360_at	**COLM**	collomin	0,0307073	2,531	0,0267854	1,765	2,148
222316_at	**VDP**	NA	0,0307578	1,528	0,0328367	1,510	1,519
238067_at	**FLJ20298**	NA	0,0308618	1,490	0,0091542	1,556	1,523
209892_at	**FUT4**	fucosyltransferase 4 (alpha (1,3) fucosyltransferase, myeloid-specific)	0,0308618	1,396	0,0315703	1,398	1,397
1554116_s_at	**PARP11**	poly (ADP-ribose) polymerase family, member 11	0,0308618	1,159	0,0341109	1,373	1,266
1557649_at	**GALE**	UDP-galactose-4-epimerase	0,0309291	1,320	0,0297633	1,449	1,385
235410_at	**NPHP3**	nephronophthisis 3 (adolescent)	0,0310018	1,413	0,0030466	1,433	1,423
221840_at	**PTPRE**	protein tyrosine phosphatase, receptor type, E	0,0310018	2,406	0,0342078	2,803	2,604
244239_at	**ANKH**	ankylosis, progressive homolog (mouse)	0,0310418	1,250	0,0287719	1,804	1,527
217395_at	**MT4**	metallothionein IV	0,0310565	1,267	0,0066543	1,189	1,228
222719_s_at	**PDGFC**	platelet derived growth factor C	0,0311014	2,069	0,0413014	2,636	2,353
208109_s_at	**C15orf5**	chromosome 15 open reading frame 5	0,0311432	1,897	0,0036915	1,654	1,776
204252_at	**CDK2**	cyclin-dependent kinase 2	0,0311483	1,771	0,0183676	1,880	1,825
37577_at	**ARHGAP19**	Rho GTPase activating protein 19	0,0311841	1,572	0,0337287	1,628	1,600
229473_at	**MAMDC4**	MAM domain containing 4	0,0312276	1,325	0,0416530	1,408	1,366
214030_at	**DKFZp667G2110**	NA	0,0312703	1,374	0,0113371	1,213	1,294
205598_at	**TRIP**	NA	0,0313327	1,343	0,0432226	1,457	1,400
209832_s_at	**CDT1**	NA	0,0313954	1,342	0,0281208	1,809	1,576
242225_at	**KIAA1040**	NA	0,0314052	1,477	0,0140517	1,185	1,331
223096_at	**NOP5/NOP58**	NA	0,0314155	1,388	0,0002114	1,737	1,562
201261_x_at	**BGN**	biglycan	0,0314314	1,293	0,0161546	1,405	1,349
212671_s_at	**HLA-DQA1**	major histocompatibility complex, class II, DQ alpha 1	0,0314766	7,322	0,0332561	10,341	8,832
223121_s_at	**SFRP2**	secreted frizzled-related protein 2	0,0314867	2,257	0,0345016	3,361	2,809
225655_at	**UHRF1**	ubiquitin-like, containing PHD and RING finger domains, 1	0,0314913	2,538	0,0234025	3,834	3,186
232865_at	**AFF4**	AF4/FMR2 family, member 4	0,0315338	1,665	0,0412018	2,232	1,948
202183_s_at	**KIF22**	kinesin family member 22	0,0315338	1,698	0,0231727	1,801	1,750
211945_s_at	**ITGB1**	integrin, beta 1 (fibronectin receptor, beta polypeptide, antigen CD29 includes MDF2, MSK12)	0,0315750	1,097	0,0332783	1,473	1,285
239892_at	**RARS**	arginyl-tRNA synthetase	0,0316045	1,237	0,0330502	1,255	1,246
241509_at	**PLEKHA5**	pleckstrin homology domain containing, family A member 5	0,0316951	1,142	0,0121137	1,393	1,268
221261_x_at	**MAGED4**	melanoma antigen family D, 4	0,0317992	1,366	0,0265466	1,678	1,522
238846_at	**TNFRSF11A**	tumor necrosis factor receptor superfamily, member 11a, NFKB activator	0,0318346	1,338	0,0066656	1,257	1,298
203868_s_at	**VCAM1**	vascular cell adhesion molecule 1	0,0318703	1,338	0,0363103	1,460	1,399
241681_at	**MBNL1**	muscleblind-like (Drosophila)	0,0318889	2,314	0,0416617	2,249	2,282
202422_s_at	**ACSL4**	acyl-CoA synthetase long-chain family member 4	0,0319849	1,703	0,0248558	1,977	1,840
234837_at	**MSRA**	methionine sulfoxide reductase A	0,0321079	1,141	0,0437671	1,196	1,168
236978_at	**THRAP1**	thyroid hormone receptor associated protein 1	0,0321319	1,568	0,0401584	1,600	1,584
39402_at	**IL1B**	interleukin 1, beta	0,0321479	10,992	0,0384583	29,076	20,034
202917_s_at	**S100A8**	S100 calcium binding protein A8 (calgranulin A)	0,0321488	17,922	0,0401473	50,671	34,296
219308_s_at	**AK5**	adenylate kinase 5	0,0321790	2,034	0,0396536	2,396	2,215
226840_at	**H2AFY**	H2A histone family, member Y	0,0321937	1,210	0,0267294	1,205	1,208
213287_s_at	**KRT10**	c("keratin 10 (epidermolytic hyperkeratosis", " keratosis palmaris et plantaris)")	0,0321937	1,284	0,0000313	1,530	1,407
200737_at	**PGK1**	phosphoglycerate kinase 1	0,0322022	1,661	0,0319202	1,657	1,659
38158_at	**ESPL1**	extra spindle poles like 1 (S. cerevisiae)	0,0322173	1,687	0,0247572	1,942	1,814
218894_s_at	**FLJ10292**	NA	0,0322544	1,421	0,0238680	1,840	1,631
206298_at	**ARHGAP22**	Rho GTPase activating protein 22	0,0322694	1,793	0,0280501	1,896	1,844
231832_at	**GALNT4**	UDP-N-acetyl-alpha-D-galactosamine:polypeptide N-acetylgalactosaminyltransferase 4 (GalNAc-T4)	0,0322778	1,445	0,0024017	1,583	1,514
209754_s_at	**TMPO**	thymopoietin	0,0322990	1,580	0,0188412	2,104	1,842
222780_s_at	**BAALC**	brain and acute leukemia, cytoplasmic	0,0323133	2,244	0,0230671	4,084	3,164
210621_s_at	**RASA1**	RAS p21 protein activator (GTPase activating protein) 1	0,0323133	1,863	0,0380710	2,260	2,061

203580_s_at	**SLC7A6**	solute carrier family 7 (cationic amino acid transporter, y+ system), member 6	0,0323530	1,401	0,0367058	1,461	1,431
223303_at	**URP2**	NA	0,0323608	1,837	0,0354872	1,713	1,775
226163_at	**ZBTB9**	zinc finger and BTB domain containing 9	0,0323807	1,206	0,0400482	1,120	1,163
244373_at	**RAF1**	v-raf-1 murine leukemia viral oncogene homolog 1	0,0324051	1,331	0,0229213	1,230	1,281
60474_at	**C20orf42**	chromosome 20 open reading frame 42	0,0324340	1,596	0,0200268	2,049	1,822
201292_at	**TOP2A**	topoisomerase (DNA) II alpha 170kDa	0,0324340	2,866	0,0217985	7,262	5,064
229383_at	**MARCH1p**	membrane-associated ring finger (C3HC4) 1	0,0324358	1,599	0,0322687	1,815	1,707
202192_s_at	**GAS7**	growth arrest-specific 7	0,0324920	1,973	0,0043943	2,012	1,992
204638_at	**ACP5**	acid phosphatase 5, tartrate resistant	0,0326659	4,160	0,0253918	2,652	3,406
221778_at	**KIAA1718**	NA	0,0326659	1,346	0,0231238	1,438	1,392
202466_at	**POLS**	polymerase (DNA directed) sigma	0,0326791	1,318	0,0424384	1,229	1,274
209891_at	**SPBC25**	spindle pole body component 25 homolog (S. cerevisiae)	0,0327550	3,094	0,0369168	6,161	4,628
222631_at	**PI4K2B**	phosphatidylinositol 4-kinase type 2 beta	0,0327705	1,561	0,0238454	1,723	1,642
200969_at	**SERP1**	NA	0,0327705	1,432	0,0281208	1,544	1,488
227751_at	**PDCD5**	programmed cell death 5	0,0328272	1,251	0,0324256	1,466	1,359
209712_at	**SLC35D1**	solute carrier family 35 (UDP-glucuronic acid/UDP-N-acetylgalactosamine dual transporter), member D1	0,0328980	1,283	0,0139671	1,246	1,264
232883_at	**SOS1**	son of sevenless homolog 1 (Drosophila)	0,0330888	1,186	0,0015879	1,104	1,145
224692_at	**PPP1R15B**	protein phosphatase 1, regulatory (inhibitor) subunit 15B	0,0331128	1,220	0,0171966	1,302	1,261
222848_at	**FKSG14**	NA	0,0331828	2,406	0,0300721	4,941	3,673
200916_at	**TAGLN2**	transgelin 2	0,0331974	1,670	0,0349848	2,122	1,896
203761_at	**SLA**	Src-like-adaptor	0,0334410	2,384	0,0338806	3,072	2,728
205566_at	**ABHD2**	abhydrolase domain containing 2	0,0334459	1,729	0,0006629	2,421	2,075
1558467_a_at	**UGCGL2**	UDP-glucose ceramide glucosyltransferase-like 2	0,0335273	1,246	0,0258354	1,726	1,486
233943_x_at	**PLEKHA8**	pleckstrin homology domain containing, family A (phosphoinositide binding specific) member 8	0,0335577	1,127	0,0367936	1,357	1,242
204612_at	**PKIA**	protein kinase (cAMP-dependent, catalytic) inhibitor alpha	0,0335962	1,630	0,0291260	2,090	1,860
211974_x_at	**RBPSUH**	recombining binding protein suppressor of hairless (Drosophila)	0,0335962	1,600	0,0002160	1,848	1,724
205733_at	**BLM**	Bloom syndrome	0,0336421	1,567	0,0399846	1,920	1,744
236010_at	**SH3D19**	NA	0,0337159	1,101	0,0227628	1,531	1,316
242865_at	**SDFR1**	stromal cell derived factor receptor 1	0,0337331	1,836	0,0436321	1,297	1,566
228570_at	**BTBD11**	BTB (POZ) domain containing 11	0,0338233	1,367	0,0303396	1,377	1,372
240600_at	**AP3B1**	adaptor-related protein complex 3, beta 1 subunit	0,0338402	1,436	0,0050470	1,241	1,339
223843_at	**SCARA3**	scavenger receptor class A, member 3	0,0338912	1,648	0,0444337	1,299	1,474
242068_at	**BIRC6**	baculoviral IAP repeat-containing 6 (apollon)	0,0339250	1,703	0,0102434	1,329	1,516
202968_s_at	**DYRK2**	dual-specificity tyrosine-(Y)-phosphorylation regulated kinase 2	0,0339526	1,350	0,0146774	1,378	1,364
211395_x_at	**FCGR2C**	NA	0,0340057	1,763	0,0166193	1,790	1,777
214981_at	**POSTN**	periostin, osteoblast specific factor	0,0340057	2,082	0,0219131	2,466	2,274
225282_at	**SMAP1L**	stromal membrane-associated protein 1-like	0,0340360	1,378	0,0388620	1,383	1,380
201105_at	**LGALS1**	lectin, galactoside-binding, soluble, 1 (galectin 1)	0,0340725	1,073	0,0273763	1,110	1,092
240452_at	**GSPT1**	G1 to S phase transition 1	0,0340863	1,608	0,0207511	1,487	1,547
209680_s_at	**KIFC1**	kinesin family member C1	0,0340915	1,460	0,0218822	1,744	1,602
226459_at	**PIK3AP1**	phosphoinositide-3-kinase adaptor protein 1	0,0342065	4,502	0,0364140	4,679	4,590
218818_at	**FHL3**	four and a half LIM domains 3	0,0343153	1,432	0,0139261	1,367	1,399
225740_x_at	**MDM4**	Mdm4, transformed 3T3 cell double minute 4, p53 binding protein (mouse)	0,0343420	1,468	0,0019082	1,416	1,442
227485_at	**DDX26B**	DEAD/H (Asp-Glu-Ala-Asp/His) box polypeptide 26B	0,0343559	1,385	0,0106579	1,314	1,350
225368_at	**HIPK2**	homeodomain interacting protein kinase 2	0,0343559	1,502	0,0204257	1,795	1,649
222030_at	**SIVA**	NA	0,0344156	1,269	0,0441293	1,121	1,195
236188_s_at	**NAP1L4**	nucleosome assembly protein 1-like 4	0,0345135	1,188	0,0368005	1,316	1,252
207668_x_at	**PDIA6**	protein disulfide isomerase family A, member 6	0,0345146	1,303	0,0306682	1,563	1,433
213334_x_at	**TREX2**	three prime repair exonuclease 2	0,0346490	1,153	0,0427028	1,291	1,222
1553956_at	**ALS2CR4**	amyotrophic lateral sclerosis 2 (juvenile) chromosome region, candidate 4	0,0346830	1,321	0,0026867	1,360	1,340
239519_at	**NRP1**	neuropilin 1	0,0348999	1,249	0,0232153	1,233	1,241
219433_at	**BCOR**	BCL6 co-repressor	0,0349287	1,430	0,0231238	1,381	1,405
223472_at	**WHSC1**	Wolf-Hirschhorn syndrome candidate 1	0,0349731	1,129	0,0309596	1,869	1,499
210232_at	**CDC42**	cell division cycle 42 (GTP binding protein, 25kDa)	0,0349841	1,251	0,0217832	1,194	1,223
216834_at	**RGS1**	regulator of G-protein signalling 1	0,0349841	11,466	0,0302308	13,730	12,598
227502_at	**LCHN**	NA	0,0352051	1,283	0,0258524	1,202	1,243
200893_at	**SFRS10**	splicing factor, arginine/serine-rich 10 (transformer 2 homolog, Drosophila)	0,0352446	1,323	0,0154426	1,898	1,610

212850_s_at	**LRP4**	low density lipoprotein receptor-related protein 4	0,0353511	1,478	0,0016014	1,670	1,574
1558097_at	**H1FX**	H1 histone family, member X	0,0353800	1,229	0,0298630	1,120	1,175
203359_s_at	**MYCBP**	c-myc binding protein	0,0353800	2,794	0,0281208	3,291	3,043
208688_x_at	**EIF3S9**	eukaryotic translation initiation factor 3, subunit 9 eta, 116kDa	0,0354290	1,262	0,0166681	1,317	1,289
225047_at	**NUPL1**	nucleoporin like 1	0,0354744	1,615	0,0006153	1,714	1,664
243894_at	**SLC41A2**	solute carrier family 41, member 2	0,0354885	1,521	0,0215451	1,629	1,575
232371_at	**MARCH7p**	membrane-associated ring finger (C3HC4) 7	0,0355617	1,268	0,0277977	1,415	1,342
209728_at	**HLA-DRB4**	major histocompatibility complex, class II, DR beta 4	0,0355675	3,489	0,0311971	4,128	3,809
206215_at	**OPCML**	opioid binding protein/cell adhesion molecule-like	0,0355675	1,592	0,0421464	2,251	1,922
222264_at	**DKFZp762N1910**	NA	0,0355846	1,198	0,0138290	1,729	1,464
205351_at	**GGCX**	gamma-glutamyl carboxylase	0,0355994	1,242	0,0083638	1,457	1,350
218923_at	**CTBS**	chitobiase, di-N-acetyl-	0,0356730	1,357	0,0325509	1,530	1,443
229633_at	**C8orf35**	chromosome 8 open reading frame 35	0,0357167	1,281	0,0155782	1,262	1,272
208241_at	**NRG1**	neuregulin 1	0,0357532	1,425	0,0275336	3,182	2,303
226302_at	**ATP8B1**	ATPase, Class I, type 8B, member 1	0,0359607	1,832	0,0287473	1,385	1,609
225773_at	**KIAA1972**	NA	0,0360387	1,223	0,0000000	1,227	1,225
209823_x_at	**HLA-DQB1**	major histocompatibility complex, class II, DQ beta 1	0,0360567	2,252	0,0344687	1,688	1,970
230522_s_at	**C9orf100**	chromosome 9 open reading frame 100	0,0361787	1,220	0,0316698	1,132	1,176
1566605_at	**TEX9**	testis expressed sequence 9	0,0361864	1,071	0,0306682	1,530	1,301
206111_at	**RNASE2**	ribonuclease, RNase A family, 2 (liver, eosinophil-derived neurotoxin)	0,0362454	1,270	0,0241566	1,232	1,251
226438_at	**SNTB1**	syntrophin, beta 1 (dystrophin-associated protein A1, 59kDa, basic component 1)	0,0362454	1,436	0,0050470	1,594	1,515
37012_at	**CAPZB**	capping protein (actin filament) muscle Z-line, beta	0,0362642	1,349	0,0320653	2,028	1,689
203484_at	**SEC61G**	Sec61 gamma subunit	0,0363835	1,348	0,0269981	1,440	1,394
210538_s_at	**BIRC3**	baculoviral IAP repeat-containing 3	0,0363989	1,793	0,0080815	2,912	2,353
207551_s_at	**MSL3L1**	male-specific lethal 3-like 1 (Drosophila)	0,0363989	1,233	0,0322284	1,219	1,226
210861_s_at	**WISP3**	WNT1 inducible signaling pathway protein 3	0,0365329	1,842	0,0400474	1,665	1,754
208322_s_at	**ST3GAL1**	ST3 beta-galactoside alpha-2,3-sialyltransferase 1	0,0366057	1,430	0,0356718	1,629	1,530
227420_at	**TNFAIP8L1**	tumor necrosis factor, alpha-induced protein 8-like 1	0,0366914	1,367	0,0321084	1,360	1,363
224839_s_at	**GPT2**	glutamic pyruvate transaminase (alanine aminotransferase) 2	0,0367205	1,567	0,0315475	1,564	1,566
243579_at	**MSI2**	musashi homolog 2 (Drosophila)	0,0367469	1,133	0,0432226	1,244	1,189
230332_at	**ZCCHC7**	zinc finger, CCHC domain containing 7	0,0367549	2,099	0,0425036	1,683	1,891
242678_at	**LPP**	LIM domain containing preferred translocation partner in lipoma	0,0368030	1,111	0,0197488	1,487	1,299
235548_at	**FLJ90166**	NA	0,0369618	1,674	0,0328732	1,963	1,818
217650_x_at	**ST3GAL2**	ST3 beta-galactoside alpha-2,3-sialyltransferase 2	0,0370615	1,223	0,0129269	1,233	1,228
217546_at	**MT1M**	metallothionein 1M	0,0371552	3,330	0,0072349	8,388	5,859
243088_at	**C2orf4**	chromosome 2 open reading frame 4	0,0371557	1,363	0,0220949	1,278	1,321
218723_s_at	**RGC32**	NA	0,0372342	3,552	0,0218355	11,065	7,309
223546_x_at	**LUC7L**	LUC7-like (S. cerevisiae)	0,0372850	1,221	0,0182334	1,524	1,372
206186_at	**MPP3**	membrane protein, palmitoylated 3 (MAGUK p55 subfamily member 3)	0,0373151	1,203	0,0252810	1,206	1,205
205568_at	**AQP9**	aquaporin 9	0,0373658	3,092	0,0444615	3,594	3,343
218332_at	**BEX1**	brain expressed, X-linked 1	0,0375669	2,431	0,0316960	4,669	3,550
212927_at	**SMC5L1**	SMC5 structural maintenance of chromosomes 5-like 1 (yeast)	0,0376615	1,448	0,0002256	1,656	1,552
222859_s_at	**DAPP1**	dual adaptor of phosphotyrosine and 3-phosphoinositides	0,0376685	1,406	0,0293101	1,129	1,268
1557505_a_at	**SRP54**	signal recognition particle 54kDa	0,0377167	1,159	0,0280563	1,425	1,292
226818_at	**MPEG1**	NA	0,0378028	4,862	0,0437671	8,768	6,815
208018_s_at	**HCK**	hemopoietic cell kinase	0,0378550	2,886	0,0219456	3,092	2,989
203664_s_at	**POLR2D**	polymerase (RNA) II (DNA directed) polypeptide D	0,0378550	1,329	0,0218822	1,472	1,401
1557738_at	**RAB6IP2**	RAB6 interacting protein 2	0,0380591	1,229	0,0395334	1,190	1,209
227645_at	**PIK3R5**	phosphoinositide-3-kinase, regulatory subunit 5, p101	0,0382061	2,226	0,0196913	2,158	2,192
214157_at	**GNAS**	GNAS complex locus	0,0382396	1,175	0,0139594	1,229	1,202
227828_s_at	**FLJ13391**	NA	0,0382982	1,661	0,0371870	2,182	1,921
240154_at	**WASPIP**	Wiskott-Aldrich syndrome protein interacting protein	0,0383575	1,244	0,0170496	1,672	1,458
235985_at	**PITPNB**	phosphatidylinositol transfer protein, beta	0,0383822	1,707	0,0002160	1,534	1,621
201204_s_at	**RRBP1**	ribosome binding protein 1 homolog 180kDa (dog)	0,0384503	1,523	0,0177951	1,457	1,490
227184_at	**FLJ13213**	NA	0,0385069	1,477	0,0030835	1,300	1,389
230069_at	**SFXN1**	sideroflexin 1	0,0385454	1,606	0,0300040	1,748	1,677

244070_at	**SYNE1**	spectrin repeat containing, nuclear envelope 1	0,0386179	1,381	0,0436863	1,897	1,639
229560_at	**TLR8**	toll-like receptor 8	0,0386240	6,736	0,0261195	6,655	6,695
1568611_at	**P4HA2**	procollagen-proline, 2-oxoglutarate 4-dioxygenase (proline 4-hydroxylase), alpha polypeptide II	0,0386523	1,837	0,0155818	1,690	1,764
236492_at	**PPP2R2A**	protein phosphatase 2 (formerly 2A), regulatory subunit B (PR 52), alpha isoform	0,0386523	1,366	0,0443519	1,368	1,367
1561394_s_at	**KIAA1755**	NA	0,0386750	1,405	0,0111232	1,425	1,415
228335_at	**CLDN11**	claudin 11 (oligodendrocyte transmembrane protein)	0,0386840	3,358	0,0055359	4,761	4,060
224399_at	**PDCD1LG2**	programmed cell death 1 ligand 2	0,0386841	1,704	0,0289842	2,921	2,312
219410_at	**TMEM45A**	transmembrane protein 45A	0,0387275	1,491	0,0281208	1,455	1,473
201920_at	**SLC20A1**	solute carrier family 20 (phosphate transporter), member 1	0,0388830	1,615	0,0014688	2,384	1,999
208249_s_at	**TGDS**	TDP-glucose 4,6-dehydratase	0,0389034	1,355	0,0039931	1,410	1,383
212351_at	**EIF2B5**	eukaryotic translation initiation factor 2B, subunit 5 epsilon, 82kDa	0,0390072	1,179	0,0453789	1,180	1,179
221648_s_at	**C1orf121**	chromosome 1 open reading frame 121	0,0390381	1,627	0,0327520	1,573	1,600
239233_at	**KIAA1212**	KIAA1212	0,0390419	1,160	0,0355988	1,756	1,458
1569041_at	**JMJD1C**	jumonji domain containing 1C	0,0390781	1,290	0,0404117	1,377	1,333
239448_at	**SMAD3**	SMAD, mothers against DPP homolog 3 (Drosophila)	0,0391820	1,977	0,0016672	2,507	2,242
230030_at	**HS6ST2**	heparan sulfate 6-O-sulfotransferase 2	0,0391994	1,165	0,0104686	1,388	1,276
241026_at	**ADAM12**	ADAM metallopeptidase domain 12 (meltrin alpha)	0,0392045	1,410	0,0208800	2,322	1,866
235847_at	**TEX27**	testis expressed sequence 27	0,0392316	1,378	0,0075743	1,211	1,294
212548_s_at	**KIAA0826**	KIAA0826	0,0392943	1,194	0,0462557	1,494	1,344
225853_at	**GNPNAT1**	glucosamine-phosphate N-acetyltransferase 1	0,0393139	1,558	0,0090508	1,705	1,631
209335_at	**DCN**	decorin	0,0393750	1,283	0,0146825	1,428	1,355
1556416_s_at	**SEC31L1**	SEC31-like 1 (S. cerevisiae)	0,0393750	1,485	0,0153941	1,194	1,340
215855_s_at	**TMF1**	TATA element modulatory factor 1	0,0393750	1,214	0,0000000	1,355	1,284
227486_at	**NT5E**	5'-nucleotidase, ecto (CD73)	0,0394151	1,901	0,0206042	2,182	2,042
218506_x_at	**N-PAC**	NA	0,0394578	1,192	0,0256553	1,181	1,187
222018_at	**NACA**	nascent-polypeptide-associated complex alpha polypeptide	0,0396204	1,454	0,0224001	1,322	1,388
236408_at	**KLHDC5**	kelch domain containing 5	0,0398152	1,137	0,0207354	1,578	1,357
236034_at	**ANGPT2**	angiopoietin 2	0,0398537	1,176	0,0111535	1,166	1,171
222162_s_at	**ADAMTS1**	ADAM metallopeptidase with thrombospondin type 1 motif, 1	0,0398924	1,828	0,0200233	1,536	1,682
1557527_at	**RUNX1**	c("runt-related transcription factor 1 (acute myeloid leukemia 1", " aml1 oncogene)")	0,0399118	1,562	0,0280031	1,446	1,504
213054_at	**KIAA0841**	KIAA0841	0,0399466	1,254	0,0221668	1,269	1,261
205583_s_at	**CXorf45**	chromosome X open reading frame 45	0,0400566	1,282	0,0451026	1,381	1,331
218828_at	**PLSCR3**	phospholipid scramblase 3	0,0401370	1,167	0,0275336	1,397	1,282
213262_at	**SACS**	spastic ataxia of Charlevoix-Saguenay (sacsin)	0,0401934	1,709	0,0019908	1,960	1,834
204393_s_at	**ACPP**	acid phosphatase, prostate	0,0402176	1,316	0,0048596	1,326	1,321
239809_at	**KLF7**	Kruppel-like factor 7 (ubiquitous)	0,0402176	1,763	0,0380925	1,812	1,788
205596_s_at	**SMURF2**	SMAD specific E3 ubiquitin protein ligase 2	0,0403074	1,595	0,0323772	1,367	1,481
1557120_at	**EEF1A1**	eukaryotic translation elongation factor 1 alpha 1	0,0403088	1,199	0,0136029	1,101	1,150
226142_at	**GLIPR1**	GLI pathogenesis-related 1 (glioma)	0,0403898	1,596	0,0371697	1,766	1,681
229864_at	**FLJ20211**	NA	0,0403954	1,488	0,0253269	1,381	1,434
215195_at	**PRKCA**	protein kinase C, alpha	0,0404389	1,634	0,0393484	1,933	1,784
210113_s_at	**NALP1**	NACHT, leucine rich repeat and PYD (pyrin domain) containing 1	0,0404828	1,454	0,0230924	1,362	1,408
232362_at	**CCDC18**	coiled-coil domain containing 18	0,0405048	1,250	0,0322071	1,236	1,243
219947_at	**CLEC4A**	C-type lectin domain family 4, member A	0,0405546	2,168	0,0312433	1,482	1,825
205306_x_at	**KMO**	kynurenine 3-monooxygenase (kynurenine 3-hydroxylase)	0,0405878	3,614	0,0236856	3,243	3,428
242837_at	**SFRS4**	splicing factor, arginine/serine-rich 4	0,0405940	1,632	0,0036169	1,569	1,600
223494_at	**MGEA5**	meningioma expressed antigen 5 (hyaluronidase)	0,0406114	1,638	0,0200268	1,666	1,652
208981_at	**PECAM1**	platelet/endothelial cell adhesion molecule (CD31 antigen)	0,0406114	3,395	0,0219957	3,949	3,672
214684_at	**MEF2A**	MADS box transcription enhancer factor 2, polypeptide A (myocyte enhancer factor 2A)	0,0406733	1,902	0,0126168	2,066	1,984
220942_x_at	**E2IG5**	NA	0,0407428	1,313	0,0270883	1,356	1,334
204715_at	**PANX1**	pannexin 1	0,0407761	1,303	0,0195015	1,624	1,464
241226_at	**CDA08**	NA	0,0409678	1,091	0,0118873	1,255	1,173
225539_at	**ZNF295**	zinc finger protein 295	0,0410103	1,439	0,0059541	1,564	1,502
215446_s_at	**LOX**	lysyl oxidase	0,0410371	1,315	0,0443242	2,752	2,033
212031_at	**RBM25**	RNA binding motif protein 25	0,0410955	1,431	0,0246440	1,448	1,440
209278_s_at	**TFPI2**	tissue factor pathway inhibitor 2	0,0412279	1,749	0,0326158	6,625	4,187
222748_s_at	**TXNL4B**	thioredoxin-like 4B	0,0412672	1,471	0,0148603	1,801	1,636
200004_at	**EIF4G2**	eukaryotic translation initiation factor 4 gamma, 2	0,0414707	1,083	0,0274382	1,138	1,111

211429_s_at	**SERPINA1**	serpin peptidase inhibitor, clade A (alpha-1 antiproteinase, antitrypsin), member 1	0,0416410	2,411	0,0468544	3,377	2,894
240231_at	**AZIN1**	antizyme inhibitor 1	0,0417082	1,180	0,0309596	1,327	1,254
225022_at	**DCBLD1**	discoidin, CUB and LCCL domain containing 1	0,0418846	1,325	0,0230671	1,352	1,339
242138_at	**DLX1**	distal-less homeo box 1	0,0419413	1,671	0,0276971	1,711	1,691
223172_s_at	**MTP18**	NA	0,0420364	1,369	0,0461567	1,496	1,432
203821_at	**HBEGF**	heparin-binding EGF-like growth factor	0,0420946	2,323	0,0234990	2,100	2,211
238611_at	**HNRPM**	heterogeneous nuclear ribonucleoprotein M	0,0420946	1,136	0,0230733	1,632	1,384
215493_x_at	**BTN2A1**	butyrophilin, subfamily 2, member A1	0,0421570	1,185	0,0346347	1,155	1,170
201582_at	**SEC23B**	Sec23 homolog B (S. cerevisiae)	0,0422253	1,642	0,0209106	1,853	1,748
244803_at	**YY1AP1**	YY1 associated protein 1	0,0422694	1,345	0,0060702	1,356	1,350
44563_at	**WDR79**	WD repeat domain 79	0,0423056	1,170	0,0251250	1,246	1,208
1557238_s_at	**FLJ10707**	NA	0,0425977	1,474	0,0321504	1,510	1,492
239096_at	**C14orf32**	chromosome 14 open reading frame 32	0,0427494	1,323	0,0417119	1,167	1,245
202703_at	**DUSP11**	dual specificity phosphatase 11 (RNA/RNP complex 1-interacting)	0,0428052	1,169	0,0394642	1,176	1,172
202565_s_at	**SVIL**	supervillin	0,0429094	1,727	0,0090508	1,697	1,712
228837_at	**TCF4**	transcription factor 4	0,0429734	1,364	0,0327969	1,320	1,342
234880_x_at	**KRTAP1-3**	keratin associated protein 1-3	0,0429888	1,604	0,0331902	2,124	1,864
204256_at	**ELOVL6**	ELOVL family member 6, elongation of long chain fatty acids (FEN1/Elo2, SUR4/Elo3-like, yeast)	0,0431130	1,513	0,0204257	1,702	1,607
211582_x_at	**LST1**	leukocyte specific transcript 1	0,0432095	1,972	0,0465743	1,862	1,917
242172_at	**MEIS1**	Meis1, myeloid ecotropic viral integration site 1 homolog (mouse)	0,0432095	1,241	0,0455042	1,286	1,264
206335_at	**GALNS**	galactosamine (N-acetyl)-6-sulfate sulfatase (Morquio syndrome, mucopolysaccharidosis type IVA)	0,0432434	1,222	0,0404117	1,187	1,204
218313_s_at	**GALNT7**	UDP-N-acetyl-alpha-D-galactosamine:polypeptide N-acetylgalactosaminyltransferase 7 (GalNAc-T7)	0,0432891	1,356	0,0414287	1,459	1,407
244414_at	**MAML2**	mastermind-like 2 (Drosophila)	0,0433494	2,720	0,0283675	1,724	2,222
225772_s_at	**MGC14288**	NA	0,0000000	0,649	0,0000000	0,728	0,689
226393_at	**CYP2U1**	cytochrome P450, family 2, subfamily U, polypeptide 1	0,0001069	0,520	0,0230671	0,518	0,519
202475_at	**NIFIE14**	NA	0,0001069	0,696	0,0443069	0,737	0,716
231851_at	**RAVER2**	NA	0,0001069	0,304	0,0006180	0,464	0,384
207983_s_at	**STAG2**	stromal antigen 2	0,0001069	0,647	0,0425645	0,843	0,745
218268_at	**TBC1D15**	TBC1 domain family, member 15	0,0001069	0,736	0,0249775	0,842	0,789
224806_at	**TRIM25**	tripartite motif-containing 25	0,0001069	0,642	0,0137391	0,774	0,708
222514_at	**RRAGC**	Ras-related GTP binding C	0,0001735	0,521	0,0148603	0,440	0,481
224640_at	**SPPL3**	NA	0,0001735	0,819	0,0222452	0,790	0,805
209655_s_at	**TMEM47**	transmembrane protein 47	0,0001735	0,468	0,0024251	0,574	0,521
203786_s_at	**TPD52L1**	tumor protein D52-like 1	0,0001735	0,403	0,0048235	0,376	0,390
221622_s_at	**HT007**	NA	0,0002565	0,604	0,0573855	0,768	0,686
218631_at	**AVPI1**	arginine vasopressin-induced 1	0,0003151	0,374	0,0312490	0,353	0,363
209275_s_at	**CLN3**	ceroid-lipofuscinosis, neuronal 3, juvenile (Batten, Spielmeyer-Vogt disease)	0,0003151	0,560	0,0002773	0,512	0,536
204925_at	**CTNS**	cystinosis, nephropathic	0,0003151	0,546	0,0005796	0,511	0,529
221519_at	**FBXW4**	F-box and WD-40 domain protein 4	0,0003151	0,800	0,0034060	0,782	0,791
225930_at	**NKIRAS1**	NFKB inhibitor interacting Ras-like 1	0,0003151	0,561	0,0006333	0,619	0,590
231161_x_at	**TRIM8**	tripartite motif-containing 8	0,0003151	0,789	0,0545438	0,846	0,817
218606_at	**ZDHHC7**	zinc finger, DHHC-type containing 7	0,0003151	0,635	0,0000000	0,638	0,636
201985_at	**KIAA0196**	KIAA0196	0,0003174	0,654	0,0218822	0,783	0,719
218191_s_at	**LMBRD1**	LMBR1 domain containing 1	0,0003174	0,696	0,0341417	0,673	0,685
217548_at	**MGC61550**	NA	0,0003174	0,674	0,0039168	0,619	0,646
225900_at	**SEC15L2**	SEC15-like 2 (S. cerevisiae)	0,0003174	0,631	0,0007133	0,574	0,602
226277_at	**COL4A3BP**	collagen, type IV, alpha 3 (Goodpasture antigen) binding protein	0,0003372	0,598	0,0171063	0,400	0,499
37943_at	**ZFYVE26**	zinc finger, FYVE domain containing 26	0,0003372	0,613	0,0164663	0,653	0,633
224447_s_at	**C17orf37**	chromosome 17 open reading frame 37	0,0003690	0,705	0,0027369	0,775	0,740
221692_s_at	**MRPL34**	mitochondrial ribosomal protein L34	0,0003690	0,584	0,0051672	0,656	0,620
209127_s_at	**SART3**	squamous cell carcinoma antigen recognised by T cells 3	0,0004072	0,595	0,0140986	0,761	0,678
226243_at	**LOC391356**	NA	0,0004141	0,701	0,0433668	0,851	0,776
204488_at	**TMEM15**	transmembrane protein 15	0,0004774	0,709	0,0004401	0,716	0,713
1552610_a_at	**JAK1**	Janus kinase 1 (a protein tyrosine kinase)	0,0005263	0,568	0,0280031	0,872	0,720
232008_s_at	**BBX**	bobby sox homolog (Drosophila)	0,0005554	0,731	0,0355707	0,600	0,665
212831_at	**EGFL5**	EGF-like-domain, multiple 5	0,0005554	0,711	0,0053701	0,686	0,698
215913_s_at	**GULP1**	GULP, engulfment adaptor PTB domain containing 1	0,0005554	0,490	0,0575751	0,404	0,447
201779_s_at	**RNF13**	ring finger protein 13	0,0005554	0,642	0,0015437	0,722	0,682
222469_s_at	**TOLLIP**	toll interacting protein	0,0005803	0,733	0,0000000	0,645	0,689
201527_at	**ATP6V1F**	ATPase, H+ transporting, lysosomal 14kDa, V1 subunit F	0,0006193	0,756	0,0008022	0,792	0,774

210139_s_at	**PMP22**	peripheral myelin protein 22	0,0006193	0,682	0,0241340	0,617	0,650
211337_s_at	**TUBGCP4**	NA	0,0006193	0,694	0,0417119	0,834	0,764
208626_s_at	**VAT1**	vesicle amine transport protein 1 homolog (T californica)	0,0006193	0,589	0,0194107	0,492	0,541
214946_x_at	**LOC387680**	NA	0,0006682	0,644	0,0017518	0,658	0,651
202844_s_at	**RALBP1**	ralA binding protein 1	0,0006682	0,670	0,0083971	0,769	0,720
218032_at	**SNN**	stannin	0,0006682	0,564	0,0034050	0,524	0,544
217852_s_at	**ARL10C**	ADP-ribosylation factor-like 10C	0,0006979	0,751	0,0125573	0,825	0,788
217827_s_at	**SPG21**	spastic paraplegia 21 (autosomal recessive, Mast syndrome)	0,0006979	0,653	0,0002160	0,672	0,662
237202_at	**PGPEP1**	pyroglutamyl-peptidase I	0,0007183	0,762	0,0009353	0,775	0,769
203803_at	**PCYOX1**	prenylcysteine oxidase 1	0,0007384	0,503	0,0198526	0,488	0,495
216903_s_at	**CBARA1**	calcium binding atopy-related autoantigen 1	0,0007419	0,645	0,0012162	0,652	0,649
224609_at	**SLC44A2**	solute carrier family 44, member 2	0,0007419	0,586	0,0206042	0,528	0,557
203635_at	**DSCR3**	Down syndrome critical region gene 3	0,0008730	0,748	0,0069118	0,798	0,773
200596_s_at	**EIF3S10**	eukaryotic translation initiation factor 3, subunit 10 theta, 150/170kDa	0,0008730	0,713	0,0406458	0,871	0,792
218167_at	**AMZ2**	NA	0,0009209	0,709	0,0168913	0,800	0,755
229526_at	**AQP11**	aquaporin 11	0,0009209	0,554	0,0012157	0,482	0,518
202382_s_at	**GNPDA1**	glucosamine-6-phosphate deaminase 1	0,0009209	0,405	0,0017727	0,470	0,438
207006_s_at	**HSU79303**	NA	0,0009209	0,745	0,0002614	0,672	0,709
208926_at	**NEU1**	sialidase 1 (lysosomal sialidase)	0,0009209	0,447	0,0002773	0,465	0,456
211941_s_at	**PBP**	prostatic binding protein	0,0009209	0,665	0,0148603	0,550	0,607
202773_s_at	**SFRS8**	splicing factor, arginine/serine-rich 8 (suppressor-of-white-apricot homolog, Drosophila)	0,0009209	0,647	0,0214208	0,750	0,699
223396_at	**TMEM60**	transmembrane protein 60	0,0009209	0,647	0,0026882	0,711	0,679
209088_s_at	**UBN1**	ubinuclein 1	0,0009209	0,642	0,0177143	0,822	0,732
226851_at	**LYPLAL1**	lysophospholipase-like 1	0,0009846	0,614	0,0070289	0,623	0,618
226101_at	**PRKCE**	protein kinase C, epsilon	0,0010267	0,577	0,0197671	0,659	0,618
223356_s_at	**MTIF3**	NA	0,0010394	0,677	0,0080990	0,846	0,762
217988_at	**CCNB1IP1**	cyclin B1 interacting protein 1	0,0011340	0,514	0,0014887	0,617	0,565
203941_at	**RC74**	NA	0,0011350	0,780	0,0281764	0,749	0,764
225862_at	**SLC25A26**	solute carrier family 25, member 26	0,0011793	0,790	0,0529693	0,843	0,817
212724_at	**RND3**	Rho family GTPase 3	0,0012355	0,628	0,0014814	0,664	0,646
215189_at	**KRTHB6**	keratin, hair, basic, 6 (monilethrix)	0,0012827	0,503	0,0056803	0,485	0,494
224995_at	**SPIRE1**	spire homolog 1 (Drosophila)	0,0013736	0,672	0,0223115	0,678	0,675
229323_at	**LOC387723**	NA	0,0014677	0,665	0,0361705	0,782	0,723
203608_at	**ALDH5A1**	aldehyde dehydrogenase 5 family, member A1 (succinate-semialdehyde dehydrogenase)	0,0015006	0,612	0,0259904	0,483	0,547
218450_at	**HEBP1**	heme binding protein 1	0,0015006	0,638	0,0204257	0,593	0,615
202562_s_at	**C14orf1**	chromosome 14 open reading frame 1	0,0015046	0,591	0,0000313	0,519	0,555
201522_x_at	**SNRPN**	small nuclear ribonucleoprotein polypeptide N	0,0015046	0,675	0,0286647	0,732	0,704
200850_s_at	**AHCYL1**	S-adenosylhomocysteine hydrolase-like 1	0,0015836	0,805	0,0025745	0,654	0,729
218072_at	**COMMD9**	COMM domain containing 9	0,0015859	0,737	0,0298361	0,893	0,815
209750_at	**NR1D2**	nuclear receptor subfamily 1, group D, member 2	0,0015859	0,440	0,0189777	0,729	0,584
220945_x_at	**MANSC1**	MANSC domain containing 1	0,0016034	0,623	0,0052829	0,672	0,648
213414_s_at	**RPS19**	ribosomal protein S19	0,0016257	0,894	0,0233467	0,700	0,797
239147_at	**DKFZp313G1735**	NA	0,0016930	0,436	0,0204257	0,364	0,400
202419_at	**FVT1**	follicular lymphoma variant translocation 1	0,0017146	0,760	0,0003910	0,666	0,713
218514_at	**FLJ10587**	NA	0,0017230	0,788	0,0273814	0,830	0,809
206847_s_at	**HOXA7**	homeo box A7	0,0017753	0,609	0,0127867	0,565	0,587
225593_at	**LSM10**	LSM10, U7 small nuclear RNA associated	0,0017856	0,696	0,0072198	0,777	0,737
225351_at	**FAM45A**	family with sequence similarity 45, member A	0,0018060	0,631	0,0040368	0,569	0,600
220755_s_at	**C6orf48**	chromosome 6 open reading frame 48	0,0018553	0,591	0,0117442	0,600	0,596
208647_at	**FDFT1**	farnesyl-diphosphate farnesyltransferase 1	0,0018896	0,550	0,0009451	0,564	0,557
212217_at	**SLC3A1**	solute carrier family 3 (cystine, dibasic and neutral amino acid transporters, activator of cystine, dibasic and neutral amino acid transport), member 1	0,0019001	0,674	0,0218822	0,789	0,731
210094_s_at	**PARD3**	par-3 partitioning defective 3 homolog (C. elegans)	0,0019053	0,744	0,0181458	0,850	0,797
235618_at	**ZNF507**	zinc finger protein 507	0,0019246	0,770	0,0148519	0,824	0,797
202246_s_at	**CDK4**	cyclin-dependent kinase 4	0,0019407	0,633	0,0042864	0,644	0,638
206584_at	**LY96**	lymphocyte antigen 96	0,0019407	0,592	0,0020442	0,709	0,650
212717_at	**PLEKHM1**	pleckstrin homology domain containing, family M (with RUN domain) member 1	0,0019407	0,701	0,0006545	0,726	0,713
227770_at	**VPS4A**	vacuolar protein sorting 4A (yeast)	0,0019407	0,754	0,0019459	0,770	0,762
212305_s_at	**KIAA0268**	NA	0,0019471	0,778	0,0574299	0,805	0,792
209553_at	**KIAA0804**	KIAA0804	0,0020538	0,744	0,0039891	0,788	0,766
203550_s_at	**C1orf2**	chromosome 1 open reading frame 2	0,0020697	0,793	0,0592984	0,724	0,758
217286_s_at	**NDRG3**	NDRG family member 3	0,0020697	0,647	0,0036068	0,724	0,685
227481_at	**CNKSR3**	CNKSR family member 3	0,0020896	0,417	0,0197769	0,527	0,472
220329_s_at	**C6orf96**	chromosome 6 open reading frame 96	0,0021012	0,606	0,0094815	0,693	0,650
214368_at	**RASGRP2**	RAS guanyl releasing protein 2 (calcium and DAG-regulated)	0,0021353	0,748	0,0535120	0,816	0,782

220041_at	**SMP3**	NA	0,0021778	0,583	0,0010781	0,554	0,569
208847_s_at	**ADH5**	alcohol dehydrogenase 5 (class III), chi polypeptide	0,0021811	0,718	0,0404117	0,769	0,744
213249_at	**FBXL7**	F-box and leucine-rich repeat protein 7	0,0021843	0,565	0,0031478	0,584	0,574
201754_at	**COX6C**	cytochrome c oxidase subunit VIc	0,0022103	0,804	0,0218176	0,876	0,840
223066_at	**SNAPAP**	SNAP-associated protein	0,0022103	0,773	0,0075085	0,801	0,787
213246_at	**C14orf109**	chromosome 14 open reading frame 109	0,0022520	0,640	0,0241006	0,755	0,697
206090_s_at	**DISC1**	disrupted in schizophrenia 1	0,0023165	0,422	0,0012744	0,475	0,448
239053_at	**WDR39**	WD repeat domain 39	0,0023305	0,775	0,0081005	0,781	0,778
205731_s_at	**NCOA2**	nuclear receptor coactivator 2	0,0023557	0,706	0,0301096	0,854	0,780
227276_at	**PLXDC2**	plexin domain containing 2	0,0023765	0,564	0,0293233	0,496	0,530
1554093_a_at	**SNAPC5**	small nuclear RNA activating complex, polypeptide 5, 19kDa	0,0023765	0,678	0,0213898	0,713	0,695
225009_at	**CKLFSF4**	chemokine-like factor superfamily 4	0,0023944	0,612	0,0288092	0,728	0,670
202378_s_at	**LEPROT**	leptin receptor overlapping transcript	0,0023944	0,713	0,0251425	0,812	0,762
224948_at	**MRPS24**	mitochondrial ribosomal protein S24	0,0023944	0,778	0,0042803	0,805	0,792
203544_s_at	**STAM**	signal transducing adaptor molecule (SH3 domain and ITAM motif) 1	0,0023966	0,724	0,0191985	0,747	0,736
222430_s_at	**YTHDF2**	YTH domain family, member 2	0,0024141	0,791	0,0483058	0,707	0,749
218519_at	**SLC35A5**	solute carrier family 35, member A5	0,0024789	0,701	0,0269008	0,831	0,766
217759_at	**TRIM44**	tripartite motif-containing 44	0,0024852	0,798	0,0443071	0,871	0,834
226638_at	**ARHGAP23**	Rho GTPase activating protein 23	0,0025083	0,664	0,0004401	0,583	0,623
209329_x_at	**HIGD2A**	HIG1 domain family, member 2A	0,0025083	0,743	0,0025196	0,821	0,782
210251_s_at	**RIPX**	NA	0,0025238	0,492	0,0293486	0,663	0,577
223478_at	**TIMM8B**	translocase of inner mitochondrial membrane 8 homolog B (yeast)	0,0025238	0,561	0,0382348	0,637	0,599
201536_at	**DUSP3**	dual specificity phosphatase 3 (vaccinia virus phosphatase VH1-related)	0,0025418	0,639	0,0300238	0,659	0,649
202322_s_at	**GGPS1**	geranylgeranyl diphosphate synthase 1	0,0025510	0,717	0,0334206	0,847	0,782
203068_at	**KLHL21**	kelch-like 21 (Drosophila)	0,0025872	0,641	0,0350632	0,783	0,712
221524_s_at	**RRAGD**	Ras-related GTP binding D	0,0026239	0,468	0,0098933	0,407	0,438
230186_at	**MGC17839**	NA	0,0026462	0,759	0,0455501	0,785	0,772
219641_at	**DET1**	de-etiolated homolog 1 (Arabidopsis)	0,0026466	0,765	0,0031434	0,760	0,763
223443_s_at	**FLJ32065**	NA	0,0026466	0,505	0,0311063	0,726	0,615
225525_at	**KIAA1671**	NA	0,0026466	0,705	0,0075743	0,669	0,687
222212_s_at	**LASS2**	LAG1 longevity assurance homolog 2 (S. cerevisiae)	0,0026466	0,751	0,0022170	0,728	0,739
203247_s_at	**ZNF24**	zinc finger protein 24 (KOX 17)	0,0026466	0,657	0,0256267	0,842	0,749
229213_at	**DIRC2**	disrupted in renal carcinoma 2	0,0026861	0,819	0,0119968	0,760	0,790
204266_s_at	**CHKA**	choline kinase alpha	0,0026867	0,728	0,0265466	0,706	0,717
214830_at	**SLC38A6**	solute carrier family 38, member 6	0,0026867	0,497	0,0028617	0,497	0,497
227430_at	**ZC3H10**	zinc finger CCCH-type containing 10	0,0026867	0,734	0,0099975	0,731	0,732
221706_s_at	**MDS032**	NA	0,0026886	0,780	0,0583527	0,797	0,789
218785_s_at	**RABL5**	RAB, member RAS oncogene family-like 5	0,0027564	0,757	0,0549150	0,725	0,741
212624_s_at	**CHN1**	chimerin (chimaerin) 1	0,0027586	0,410	0,0518398	0,502	0,456
55692_at	**ELMO2**	engulfment and cell motility 2 (ced-12 homolog, C. elegans)	0,0027586	0,773	0,0000517	0,745	0,759
223253_at	**EPDR1**	ependymin related protein 1 (zebrafish)	0,0027586	0,570	0,0265811	0,642	0,606
202024_at	**ASNA1**	arsA arsenite transporter, ATP-binding, homolog 1 (bacterial)	0,0027712	0,725	0,0400482	0,844	0,784
214150_x_at	**ATP6V0E**	ATPase, H+ transporting, lysosomal 9kDa, V0 subunit e	0,0027712	0,855	0,0325509	0,611	0,733
203181_x_at	**SRPK2**	SFRS protein kinase 2	0,0027712	0,776	0,0157790	0,781	0,778
201193_at	**IDH1**	isocitrate dehydrogenase 1 (NADP+), soluble	0,0028223	0,448	0,0000517	0,442	0,445
212101_at	**KPNA6**	karyopherin alpha 6 (importin alpha 7)	0,0028407	0,728	0,0044091	0,764	0,746
231059_x_at	**SCAND1**	SCAN domain containing 1	0,0028416	0,747	0,0018035	0,741	0,744
200761_s_at	**ARL6IP5**	ADP-ribosylation-like factor 6 interacting protein 5	0,0028733	0,671	0,0256751	0,734	0,703
1553987_at	**FLJ39616**	NA	0,0029103	0,623	0,0064363	0,611	0,617
218023_s_at	**FAM53C**	family with sequence similarity 53, member C	0,0029110	0,834	0,0002160	0,757	0,796
238444_at	**ZNF618**	zinc finger protein 618	0,0029110	0,597	0,0270560	0,683	0,640
200789_at	**ECH1**	enoyl Coenzyme A hydratase 1, peroxisomal	0,0029156	0,710	0,0288245	0,799	0,755
230106_at	**ZXDC**	ZXD family zinc finger C	0,0029156	0,732	0,0219329	0,789	0,760
242935_at	**SBF2**	SET binding factor 2	0,0029286	0,660	0,0154934	0,746	0,703
224337_s_at	**FZD4**	frizzled homolog 4 (Drosophila)	0,0029594	0,633	0,0019934	0,638	0,636
225052_at	**MGC14327**	NA	0,0029594	0,795	0,0123336	0,865	0,830
212660_at	**PHF15**	PHD finger protein 15	0,0029594	0,614	0,0119039	0,788	0,701
209833_at	**CRADD**	CASP2 and RIPK1 domain containing adaptor with death domain	0,0029595	0,783	0,0477320	0,879	0,831
223248_at	**HSDL1**	hydroxysteroid dehydrogenase like 1	0,0030181	0,765	0,0086854	0,725	0,745
208158_s_at	**OSBPL1A**	oxysterol binding protein-like 1A	0,0030181	0,533	0,0238670	0,589	0,561
217722_s_at	**NGRN**	neugrin, neurite outgrowth associated	0,0030469	0,739	0,0308931	0,825	0,782
232412_at	**FBXL20**	F-box and leucine-rich repeat protein 20	0,0030509	0,402	0,0006629	0,494	0,448
223445_at	**DTNBP1**	dystrobrevin binding protein 1	0,0031193	0,743	0,0175274	0,801	0,772
228073_at	**HDHD4**	haloacid dehalogenase-like hydrolase domain containing 4	0,0031193	0,720	0,0136584	0,842	0,781
213161_at	**TMOD1**	tropomodulin 1	0,0031193	0,736	0,0144847	0,783	0,759
235643_at	**SAMD9L**	sterile alpha motif domain containing 9-like	0,0031472	0,563	0,0090459	0,646	0,604

201790_s_at	**DHCR7**	7-dehydrocholesterol reductase	0,0032043	0,397	0,0000313	0,311	0,354
202218_s_at	**FADS2**	fatty acid desaturase 2	0,0032043	0,399	0,0002614	0,401	0,400
209230_s_at	**P8**	NA	0,0032043	0,564	0,0221068	0,617	0,591
224836_at	**TP53INP2**	tumor protein p53 inducible nuclear protein 2	0,0032043	0,550	0,0000000	0,517	0,533
222908_at	**FAM38B**	family with sequence similarity 38, member B	0,0032209	0,347	0,0042859	0,327	0,337
203897_at	**LOC57149**	NA	0,0032209	0,720	0,0225056	0,775	0,748
1553158_at	**MGC14126**	NA	0,0032209	0,616	0,0060209	0,581	0,599
222351_at	**PPP2R1B**	protein phosphatase 2 (formerly 2A), regulatory subunit A (PR 65), beta isoform	0,0032209	0,528	0,0067407	0,700	0,614
227151_at	**SH3PX3**	SH3 and PX domain containing 3	0,0032209	0,723	0,0000313	0,612	0,667
226679_at	**SLC26A11**	solute carrier family 26, member 11	0,0032395	0,761	0,0000517	0,714	0,738
218583_s_at	**RP42**	NA	0,0032425	0,800	0,0419524	0,792	0,796
226425_at	**RSNL2**	restin-like 2	0,0032425	0,624	0,0424384	0,754	0,689
223009_at	**FLJ20625**	NA	0,0032616	0,727	0,0000000	0,689	0,708
201275_at	**FDPS**	farnesyl diphosphate synthase (farnesyl pyrophosphate synthetase, dimethylallyltranstransferase, geranyltranstransferase)	0,0032792	0,579	0,0432516	0,678	0,628
202114_at	**SNX2**	sorting nexin 2	0,0032792	0,647	0,0112699	0,733	0,690
227482_at	**ADCK1**	aarF domain containing kinase 1	0,0033205	0,719	0,0232089	0,756	0,737
226657_at	**DKFZp762H185**	NA	0,0033299	0,455	0,0004401	0,346	0,401
224688_at	**FLJ10099**	NA	0,0034293	0,793	0,0263483	0,814	0,804
211423_s_at	**SC5DL**	sterol-C5-desaturase (ERG3 delta-5-desaturase homolog, fungal)-like	0,0034293	0,604	0,0134808	0,605	0,604
219460_s_at	**FLJ20507**	NA	0,0034590	0,762	0,0004401	0,723	0,742
1560021_at	**RPS20**	ribosomal protein S20	0,0034590	0,736	0,0252405	0,693	0,714
225653_at	**TGFBRAP1**	transforming growth factor, beta receptor associated protein 1	0,0034590	0,772	0,0066557	0,665	0,719
229060_at	**YPEL2**	yippee-like 2 (Drosophila)	0,0034590	0,638	0,0015269	0,652	0,645
229392_s_at	**PIK3R2**	phosphoinositide-3-kinase, regulatory subunit 2 (p85 beta)	0,0034606	0,855	0,0320467	0,780	0,818
213729_at	**FNBP3**	formin binding protein 3	0,0035554	0,679	0,0601976	0,875	0,777
212526_at	**SPG20**	spastic paraplegia 20, spartin (Troyer syndrome)	0,0035554	0,646	0,0238523	0,750	0,698
203665_at	**HMOX1**	heme oxygenase (decycling) 1	0,0036029	0,554	0,0129483	0,530	0,542
218190_s_at	**UCRC**	NA	0,0036601	0,855	0,0014814	0,663	0,759
210817_s_at	**NDP52**	NA	0,0036655	0,802	0,0405844	0,810	0,806
208474_at	**CLDN6**	claudin 6	0,0036774	0,804	0,0477825	0,826	0,815
217173_s_at	**LDLR**	low density lipoprotein receptor (familial hypercholesterolemia)	0,0036774	0,433	0,0046091	0,469	0,451
218550_s_at	**LRRC20**	leucine rich repeat containing 20	0,0036774	0,616	0,0305418	0,635	0,625
226798_at	**BCL2L13**	BCL2-like 13 (apoptosis facilitator)	0,0036800	0,699	0,0261162	0,697	0,698
217764_s_at	**RAB31**	RAB31, member RAS oncogene family	0,0037216	0,692	0,0206042	0,645	0,669
203588_s_at	**TFDP2**	transcription factor Dp-2 (E2F dimerization partner 2)	0,0037242	0,730	0,0373926	0,844	0,787
203292_s_at	**VPS11**	vacuolar protein sorting 11 (yeast)	0,0037268	0,717	0,0002614	0,725	0,721
225029_at	**LOC550643**	NA	0,0037768	0,829	0,0174388	0,856	0,843
229465_s_at	**PTPRS**	protein tyrosine phosphatase, receptor type, S	0,0037768	0,617	0,0158770	0,754	0,686
226929_at	**MTHFR**	5,10-methylenetetrahydrofolate reductase (NADPH)	0,0038372	0,677	0,0005796	0,548	0,613
203076_s_at	**SMAD2**	SMAD, mothers against DPP homolog 2 (Drosophila)	0,0038372	0,821	0,0404417	0,873	0,847
230421_at	**DKFZp686E2433**	NA	0,0038620	0,649	0,0015354	0,610	0,629
218548_x_at	**TEX264**	testis expressed sequence 264	0,0038807	0,774	0,0000000	0,690	0,732
1555058_a_at	**LPGAT1**	lysophosphatidylglycerol acyltransferase 1	0,0039097	0,635	0,0039656	0,682	0,658
228184_at	**DISP1**	dispatched homolog 1 (Drosophila)	0,0039339	0,718	0,0084378	0,689	0,704
1553974_at	**LOC128977**	NA	0,0039493	0,650	0,0163986	0,751	0,700
209224_s_at	**NDUFA2**	NADH dehydrogenase (ubiquinone) 1 alpha subcomplex, 2, 8kDa	0,0039515	0,763	0,0023942	0,695	0,729
235030_at	**FAM55C**	family with sequence similarity 55, member C	0,0040316	0,381	0,0400472	0,521	0,451
228951_at	**FLJ10815**	NA	0,0040487	0,752	0,0177344	0,738	0,745
223808_s_at	**PTPMT1**	protein tyrosine phosphatase, mitochondrial 1	0,0040487	0,747	0,0182843	0,782	0,765
220864_s_at	**NDUFA13**	NADH dehydrogenase (ubiquinone) 1 alpha subcomplex, 13	0,0040864	0,804	0,0066448	0,848	0,826
211594_s_at	**MRPL9**	mitochondrial ribosomal protein L9	0,0040921	0,808	0,0373345	0,871	0,840
222672_at	**C6orf149**	chromosome 6 open reading frame 149	0,0040981	0,690	0,0246987	0,756	0,723
212332_at	**RBL2**	retinoblastoma-like 2 (p130)	0,0041273	0,572	0,0069157	0,804	0,688
200878_at	**EPAS1**	endothelial PAS domain protein 1	0,0041558	0,627	0,0118108	0,696	0,662
212929_s_at	**FAM21C**	family with sequence similarity 21, member C	0,0041740	0,709	0,0352772	0,711	0,710
242561_at	**IPO9**	importin 9	0,0041890	0,760	0,0440580	0,845	0,803
212625_at	**STX10**	syntaxin 10	0,0041890	0,759	0,0014456	0,785	0,772
225729_at	**C6orf89**	chromosome 6 open reading frame 89	0,0042131	0,745	0,0019981	0,662	0,703
202263_at	**CYB5R1**	cytochrome b5 reductase 1	0,0042591	0,724	0,0005731	0,612	0,668
201076_at	**NHP2L1**	NHP2 non-histone chromosome protein 2-like 1 (S. cerevisiae)	0,0043019	0,761	0,0392819	0,885	0,823
235431_s_at	**PELI3**	pellino homolog 3 (Drosophila)	0,0043054	0,723	0,0002160	0,689	0,706

235765_at	**TLE4**	transducin-like enhancer of split 4 (E(sp1) homolog, Drosophila)	0,0043088	0,704	0,0038582	0,655	0,679
228183_s_at	**RIP**	NA	0,0043105	0,660	0,0136758	0,696	0,678
218694_at	**ARMCX1**	armadillo repeat containing, X-linked 1	0,0043223	0,677	0,0435599	0,800	0,739
235713_at	**LOC91801**	NA	0,0043710	0,694	0,0379701	0,819	0,757
201398_s_at	**TRAM1**	translocation associated membrane protein 1	0,0043710	0,791	0,0041789	0,768	0,779
220261_s_at	**ZDHHC4**	zinc finger, DHHC-type containing 4	0,0043726	0,703	0,0024271	0,629	0,666
223060_at	**C14orf119**	chromosome 14 open reading frame 119	0,0043882	0,774	0,0581975	0,902	0,838
210774_s_at	**NCOA4**	nuclear receptor coactivator 4	0,0043882	0,752	0,0519148	0,854	0,803
225882_at	**SLC35B4**	solute carrier family 35, member B4	0,0044214	0,714	0,0079970	0,838	0,776
1557137_at	**TMEM17**	transmembrane protein 17	0,0044422	0,561	0,0002711	0,566	0,564
225564_at	**SPATA13**	spermatogenesis associated 13	0,0045115	0,554	0,0331895	0,797	0,675
226763_at	**SESTD1**	SEC14 and spectrin domains 1	0,0045913	0,518	0,0021104	0,539	0,528
223376_s_at	**BRI3**	brain protein I3	0,0046100	0,682	0,0001227	0,672	0,677
202326_at	**EHMT2**	euchromatic histone-lysine N-methyltransferase 2	0,0046272	0,691	0,0208800	0,796	0,743
210007_s_at	**GPD2**	glycerol-3-phosphate dehydrogenase 2 (mitochondrial)	0,0046793	0,593	0,0577081	0,794	0,693
204981_at	**SLC22A18**	solute carrier family 22 (organic cation transporter), member 18	0,0047094	0,642	0,0277277	0,618	0,630
205917_at	**ZNF264**	zinc finger protein 264	0,0047146	0,714	0,0161187	0,765	0,739
202201_at	**BLVRB**	biliverdin reductase B (flavin reductase (NADPH))	0,0048846	0,521	0,0045354	0,570	0,545
209490_s_at	**PPT2**	palmitoyl-protein thioesterase 2	0,0049088	0,786	0,0287838	0,822	0,804
213272_s_at	**LOC57146**	NA	0,0049385	0,487	0,0043096	0,566	0,527
219757_s_at	**C14orf101**	chromosome 14 open reading frame 101	0,0049515	0,610	0,0083484	0,690	0,650
201781_s_at	**AIP**	aryl hydrocarbon receptor interacting protein	0,0049967	0,765	0,0011564	0,782	0,774
223388_s_at	**ZFYVE1**	zinc finger, FYVE domain containing 1	0,0050123	0,680	0,0274738	0,725	0,702
239035_at	**C1orf167**	chromosome 1 open reading frame 167	0,0050446	0,622	0,0058575	0,635	0,629
227222_at	**FBXO10**	F-box protein 10	0,0050455	0,705	0,0274285	0,770	0,738
204565_at	**THEM2**	thioesterase superfamily member 2	0,0050939	0,623	0,0193138	0,665	0,644
203884_s_at	**RAB11FIP2**	RAB11 family interacting protein 2 (class I)	0,0051516	0,591	0,0500836	0,637	0,614
225870_s_at	**TRAPPC5**	trafficking protein particle complex 5	0,0051713	0,712	0,0441531	0,848	0,780
202125_s_at	**ALS2CR3**	amyotrophic lateral sclerosis 2 (juvenile) chromosome region, candidate 3	0,0051862	0,750	0,0528682	0,775	0,763
228768_at	**KIAA1961**	NA	0,0052094	0,724	0,0341038	0,609	0,667
223227_at	**BBS2**	Bardet-Biedl syndrome 2	0,0052490	0,650	0,0155863	0,701	0,676
221142_s_at	**PECR**	peroxisomal trans-2-enoyl-CoA reductase	0,0052883	0,664	0,0534086	0,824	0,744
201751_at	**KIAA0063**	NA	0,0053527	0,745	0,0041324	0,747	0,746
205452_at	**PIGB**	phosphatidylinositol glycan, class B	0,0053527	0,726	0,0083358	0,637	0,681
203241_at	**UVRAG**	UV radiation resistance associated gene	0,0053527	0,682	0,0321664	0,897	0,790
235990_at	**RAD9A**	RAD9 homolog A (S. pombe)	0,0053543	0,653	0,0017396	0,676	0,665
235819_at	**BTF3L4**	basic transcription factor 3-like 4	0,0053560	0,652	0,0109442	0,733	0,693
205134_s_at	**NUFIP1**	nuclear fragile X mental retardation protein interacting protein 1	0,0053560	0,713	0,0107935	0,765	0,739
201568_at	**QP-C**	NA	0,0053617	0,805	0,0535593	0,905	0,855
229803_s_at	**NUDT3**	nudix (nucleoside diphosphate linked moiety X)-type motif 3	0,0054791	0,652	0,0188412	0,687	0,670
214749_s_at	**ARMCX6**	armadillo repeat containing, X-linked 6	0,0054817	0,709	0,0319002	0,814	0,761
207971_s_at	**KIAA0582**	KIAA0582	0,0054923	0,689	0,0397478	0,690	0,690
207081_s_at	**PIK4CA**	phosphatidylinositol 4-kinase, catalytic, alpha polypeptide	0,0055237	0,678	0,0204257	0,731	0,705
233140_s_at	**C14orf133**	chromosome 14 open reading frame 133	0,0055556	0,742	0,0237613	0,867	0,805
227759_at	**PCSK9**	proprotein convertase subtilisin/kexin type 9	0,0055682	0,687	0,0062166	0,628	0,658
212880_at	**WDR7**	WD repeat domain 7	0,0055821	0,768	0,0003661	0,753	0,760
202108_at	**PEPD**	peptidase D	0,0056080	0,696	0,0012597	0,744	0,720
203885_at	**RAB21**	RAB21, member RAS oncogene family	0,0056080	0,795	0,0460562	0,885	0,840
200029_at	**RPL19**	ribosomal protein L19	0,0056187	0,904	0,0434799	0,928	0,916
207040_s_at	**ST13**	suppression of tumorigenicity 13 (colon carcinoma) (Hsp70 interacting protein)	0,0056363	0,724	0,0322305	0,756	0,740
222522_x_at	**MRPS10**	mitochondrial ribosomal protein S10	0,0056365	0,705	0,0452487	0,813	0,759
219816_s_at	**RBM23**	RNA binding motif protein 23	0,0056721	0,795	0,0371848	0,806	0,801
201403_s_at	**MGST3**	microsomal glutathione S-transferase 3	0,0056822	0,775	0,0144865	0,879	0,827
219831_at	**CDKL3**	cyclin-dependent kinase-like 3	0,0056974	0,707	0,0059475	0,753	0,730
208774_at	**CSNK1D**	casein kinase 1, delta	0,0056974	0,681	0,0501333	0,769	0,725
226208_at	**MGC40405**	NA	0,0057029	0,728	0,0083971	0,795	0,761
224415_s_at	**HINT2**	histidine triad nucleotide binding protein 2	0,0057273	0,780	0,0196913	0,822	0,801
208786_s_at	**MAP1LC3B**	microtubule-associated protein 1 light chain 3 beta	0,0057323	0,751	0,0245904	0,822	0,786
221984_s_at	**C2orf17**	chromosome 2 open reading frame 17	0,0057379	0,829	0,0280954	0,678	0,754
1567014_s_at	**NFE2L2**	nuclear factor (erythroid-derived 2)-like 2	0,0057379	0,608	0,0526931	0,819	0,714
201681_s_at	**DLG5**	discs, large homolog 5 (Drosophila)	0,0057579	0,647	0,0223679	0,684	0,666
214101_s_at	**NPEPPS**	aminopeptidase puromycin sensitive	0,0058109	0,706	0,0056921	0,780	0,743
226239_at	**FLJ90024**	NA	0,0059016	0,756	0,0019161	0,742	0,749
200958_s_at	**SDCBP**	syndecan binding protein (syntenin)	0,0059347	0,737	0,0220406	0,821	0,779
243731_at	**SLC27A5**	solute carrier family 27 (fatty acid transporter), member 5	0,0059347	0,710	0,0041304	0,761	0,735
219124_at	**C8orf41**	chromosome 8 open reading frame 41	0,0059861	0,839	0,0318953	0,843	0,841

223950_s_at	**FLYWCH1**	FLYWCH-type zinc finger 1	0,0059954	0,742	0,0032293	0,548	0,645
206491_s_at	**NAPA**	N-ethylmaleimide-sensitive factor attachment protein, alpha	0,0059954	0,781	0,0008523	0,589	0,685
212510_at	**GPD1L**	glycerol-3-phosphate dehydrogenase 1-like	0,0061655	0,649	0,0515554	0,676	0,663
210716_s_at	**RSN**	restin (Reed-Steinberg cell-expressed intermediate filament-associated protein)	0,0061655	0,640	0,0029344	0,421	0,530
225503_at	**DHRSX**	dehydrogenase/reductase (SDR family) X-linked	0,0061876	0,704	0,0137403	0,736	0,720
212261_at	**TNRC15**	trinucleotide repeat containing 15	0,0062332	0,860	0,0226703	0,883	0,872
215071_s_at	**HIST1H2AC**	histone 1, H2ac	0,0062420	0,416	0,0022158	0,454	0,435
208717_at	**OXA1L**	oxidase (cytochrome c) assembly 1-like	0,0062458	0,654	0,0554256	0,787	0,720
239742_at	**TULP4**	tubby like protein 4	0,0062458	0,512	0,0432266	0,797	0,654
227127_at	**MGC52022**	NA	0,0062806	0,831	0,0305263	0,758	0,794
233802_at	**KIAA1276**	NA	0,0064076	0,824	0,0027369	0,766	0,795
229144_at	**KIAA1026**	NA	0,0064476	0,571	0,0111010	0,488	0,529
242761_s_at	**ZNF420**	zinc finger protein 420	0,0064950	0,678	0,0038049	0,726	0,702
205090_s_at	**NAGPA**	N-acetylglucosamine-1-phosphodiester alpha-N-acetylglucosaminidase	0,0065388	0,688	0,0008788	0,680	0,684
228411_at	**ALS2CR19**	amyotrophic lateral sclerosis 2 (juvenile) chromosome region, candidate 19	0,0065878	0,634	0,0145505	0,566	0,600
209858_x_at	**MPPE1**	metallophosphoesterase 1	0,0065878	0,732	0,0424829	0,733	0,732
225998_at	**GAB1**	GRB2-associated binding protein 1	0,0066006	0,645	0,0534928	0,788	0,716
1554667_s_at	**FLJ13984**	NA	0,0066160	0,644	0,0343878	0,723	0,684
221488_s_at	**C6orf82**	chromosome 6 open reading frame 82	0,0066454	0,764	0,0002160	0,760	0,762
202640_s_at	**RANBP3**	RAN binding protein 3	0,0066454	0,796	0,0069164	0,724	0,760
201411_s_at	**PLEKHB2**	pleckstrin homology domain containing, family B (evectins) member 2	0,0066643	0,552	0,0254919	0,751	0,651
212263_at	**QKI**	quaking homolog, KH domain RNA binding (mouse)	0,0066893	0,798	0,0279723	0,765	0,781
213889_at	**PIGL**	phosphatidylinositol glycan, class L	0,0067224	0,755	0,0448603	0,724	0,740
217354_s_at	**HPS1**	Hermansky-Pudlak syndrome 1	0,0067371	0,762	0,0200268	0,593	0,678
217864_s_at	**PIAS1**	protein inhibitor of activated STAT, 1	0,0069371	0,789	0,0497975	0,816	0,802
213707_s_at	**DLX5**	distal-less homeo box 5	0,0069894	0,538	0,0371176	0,404	0,471
212573_at	**KIAA0830**	NA	0,0069894	0,567	0,0119012	0,547	0,557
208024_s_at	**DGCR6**	DiGeorge syndrome critical region gene 6	0,0070813	0,796	0,0417091	0,709	0,753
202256_at	**CD2BP2**	CD2 antigen (cytoplasmic tail) binding protein 2	0,0070931	0,843	0,0600756	0,874	0,859
1555266_a_at	**ASXL2**	additional sex combs like 2 (Drosophila)	0,0071891	0,780	0,0389613	0,833	0,807
212398_at	**RDX**	radixin	0,0071891	0,684	0,0269629	0,888	0,786
209237_s_at	**SLC23A2**	solute carrier family 23 (nucleobase transporters), member 2	0,0071891	0,776	0,0023703	0,578	0,677
227875_at	**KLHL13**	kelch-like 13 (Drosophila)	0,0072394	0,285	0,0036012	0,424	0,355
217862_at	**CALML4**	calmodulin-like 4	0,0072758	0,681	0,0541016	0,813	0,747
200925_at	**COX6A1**	cytochrome c oxidase subunit VIa polypeptide 1	0,0072758	0,871	0,0404417	0,872	0,872
1555961_a_at	**HINT1**	histidine triad nucleotide binding protein 1	0,0072758	0,871	0,0515635	0,923	0,897
1552299_at	**C6orf199**	chromosome 6 open reading frame 199	0,0073154	0,753	0,0065144	0,753	0,753
205388_at	**TNNC2**	troponin C2, fast	0,0073154	0,775	0,0232089	0,600	0,687
207469_s_at	**PIR**	pirin (iron-binding nuclear protein)	0,0074190	0,379	0,0038582	0,373	0,376
204321_at	**NEO1**	neogenin homolog 1 (chicken)	0,0074806	0,755	0,0232773	0,607	0,681
200062_s_at	**RPL30**	ribosomal protein L30	0,0075006	0,904	0,0270409	0,948	0,926
1554690_a_at	**TACC1**	transforming, acidic coiled-coil containing protein 1	0,0075439	0,630	0,0053893	0,610	0,620
224573_at	**MGC71993**	NA	0,0075724	0,784	0,0009353	0,741	0,763
212083_at	**TEX261**	testis expressed sequence 261	0,0076126	0,825	0,0381295	0,833	0,829
202501_at	**MAPRE2**	microtubule-associated protein, RP/EB family, member 2	0,0076515	0,725	0,0187973	0,768	0,746
225576_at	**C6orf72**	chromosome 6 open reading frame 72	0,0077619	0,759	0,0281709	0,710	0,734
201678_s_at	**DC12**	NA	0,0077619	0,773	0,0301214	0,821	0,797
1553243_at	**ITIH5**	inter-alpha (globulin) inhibitor H5	0,0077619	0,378	0,0103059	0,304	0,341
217987_at	**NS3TP1**	NA	0,0077619	0,808	0,0590022	0,860	0,834
209383_at	**DDIT3**	DNA-damage-inducible transcript 3	0,0077999	0,500	0,0175260	0,474	0,487
212146_at	**PLEKHM2**	pleckstrin homology domain containing, family M (with RUN domain) member 2	0,0077999	0,690	0,0277187	0,715	0,703
209075_s_at	**NIFUN**	NifU-like N-terminal domain containing	0,0078696	0,768	0,0521833	0,808	0,788
206076_at	**B7**	NA	0,0079461	0,759	0,0186073	0,762	0,760
203005_at	**LTBR**	lymphotoxin beta receptor (TNFR superfamily, member 3)	0,0079461	0,690	0,0335828	0,722	0,706
237764_at	**LOC389089**	NA	0,0079663	0,868	0,0201316	0,848	0,858
204863_s_at	**IL6ST**	interleukin 6 signal transducer (gp130, oncostatin M receptor)	0,0079890	0,518	0,0139671	0,714	0,616
228669_x_at	**PARP10**	poly (ADP-ribose) polymerase family, member 10	0,0080576	0,823	0,0356081	0,845	0,834
225975_at	**PCDH18**	protocadherin 18	0,0080999	0,567	0,0283980	0,475	0,521
218444_at	**ALG12**	asparagine-linked glycosylation 12 homolog (yeast, alpha-1,6-mannosyltransferase)	0,0081248	0,808	0,0000000	0,673	0,740
222860_s_at	**PDGFD**	platelet derived growth factor D	0,0081248	0,462	0,0376683	0,572	0,517
204554_at	**PPP1R3D**	protein phosphatase 1, regulatory subunit 3D	0,0081248	0,693	0,0019161	0,761	0,727

1555910_at	**PTCD2**	pentatricopeptide repeat domain 2	0,0081248	0,770	0,0478581	0,833	0,801
200862_at	**DHCR24**	24-dehydrocholesterol reductase	0,0082166	0,424	0,0451794	0,317	0,370
215844_at	**TNPO2**	transportin 2 (importin 3, karyopherin beta 2b)	0,0082171	0,797	0,0580472	0,813	0,805
1569025_s_at	**FAM13A1**	family with sequence similarity 13, member A1	0,0082231	0,694	0,0047651	0,589	0,641
227338_at	**THRAP3**	thyroid hormone receptor associated protein 3	0,0082231	0,731	0,0071441	0,682	0,706
225131_at	**ZRANB1**	zinc finger, RAN-binding domain containing 1	0,0082231	0,774	0,0214687	0,763	0,769
223189_x_at	**MLL5**	myeloid/lymphoid or mixed-lineage leukemia 5 (trithorax homolog, Drosophila)	0,0082263	0,611	0,0303756	0,884	0,748
230330_at	**PPM1D**	protein phosphatase 1D magnesium-dependent, delta isoform	0,0082499	0,772	0,0563678	0,739	0,755
221908_at	**FLJ14627**	NA	0,0082531	0,713	0,0424558	0,777	0,745
220605_s_at	**SIRT2**	sirtuin (silent mating type information regulation 2 homolog) 2 (S. cerevisiae)	0,0082607	0,760	0,0462859	0,831	0,795
224462_s_at	**CHCHD6**	coiled-coil-helix-coiled-coil-helix domain containing 6	0,0084089	0,797	0,0165121	0,868	0,833
201118_at	**PGD**	phosphogluconate dehydrogenase	0,0084089	0,499	0,0170496	0,571	0,535
203656_at	**KIAA0274**	KIAA0274	0,0084236	0,805	0,0122413	0,796	0,801
218563_at	**NDUFA3**	NADH dehydrogenase (ubiquinone) 1 alpha subcomplex, 3, 9kDa	0,0084236	0,797	0,0130605	0,814	0,806
205934_at	**PLCL1**	phospholipase C-like 1	0,0084236	0,456	0,0286020	0,479	0,468
204362_at	**SCAP2**	src family associated phosphoprotein 2	0,0084242	0,712	0,0270111	0,747	0,729
223868_s_at	**WWOX**	WW domain containing oxidoreductase	0,0084242	0,696	0,0022972	0,822	0,759
225521_at	**ANAPC7**	anaphase promoting complex subunit 7	0,0085224	0,635	0,0075912	0,697	0,666
218504_at	**FAHD2A**	fumarylacetoacetate hydrolase domain containing 2A	0,0085224	0,726	0,0532538	0,780	0,753
227880_s_at	**FAM11A**	family with sequence similarity 11, member A	0,0085224	0,833	0,0259338	0,814	0,824
65630_at	**LOC283232**	NA	0,0085224	0,635	0,0171692	0,668	0,651
232861_at	**PDP2**	NA	0,0085224	0,420	0,0455334	0,488	0,454
213223_at	**RPL28**	ribosomal protein L28	0,0085224	0,600	0,0071488	0,682	0,641
1554077_a_at	**TMEM53**	transmembrane protein 53	0,0085224	0,752	0,0020098	0,659	0,706
200023_s_at	**EIF3S5**	eukaryotic translation initiation factor 3, subunit 5 epsilon, 47kDa	0,0085619	0,763	0,0111363	0,822	0,792
209150_s_at	**TM9SF1**	transmembrane 9 superfamily member 1	0,0086039	0,870	0,0026073	0,780	0,825
240615_at	**PTOV1**	prostate tumor overexpressed gene 1	0,0086200	0,812	0,0008719	0,657	0,735
230795_at	**HIST2H4**	histone 2, H4	0,0086208	0,519	0,0251382	0,798	0,659
219346_at	**LRFN3**	leucine rich repeat and fibronectin type III domain containing 3	0,0086314	0,731	0,0384205	0,839	0,785
241955_at	**HECTD1**	HECT domain containing 1	0,0086523	0,598	0,0378926	0,577	0,587
224808_s_at	**C7orf20**	chromosome 7 open reading frame 20	0,0087083	0,832	0,0228315	0,886	0,859
201901_s_at	**YY1**	YY1 transcription factor	0,0087211	0,804	0,0342111	0,856	0,830
220152_at	**C10orf95**	chromosome 10 open reading frame 95	0,0088179	0,819	0,0304251	0,829	0,824
200704_at	**LITAF**	lipopolysaccharide-induced TNF factor	0,0088179	0,495	0,0006629	0,420	0,457
225055_at	**LOC440472**	NA	0,0088264	0,671	0,0047571	0,640	0,656
222067_x_at	**HIST1H2BD**	histone 1, H2bd	0,0088368	0,666	0,0257051	0,694	0,680
226258_at	**LOC196394**	NA	0,0088505	0,627	0,0161642	0,693	0,660
233250_x_at	**FLJ23322**	NA	0,0088722	0,756	0,0483058	0,866	0,811
220128_s_at	**NPAL2**	NIPA-like domain containing 2	0,0088773	0,702	0,0038582	0,642	0,672
224872_at	**KIAA1463**	NA	0,0088909	0,707	0,0149852	0,744	0,725
220761_s_at	**TAOK3**	TAO kinase 3	0,0089160	0,777	0,0093677	0,895	0,836
201412_at	**LRP10**	low density lipoprotein receptor-related protein 10	0,0089277	0,790	0,0021875	0,655	0,723
218246_at	**C1orf166**	chromosome 1 open reading frame 166	0,0089411	0,792	0,0270732	0,824	0,808
221688_s_at	**IMP3**	IMP3, U3 small nucleolar ribonucleoprotein, homolog (yeast)	0,0089411	0,764	0,0271294	0,858	0,811
244266_at	**AKR1C2**	c("aldo-keto reductase family 1, member C2 (dihydrodiol dehydrogenase 2", " bile acid binding protein", " 3-alpha hydroxysteroid dehydrogenase, type III)")	0,0089527	0,786	0,0230909	0,428	0,607
1556389_at	**TNRC5**	trinucleotide repeat containing 5	0,0089841	0,672	0,0098725	0,711	0,691
212186_at	**ACACA**	acetyl-Coenzyme A carboxylase alpha	0,0090280	0,642	0,0057709	0,631	0,637
211006_s_at	**KCNB1**	potassium voltage-gated channel, Shab-related subfamily, member 1	0,0090280	0,242	0,0295704	0,327	0,285
203152_at	**MRPL40**	mitochondrial ribosomal protein L40	0,0090280	0,791	0,0232449	0,833	0,812
201164_s_at	**PUM1**	pumilio homolog 1 (Drosophila)	0,0090280	0,803	0,0154509	0,826	0,814
209814_at	**ZNF330**	zinc finger protein 330	0,0090280	0,677	0,0204560	0,685	0,681
224445_s_at	**ZFYVE21**	zinc finger, FYVE domain containing 21	0,0090360	0,795	0,0012063	0,755	0,775
218418_s_at	**ANKRD25**	ankyrin repeat domain 25	0,0090649	0,731	0,0245690	0,693	0,712
201576_s_at	**GLB1**	galactosidase, beta 1	0,0090649	0,784	0,0021152	0,671	0,727
222403_at	**MTCH2**	mitochondrial carrier homolog 2 (C. elegans)	0,0090825	0,787	0,0032293	0,826	0,807
1558184_s_at	**ZNF17**	zinc finger protein 17 (HPF3, KOX 10)	0,0090825	0,753	0,0096991	0,793	0,773
229729_at	**C9orf127**	chromosome 9 open reading frame 127	0,0090920	0,793	0,0003110	0,745	0,769
218402_s_at	**HPS4**	Hermansky-Pudlak syndrome 4	0,0091209	0,772	0,0034045	0,727	0,750
1552825_at	**ZNF396**	zinc finger protein 396	0,0091529	0,873	0,0388465	0,729	0,801
209581_at	**HRASLS3**	HRAS-like suppressor 3	0,0092197	0,623	0,0014814	0,596	0,609
218285_s_at	**DHRS6**	dehydrogenase/reductase (SDR family) member 6	0,0092210	0,641	0,0571282	0,720	0,680
204992_s_at	**PFN2**	profilin 2	0,0093026	0,718	0,0014977	0,678	0,698

224931_at	**SLC41A3**	solute carrier family 41, member 3	0,0093026	0,653	0,0021377	0,701	0,677
208710_s_at	**AP3D1**	adaptor-related protein complex 3, delta 1 subunit	0,0093093	0,820	0,0251783	0,688	0,754
1557991_at	**MGC24132**	NA	0,0093093	0,801	0,0264684	0,809	0,805
1569277_at	**ZNF91**	zinc finger protein 91 (HPF7, HTF10)	0,0093093	0,892	0,0178131	0,726	0,809
209627_s_at	**OSBPL3**	oxysterol binding protein-like 3	0,0093197	0,570	0,0021056	0,511	0,541
221827_at	**C20orf18**	chromosome 20 open reading frame 18	0,0093225	0,721	0,0043973	0,668	0,694
217100_s_at	**KIAA0794**	NA	0,0093435	0,817	0,0406713	0,885	0,851
218480_at	**FLJ21839**	NA	0,0094001	0,683	0,0005337	0,638	0,661
201247_at	**SREBF2**	sterol regulatory element binding transcription factor 2	0,0094813	0,624	0,0002160	0,555	0,589
211994_at	**WNK1**	WNK lysine deficient protein kinase 1	0,0095164	0,760	0,0099082	0,751	0,756
225296_at	**ZNF317**	zinc finger protein 317	0,0095164	0,818	0,0356081	0,866	0,842
217918_at	**DNCL2A**	dynein, cytoplasmic, light polypeptide 2A	0,0095710	0,794	0,0183053	0,752	0,773
214436_at	**FBXL2**	F-box and leucine-rich repeat protein 2	0,0095710	0,631	0,0232207	0,648	0,639
212451_at	**KIAA0256**	NA	0,0095710	0,579	0,0240516	0,519	0,549
204738_s_at	**KRIT1**	KRIT1, ankyrin repeat containing	0,0095710	0,770	0,0029435	0,663	0,716
225030_at	**FAM44B**	family with sequence similarity 44, member B	0,0096110	0,685	0,0185098	0,764	0,724
234855_at	**ORF1**	NA	0,0096110	0,871	0,0177244	0,867	0,869
217829_s_at	**USP39**	ubiquitin specific peptidase 39	0,0096133	0,831	0,0014456	0,824	0,827
235758_at	**PNMA6A**	paraneoplastic antigen like 6A	0,0096175	0,570	0,0461538	0,570	0,570
226800_at	**KIAA1799**	NA	0,0096396	0,534	0,0339816	0,570	0,552
222209_s_at	**FLJ22104**	NA	0,0096468	0,562	0,0338922	0,768	0,665
208003_s_at	**NFAT5**	nuclear factor of activated T-cells 5, tonicity-responsive	0,0096556	0,575	0,0531852	0,813	0,694
200831_s_at	**SCD**	stearoyl-CoA desaturase (delta-9-desaturase)	0,0096556	0,329	0,0395334	0,406	0,368
221206_at	**PMS2**	PMS2 postmeiotic segregation increased 2 (S. cerevisiae)	0,0096811	0,738	0,0007641	0,684	0,711
204849_at	**TCFL5**	transcription factor-like 5 (basic helix-loop-helix)	0,0096811	0,672	0,0181179	0,629	0,650
218597_s_at	**C10orf70**	chromosome 10 open reading frame 70	0,0097013	0,742	0,0453474	0,847	0,794
204778_x_at	**HOXB7**	homeo box B7	0,0097144	0,781	0,0534319	0,775	0,778
224813_at	**WASL**	Wiskott-Aldrich syndrome-like	0,0097453	0,767	0,0040349	0,629	0,698
222109_at	**GNL3L**	guanine nucleotide binding protein-like 3 (nucleolar)-like	0,0100114	0,771	0,0395334	0,773	0,772
217978_s_at	**UBE2Q1**	ubiquitin-conjugating enzyme E2Q (putative) 1	0,0100114	0,841	0,0232089	0,828	0,834
216520_s_at	**TPT1**	tumor protein, translationally-controlled 1	0,0100243	0,920	0,0344502	0,965	0,942
202261_at	**VPS72**	vacuolar protein sorting 72 (yeast)	0,0100429	0,856	0,0563537	0,876	0,866
202204_s_at	**AMFR**	autocrine motility factor receptor	0,0101076	0,762	0,0007567	0,725	0,743
215157_x_at	**PABPC1**	poly(A) binding protein, cytoplasmic 1	0,0101233	0,826	0,0214906	0,857	0,841
213312_at	**C6orf162**	chromosome 6 open reading frame 162	0,0102262	0,606	0,0388794	0,804	0,705
33494_at	**ETFDH**	electron-transferring-flavoprotein dehydrogenase	0,0102669	0,759	0,0329204	0,761	0,760
203458_at	**SPR**	sepiapterin reductase (7,8-dihydrobiopterin:NADP+ oxidoreductase)	0,0102941	0,788	0,0000517	0,657	0,722
209123_at	**QDPR**	quinoid dihydropteridine reductase	0,0103194	0,795	0,0362183	0,803	0,799
225460_at	**SEC22L3**	SEC22 vesicle trafficking protein-like 3 (S. cerevisiae)	0,0103251	0,805	0,0031271	0,673	0,739
219103_at	**DDEFL1**	development and differentiation enhancing factor-like 1	0,0103652	0,497	0,0003910	0,369	0,433
224676_at	**TMED4**	transmembrane emp24 protein transport domain containing 4	0,0103967	0,667	0,0337287	0,720	0,694
202171_at	**ZNF161**	zinc finger protein 161	0,0104185	0,696	0,0321800	0,710	0,703
223173_at	**SPIN1**	NA	0,0104252	0,709	0,0097998	0,721	0,715
213577_at	**SQLE**	squalene epoxidase	0,0104374	0,666	0,0003110	0,655	0,660
203538_at	**CAMLG**	calcium modulating ligand	0,0105512	0,651	0,0036298	0,717	0,684
221808_at	**RAB9A**	RAB9A, member RAS oncogene family	0,0105512	0,627	0,0186595	0,739	0,683
225531_at	**CABLES1**	Cdk5 and Abl enzyme substrate 1	0,0105605	0,634	0,0074248	0,607	0,620
242100_at	**CSS3**	NA	0,0105689	0,600	0,0243770	0,656	0,628
208912_s_at	**CNP**	2',3'-cyclic nucleotide 3' phosphodiesterase	0,0105815	0,733	0,0514912	0,896	0,814
211514_at	**RIPK5**	receptor interacting protein kinase 5	0,0106644	0,794	0,0005784	0,799	0,796
223517_at	**FBXO44**	F-box protein 44	0,0106976	0,736	0,0006545	0,718	0,727
216741_at	**GLIS1**	GLIS family zinc finger 1	0,0107477	0,830	0,0502198	0,910	0,870
219237_s_at	**DNAJB14**	DnaJ (Hsp40) homolog, subfamily B, member 14	0,0107829	0,775	0,0327572	0,839	0,807
201927_s_at	**PKP4**	plakophilin 4	0,0107897	0,630	0,0301569	0,823	0,727
219567_s_at	**C1orf176**	chromosome 1 open reading frame 176	0,0107988	0,764	0,0179623	0,806	0,785
226031_at	**FLJ20097**	NA	0,0108212	0,822	0,0379790	0,868	0,845
222143_s_at	**FLJ22405**	NA	0,0108420	0,827	0,0436068	0,912	0,869
48031_r_at	**C5orf4**	chromosome 5 open reading frame 4	0,0108470	0,611	0,0275515	0,474	0,542
213408_s_at	**LOC375133**	NA	0,0108475	0,786	0,0197154	0,874	0,830
228456_s_at	**LOC149832**	NA	0,0108907	0,755	0,0173519	0,588	0,672
212914_at	**CBX7**	chromobox homolog 7	0,0109894	0,697	0,0023942	0,570	0,634
231864_at	**ZNF33A**	zinc finger protein 33A	0,0109894	0,640	0,0186571	0,623	0,632
218500_at	**C8orf55**	chromosome 8 open reading frame 55	0,0110231	0,736	0,0002160	0,610	0,673
227585_at	**ATAD1**	ATPase family, AAA domain containing 1	0,0110438	0,688	0,0214906	0,748	0,718
1554082_a_at	**NOL9**	nucleolar protein 9	0,0110661	0,725	0,0074649	0,773	0,749

207305_s_at	**KIAA1012**	KIAA1012	0,0111410	0,822	0,0440810	0,849	0,835
221550_at	**COX15**	COX15 homolog, cytochrome c oxidase assembly protein (yeast)	0,0112057	0,692	0,0217396	0,737	0,714
204181_s_at	**ZNF297B**	zinc finger protein 297B	0,0112749	0,766	0,0035777	0,728	0,747
209279_s_at	**NSDHL**	NAD(P) dependent steroid dehydrogenase-like	0,0112779	0,709	0,0432118	0,813	0,761
235614_at	**MGC33486**	NA	0,0113116	0,797	0,0033006	0,742	0,769
1554547_at	**FAM13C1**	family with sequence similarity 13, member C1	0,0113365	0,193	0,0380027	0,153	0,173
236356_at	**NDUFS1**	NADH dehydrogenase (ubiquinone) Fe-S protein 1, 75kDa (NADH-coenzyme Q reductase)	0,0113512	0,524	0,0006629	0,563	0,544
231769_at	**FBXO6**	F-box protein 6	0,0113527	0,667	0,0058311	0,587	0,627
210142_x_at	**FLOT1**	flotillin 1	0,0113557	0,751	0,0045000	0,684	0,718
204529_s_at	**TOX**	NA	0,0113586	0,294	0,0114590	0,259	0,277
200743_s_at	**TPP1**	tripeptidyl peptidase I	0,0113869	0,660	0,0388794	0,678	0,669
212156_at	**VPS39**	vacuolar protein sorting 39 (yeast)	0,0113869	0,756	0,0023107	0,801	0,779
205964_at	**ZNF426**	zinc finger protein 426	0,0113869	0,661	0,0223679	0,687	0,674
243038_at	**FLJ45645**	NA	0,0114120	0,674	0,0068464	0,735	0,704
217868_s_at	**DREV1**	NA	0,0114774	0,786	0,0515068	0,868	0,827
228089_x_at	**LOC374395**	NA	0,0114774	0,801	0,0486469	0,854	0,827
234971_x_at	**PLCD3**	phospholipase C, delta 3	0,0115051	0,779	0,0039797	0,707	0,743
232981_s_at	**AP1GBP1**	AP1 gamma subunit binding protein 1	0,0116570	0,779	0,0451778	0,708	0,744
1556427_s_at	**LOC221091**	NA	0,0117648	0,329	0,0025412	0,238	0,283
209784_s_at	**JAG2**	jagged 2	0,0117757	0,819	0,0012460	0,695	0,757
201256_at	**COX7A2L**	cytochrome c oxidase subunit VIIa polypeptide 2 like	0,0118108	0,788	0,0000000	0,786	0,787
209538_at	**ZNF32**	zinc finger protein 32 (KOX 30)	0,0118108	0,667	0,0324169	0,736	0,702
203859_s_at	**PALM**	paralemmin	0,0118214	0,618	0,0000000	0,460	0,539
231933_at	**MARCH8**	membrane-associated ring finger (C3HC4) 8	0,0118559	0,848	0,0487607	0,737	0,792
1558693_s_at	**C1orf85**	chromosome 1 open reading frame 85	0,0118642	0,563	0,0502354	0,710	0,637
214600_at	**TEAD1**	TEA domain family member 1 (SV40 transcriptional enhancer factor)	0,0118642	0,654	0,0332783	0,866	0,760
227632_at	**KIAA1171**	NA	0,0118801	0,738	0,0095495	0,648	0,693
212761_at	**TCF7L2**	transcription factor 7-like 2 (T-cell specific, HMG-box)	0,0119196	0,671	0,0084466	0,666	0,668
219137_s_at	**C2orf33**	chromosome 2 open reading frame 33	0,0119932	0,808	0,0355131	0,806	0,807
1558685_a_at	**LOC158960**	NA	0,0120220	0,664	0,0068750	0,620	0,642
220631_at	**OSGEPL1**	O-sialoglycoprotein endopeptidase-like 1	0,0120559	0,643	0,0175002	0,739	0,691
222819_at	**CTPS2**	CTP synthase II	0,0120998	0,715	0,0089870	0,748	0,732
204791_at	**NR2C1**	nuclear receptor subfamily 2, group C, member 1	0,0120998	0,776	0,0232588	0,800	0,788
227841_at	**CGI-14**	NA	0,0121075	0,701	0,0011723	0,623	0,662
221192_x_at	**ET**	NA	0,0121566	0,855	0,0254548	0,857	0,856
203835_at	**LRRC32**	leucine rich repeat containing 32	0,0122495	0,610	0,0255368	0,610	0,610
221015_s_at	**CDADC1**	cytidine and dCMP deaminase domain containing 1	0,0122778	0,780	0,0034100	0,714	0,747
235005_at	**MGC4562**	NA	0,0122875	0,672	0,0314684	0,651	0,662
206790_s_at	**NDUFB1**	NADH dehydrogenase (ubiquinone) 1 beta subcomplex, 1, 7kDa	0,0123053	0,821	0,0331545	0,871	0,846
232594_at	**LOC440498**	NA	0,0123072	0,785	0,0212705	0,694	0,740
226589_at	**FLJ38482**	NA	0,0124165	0,587	0,0270732	0,654	0,621
233790_at	**GNG7**	guanine nucleotide binding protein (G protein), gamma 7	0,0124350	0,878	0,0320467	0,704	0,791
201674_s_at	**AKAP1**	A kinase (PRKA) anchor protein 1	0,0126098	0,706	0,0409683	0,689	0,698
209790_s_at	**CASP6**	caspase 6, apoptosis-related cysteine peptidase	0,0126098	0,607	0,0397336	0,782	0,694
208964_s_at	**FADS1**	fatty acid desaturase 1	0,0126098	0,324	0,0355988	0,220	0,272
212017_at	**LOC130074**	NA	0,0126098	0,798	0,0448353	0,812	0,805
211063_s_at	**NCK1**	NCK adaptor protein 1	0,0126950	0,730	0,0288568	0,707	0,718
227225_at	**ZNF503**	zinc finger protein 503	0,0126950	0,795	0,0271900	0,757	0,776
228525_at	**SLC7A10**	solute carrier family 7, (neutral amino acid transporter, y+ system) member 10	0,0127022	0,769	0,0136647	0,661	0,715
215816_at	**LOC91316**	NA	0,0127390	0,895	0,0337287	0,825	0,860
239039_at	**ANGPTL2**	angiopoietin-like 2	0,0128627	0,741	0,0570896	0,599	0,670
213551_x_at	**PCGF2**	polycomb group ring finger 2	0,0128962	0,814	0,0118761	0,783	0,799
208945_s_at	**BECN1**	beclin 1 (coiled-coil, myosin-like BCL2 interacting protein)	0,0129329	0,766	0,0493608	0,880	0,823
230009_at	**FLJ21103**	NA	0,0129458	0,745	0,0012744	0,820	0,782
241428_x_at	**TCEA2**	transcription elongation factor A (SII), 2	0,0129471	0,815	0,0212480	0,826	0,820
218169_at	**VAC14**	Vac14 homolog (S. cerevisiae)	0,0129620	0,709	0,0013263	0,645	0,677
1569433_at	**LOC389432**	NA	0,0130027	0,463	0,0063512	0,315	0,389
221750_at	**HMGCS1**	3-hydroxy-3-methylglutaryl-Coenzyme A synthase 1 (soluble)	0,0130232	0,539	0,0100680	0,608	0,573
225089_at	**USP40**	ubiquitin specific peptidase 40	0,0130232	0,645	0,0045178	0,742	0,694
209623_at	**MCCC2**	methylcrotonoyl-Coenzyme A carboxylase 2 (beta)	0,0130374	0,846	0,0427028	0,864	0,855
235722_at	**SYNJ2BP**	synaptojanin 2 binding protein	0,0130374	0,689	0,0026651	0,759	0,724

221471_at	**TDE1**	tumor differentially expressed 1	0,0130374	0,762	0,0173264	0,624	0,693
206138_s_at	**PIK4CB**	phosphatidylinositol 4-kinase, catalytic, beta polypeptide	0,0131182	0,827	0,0282384	0,729	0,778
213141_at	**PSKH1**	protein serine kinase H1	0,0131182	0,795	0,0012315	0,775	0,785
223412_at	**KBTBD7**	kelch repeat and BTB (POZ) domain containing 7	0,0131504	0,693	0,0311063	0,677	0,685
222893_s_at	**C1orf82**	chromosome 1 open reading frame 82	0,0131757	0,796	0,0498936	0,722	0,759
209283_at	**CRYAB**	crystallin, alpha B	0,0132020	0,565	0,0003110	0,476	0,521
226495_at	**LOC440752**	NA	0,0132456	0,759	0,0240320	0,779	0,769
200901_s_at	**M6PR**	mannose-6-phosphate receptor (cation dependent)	0,0132456	0,743	0,0033528	0,745	0,744
222576_s_at	**EIF2C1**	eukaryotic translation initiation factor 2C, 1	0,0132723	0,758	0,0277598	0,751	0,755
1565657_at	**CKLFSF6**	chemokine-like factor superfamily 6	0,0132921	0,935	0,0556460	0,846	0,891
227136_s_at	**C10orf46**	chromosome 10 open reading frame 46	0,0133093	0,811	0,0263238	0,858	0,834
202082_s_at	**SEC14L1**	SEC14-like 1 (S. cerevisiae)	0,0133093	0,581	0,0273595	0,777	0,679
211703_s_at	**TM2D1**	TM2 domain containing 1	0,0133093	0,802	0,0170671	0,777	0,789
220040_x_at	**KIAA1166**	KIAA1166	0,0133209	0,788	0,0005899	0,707	0,747
223559_s_at	**C9orf80**	chromosome 9 open reading frame 80	0,0133450	0,721	0,0363181	0,916	0,819
205364_at	**ACOX2**	acyl-Coenzyme A oxidase 2, branched chain	0,0133592	0,622	0,0044184	0,647	0,634
208858_s_at	**FAM62A**	family with sequence similarity 62 (C2 domain containing), member A	0,0133592	0,824	0,0304079	0,632	0,728
220321_s_at	**FLJ13646**	NA	0,0133592	0,709	0,0418895	0,855	0,782
219566_at	**PLEKHF1**	pleckstrin homology domain containing, family F (with FYVE domain) member 1	0,0133592	0,631	0,0069856	0,691	0,661
222148_s_at	**RHOT1**	ras homolog gene family, member T1	0,0133592	0,823	0,0095416	0,750	0,786
211085_s_at	**STK4**	serine/threonine kinase 4	0,0133592	0,745	0,0467125	0,732	0,738
229712_at	**PSIP1**	PC4 and SFRS1 interacting protein 1	0,0133836	0,672	0,0087062	0,709	0,690
213145_at	**FBXL14**	F-box and leucine-rich repeat protein 14	0,0133889	0,738	0,0405587	0,806	0,772
211938_at	**EIF4B**	eukaryotic translation initiation factor 4B	0,0134070	0,676	0,0297622	0,749	0,712
219909_at	**MMP28**	matrix metallopeptidase 28	0,0134327	0,620	0,0246764	0,481	0,550
208860_s_at	**ATRX**	alpha thalassemia/mental retardation syndrome X-linked (RAD54 homolog, S. cerevisiae)	0,0134426	0,753	0,0521873	0,720	0,736
243777_at	**RAB7L1**	RAB7, member RAS oncogene family-like 1	0,0134443	0,759	0,0001933	0,457	0,608
225287_s_at	**TMEM55B**	transmembrane protein 55B	0,0134635	0,812	0,0513316	0,765	0,788
227172_at	**LOC89894**	NA	0,0135282	0,589	0,0174822	0,555	0,572
204546_at	**KIAA0513**	KIAA0513	0,0135317	0,705	0,0021024	0,640	0,672
212119_at	**RHOQ**	ras homolog gene family, member Q	0,0135383	0,664	0,0233822	0,758	0,711
205571_at	**LIPT1**	lipoyltransferase 1	0,0135483	0,519	0,0473537	0,624	0,571
204568_at	**KIAA0831**	KIAA0831	0,0135746	0,751	0,0177635	0,762	0,757
238476_at	**LOC153222**	adult retina protein	0,0135746	0,625	0,0103596	0,601	0,613
217990_at	**GMPR2**	guanosine monophosphate reductase 2	0,0136284	0,730	0,0602339	0,800	0,765
223711_s_at	**THY28**	NA	0,0136406	0,746	0,0208915	0,755	0,750
221979_at	**TOPORS**	topoisomerase I binding, arginine/serine-rich	0,0136406	0,760	0,0050915	0,767	0,764
224664_at	**C10orf104**	chromosome 10 open reading frame 104	0,0137710	0,740	0,0306682	0,755	0,748
225629_s_at	**ZBTB4**	zinc finger and BTB domain containing 4	0,0137799	0,788	0,0204257	0,664	0,726
212041_at	**ATP6V0D1**	ATPase, H+ transporting, lysosomal 38kDa, V0 subunit d isoform 1	0,0138107	0,780	0,0253362	0,759	0,770
214909_s_at	**DDAH2**	dimethylarginine dimethylaminohydrolase 2	0,0139434	0,725	0,0542185	0,683	0,704
1557169_x_at	**HCG11**	HLA complex group 11	0,0140482	0,609	0,0390259	0,630	0,620
220417_s_at	**THAP4**	THAP domain containing 4	0,0141561	0,819	0,0286213	0,742	0,780
218453_s_at	**C6orf35**	chromosome 6 open reading frame 35	0,0141747	0,738	0,0576362	0,806	0,772
231716_at	**MNAB**	membrane associated DNA binding protein	0,0141903	0,799	0,0572020	0,800	0,799
226214_at	**MIR16**	NA	0,0142184	0,780	0,0095416	0,805	0,792
1566603_s_at	**RPUSD3**	RNA pseudouridylate synthase domain containing 3	0,0142369	0,784	0,0495274	0,917	0,850
204247_s_at	**CDK5**	cyclin-dependent kinase 5	0,0142531	0,828	0,0111021	0,734	0,781
1554628_at	**LOC126295**	NA	0,0142817	0,777	0,0256317	0,751	0,764
223100_s_at	**NUDT5**	nudix (nucleoside diphosphate linked moiety X)-type motif 5	0,0142817	0,816	0,0193734	0,701	0,758
204303_s_at	**KIAA0427**	KIAA0427	0,0144014	0,681	0,0216910	0,719	0,700
226159_at	**LOC285636**	NA	0,0144445	0,813	0,0288568	0,832	0,822
223008_s_at	**C9orf5**	chromosome 9 open reading frame 5	0,0144973	0,721	0,0005607	0,671	0,696
242214_at	**LOC388720**	NA	0,0145611	0,640	0,0108616	0,787	0,714
203516_at	**SNTA1**	syntrophin, alpha 1 (dystrophin-associated protein A1, 59kDa, acidic component)	0,0145611	0,808	0,0002614	0,509	0,658
1553301_a_at	**FLJ30294**	NA	0,0145996	0,716	0,0320653	0,801	0,759
210624_s_at	**ILVBL**	ilvB (bacterial acetolactate synthase)-like	0,0146251	0,812	0,0020442	0,715	0,763
204295_at	**SURF1**	surfeit 1	0,0146251	0,765	0,0070092	0,745	0,755
235626_at	**CAMK1D**	calcium/calmodulin-dependent protein kinase ID	0,0146395	0,731	0,0072349	0,682	0,706
209092_s_at	**C17orf25**	chromosome 17 open reading frame 25	0,0146555	0,785	0,0302242	0,859	0,822
208660_at	**CS**	citrate synthase	0,0146764	0,792	0,0008719	0,788	0,790
227959_at	**ANKRD9**	ankyrin repeat domain 9	0,0146905	0,560	0,0294436	0,633	0,596
208678_at	**ATP6V1E1**	ATPase, H+ transporting, lysosomal 31kDa, V1 subunit E isoform 1	0,0147289	0,731	0,0342459	0,783	0,757
209806_at	**HIST1H2BK**	histone 1, H2bk	0,0147289	0,687	0,0113039	0,690	0,689

202090_s_at	**UQCR**	ubiquinol-cytochrome c reductase, 6.4kDa subunit	0,0147300	0,889	0,0387133	0,874	0,881
228368_at	**ARHGAP20**	Rho GTPase activating protein 20	0,0147884	0,259	0,0148603	0,228	0,243
201540_at	**FHL1**	four and a half LIM domains 1	0,0147884	0,664	0,0486766	0,643	0,653
212910_at	**THAP11**	THAP domain containing 11	0,0147884	0,765	0,0164350	0,827	0,796
219219_at	**FLJ20512**	NA	0,0148602	0,691	0,0014728	0,716	0,703
211260_at	**BMP7**	bone morphogenetic protein 7 (osteogenic protein 1)	0,0148865	0,837	0,0223679	0,886	0,862
201036_s_at	**HADHSC**	L-3-hydroxyacyl-Coenzyme A dehydrogenase, short chain	0,0149061	0,770	0,0090508	0,759	0,765
1553940_a_at	**LRRC28**	leucine rich repeat containing 28	0,0149061	0,652	0,0230671	0,857	0,754
235698_at	**ZFP90**	zinc finger protein 90 homolog (mouse)	0,0149061	0,637	0,0013125	0,696	0,666
234628_at	**RAB28**	RAB28, member RAS oncogene family	0,0149137	0,880	0,0491268	0,919	0,900
202804_at	**ABCC1**	ATP-binding cassette, sub-family C (CFTR/MRP), member 1	0,0150065	0,747	0,0219456	0,745	0,746
201972_at	**ATP6V1A**	ATPase, H+ transporting, lysosomal 70kDa, V1 subunit A	0,0150065	0,642	0,0231221	0,650	0,646
235396_at	**DKFZp761P1121**	NA	0,0150065	0,754	0,0000000	0,623	0,688
208490_x_at	**HIST1H2BF**	histone 1, H2bf	0,0150065	0,676	0,0401700	0,775	0,725
232035_at	**HIST1H4H**	histone 1, H4h	0,0150065	0,488	0,0006153	0,533	0,510
222616_s_at	**USP16**	ubiquitin specific peptidase 16	0,0150065	0,721	0,0518212	0,859	0,790
209155_s_at	**NT5C2**	5'-nucleotidase, cytosolic II	0,0150583	0,784	0,0200268	0,772	0,778
227264_at	**TRAF6**	TNF receptor-associated factor 6	0,0150760	0,759	0,0003594	0,822	0,791
226668_at	**WDSUB1**	WD repeat, SAM and U-box domain containing 1	0,0150760	0,579	0,0112762	0,593	0,586
224682_at	**ANKIB1**	ankyrin repeat and IBR domain containing 1	0,0150792	0,773	0,0043825	0,758	0,765
203173_s_at	**MGC16824**	NA	0,0151428	0,830	0,0119039	0,856	0,843
210976_s_at	**PFKM**	phosphofructokinase, muscle	0,0151428	0,681	0,0068974	0,720	0,700
220216_at	**C8orf44**	chromosome 8 open reading frame 44	0,0151540	0,813	0,0418991	0,783	0,798
218354_at	**HSPC176**	NA	0,0152523	0,715	0,0563564	0,837	0,776
221256_s_at	**HDHD3**	haloacid dehalogenase-like hydrolase domain containing 3	0,0152957	0,575	0,0001600	0,527	0,551
212121_at	**C10orf61**	chromosome 10 open reading frame 61	0,0153048	0,781	0,0020088	0,744	0,762
242082_at	**MMAB**	methylmalonic aciduria (cobalamin deficiency) cblB type	0,0153048	0,727	0,0338189	0,746	0,736
204730_at	**RIMS3**	regulating synaptic membrane exocytosis 3	0,0153193	0,315	0,0303812	0,315	0,315
213433_at	**ARL3**	ADP-ribosylation factor-like 3	0,0153501	0,834	0,0015063	0,751	0,792
201089_at	**ATP6V1B2**	ATPase, H+ transporting, lysosomal 56/58kDa, V1 subunit B, isoform 2	0,0153501	0,732	0,0561409	0,799	0,765
201570_at	**CGI-51**	NA	0,0153501	0,842	0,0433316	0,902	0,872
218692_at	**FLJ20366**	NA	0,0153501	0,388	0,0221912	0,327	0,358
218173_s_at	**WHSC1L1**	Wolf-Hirschhorn syndrome candidate 1-like 1	0,0153501	0,805	0,0009353	0,657	0,731
202398_at	**AP3S2**	adaptor-related protein complex 3, sigma 2 subunit	0,0153920	0,857	0,0498751	0,831	0,844
228523_at	**NANOS1**	nanos homolog 1 (Drosophila)	0,0154398	0,418	0,0315766	0,268	0,343
238693_at	**PHC3**	polyhomeotic like 3 (Drosophila)	0,0154398	0,691	0,0268317	0,855	0,773
200694_s_at	**DDX24**	DEAD (Asp-Glu-Ala-Asp) box polypeptide 24	0,0154421	0,777	0,0000313	0,592	0,684
212686_at	**PPM1H**	protein phosphatase 1H (PP2C domain containing)	0,0155279	0,583	0,0068209	0,481	0,532
226527_at	**KIAA0460**	KIAA0460	0,0155552	0,792	0,0034050	0,763	0,777
234996_at	**CALCRL**	calcitonin receptor-like	0,0156051	0,700	0,0079970	0,613	0,656
1555536_at	**ANTXR2**	anthrax toxin receptor 2	0,0156891	0,668	0,0058654	0,611	0,640
217543_s_at	**MBTPS1**	membrane-bound transcription factor peptidase, site 1	0,0156935	0,824	0,0044184	0,826	0,825
230876_at	**LOC169834**	NA	0,0157275	0,480	0,0354872	0,609	0,545
203814_s_at	**NQO2**	NAD(P)H dehydrogenase, quinone 2	0,0158242	0,647	0,0339466	0,637	0,642
226843_s_at	**PAPD5**	PAP associated domain containing 5	0,0158242	0,861	0,0015269	0,771	0,816
201182_s_at	**CHD4**	chromodomain helicase DNA binding protein 4	0,0158542	0,716	0,0102123	0,806	0,761
214176_s_at	**PBXIP1**	pre-B-cell leukemia transcription factor interacting protein 1	0,0159243	0,664	0,0424148	0,741	0,703
210613_s_at	**SYNGR1**	synaptogyrin 1	0,0159278	0,650	0,0323097	0,638	0,644
234728_s_at	**DHX35**	DEAH (Asp-Glu-Ala-His) box polypeptide 35	0,0159792	0,811	0,0567808	0,802	0,807
212813_at	**JAM3**	junctional adhesion molecule 3	0,0159879	0,772	0,0316729	0,740	0,756
209146_at	**SC4MOL**	sterol-C4-methyl oxidase-like	0,0160152	0,494	0,0026867	0,502	0,498
221777_at	**FLJ14827**	NA	0,0160304	0,843	0,0201261	0,793	0,818
238778_at	**MPP7**	membrane protein, palmitoylated 7 (MAGUK p55 subfamily member 7)	0,0160867	0,764	0,0027820	0,679	0,721
218099_at	**HT008**	NA	0,0161673	0,819	0,0349218	0,849	0,834
223136_at	**AIG1**	androgen-induced 1	0,0161958	0,605	0,0285034	0,807	0,706
212805_at	**KIAA0367**	KIAA0367	0,0161958	0,623	0,0036613	0,609	0,616
242370_at	**MTHFD2L**	methylenetetrahydrofolate dehydrogenase (NADP+ dependent) 2-like	0,0162403	0,907	0,0313164	0,876	0,892
222749_at	**SUFU**	suppressor of fused homolog (Drosophila)	0,0162403	0,778	0,0004756	0,693	0,735
212399_s_at	**VGLL4**	vestigial like 4 (Drosophila)	0,0162485	0,765	0,0369148	0,810	0,787
214778_at	**EGFL4**	EGF-like-domain, multiple 4	0,0163508	0,587	0,0015492	0,605	0,596
213799_s_at	**PTPRA**	protein tyrosine phosphatase, receptor type, A	0,0163823	0,727	0,0052829	0,721	0,724

219115_s_at	**IL20RA**	interleukin 20 receptor, alpha	0,0163888	0,903	0,0153813	0,856	0,880
201924_at	**AFF1**	AF4/FMR2 family, member 1	0,0163934	0,774	0,0394432	0,723	0,749
228993_s_at	**PDCD4**	programmed cell death 4 (neoplastic transformation inhibitor)	0,0163934	0,615	0,0372456	0,740	0,677
1553852_at	**VPS13B**	vacuolar protein sorting 13B (yeast)	0,0164075	0,799	0,0393246	0,666	0,732
212078_s_at	**MLL**	myeloid/lymphoid or mixed-lineage leukemia (trithorax homolog, Drosophila)	0,0164118	0,815	0,0365116	0,703	0,759
225427_s_at	**APOA1BP**	apolipoprotein A-I binding protein	0,0164741	0,799	0,0006372	0,765	0,782
235109_at	**ZBED3**	zinc finger, BED-type containing 3	0,0164741	0,528	0,0104296	0,489	0,509
1554021_a_at	**ZNF12**	zinc finger protein 12	0,0164810	0,747	0,0336444	0,740	0,743
218416_s_at	**HDAC7A**	histone deacetylase 7A	0,0165021	0,761	0,0261162	0,598	0,680
210946_at	**PPAP2A**	phosphatidic acid phosphatase type 2A	0,0165693	0,623	0,0065496	0,658	0,640
214464_at	**CDC42BPA**	CDC42 binding protein kinase alpha (DMPK-like)	0,0165934	0,583	0,0000517	0,709	0,646
224862_at	**GNAQ**	guanine nucleotide binding protein (G protein), q polypeptide	0,0167042	0,827	0,0254915	0,907	0,867
235393_at	**RG9MTD3**	RNA (guanine-9-) methyltransferase domain containing 3	0,0167042	0,814	0,0026867	0,826	0,820
227897_at	**RAP2B**	RAP2B, member of RAS oncogene family	0,0167168	0,658	0,0531501	0,574	0,616
203833_s_at	**TGOLN2**	trans-golgi network protein 2	0,0167211	0,809	0,0436328	0,812	0,810
222605_at	**RCOR3**	REST corepressor 3	0,0168141	0,750	0,0079928	0,745	0,748
226815_at	**MGC16471**	NA	0,0168651	0,826	0,0447524	0,840	0,833
209198_s_at	**SYT11**	synaptotagmin XI	0,0168833	0,734	0,0495518	0,789	0,762
202537_s_at	**CHMP2B**	chromatin modifying protein 2B	0,0168916	0,778	0,0064124	0,826	0,802
238877_at	**EYA4**	eyes absent homolog 4 (Drosophila)	0,0169173	0,599	0,0521833	0,425	0,512
225274_at	**SNRPG**	small nuclear ribonucleoprotein polypeptide G	0,0169364	0,724	0,0212349	0,576	0,650
202782_s_at	**SKIP**	NA	0,0169436	0,834	0,0241750	0,774	0,804
202032_s_at	**MAN2A2**	mannosidase, alpha, class 2A, member 2	0,0169519	0,804	0,0081458	0,839	0,822
232403_at	**FLJ21439**	NA	0,0169696	0,808	0,0346113	0,786	0,797
226361_at	**TMEM42**	transmembrane protein 42	0,0169696	0,725	0,0007902	0,641	0,683
219246_s_at	**FLJ13491**	NA	0,0169699	0,834	0,0122227	0,799	0,816
201906_s_at	**CTDSPL**	CTD (carboxy-terminal domain, RNA polymerase II, polypeptide A) small phosphatase-like	0,0169702	0,793	0,0438785	0,785	0,789
231914_at	**NUDT14**	nudix (nucleoside diphosphate linked moiety X)-type motif 14	0,0169811	0,717	0,0007599	0,633	0,675
218496_at	**RNASEH1**	ribonuclease H1	0,0170040	0,784	0,0261591	0,716	0,750
202219_at	**SLC6A8**	solute carrier family 6 (neurotransmitter transporter, creatine), member 8	0,0170040	0,691	0,0001761	0,595	0,643
205808_at	**ASPH**	aspartate beta-hydroxylase	0,0170368	0,492	0,0395334	0,776	0,634
221864_at	**MGC13024**	NA	0,0170368	0,693	0,0000000	0,592	0,643
241985_at	**JMY**	NA	0,0170478	0,765	0,0396253	0,830	0,798
241779_at	**MTX3**	metaxin 3	0,0170702	0,862	0,0592514	0,864	0,863
201718_s_at	**EPB41L2**	erythrocyte membrane protein band 4.1-like 2	0,0171048	0,644	0,0525980	0,779	0,711
222785_x_at	**C11orf1**	chromosome 11 open reading frame 1	0,0171521	0,685	0,0436930	0,688	0,686
221575_at	**SCLY**	selenocysteine lyase	0,0171521	0,810	0,0436930	0,794	0,802
203518_at	**LYST**	lysosomal trafficking regulator	0,0171564	0,551	0,0002160	0,577	0,564
226291_at	**ALS2**	amyotrophic lateral sclerosis 2 (juvenile)	0,0171569	0,756	0,0214687	0,733	0,744
208546_x_at	**HIST1H2BH**	histone 1, H2bh	0,0171569	0,641	0,0590558	0,777	0,709
212658_at	**LHFPL2**	lipoma HMGIC fusion partner-like 2	0,0171569	0,616	0,0295559	0,532	0,574
201462_at	**SCRN1**	secernin 1	0,0171569	0,684	0,0577081	0,719	0,701
231137_at	**ACSBG1**	acyl-CoA synthetase bubblegum family member 1	0,0171630	0,700	0,0002732	0,721	0,711
213940_s_at	**FNBP1**	formin binding protein 1	0,0172157	0,785	0,0401584	0,657	0,721
213979_s_at	**CTBP1**	C-terminal binding protein 1	0,0172204	0,660	0,0384205	0,838	0,749
209448_at	**HTATIP2**	HIV-1 Tat interactive protein 2, 30kDa	0,0172204	0,691	0,0557438	0,807	0,749
228231_at	**SNX8**	sorting nexin 8	0,0172474	0,755	0,0404837	0,806	0,781
217925_s_at	**C6orf106**	chromosome 6 open reading frame 106	0,0173421	0,828	0,0002114	0,704	0,766
234097_s_at	**C6orf12**	chromosome 6 open reading frame 12	0,0173421	0,908	0,0559555	0,887	0,898
213457_at	**MFHAS1**	malignant fibrous histiocytoma amplified sequence 1	0,0173518	0,684	0,0593866	0,696	0,690
217516_x_at	**ARVCF**	armadillo repeat gene deletes in velocardiofacial syndrome	0,0174134	0,834	0,0245205	0,779	0,807
232163_at	**WDR19**	WD repeat domain 19	0,0174195	0,869	0,0509055	0,878	0,873
201201_at	**CSTB**	cystatin B (stefin B)	0,0175303	0,796	0,0021875	0,737	0,766
239152_at	**IVNS1ABP**	influenza virus NS1A binding protein	0,0175303	0,888	0,0122043	0,798	0,843
201278_at	**DAB2**	disabled homolog 2, mitogen-responsive phosphoprotein (Drosophila)	0,0175318	0,818	0,0474086	0,705	0,762
201361_at	**MGC5508**	NA	0,0175742	0,874	0,0125615	0,821	0,847
204944_at	**PTPRG**	protein tyrosine phosphatase, receptor type, G	0,0175742	0,654	0,0137391	0,689	0,671
206510_at	**SIX2**	sine oculis homeobox homolog 2 (Drosophila)	0,0175742	0,548	0,0024092	0,528	0,538
211458_s_at	**GABARAPL3**	GABA(A) receptors associated protein like 3	0,0175830	0,657	0,0315573	0,624	0,641
231829_at	**KIAA1271**	NA	0,0176546	0,526	0,0012580	0,472	0,499
225320_at	**C10orf42**	chromosome 10 open reading frame 42	0,0176754	0,780	0,0019970	0,720	0,750
223171_at	**DYM**	dymeclin	0,0176866	0,760	0,0002160	0,538	0,649
213623_at	**KIF3A**	kinesin family member 3A	0,0177109	0,598	0,0112684	0,679	0,638
208818_s_at	**COMT**	catechol-O-methyltransferase	0,0177493	0,733	0,0002561	0,699	0,716

230903_s_at	**C8orf42**	chromosome 8 open reading frame 42	0,0177648	0,707	0,0381424	0,843	0,775
243111_at	**ENTPD1**	ectonucleoside triphosphate diphosphohydrolase 1	0,0177701	0,824	0,0219451	0,452	0,638
230224_at	**MCART6**	mitochondrial carrier triple repeat 6	0,0177701	0,661	0,0495051	0,809	0,735
218440_at	**MCCC1**	methylcrotonoyl-Coenzyme A carboxylase 1 (alpha)	0,0177701	0,707	0,0460710	0,709	0,708
204710_s_at	**WIPI-2**	NA	0,0177701	0,766	0,0194098	0,805	0,786
222804_x_at	**WDR32**	WD repeat domain 32	0,0177799	0,844	0,0053420	0,824	0,834
227112_at	**TMCC1**	transmembrane and coiled-coil domain family 1	0,0177837	0,736	0,0267816	0,653	0,694
221702_s_at	**TM2D3**	TM2 domain containing 3	0,0177876	0,796	0,0295559	0,799	0,797
239466_at	**LOC344595**	NA	0,0177932	0,615	0,0063430	0,591	0,603
219790_s_at	**NPR3**	natriuretic peptide receptor C/guanylate cyclase C (atrionatriuretic peptide receptor C)	0,0178337	0,588	0,0505090	0,612	0,600
202016_at	**MEST**	mesoderm specific transcript homolog (mouse)	0,0178614	0,657	0,0529437	0,584	0,620
223031_s_at	**TRAF7**	TNF receptor-associated factor 7	0,0178907	0,821	0,0199360	0,833	0,827
205608_s_at	**ANGPT1**	angiopoietin 1	0,0179072	0,340	0,0033315	0,891	0,616
210027_s_at	**APEX1**	APEX nuclease (multifunctional DNA repair enzyme) 1	0,0179164	0,740	0,0195858	0,776	0,758
231285_at	**SIMP**	NA	0,0179164	0,848	0,0038049	0,817	0,832
207281_x_at	**VCX2**	variable charge, X-linked 2	0,0179302	0,792	0,0441380	0,807	0,799
213357_at	**GTF2H5**	general transcription factor IIH, polypeptide 5	0,0179419	0,760	0,0239347	0,682	0,721
213057_at	**ATPAF2**	ATP synthase mitochondrial F1 complex assembly factor 2	0,0179455	0,866	0,0402356	0,844	0,855
215082_at	**ELOVL5**	ELOVL family member 5, elongation of long chain fatty acids (FEN1/Elo2, SUR4/Elo3-like, yeast)	0,0180018	0,807	0,0091656	0,837	0,822
207629_s_at	**ARHGEF2**	rho/rac guanine nucleotide exchange factor (GEF) 2	0,0180081	0,687	0,0347795	0,728	0,707
237484_at	**LOC440087**	NA	0,0180289	0,649	0,0384167	0,420	0,534
212337_at	**TUG1**	NA	0,0180380	0,809	0,0563892	0,830	0,820
1559681_a_at	**LOC147166**	NA	0,0180432	0,694	0,0432226	0,787	0,740
206338_at	**ELAVL3**	ELAV (embryonic lethal, abnormal vision, Drosophila)-like 3 (Hu antigen C)	0,0180573	0,791	0,0518200	0,908	0,849
204293_at	**SGSH**	N-sulfoglucosamine sulfohydrolase (sulfamidase)	0,0180573	0,709	0,0274801	0,586	0,648
200763_s_at	**RPLP1**	ribosomal protein, large, P1	0,0180877	0,946	0,0014977	0,941	0,943
224448_s_at	**C6orf125**	chromosome 6 open reading frame 125	0,0181124	0,831	0,0535882	0,694	0,762
203056_s_at	**PRDM2**	PR domain containing 2, with ZNF domain	0,0181124	0,741	0,0160984	0,748	0,745
204477_at	**RABIF**	RAB interacting factor	0,0181124	0,859	0,0486729	0,824	0,842
200909_s_at	**RPLP2**	ribosomal protein, large, P2	0,0181124	0,867	0,0343437	0,897	0,882
203943_at	**KIF3B**	kinesin family member 3B	0,0181568	0,851	0,0065496	0,844	0,848
212302_at	**RTF1**	Rtf1, Paf1/RNA polymerase II complex component, homolog (S. cerevisiae)	0,0181568	0,756	0,0223200	0,875	0,816
227823_at	**RGAG4**	retrotransposon gag domain containing 4	0,0182650	0,752	0,0337888	0,795	0,773
219061_s_at	**DXS9879E**	NA	0,0183355	0,656	0,0402238	0,727	0,692
244506_at	**ARG99**	NA	0,0184512	0,474	0,0222056	0,421	0,447
231640_at	**LOC144363**	NA	0,0184558	0,888	0,0343908	0,898	0,893
212057_at	**KIAA0182**	NA	0,0184607	0,672	0,0007794	0,607	0,639
201266_at	**TXNRD1**	thioredoxin reductase 1	0,0184607	0,633	0,0217142	0,682	0,657
229395_at	**STX4A**	syntaxin 4A (placental)	0,0184854	0,786	0,0408778	0,892	0,839
1552472_a_at	**CENTB2**	centaurin, beta 2	0,0185028	0,645	0,0485767	0,857	0,751
208579_x_at	**H2BFS**	H2B histone family, member S	0,0185450	0,566	0,0555798	0,717	0,641
209235_at	**CLCN7**	chloride channel 7	0,0185926	0,793	0,0181179	0,635	0,714
214920_at	**LOC221981**	NA	0,0185926	0,725	0,0195364	0,750	0,738
218419_s_at	**MGC3123**	NA	0,0186154	0,761	0,0000000	0,696	0,728
218466_at	**TBC1D17**	TBC1 domain family, member 17	0,0186165	0,758	0,0000000	0,629	0,693
212230_at	**PPAP2B**	phosphatidic acid phosphatase type 2B	0,0186181	0,659	0,0496191	0,657	0,658
205259_at	**NR3C2**	nuclear receptor subfamily 3, group C, member 2	0,0186326	0,556	0,0017801	0,483	0,520
226951_at	**MGC5509**	NA	0,0186494	0,747	0,0300706	0,818	0,783
220215_at	**ZNF669**	zinc finger protein 669	0,0186509	0,769	0,0137538	0,792	0,780
211086_x_at	**NEK1**	NIMA (never in mitosis gene a)-related kinase 1	0,0186664	0,563	0,0081170	0,576	0,570
204310_s_at	**NPR2**	natriuretic peptide receptor B/guanylate cyclase B (atrionatriuretic peptide receptor B)	0,0186777	0,756	0,0121227	0,679	0,718
225204_at	**TA-PP2C**	NA	0,0187674	0,774	0,0378012	0,807	0,791
213587_s_at	**ATP6V0E2L**	ATPase, H+ transporting V0 subunit E isoform 2-like (rat)	0,0187826	0,681	0,0020853	0,634	0,658
223499_at	**C1QTNF5**	C1q and tumor necrosis factor related protein 5	0,0187873	0,412	0,0014977	0,375	0,394
230347_at	**C7orf19**	chromosome 7 open reading frame 19	0,0187873	0,860	0,0574502	0,802	0,831
201301_s_at	**ANXA4**	annexin A4	0,0188067	0,753	0,0438346	0,815	0,784
200794_x_at	**DAZAP2**	DAZ associated protein 2	0,0188306	0,862	0,0020364	0,822	0,842
233650_at	**Cep63**	NA	0,0188373	0,836	0,0412757	0,689	0,762
1555653_at	**HNRPA3P1**	heterogeneous nuclear ribonucleoprotein A3 pseudogene 1	0,0188777	0,780	0,0017270	0,617	0,699

223415_at	**RPP25**	ribonuclease P 25kDa subunit	0,0188827	0,720	0,0241530	0,701	0,710
219250_s_at	**FLRT3**	fibronectin leucine rich transmembrane protein 3	0,0188971	0,405	0,0384483	0,607	0,506
226577_at	**PSEN1**	presenilin 1 (Alzheimer disease 3)	0,0188971	0,798	0,0265457	0,905	0,851
218477_at	**TMEM14A**	transmembrane protein 14A	0,0189059	0,858	0,0442591	0,864	0,861
216869_at	**PDE1C**	phosphodiesterase 1C, calmodulin-dependent 70kDa	0,0189081	0,557	0,0468395	0,745	0,651
221858_at	**TBC1D12**	TBC1 domain family, member 12	0,0189804	0,601	0,0338806	0,683	0,642
235775_at	**DKFZp762A217**	NA	0,0191455	0,715	0,0137538	0,817	0,766
212674_s_at	**DHX30**	DEAH (Asp-Glu-Ala-His) box polypeptide 30	0,0191664	0,846	0,0104296	0,754	0,800
232394_at	**KIAA0703**	NA	0,0192542	0,888	0,0413014	0,872	0,880
200833_s_at	**RAP1B**	RAP1B, member of RAS oncogene family	0,0192986	0,810	0,0308532	0,842	0,826
209577_at	**PCYT2**	phosphate cytidylyltransferase 2, ethanolamine	0,0193906	0,716	0,0000000	0,524	0,620
222477_s_at	**TM7SF3**	transmembrane 7 superfamily member 3	0,0193987	0,785	0,0597531	0,810	0,797
201471_s_at	**SQSTM1**	sequestosome 1	0,0194102	0,644	0,0322253	0,640	0,642
216296_at	**CLTA**	clathrin, light polypeptide (Lca)	0,0194845	0,876	0,0266313	0,847	0,862
223114_at	**MGC4767**	NA	0,0195192	0,802	0,0225927	0,820	0,811
235507_at	**LOC115294**	NA	0,0195690	0,671	0,0443796	0,612	0,642
213531_s_at	**ZRANB3**	zinc finger, RAN-binding domain containing 3	0,0196390	0,840	0,0385929	0,772	0,806
223098_s_at	**LONPL**	NA	0,0196652	0,753	0,0000000	0,708	0,731
226008_at	**NDNL2**	necdin-like 2	0,0197230	0,856	0,0204257	0,738	0,797
201106_at	**GPX4**	glutathione peroxidase 4 (phospholipid hydroperoxidase)	0,0197462	0,649	0,0040728	0,661	0,655
208527_x_at	**HIST1H2BE**	histone 1, H2be	0,0198259	0,604	0,0299884	0,730	0,667
224212_s_at	**PCDHA10**	protocadherin alpha 10	0,0198802	0,475	0,0208800	0,467	0,471
228080_at	**LOC143903**	NA	0,0199664	0,690	0,0526192	0,700	0,695
201463_s_at	**TALDO1**	transaldolase 1	0,0200231	0,825	0,0499447	0,863	0,844
241670_x_at	**LOC401237**	NA	0,0200381	0,789	0,0204257	0,874	0,831
209160_at	**AKR1C3**	aldo-keto reductase family 1, member C3 (3-alpha hydroxysteroid dehydrogenase, type II)	0,0200579	0,341	0,0102434	0,371	0,356
202669_s_at	**EFNB2**	ephrin-B2	0,0200579	0,505	0,0298015	0,437	0,471
234936_s_at	**KIAA1345**	NA	0,0200579	0,653	0,0337113	0,785	0,719
230221_at	**BAT5**	HLA-B associated transcript 5	0,0200854	0,797	0,0211349	0,803	0,800
226557_at	**TTC5**	tetratricopeptide repeat domain 5	0,0201499	0,873	0,0068660	0,791	0,832
230418_s_at	**GALNTL1**	UDP-N-acetyl-alpha-D-galactosamine:polypeptide N-acetylgalactosaminyltransferase-like 1	0,0201613	0,566	0,0003594	0,850	0,708
204866_at	**PHF16**	PHD finger protein 16	0,0202605	0,681	0,0567324	0,651	0,666
216928_at	**TAL1**	T-cell acute lymphocytic leukemia 1	0,0202959	0,876	0,0357973	0,771	0,823
213578_at	**BMPR1A**	bone morphogenetic protein receptor, type IA	0,0203039	0,801	0,0279723	0,830	0,815
221667_s_at	**HSPB8**	heat shock 22kDa protein 8	0,0203039	0,524	0,0334370	0,575	0,550
217963_s_at	**NGFRAP1**	nerve growth factor receptor (TNFRSF16) associated protein 1	0,0203039	0,850	0,0359085	0,838	0,844
223795_at	**TSPAN10**	tetraspanin 10	0,0203092	0,736	0,0117395	0,782	0,759
209917_s_at	**TP53AP1**	TP53 activated protein 1	0,0203501	0,613	0,0024485	0,555	0,584
225058_at	**GPR108**	G protein-coupled receptor 108	0,0203560	0,815	0,0021916	0,755	0,785
227790_at	**C6orf157**	chromosome 6 open reading frame 157	0,0203793	0,697	0,0417667	0,755	0,726
219834_at	**ALS2CR8**	amyotrophic lateral sclerosis 2 (juvenile) chromosome region, candidate 8	0,0203820	0,635	0,0378936	0,757	0,696
220608_s_at	**FLJ20582**	NA	0,0204065	0,873	0,0268447	0,892	0,883
1556175_at	**LOC92154**	NA	0,0204174	0,822	0,0000000	0,367	0,595
226448_at	**FAM89A**	family with sequence similarity 89, member A	0,0205247	0,472	0,0099196	0,441	0,456
229107_at	**LOC388227**	NA	0,0205247	0,671	0,0100446	0,762	0,717
235009_at	**FAM44A**	family with sequence similarity 44, member A	0,0206438	0,527	0,0325509	0,761	0,644
226543_at	**TXNDC5**	thioredoxin domain containing 5	0,0206529	0,645	0,0316563	0,878	0,762
223013_at	**TBL1XR1**	transducin (beta)-like 1X-linked receptor 1	0,0207198	0,708	0,0241340	0,727	0,717
227166_at	**DNAJC18**	DnaJ (Hsp40) homolog, subfamily C, member 18	0,0207606	0,705	0,0393484	0,622	0,664
213311_s_at	**KIAA1049**	NA	0,0207606	0,802	0,0436333	0,878	0,840
209390_at	**TSC1**	tuberous sclerosis 1	0,0207606	0,816	0,0089485	0,792	0,804
224812_at	**HIBADH**	3-hydroxyisobutyrate dehydrogenase	0,0208572	0,652	0,0532096	0,780	0,716
217971_at	**MAP2K1IP1**	mitogen-activated protein kinase kinase 1 interacting protein 1	0,0208572	0,782	0,0379730	0,835	0,809
210519_s_at	**NQO1**	NAD(P)H dehydrogenase, quinone 1	0,0208721	0,701	0,0502574	0,698	0,700
219926_at	**POPDC3**	popeye domain containing 3	0,0209106	0,619	0,0254511	0,628	0,623
205138_s_at	**UST**	uronyl-2-sulfotransferase	0,0209570	0,807	0,0193138	0,626	0,717
216345_at	**KIAA0913**	KIAA0913	0,0209732	0,777	0,0041220	0,737	0,757
210203_at	**CNOT4**	CCR4-NOT transcription complex, subunit 4	0,0210075	0,805	0,0007834	0,722	0,763
203567_s_at	**TRIM38**	tripartite motif-containing 38	0,0210603	0,845	0,0285976	0,742	0,794
218204_s_at	**FYCO1**	FYVE and coiled-coil domain containing 1	0,0211174	0,608	0,0396481	0,685	0,647
207034_s_at	**GLI2**	GLI-Kruppel family member GLI2	0,0211289	0,690	0,0485374	0,747	0,719
224882_at	**ACSS1**	acyl-CoA synthetase short-chain family member 1	0,0211405	0,800	0,0438351	0,830	0,815
229984_at	**DTWD1**	DTW domain containing 1	0,0211405	0,608	0,0471726	0,577	0,593
221751_at	**PANK3**	pantothenate kinase 3	0,0211405	0,779	0,0282125	0,730	0,755
223157_at	**C4orf14**	chromosome 4 open reading frame 14	0,0211835	0,733	0,0386231	0,804	0,769
238969_at	**LOC152078**	NA	0,0211835	0,404	0,0194743	0,427	0,415

214180_at	**MAN1C1**	mannosidase, alpha, class 1C, member 1	0,0211923	0,229	0,0015269	0,194	0,212
201561_s_at	**CLSTN1**	calsyntenin 1	0,0212265	0,741	0,0000000	0,651	0,696
214828_s_at	**dJ222E13.2**	NA	0,0212364	0,848	0,0242010	0,897	0,873
213333_at	**MDH2**	malate dehydrogenase 2, NAD (mitochondrial)	0,0212425	0,867	0,0051125	0,752	0,810
225817_at	**CGNL1**	cingulin-like 1	0,0212457	0,513	0,0115551	0,888	0,701
209102_s_at	**HBP1**	HMG-box transcription factor 1	0,0212481	0,770	0,0134247	0,757	0,764
41047_at	**C9orf16**	chromosome 9 open reading frame 16	0,0212606	0,769	0,0269073	0,668	0,719
218956_s_at	**PTCD1**	pentatricopeptide repeat domain 1	0,0213063	0,876	0,0173539	0,842	0,859
202053_s_at	**ALDH3A2**	aldehyde dehydrogenase 3 family, member A2	0,0213254	0,629	0,0301166	0,522	0,576
218521_s_at	**FLJ11011**	NA	0,0214085	0,686	0,0422245	0,879	0,783
214496_x_at	**MYST4**	MYST histone acetyltransferase (monocytic leukemia) 4	0,0214111	0,799	0,0137024	0,795	0,797
202916_s_at	**FAM20B**	family with sequence similarity 20, member B	0,0214122	0,783	0,0000000	0,637	0,710
221735_at	**WDR48**	WD repeat domain 48	0,0214178	0,796	0,0565204	0,845	0,821
211042_x_at	**MCAM**	melanoma cell adhesion molecule	0,0214600	0,786	0,0219563	0,273	0,529
205862_at	**GREB1**	NA	0,0214641	0,775	0,0155782	0,815	0,795
236219_at	**TMEM20**	transmembrane protein 20	0,0214641	0,779	0,0162783	0,796	0,788
203425_s_at	**IGFBP5**	insulin-like growth factor binding protein 5	0,0214797	0,538	0,0239475	0,402	0,470
217948_at	**DKFZP564B147**	NA	0,0215512	0,784	0,0039168	0,717	0,751
241933_at	**QRSL1**	glutaminyl-tRNA synthase (glutamine-hydrolyzing)-like 1	0,0216154	0,764	0,0053420	0,752	0,758
221689_s_at	**DSCR5**	Down syndrome critical region gene 5	0,0216828	0,702	0,0318109	0,692	0,697
229357_at	**ADAMTS5**	ADAM metallopeptidase with thrombospondin type 1 motif, 5 (aggrecanase-2)	0,0217127	0,539	0,0255380	0,428	0,484
229038_at	**CWF19L1**	CWF19-like 1, cell cycle control (S. pombe)	0,0217311	0,764	0,0114590	0,786	0,775
227091_at	**KIAA1505**	NA	0,0217311	0,732	0,0424395	0,768	0,750
201716_at	**SNX1**	sorting nexin 1	0,0217311	0,795	0,0116621	0,779	0,787
203222_s_at	**TLE1**	transducin-like enhancer of split 1 (E(sp1) homolog, Drosophila)	0,0217311	0,704	0,0540302	0,769	0,737
224815_at	**COMMD7**	COMM domain containing 7	0,0217803	0,752	0,0100848	0,844	0,798
209129_at	**TRIP6**	thyroid hormone receptor interactor 6	0,0218315	0,802	0,0000313	0,679	0,740
46270_at	**UBAP1**	ubiquitin associated protein 1	0,0218395	0,864	0,0267754	0,855	0,859
238013_at	**PLEKHA2**	pleckstrin homology domain containing, family A (phosphoinositide binding specific) member 2	0,0218708	0,452	0,0115013	0,529	0,491
226019_at	**OMA1**	OMA1 homolog, zinc metallopeptidase (S. cerevisiae)	0,0218738	0,644	0,0445308	0,719	0,682
210023_s_at	**PCGF1**	polycomb group ring finger 1	0,0218796	0,797	0,0230764	0,715	0,756
1561666_a_at	**KIAA1908**	NA	0,0219261	0,716	0,0154065	0,528	0,622
219023_at	**C4orf16**	chromosome 4 open reading frame 16	0,0219490	0,674	0,0121227	0,712	0,693
243167_at	**ABCB5**	ATP-binding cassette, sub-family B (MDR/TAP), member 5	0,0219727	0,821	0,0329235	0,731	0,776
226306_at	**C6orf1**	chromosome 6 open reading frame 1	0,0220082	0,695	0,0249995	0,649	0,672
221959_at	**C8orf72**	chromosome 8 open reading frame 72	0,0220082	0,477	0,0270315	0,365	0,421
211052_s_at	**TBCD**	tubulin-specific chaperone d	0,0220082	0,821	0,0141727	0,760	0,790
226738_at	**FLJ33817**	NA	0,0220107	0,782	0,0269719	0,800	0,791
208906_at	**BSCL2**	Bernardinelli-Seip congenital lipodystrophy 2 (seipin)	0,0220859	0,796	0,0128796	0,719	0,758
233919_s_at	**CDC14B**	CDC14 cell division cycle 14 homolog B (S. cerevisiae)	0,0221142	0,769	0,0221859	0,692	0,731
230693_at	**ATP2A1**	ATPase, Ca++ transporting, cardiac muscle, fast twitch 1	0,0221413	0,874	0,0378333	0,865	0,870
1557966_x_at	**MTERFD2**	MTERF domain containing 2	0,0221592	0,735	0,0390073	0,843	0,789
225270_at	**LOC388134**	NA	0,0221651	0,676	0,0054445	0,669	0,672
222662_at	**LOC286044**	NA	0,0222107	0,580	0,0153451	0,520	0,550
234918_at	**GLTSCR2**	glioma tumor suppressor candidate region gene 2	0,0223009	0,865	0,0477154	0,702	0,783
229719_s_at	**DERL3**	Der1-like domain family, member 3	0,0223142	0,780	0,0408663	0,795	0,787
1561963_at	**CTBP2**	C-terminal binding protein 2	0,0223743	0,894	0,0075353	0,719	0,806
1555787_at	**FLJ23554**	NA	0,0223966	0,860	0,0578490	0,775	0,817
244226_s_at	**FLJ20315**	NA	0,0223981	0,888	0,0529535	0,859	0,874
208709_s_at	**NRD1**	nardilysin (N-arginine dibasic convertase)	0,0224375	0,851	0,0245243	0,880	0,866
241902_at	**C10orf48**	chromosome 10 open reading frame 48	0,0224385	0,319	0,0300607	0,397	0,358
203334_at	**DHX8**	DEAH (Asp-Glu-Ala-His) box polypeptide 8	0,0224486	0,862	0,0280375	0,794	0,828
212069_s_at	**KIAA0515**	KIAA0515	0,0225146	0,795	0,0545850	0,791	0,793
219525_at	**FLJ10847**	NA	0,0225891	0,572	0,0463234	0,534	0,553
228048_at	**C10orf41**	chromosome 10 open reading frame 41	0,0226018	0,828	0,0211930	0,852	0,840
229504_at	**RAB23**	RAB23, member RAS oncogene family	0,0226291	0,648	0,0332692	0,717	0,683
209322_s_at	**SH2B**	NA	0,0226577	0,848	0,0092381	0,791	0,819
203215_s_at	**MYO6**	myosin VI	0,0227228	0,671	0,0237590	0,813	0,742
213568_at	**OSR2**	odd-skipped related 2 (Drosophila)	0,0227357	0,177	0,0238263	0,161	0,169
225948_at	**C14orf153**	chromosome 14 open reading frame 153	0,0227531	0,784	0,0207754	0,868	0,826
203503_s_at	**PEX14**	peroxisomal biogenesis factor 14	0,0227694	0,817	0,0389738	0,891	0,854
233618_at	**LOC439948**	NA	0,0227760	0,851	0,0368221	0,849	0,850
226959_at	**LOC283070**	NA	0,0227975	0,779	0,0418108	0,745	0,762

34697_at	**LRP6**	low density lipoprotein receptor-related protein 6	0,0228012	0,785	0,0196637	0,767	0,776
231727_s_at	**AD023**	NA	0,0228258	0,726	0,0148603	0,587	0,656
221954_at	**C20orf111**	chromosome 20 open reading frame 111	0,0228258	0,803	0,0012787	0,703	0,753
209730_at	**SEMA3F**	sema domain, immunoglobulin domain (Ig), short basic domain, secreted, (semaphorin) 3F	0,0228877	0,748	0,0084725	0,752	0,750
236678_at	**JAG1**	jagged 1 (Alagille syndrome)	0,0229072	0,764	0,0522994	0,814	0,789
1554004_a_at	**RGNEF**	NA	0,0229542	0,806	0,0437677	0,737	0,772
226637_at	**UBE2H**	ubiquitin-conjugating enzyme E2H (UBC8 homolog, yeast)	0,0229542	0,669	0,0373662	0,695	0,682
242065_x_at	**IDI1**	isopentenyl-diphosphate delta isomerase 1	0,0230083	0,752	0,0012460	0,483	0,617
210674_s_at	**PCDHA12**	protocadherin alpha 12	0,0230083	0,444	0,0436333	0,451	0,447
222895_s_at	**BCL11B**	B-cell CLL/lymphoma 11B (zinc finger protein)	0,0230138	0,538	0,0437677	0,703	0,621
206858_s_at	**HOXC6**	homeo box C6	0,0230377	0,795	0,0200268	0,731	0,763
203318_s_at	**ZNF148**	zinc finger protein 148 (pHZ-52)	0,0230400	0,768	0,0299632	0,760	0,764
202047_s_at	**CBX6**	chromobox homolog 6	0,0231042	0,819	0,0422790	0,873	0,846
223059_s_at	**C10orf45**	chromosome 10 open reading frame 45	0,0231372	0,560	0,0551963	0,648	0,604
209120_at	**NR2F2**	nuclear receptor subfamily 2, group F, member 2	0,0231372	0,581	0,0312167	0,445	0,513
213221_s_at	**SNF1LK2**	SNF1-like kinase 2	0,0231372	0,561	0,0035379	0,525	0,543
210532_s_at	**C14orf2**	chromosome 14 open reading frame 2	0,0231586	0,905	0,0306477	0,761	0,833
224512_s_at	**MGC14151**	NA	0,0232619	0,831	0,0325273	0,830	0,830
201022_s_at	**DSTN**	destrin (actin depolymerizing factor)	0,0233967	0,911	0,0414331	0,554	0,733
221221_s_at	**KLHL3**	kelch-like 3 (Drosophila)	0,0234371	0,565	0,0234494	0,550	0,558
206833_s_at	**ACYP2**	acylphosphatase 2, muscle type	0,0234463	0,758	0,0443189	0,765	0,761
230586_s_at	**ZNF703**	zinc finger protein 703	0,0234509	0,816	0,0263081	0,642	0,729
219654_at	**PTPLA**	protein tyrosine phosphatase-like (proline instead of catalytic arginine), member a	0,0235058	0,686	0,0000517	0,612	0,649
206039_at	**RAB33A**	RAB33A, member RAS oncogene family	0,0235142	0,285	0,0043796	0,231	0,258
219609_at	**MGC4645**	NA	0,0235157	0,804	0,0534777	0,843	0,824
234974_at	**GALM**	galactose mutarotase (aldose 1-epimerase)	0,0235807	0,725	0,0109057	0,591	0,658
38340_at	**HIP1R**	NA	0,0236352	0,784	0,0236544	0,617	0,701
209276_s_at	**GLRX**	glutaredoxin (thioltransferase)	0,0236576	0,611	0,0477787	0,759	0,685
219379_x_at	**ZNF358**	zinc finger protein 358	0,0236576	0,779	0,0001433	0,675	0,727
201339_s_at	**SCP2**	sterol carrier protein 2	0,0237135	0,808	0,0382085	0,906	0,857
227559_at	**C6orf66**	chromosome 6 open reading frame 66	0,0237384	0,714	0,0386620	0,765	0,740
219123_at	**ZNF232**	zinc finger protein 232	0,0237384	0,787	0,0549333	0,859	0,823
1558782_a_at	**NFYC**	nuclear transcription factor Y, gamma	0,0237690	0,838	0,0019074	0,752	0,795
222918_at	**RAB9B**	RAB9B, member RAS oncogene family	0,0237880	0,770	0,0506630	0,794	0,782
203139_at	**DAPK1**	death-associated protein kinase 1	0,0238316	0,642	0,0086993	0,539	0,591
223692_at	**NMNAT1**	nicotinamide nucleotide adenylyltransferase 1	0,0238474	0,635	0,0001227	0,597	0,616
212428_at	**KIAA0368**	KIAA0368	0,0238519	0,812	0,0434927	0,688	0,750
235625_at	**VPS41**	vacuolar protein sorting 41 (yeast)	0,0238519	0,676	0,0044184	0,590	0,633
226723_at	**CCDC23**	coiled-coil domain containing 23	0,0238592	0,776	0,0224733	0,671	0,724
228917_at	**ZNF510**	zinc finger protein 510	0,0238592	0,799	0,0539950	0,813	0,806
202723_s_at	**FOXO1A**	forkhead box O1A (rhabdomyosarcoma)	0,0238794	0,654	0,0200268	0,891	0,772
212584_at	**AQR**	aquarius homolog (mouse)	0,0238982	0,878	0,0282191	0,848	0,863
204058_at	**ME1**	malic enzyme 1, NADP(+)-dependent, cytosolic	0,0239029	0,584	0,0034872	0,527	0,555
201844_s_at	**RYBP**	RING1 and YY1 binding protein	0,0240129	0,717	0,0204257	0,744	0,730
216831_s_at	**RUNX1T1**	c("runt-related transcription factor 1", " translocated to, 1 (cyclin D-related)")	0,0240142	0,694	0,0373005	0,637	0,665
227473_at	**CTTN**	cortactin	0,0240261	0,677	0,0253288	0,870	0,773
224859_at	**CD276**	CD276 antigen	0,0241326	0,715	0,0004401	0,658	0,687
230625_s_at	**TSPAN12**	tetraspanin 12	0,0241326	0,807	0,0469624	0,647	0,727
213482_at	**DOCK3**	dedicator of cytokinesis 3	0,0241598	0,742	0,0222913	0,609	0,676
204841_s_at	**EEA1**	early endosome antigen 1, 162kD	0,0241752	0,686	0,0026329	0,557	0,621
225800_at	**JAZF1**	NA	0,0241752	0,663	0,0387449	0,712	0,687
205111_s_at	**PLCE1**	phospholipase C, epsilon 1	0,0241752	0,622	0,0082989	0,517	0,569
201500_s_at	**PPP1R11**	protein phosphatase 1, regulatory (inhibitor) subunit 11	0,0241752	0,896	0,0044944	0,707	0,802
212957_s_at	**LOC92249**	NA	0,0242750	0,736	0,0027548	0,701	0,718
1569114_at	**TRIM37**	tripartite motif-containing 37	0,0242750	0,880	0,0118471	0,778	0,829
212161_at	**AP2A2**	adaptor-related protein complex 2, alpha 2 subunit	0,0243194	0,853	0,0060287	0,774	0,813
223390_at	**C9orf37**	chromosome 9 open reading frame 37	0,0244354	0,791	0,0100348	0,794	0,793
225624_at	**LOC92017**	NA	0,0244776	0,838	0,0072859	0,798	0,818
233679_at	**MAP3K7IP1**	mitogen-activated protein kinase kinase kinase 7 interacting protein 1	0,0244969	0,898	0,0018798	0,804	0,851
204720_s_at	**DNAJC6**	DnaJ (Hsp40) homolog, subfamily C, member 6	0,0245368	0,502	0,0006416	0,560	0,531
228950_s_at	**C1orf139**	chromosome 1 open reading frame 139	0,0245860	0,635	0,0397743	0,787	0,711
243065_at	**PS1TP4**	NA	0,0245860	0,668	0,0004401	0,485	0,576
219327_s_at	**GPRC5C**	G protein-coupled receptor, family C, group 5, member C	0,0245977	0,707	0,0222937	0,658	0,683
226541_at	**FBXO30**	F-box protein 30	0,0246451	0,750	0,0306477	0,823	0,786

229417_at	**STAU2**	staufen, RNA binding protein, homolog 2 (Drosophila)	0,0247093	0,767	0,0065321	0,733	0,750
229408_at	**HDAC5**	histone deacetylase 5	0,0247393	0,816	0,0022951	0,597	0,707
223479_s_at	**CHCHD5**	coiled-coil-helix-coiled-coil-helix domain containing 5	0,0247573	0,801	0,0054445	0,799	0,800
64883_at	**MOSPD2**	motile sperm domain containing 2	0,0247904	0,734	0,0007794	0,642	0,688
225442_at	**DDR2**	discoidin domain receptor family, member 2	0,0248298	0,693	0,0068874	0,638	0,665
213082_s_at	**SLC35D2**	solute carrier family 35, member D2	0,0248298	0,828	0,0025657	0,737	0,782
203031_s_at	**UROS**	uroporphyrinogen III synthase (congenital erythropoietic porphyria)	0,0248323	0,762	0,0016655	0,732	0,747
238628_s_at	**LOC390748**	NA	0,0250361	0,832	0,0403689	0,768	0,800
225804_at	**MGC32124**	NA	0,0250730	0,742	0,0432534	0,699	0,720
223469_at	**MGC10812**	NA	0,0251559	0,731	0,0000517	0,581	0,656
202427_s_at	**BRP44**	brain protein 44	0,0251958	0,727	0,0253444	0,771	0,749
220268_at	**PTD015**	NA	0,0252143	0,861	0,0311742	0,820	0,840
212490_at	**DNAJC8**	DnaJ (Hsp40) homolog, subfamily C, member 8	0,0252500	0,763	0,0166193	0,836	0,799
52975_at	**C9orf28**	chromosome 9 open reading frame 28	0,0252987	0,854	0,0252659	0,856	0,855
228249_at	**LOC119710**	NA	0,0252987	0,697	0,0335828	0,747	0,722
228638_at	**FAM76A**	family with sequence similarity 76, member A	0,0253201	0,626	0,0113755	0,686	0,656
203054_s_at	**TCTA**	T-cell leukemia translocation altered gene	0,0253297	0,809	0,0066153	0,722	0,766
202284_s_at	**CDKN1A**	cyclin-dependent kinase inhibitor 1A (p21, Cip1)	0,0253583	0,775	0,0004401	0,662	0,718
230257_s_at	**C1orf19**	chromosome 1 open reading frame 19	0,0253641	0,747	0,0230644	0,822	0,784
210778_s_at	**MXD4**	MAX dimerization protein 4	0,0253956	0,750	0,0135495	0,729	0,739
224880_at	**RALA**	v-ral simian leukemia viral oncogene homolog A (ras related)	0,0253956	0,829	0,0427636	0,837	0,833
213437_at	**LOC441022**	NA	0,0254346	0,689	0,0343027	0,609	0,649
222413_s_at	**MLL3**	myeloid/lymphoid or mixed-lineage leukemia 3	0,0254811	0,619	0,0524628	0,896	0,757
201959_s_at	**MYCBP2**	MYC binding protein 2	0,0254811	0,627	0,0204257	0,603	0,615
201246_s_at	**OTUB1**	OTU domain, ubiquitin aldehyde binding 1	0,0254811	0,853	0,0233628	0,774	0,813
220176_at	**NUBPL**	nucleotide binding protein-like	0,0255370	0,757	0,0228044	0,798	0,778
201722_s_at	**GALNT1**	UDP-N-acetyl-alpha-D-galactosamine:polypeptide N-acetylgalactosaminyltransferase 1 (GalNAc-T1)	0,0255525	0,819	0,0002160	0,586	0,702
202616_s_at	**MECP2**	methyl CpG binding protein 2 (Rett syndrome)	0,0255884	0,842	0,0017055	0,736	0,789
225794_s_at	**LOC91689**	NA	0,0256583	0,781	0,0397336	0,876	0,828
236530_at	**HS1BP3**	NA	0,0257985	0,844	0,0081851	0,792	0,818
209254_at	**KIAA0265**	NA	0,0257985	0,688	0,0393277	0,814	0,751
212823_s_at	**PLEKHG3**	pleckstrin homology domain containing, family G (with RhoGef domain) member 3	0,0258014	0,841	0,0248596	0,727	0,784
1562953_s_at	**C4orf12**	chromosome 4 open reading frame 12	0,0258762	0,645	0,0118503	0,444	0,545
226451_at	**MGC19604**	NA	0,0259237	0,812	0,0008754	0,752	0,782
228775_at	**LOC55831**	NA	0,0259339	0,835	0,0501955	0,849	0,842
202161_at	**PKN1**	protein kinase N1	0,0259712	0,844	0,0028212	0,592	0,718
204381_at	**LRP3**	low density lipoprotein receptor-related protein 3	0,0259895	0,802	0,0547085	0,840	0,821
215029_at	**C1orf108**	chromosome 1 open reading frame 108	0,0260651	0,644	0,0003716	0,605	0,625
227954_at	**LOC162073**	NA	0,0260651	0,769	0,0376072	0,783	0,776
223435_s_at	**PCDHA6**	protocadherin alpha 6	0,0260712	0,468	0,0422978	0,887	0,678
242726_at	**ACSL3**	acyl-CoA synthetase long-chain family member 3	0,0261257	0,895	0,0485255	0,734	0,815
229115_at	**DNCH1**	dynein, cytoplasmic, heavy polypeptide 1	0,0261257	0,668	0,0506283	0,845	0,756
226233_at	**B3GALNT2**	UDP-GalNAc:betaGlcNAc beta 1,3-galactosaminyltransferase, polypeptide 2	0,0261534	0,766	0,0273914	0,807	0,786
202017_at	**EPHX1**	epoxide hydrolase 1, microsomal (xenobiotic)	0,0261756	0,471	0,0014814	0,345	0,408
201429_s_at	**RPL37A**	ribosomal protein L37a	0,0261971	0,952	0,0326234	0,595	0,774
213405_at	**RAB22A**	RAB22A, member RAS oncogene family	0,0262466	0,803	0,0497770	0,861	0,832
244834_at	**C1orf89**	chromosome 1 open reading frame 89	0,0262873	0,775	0,0270732	0,788	0,781
205745_x_at	**ADAM17**	ADAM metallopeptidase domain 17 (tumor necrosis factor, alpha, converting enzyme)	0,0262989	0,806	0,0424836	0,694	0,750
217865_at	**RNF130**	ring finger protein 130	0,0264169	0,688	0,0035274	0,627	0,658
223219_s_at	**CNOT10**	CCR4-NOT transcription complex, subunit 10	0,0264196	0,766	0,0488646	0,640	0,703
205881_at	**ZNF74**	zinc finger protein 74 (Cos52)	0,0264322	0,717	0,0015575	0,730	0,724
224946_s_at	**MGC12981**	NA	0,0264671	0,697	0,0018613	0,685	0,691
224716_at	**SLC35B2**	solute carrier family 35, member B2	0,0264672	0,840	0,0000313	0,690	0,765
225877_at	**TYSND1**	trypsin domain containing 1	0,0264672	0,702	0,0005337	0,736	0,719
209885_at	**RHOD**	ras homolog gene family, member D	0,0265166	0,705	0,0545438	0,793	0,749
225346_at	**MTERFD3**	MTERF domain containing 3	0,0266992	0,621	0,0382123	0,565	0,593
203572_s_at	**TAF6**	TAF6 RNA polymerase II, TATA box binding protein (TBP)-associated factor, 80kDa	0,0267208	0,834	0,0124186	0,739	0,786
226830_x_at	**LOC440309**	NA	0,0267853	0,819	0,0442911	0,843	0,831
222453_at	**CYBRD1**	cytochrome b reductase 1	0,0269467	0,653	0,0137806	0,684	0,668
236234_at	**PDE1A**	phosphodiesterase 1A, calmodulin-dependent	0,0270246	0,538	0,0349218	0,250	0,394
1554575_a_at	**BPNT1**	3'(2'), 5'-bisphosphate nucleotidase 1	0,0270666	0,833	0,0047472	0,696	0,765

225359_at	**DNAJC19**	DnaJ (Hsp40) homolog, subfamily C, member 19	0,0270666	0,803	0,0283592	0,822	0,812
228161_at	**RAB32**	RAB32, member RAS oncogene family	0,0270666	0,821	0,0005607	0,628	0,725
224564_s_at	**RTN3**	reticulon 3	0,0270789	0,840	0,0137248	0,681	0,760
221830_at	**RAP2A**	RAP2A, member of RAS oncogene family	0,0271400	0,441	0,0237613	0,520	0,480
205811_at	**POLG2**	polymerase (DNA directed), gamma 2, accessory subunit	0,0272018	0,799	0,0319002	0,795	0,797
210421_s_at	**SLC24A1**	solute carrier family 24 (sodium/potassium/calcium exchanger), member 1	0,0272964	0,796	0,0559648	0,920	0,858
201854_s_at	**ASCIZ**	NA	0,0273070	0,844	0,0373499	0,887	0,865
238526_at	**RAB3IP**	RAB3A interacting protein (rabin3)	0,0273522	0,874	0,0502198	0,705	0,789
202594_at	**LEPROTL1**	leptin receptor overlapping transcript-like 1	0,0274427	0,831	0,0091816	0,808	0,819
201935_s_at	**EIF4G3**	eukaryotic translation initiation factor 4 gamma, 3	0,0274803	0,758	0,0253542	0,737	0,748
224901_at	**SCD5**	stearoyl-CoA desaturase 5	0,0274898	0,574	0,0557026	0,590	0,582
209599_s_at	**PRUNE**	prune homolog (Drosophila)	0,0275077	0,741	0,0017413	0,776	0,758
218624_s_at	**MGC2752**	NA	0,0275454	0,820	0,0019074	0,692	0,756
236158_at	**LOC147975**	NA	0,0276367	0,872	0,0054928	0,780	0,826
229888_at	**MGC47869**	NA	0,0276375	0,776	0,0414180	0,718	0,747
224742_at	**C20orf22**	chromosome 20 open reading frame 22	0,0276391	0,841	0,0499582	0,757	0,799
228398_at	**SENP8**	SUMO/sentrin specific peptidase family member 8	0,0276589	0,748	0,0487607	0,892	0,820
241489_at	**NUDT9**	nudix (nucleoside diphosphate linked moiety X)-type motif 9	0,0277454	0,883	0,0182585	0,863	0,873
200736_s_at	**GPX1**	glutathione peroxidase 1	0,0277599	0,623	0,0150247	0,706	0,665
205540_s_at	**RRAGB**	Ras-related GTP binding B	0,0277868	0,686	0,0040787	0,619	0,652
221016_s_at	**TCF7L1**	transcription factor 7-like 1 (T-cell specific, HMG-box)	0,0277868	0,702	0,0021912	0,494	0,598
217886_at	**EPS15**	epidermal growth factor receptor pathway substrate 15	0,0277931	0,797	0,0286674	0,758	0,777
223505_s_at	**RBJ**	NA	0,0277931	0,822	0,0009396	0,799	0,811
204359_at	**FLRT2**	fibronectin leucine rich transmembrane protein 2	0,0278027	0,568	0,0191443	0,437	0,502
230200_at	**NSUN6**	NOL1/NOP2/Sun domain family, member 6	0,0278110	0,818	0,0417091	0,902	0,860
223848_at	**CRBN**	cereblon	0,0278179	0,921	0,0052207	0,691	0,806
221577_x_at	**GDF15**	growth differentiation factor 15	0,0278365	0,526	0,0064069	0,380	0,453
218912_at	**GCC1**	GRIP and coiled-coil domain containing 1	0,0278594	0,841	0,0006153	0,795	0,818
229919_at	**PCNXL2**	pecanex-like 2 (Drosophila)	0,0278906	0,774	0,0204257	0,517	0,645
215045_at	**TNRC4**	trinucleotide repeat containing 4	0,0279208	0,860	0,0584362	0,846	0,853
223109_at	**TRUB2**	TruB pseudouridine (psi) synthase homolog 2 (E. coli)	0,0279512	0,808	0,0556068	0,890	0,849
51228_at	**LOC389677**	NA	0,0280960	0,751	0,0468461	0,920	0,835
207231_at	**DZIP3**	NA	0,0281522	0,698	0,0145964	0,709	0,703
213289_at	**SATL1**	spermidine/spermine N1-acetyl transferase-like 1	0,0282202	0,717	0,0254127	0,779	0,748
212421_at	**C22orf9**	chromosome 22 open reading frame 9	0,0283057	0,756	0,0002160	0,635	0,696
204262_s_at	**PSEN2**	presenilin 2 (Alzheimer disease 4)	0,0283057	0,807	0,0163183	0,810	0,809
213565_s_at	**SMAD6**	SMAD, mothers against DPP homolog 6 (Drosophila)	0,0283369	0,835	0,0564828	0,638	0,736
222407_s_at	**ZFP106**	zinc finger protein 106 homolog (mouse)	0,0283449	0,572	0,0287868	0,619	0,595
202119_s_at	**CPNE3**	copine III	0,0283688	0,834	0,0436930	0,784	0,809
221065_s_at	**CHST8**	carbohydrate (N-acetylgalactosamine 4-0) sulfotransferase 8	0,0283846	0,877	0,0212349	0,891	0,884
238344_at	**COPG2**	coatomer protein complex, subunit gamma 2	0,0283910	0,831	0,0101719	0,855	0,843
212946_at	**KIAA0564**	NA	0,0284211	0,750	0,0464426	0,804	0,777
228820_at	**LOC63929**	NA	0,0284211	0,825	0,0002773	0,679	0,752
202417_at	**KEAP1**	kelch-like ECH-associated protein 1	0,0284783	0,804	0,0137966	0,815	0,810
228493_at	**AKAP14**	A kinase (PRKA) anchor protein 14	0,0285341	0,660	0,0280504	0,836	0,748
202704_at	**TOB1**	transducer of ERBB2, 1	0,0285475	0,785	0,0428724	0,657	0,721
218901_at	**PLSCR4**	phospholipid scramblase 4	0,0285502	0,563	0,0311100	0,538	0,551
222956_at	**FIGN**	fidgetin	0,0285530	0,859	0,0505839	0,657	0,758
204066_s_at	**CENTG2**	centaurin, gamma 2	0,0285584	0,774	0,0032613	0,682	0,728
225391_at	**LOC93622**	NA	0,0285632	0,793	0,0247628	0,777	0,785
206557_at	**ZNF702**	zinc finger protein 702	0,0285632	0,660	0,0100678	0,657	0,659
232341_x_at	**HABP4**	hyaluronan binding protein 4	0,0285677	0,768	0,0266313	0,636	0,702
213026_at	**ATG12**	ATG12 autophagy related 12 homolog (S. cerevisiae)	0,0286354	0,727	0,0244185	0,885	0,806
200666_s_at	**DNAJB1**	DnaJ (Hsp40) homolog, subfamily B, member 1	0,0286388	0,790	0,0002256	0,688	0,739
214670_at	**ZKSCAN1**	zinc finger with KRAB and SCAN domains 1	0,0286388	0,804	0,0230671	0,728	0,766
237262_at	**FAM59A**	family with sequence similarity 59, member A	0,0286915	0,736	0,0584660	0,651	0,693
218153_at	**FLJ12118**	NA	0,0287407	0,730	0,0083280	0,795	0,762
227862_at	**LOC388610**	NA	0,0287407	0,647	0,0174500	0,854	0,751
1554447_at	**LOC554203**	NA	0,0287407	0,744	0,0137538	0,687	0,716
204211_x_at	**EIF2AK2**	eukaryotic translation initiation factor 2-alpha kinase 2	0,0287549	0,808	0,0137966	0,839	0,823
227546_x_at	**AURKAIP1**	aurora kinase A interacting protein 1	0,0288157	0,792	0,0193949	0,752	0,772

218398_at	**MRPS30**	mitochondrial ribosomal protein S30	0,0288563	0,839	0,0078245	0,727	0,783
205635_at	**KALRN**	kalirin, RhoGEF kinase	0,0288623	0,829	0,0437157	0,762	0,795
212796_s_at	**KIAA1055**	NA	0,0288623	0,550	0,0009353	0,544	0,547
211971_s_at	**LRPPRC**	leucine-rich PPR-motif containing	0,0288623	0,818	0,0053093	0,799	0,809
205652_s_at	**TTLL1**	tubulin tyrosine ligase-like family, member 1	0,0288805	0,820	0,0072601	0,759	0,789
203206_at	**FAM53B**	family with sequence similarity 53, member B	0,0289459	0,878	0,0538731	0,895	0,886
225695_at	**C2orf18**	chromosome 2 open reading frame 18	0,0289525	0,833	0,0396536	0,672	0,752
242415_at	**KIAA1545**	NA	0,0289961	0,833	0,0060299	0,743	0,788
212318_at	**TNPO3**	transportin 3	0,0290203	0,844	0,0071483	0,870	0,857
227983_at	**MGC7036**	NA	0,0290234	0,853	0,0545279	0,798	0,825
218234_at	**ING4**	inhibitor of growth family, member 4	0,0290742	0,802	0,0461615	0,830	0,816
214634_at	**HIST1H4I**	histone 1, H4i	0,0290965	0,896	0,0213898	0,897	0,897
225228_at	**MGC54289**	NA	0,0291278	0,779	0,0024963	0,691	0,735
211299_s_at	**FLOT2**	flotillin 2	0,0292103	0,795	0,0016785	0,632	0,714
222831_at	**SAP30L**	NA	0,0292349	0,728	0,0131810	0,720	0,724
217122_s_at	**SLC35E2**	solute carrier family 35, member E2	0,0292567	0,686	0,0021152	0,601	0,644
225987_at	**STEAP4**	STEAP family member 4	0,0293485	0,121	0,0237613	0,346	0,234
213222_at	**PLCB1**	phospholipase C, beta 1 (phosphoinositide-specific)	0,0293688	0,471	0,0387449	0,517	0,494
209343_at	**EFHD1**	EF-hand domain family, member D1	0,0293888	0,493	0,0566663	0,578	0,536
210740_s_at	**ITPK1**	inositol 1,3,4-triphosphate 5/6 kinase	0,0294187	0,799	0,0218822	0,689	0,744
228243_at	**PAXIP1**	PAX interacting (with transcription-activation domain) protein 1	0,0294272	0,667	0,0126580	0,573	0,620
1560006_a_at	**LOC442522**	NA	0,0294532	0,897	0,0295174	0,610	0,754
236565_s_at	**LARP6**	La ribonucleoprotein domain family, member 6	0,0294930	0,764	0,0065698	0,656	0,710
238051_x_at	**PWWP2**	PWWP domain containing 2	0,0295033	0,846	0,0029678	0,719	0,783
239084_at	**SNAP29**	synaptosomal-associated protein, 29kDa	0,0295047	0,747	0,0125800	0,520	0,634
217967_s_at	**C1orf24**	chromosome 1 open reading frame 24	0,0295213	0,484	0,0596927	0,501	0,492
202764_at	**STIM1**	stromal interaction molecule 1	0,0295730	0,691	0,0005407	0,589	0,640
241944_x_at	**TNFRSF1A**	tumor necrosis factor receptor superfamily, member 1A	0,0296463	0,814	0,0043096	0,723	0,769
218734_at	**FLJ13848**	NA	0,0296681	0,806	0,0022643	0,736	0,771
224809_x_at	**TINF2**	TERF1 (TRF1)-interacting nuclear factor 2	0,0296715	0,832	0,0103462	0,864	0,848
212582_at	**OSBPL8**	oxysterol binding protein-like 8	0,0296960	0,765	0,0163183	0,677	0,721
221552_at	**ABHD6**	abhydrolase domain containing 6	0,0297351	0,777	0,0055320	0,731	0,754
1556667_at	**LOC348751**	NA	0,0299234	0,749	0,0418991	0,740	0,745
213464_at	**SHC2**	SHC (Src homology 2 domain containing) transforming protein 2	0,0299387	0,688	0,0371740	0,707	0,698
202529_at	**PRPSAP1**	phosphoribosyl pyrophosphate synthetase-associated protein 1	0,0299451	0,781	0,0124625	0,782	0,782
208869_s_at	**GABARAPL1**	GABA(A) receptor-associated protein like 1	0,0299778	0,674	0,0530484	0,657	0,665
208407_s_at	**CTNND1**	catenin (cadherin-associated protein), delta 1	0,0300001	0,810	0,0395427	0,775	0,793
225202_at	**RHOBTB3**	Rho-related BTB domain containing 3	0,0303600	0,585	0,0025605	0,435	0,510
202151_s_at	**UBADC1**	ubiquitin associated domain containing 1	0,0303648	0,826	0,0006153	0,654	0,740
235435_at	**AASDH**	NA	0,0303966	0,630	0,0062077	0,661	0,646
232345_at	**C18orf8**	chromosome 18 open reading frame 8	0,0303966	0,840	0,0550224	0,859	0,850
217936_at	**ARHGAP5**	Rho GTPase activating protein 5	0,0304352	0,764	0,0058575	0,708	0,736
229001_at	**KIAA1443**	KIAA1443	0,0304618	0,767	0,0358641	0,788	0,778
213296_at	**PEX10**	peroxisome biogenesis factor 10	0,0304618	0,855	0,0196944	0,851	0,853
239210_at	**PBX1**	pre-B-cell leukemia transcription factor 1	0,0305628	0,841	0,0339505	0,656	0,748
223475_at	**CRISPLD1**	cysteine-rich secretory protein LCCL domain containing 1	0,0305674	0,423	0,0090387	0,245	0,334
1552474_a_at	**GAMT**	guanidinoacetate N-methyltransferase	0,0305806	0,789	0,0007092	0,674	0,731
212908_at	**DNAJC16**	DnaJ (Hsp40) homolog, subfamily C, member 16	0,0305892	0,739	0,0078594	0,767	0,753
209979_at	**ADARB1**	adenosine deaminase, RNA-specific, B1 (RED1 homolog rat)	0,0305952	0,833	0,0345347	0,822	0,828
206866_at	**CDH4**	cadherin 4, type 1, R-cadherin (retinal)	0,0305975	0,695	0,0522947	0,673	0,684
204985_s_at	**MGC2650**	NA	0,0306096	0,650	0,0091656	0,603	0,627
223993_s_at	**CNIH4**	cornichon homolog 4 (Drosophila)	0,0306130	0,875	0,0582163	0,853	0,864
40255_at	**DDX28**	DEAD (Asp-Glu-Ala-Asp) box polypeptide 28	0,0306714	0,860	0,0161187	0,872	0,866
206197_at	**NME5**	non-metastatic cells 5, protein expressed in (nucleoside-diphosphate kinase)	0,0306722	0,718	0,0479265	0,756	0,737
205775_at	**FAM50B**	family with sequence similarity 50, member B	0,0308098	0,865	0,0471341	0,798	0,832
224319_s_at	**FLJ20232**	NA	0,0308202	0,790	0,0012580	0,799	0,795
201321_s_at	**SMARCC2**	SWI/SNF related, matrix associated, actin dependent regulator of chromatin, subfamily c, member 2	0,0308515	0,828	0,0047134	0,733	0,781
224461_s_at	**AMID**	apoptosis-inducing factor (AIF)-like mitochondrion-associated inducer of death	0,0308618	0,880	0,0093761	0,765	0,822
207233_s_at	**MITF**	microphthalmia-associated transcription factor	0,0308618	0,692	0,0009353	0,537	0,615
227965_at	**APC2**	adenomatosis polyposis coli 2	0,0308701	0,754	0,0017270	0,695	0,725
217054_at	**MUC3A**	mucin 3A, intestinal	0,0308701	0,810	0,0414054	0,816	0,813
200788_s_at	**PEA15**	phosphoprotein enriched in astrocytes 15	0,0309720	0,889	0,0178330	0,859	0,874
203796_s_at	**BCL7A**	B-cell CLL/lymphoma 7A	0,0310018	0,850	0,0284491	0,752	0,801
225101_s_at	**SNX14**	sorting nexin 14	0,0310211	0,820	0,0509015	0,904	0,862
214680_at	**NTRK2**	neurotrophic tyrosine kinase, receptor, type 2	0,0310565	0,365	0,0325152	0,398	0,382
204341_at	**TRIM16**	tripartite motif-containing 16	0,0311159	0,727	0,0061755	0,587	0,657

203365_s_at	**MMP15**	matrix metallopeptidase 15 (membrane-inserted)	0,0311432	0,760	0,0444921	0,837	0,798
207826_s_at	**ID3**	inhibitor of DNA binding 3, dominant negative helix-loop-helix protein	0,0312862	0,621	0,0304220	0,619	0,620
1569029_at	**MAP1D**	NA	0,0312862	0,771	0,0601829	0,751	0,761
203768_s_at	**STS**	steroid sulfatase (microsomal), arylsulfatase C, isozyme S	0,0313043	0,724	0,0315805	0,679	0,701
240890_at	**LOC440066**	NA	0,0313460	0,376	0,0088796	0,334	0,355
239764_at	**LOC401048**	NA	0,0314200	0,885	0,0067407	0,861	0,873
224466_s_at	**MAFG**	v-maf musculoaponeurotic fibrosarcoma oncogene homolog G (avian)	0,0314234	0,812	0,0002160	0,654	0,733
239546_at	**PQLC2**	PQ loop repeat containing 2	0,0314766	0,857	0,0204257	0,748	0,802
212282_at	**MAC30**	NA	0,0315109	0,561	0,0070092	0,415	0,488
226440_at	**DUSP22**	dual specificity phosphatase 22	0,0315338	0,805	0,0469770	0,788	0,796
226747_at	**KIAA1344**	KIAA1344	0,0315750	0,540	0,0293878	0,579	0,559
209113_s_at	**HMG20B**	high-mobility group 20B	0,0315779	0,794	0,0050096	0,702	0,748
217804_s_at	**ILF3**	interleukin enhancer binding factor 3, 90kDa	0,0316045	0,891	0,0381030	0,826	0,858
218523_at	**LHPP**	NA	0,0316146	0,877	0,0551055	0,791	0,834
210114_at	**INVS**	inversin	0,0316671	0,760	0,0520961	0,797	0,778
207566_at	**MR1**	major histocompatibility complex, class I-related	0,0316712	0,750	0,0553315	0,774	0,762
221410_x_at	**PCDHB3**	protocadherin beta 3	0,0316856	0,776	0,0176714	0,761	0,769
1560115_a_at	**KIAA1217**	KIAA1217	0,0316951	0,744	0,0383722	0,878	0,811
218701_at	**LACTB2**	lactamase, beta 2	0,0317001	0,558	0,0458018	0,521	0,539
226933_s_at	**ID4**	inhibitor of DNA binding 4, dominant negative helix-loop-helix protein	0,0317089	0,593	0,0393701	0,554	0,573
226816_s_at	**KIAA1143**	KIAA1143	0,0317149	0,787	0,0320653	0,751	0,769
225733_at	**B3GALT6**	UDP-Gal:betaGal beta 1,3-galactosyltransferase polypeptide 6	0,0317892	0,846	0,0457121	0,872	0,859
210910_s_at	**POMZP3**	POM (POM121 homolog, rat) and ZP3 fusion	0,0318199	0,761	0,0293233	0,511	0,636
224636_at	**ZFP91**	zinc finger protein 91 homolog (mouse)	0,0318350	0,869	0,0157490	0,696	0,782
214958_s_at	**EVER1**	epidermodysplasia verruciformis 1	0,0319358	0,822	0,0276152	0,854	0,838
235992_s_at	**LOC200008**	NA	0,0319423	0,830	0,0011348	0,596	0,713
238129_s_at	**FZD2**	frizzled homolog 2 (Drosophila)	0,0319590	0,704	0,0111801	0,599	0,651
203151_at	**MAP1A**	microtubule-associated protein 1A	0,0319682	0,680	0,0345541	0,591	0,636
212108_at	**ETEA**	NA	0,0320136	0,846	0,0511079	0,856	0,851
201871_s_at	**LOC51035**	NA	0,0320586	0,786	0,0060850	0,799	0,792
229889_at	**FLJ35696**	NA	0,0321650	0,842	0,0019074	0,811	0,827
240187_at	**PPP1R3C**	protein phosphatase 1, regulatory (inhibitor) subunit 3C	0,0321937	0,576	0,0048648	0,656	0,616
221503_s_at	**KPNA3**	karyopherin alpha 3 (importin alpha 4)	0,0322273	0,673	0,0000000	0,771	0,722
238426_at	**DKFZp761L1417**	NA	0,0322291	0,648	0,0288259	0,282	0,465
224766_at	**RPL37**	ribosomal protein L37	0,0322694	0,746	0,0424311	0,582	0,664
215843_s_at	**TLL2**	tolloid-like 2	0,0323133	0,734	0,0367936	0,770	0,752
201061_s_at	**STOM**	stomatin	0,0323729	0,522	0,0408620	0,561	0,542
208101_s_at	**C9orf74**	chromosome 9 open reading frame 74	0,0324051	0,902	0,0137539	0,882	0,892
213893_x_at	**PMS2L5**	postmeiotic segregation increased 2-like 5	0,0324051	0,850	0,0008206	0,772	0,811
221277_s_at	**PUS3**	pseudouridylate synthase 3	0,0325689	0,782	0,0259829	0,828	0,805
217951_s_at	**PHF3**	PHD finger protein 3	0,0325933	0,696	0,0450546	0,815	0,756
227534_at	**C9orf21**	chromosome 9 open reading frame 21	0,0326000	0,781	0,0086199	0,727	0,754
202658_at	**PEX11B**	peroxisomal biogenesis factor 11B	0,0326432	0,907	0,0191998	0,806	0,856
203909_at	**SLC9A6**	solute carrier family 9 (sodium/hydrogen exchanger), member 6	0,0326632	0,788	0,0026634	0,778	0,783
218417_s_at	**FLJ20489**	NA	0,0327053	0,724	0,0000313	0,527	0,625
238681_at	**GDPD1**	glycerophosphodiester phosphodiesterase domain containing 1	0,0327814	0,750	0,0537455	0,735	0,742
235424_at	**C9orf42**	chromosome 9 open reading frame 42	0,0327870	0,772	0,0405844	0,826	0,799
226065_at	**PRICKLE1**	prickle-like 1 (Drosophila)	0,0328054	0,600	0,0351794	0,540	0,570
218759_at	**DVL2**	dishevelled, dsh homolog 2 (Drosophila)	0,0328130	0,707	0,0003403	0,574	0,641
228189_at	**BAG4**	BCL2-associated athanogene 4	0,0328964	0,799	0,0064069	0,615	0,707
228130_at	**ZNF397**	zinc finger protein 397	0,0329030	0,790	0,0524745	0,892	0,841
217929_s_at	**KIAA0319L**	KIAA0319-like	0,0329341	0,787	0,0000000	0,638	0,712
219369_s_at	**OTUB2**	OTU domain, ubiquitin aldehyde binding 2	0,0330686	0,849	0,0086107	0,768	0,808
241984_at	**CHES1**	checkpoint suppressor 1	0,0330733	0,777	0,0245690	0,454	0,616
236841_at	**FLJ25222**	NA	0,0330770	0,614	0,0294626	0,794	0,704
219054_at	**FLJ14054**	NA	0,0331018	0,692	0,0446588	0,673	0,683
204719_at	**ABCA8**	ATP-binding cassette, sub-family A (ABC1), member 8	0,0331128	0,473	0,0275551	0,398	0,436
235021_at	**KIAA2026**	KIAA2026	0,0331128	0,871	0,0463699	0,847	0,859
218138_at	**MKKS**	McKusick-Kaufman syndrome	0,0331128	0,814	0,0509925	0,739	0,776
206240_s_at	**ZNF136**	zinc finger protein 136 (clone pHZ-20)	0,0331128	0,757	0,0262034	0,815	0,786
208510_s_at	**PPARG**	peroxisome proliferative activated receptor, gamma	0,0331525	0,541	0,0280563	0,434	0,487
219508_at	**GCNT3**	glucosaminyl (N-acetyl) transferase 3, mucin type	0,0331828	0,642	0,0047009	0,757	0,700
227731_at	**ZNF9**	zinc finger protein 9 (a cellular retroviral nucleic acid binding protein)	0,0332488	0,824	0,0021875	0,811	0,817

201229_s_at	**ARIH2**	ariadne homolog 2 (Drosophila)	0,0332706	0,832	0,0357161	0,794	0,813
226552_at	**IER5L**	immediate early response 5-like	0,0333458	0,704	0,0001433	0,546	0,625
223684_s_at	**SMUG1**	NA	0,0333498	0,835	0,0541178	0,718	0,777
224626_at	**SLC35A4**	solute carrier family 35, member A4	0,0333793	0,850	0,0225278	0,822	0,836
219228_at	**ZNF331**	zinc finger protein 331	0,0333801	0,749	0,0044184	0,612	0,681
203277_at	**DFFA**	DNA fragmentation factor, 45kDa, alpha polypeptide	0,0333810	0,773	0,0000000	0,577	0,675
218999_at	**FLJ11000**	NA	0,0335577	0,423	0,0076449	0,328	0,375
201810_s_at	**SH3BP5**	SH3-domain binding protein 5 (BTK-associated)	0,0336424	0,720	0,0177471	0,587	0,654
219931_s_at	**KLHL12**	kelch-like 12 (Drosophila)	0,0336611	0,736	0,0155365	0,802	0,769
222360_at	**DPH5**	DPH5 homolog (S. cerevisiae)	0,0337141	0,708	0,0328483	0,722	0,715
1552739_s_at	**ST7L**	suppression of tumorigenicity 7 like	0,0337470	0,820	0,0214687	0,773	0,796
233532_x_at	**C20orf9**	chromosome 20 open reading frame 9	0,0337688	0,725	0,0048299	0,751	0,738
229949_at	**FKBP6**	FK506 binding protein 6, 36kDa	0,0338217	0,772	0,0080574	0,718	0,745
208928_at	**POR**	P450 (cytochrome) oxidoreductase	0,0338217	0,746	0,0002614	0,587	0,666
229690_at	**FLJ32356**	NA	0,0338402	0,848	0,0004756	0,722	0,785
215412_x_at	**PMS2L2**	postmeiotic segregation increased 2-like 2	0,0338402	0,874	0,0105842	0,787	0,830
204082_at	**PBX3**	pre-B-cell leukemia transcription factor 3	0,0339250	0,790	0,0254285	0,731	0,761
218638_s_at	**SPON2**	spondin 2, extracellular matrix protein	0,0339572	0,450	0,0256365	0,519	0,485
226813_at	**C1orf57**	chromosome 1 open reading frame 57	0,0340057	0,814	0,0485685	0,808	0,811
230733_at	**MRCL3**	NA	0,0340195	0,575	0,0431918	0,556	0,566
242503_at	**CHST13**	carbohydrate (chondroitin 4) sulfotransferase 13	0,0340915	0,840	0,0357209	0,857	0,848
212343_at	**YIPF6**	Yip1 domain family, member 6	0,0340915	0,844	0,0547533	0,800	0,822
224681_at	**GNA12**	guanine nucleotide binding protein (G protein) alpha 12	0,0341138	0,867	0,0105657	0,837	0,852
203027_s_at	**MVD**	mevalonate (diphospho) decarboxylase	0,0341167	0,775	0,0070222	0,718	0,746
236737_at	**FLJ31528**	NA	0,0341319	0,908	0,0444669	0,901	0,905
230453_s_at	**ATP2A3**	ATPase, Ca++ transporting, ubiquitous	0,0341560	0,857	0,0452815	0,861	0,859
205508_at	**SCN1B**	sodium channel, voltage-gated, type I, beta	0,0343400	0,744	0,0595813	0,769	0,757
203179_at	**GALT**	galactose-1-phosphate uridylyltransferase	0,0343559	0,783	0,0051353	0,781	0,782
225693_s_at	**CAMTA1**	calmodulin binding transcription activator 1	0,0343729	0,875	0,0541474	0,859	0,867
201080_at	**PIP5K2B**	phosphatidylinositol-4-phosphate 5-kinase, type II, beta	0,0344315	0,824	0,0170496	0,829	0,827
244401_at	**C6orf152**	chromosome 6 open reading frame 152	0,0345596	0,778	0,0420365	0,870	0,824
212861_at	**MGC11308**	NA	0,0345878	0,821	0,0025745	0,752	0,787
235487_at	**CD63**	CD63 antigen (melanoma 1 antigen)	0,0346197	0,859	0,0365116	0,773	0,816
53987_at	**RANBP10**	RAN binding protein 10	0,0346197	0,859	0,0006629	0,755	0,807
210870_s_at	**EPM2A**	epilepsy, progressive myoclonus type 2A, Lafora disease (laforin)	0,0346211	0,745	0,0371404	0,687	0,716
202245_at	**LSS**	lanosterol synthase (2,3-oxidosqualene-lanosterol cyclase)	0,0346277	0,685	0,0048596	0,554	0,619
212240_s_at	**PIK3R1**	phosphoinositide-3-kinase, regulatory subunit 1 (p85 alpha)	0,0346405	0,566	0,0386841	0,625	0,595
218922_s_at	**LASS4**	LAG1 longevity assurance homolog 4 (S. cerevisiae)	0,0346472	0,812	0,0284478	0,710	0,761
220419_s_at	**USP25**	ubiquitin specific peptidase 25	0,0346472	0,754	0,0044632	0,730	0,742
219941_at	**TMEM19**	transmembrane protein 19	0,0346522	0,854	0,0324992	0,870	0,862
225063_at	**UBL7**	ubiquitin-like 7 (bone marrow stromal cell-derived)	0,0346603	0,885	0,0164081	0,823	0,854
221582_at	**HIST3H2A**	histone 3, H2a	0,0347642	0,662	0,0553827	0,718	0,690
236042_at	**RAD52**	RAD52 homolog (S. cerevisiae)	0,0348253	0,630	0,0091656	0,701	0,665
232067_at	**C6orf168**	chromosome 6 open reading frame 168	0,0348708	0,788	0,0347790	0,773	0,781
207661_s_at	**SH3MD1**	SH3 multiple domains 1	0,0348846	0,783	0,0288318	0,709	0,746
219681_s_at	**RAB11FIP1**	RAB11 family interacting protein 1 (class I)	0,0348950	0,581	0,0207848	0,564	0,572
237423_at	**RSPO4**	R-spondin family, member 4	0,0348950	0,879	0,0288735	0,760	0,819
227649_s_at	**SRGAP2**	SLIT-ROBO Rho GTPase activating protein 2	0,0349085	0,837	0,0333386	0,750	0,794
236555_at	**REV3L**	REV3-like, catalytic subunit of DNA polymerase zeta (yeast)	0,0349546	0,795	0,0393764	0,888	0,841
226736_at	**CHURC1**	churchill domain containing 1	0,0349838	0,809	0,0553445	0,713	0,761
226021_at	**RDH10**	retinol dehydrogenase 10 (all-trans)	0,0349838	0,747	0,0072624	0,514	0,630
203096_s_at	**RAPGEF2**	Rap guanine nucleotide exchange factor (GEF) 2	0,0349841	0,697	0,0547647	0,897	0,797
243575_at	**LOC375449**	NA	0,0350163	0,832	0,0279190	0,858	0,845
1561180_at	**LRP11**	low density lipoprotein receptor-related protein 11	0,0350171	0,451	0,0008206	0,360	0,405
213400_s_at	**TBL1X**	transducin (beta)-like 1X-linked	0,0350330	0,771	0,0510309	0,789	0,780
224801_at	**NDFIP2**	Nedd4 family interacting protein 2	0,0351223	0,660	0,0281764	0,687	0,674
202317_s_at	**UBE4B**	ubiquitination factor E4B (UFD2 homolog, yeast)	0,0351883	0,835	0,0446984	0,783	0,809
230433_at	**LOC400764**	NA	0,0351948	0,815	0,0404046	0,741	0,778
227229_at	**FLJ10979**	NA	0,0352100	0,872	0,0284118	0,770	0,821
225651_at	**UBE2E2**	ubiquitin-conjugating enzyme E2E 2 (UBC4/5 homolog, yeast)	0,0352100	0,715	0,0095315	0,645	0,680
224669_at	**C20orf35**	chromosome 20 open reading frame 35	0,0352446	0,824	0,0317752	0,823	0,824
205698_s_at	**MAP2K6**	mitogen-activated protein kinase kinase 6	0,0352446	0,659	0,0171692	0,837	0,748

205348_s_at	**DNCI1**	dynein, cytoplasmic, intermediate polypeptide 1	0,0353800	0,705	0,0469386	0,643	0,674
213040_s_at	**NPTXR**	neuronal pentraxin receptor	0,0355246	0,495	0,0019161	0,472	0,483
226500_at	**ZNF651**	zinc finger protein 651	0,0355246	0,834	0,0246884	0,803	0,818
202922_at	**GCLC**	glutamate-cysteine ligase, catalytic subunit	0,0355361	0,801	0,0481322	0,736	0,769
202793_at	**C3F**	NA	0,0355771	0,786	0,0106579	0,819	0,803
210124_x_at	**SEMA4F**	sema domain, immunoglobulin domain (Ig), transmembrane domain (TM) and short cytoplasmic domain, (semaphorin) 4F	0,0355771	0,806	0,0375959	0,849	0,827
235050_at	**SLC2A12**	solute carrier family 2 (facilitated glucose transporter), member 12	0,0356079	0,632	0,0255380	0,191	0,411
238754_at	**PTCH**	patched homolog (Drosophila)	0,0356928	0,829	0,0545850	0,888	0,858
201544_x_at	**PABPN1**	poly(A) binding protein, nuclear 1	0,0357007	0,819	0,0024963	0,752	0,785
224011_at	**SLC1A5**	solute carrier family 1 (neutral amino acid transporter), member 5	0,0357031	0,857	0,0064297	0,721	0,789
223183_at	**AGPAT3**	1-acylglycerol-3-phosphate O-acyltransferase 3	0,0357086	0,807	0,0048133	0,672	0,739
219751_at	**FLJ21148**	NA	0,0357238	0,782	0,0356054	0,784	0,783
209349_at	**RAD50**	RAD50 homolog (S. cerevisiae)	0,0357819	0,746	0,0412746	0,739	0,743
226534_at	**KITLG**	KIT ligand	0,0358033	0,487	0,0486469	0,578	0,532
203616_at	**POLB**	polymerase (DNA directed), beta	0,0358590	0,869	0,0094390	0,907	0,888
244187_at	**CXorf33**	chromosome X open reading frame 33	0,0358845	0,491	0,0471714	0,758	0,625
224887_at	**GNPTG**	N-acetylglucosamine-1-phosphate transferase, gamma subunit	0,0359011	0,816	0,0304700	0,749	0,782
208752_x_at	**NAP1L1**	nucleosome assembly protein 1-like 1	0,0359110	0,821	0,0158770	0,843	0,832
243761_at	**CLDN12**	claudin 12	0,0359121	0,872	0,0047620	0,721	0,796
216525_x_at	**PMS2L11**	postmeiotic segregation increased 2-like 11	0,0359883	0,820	0,0045215	0,853	0,836
206797_at	**NAT2**	N-acetyltransferase 2 (arylamine N-acetyltransferase)	0,0360043	0,744	0,0317552	0,763	0,754
209246_at	**ABCF2**	ATP-binding cassette, sub-family F (GCN20), member 2	0,0360398	0,866	0,0040832	0,724	0,795
223657_at	**C1orf90**	chromosome 1 open reading frame 90	0,0360764	0,687	0,0000000	0,603	0,645
228263_at	**GRASP**	GRP1 (general receptor for phosphoinositides 1)-associated scaffold protein	0,0360764	0,815	0,0236325	0,750	0,783
212228_s_at	**C16orf49**	chromosome 16 open reading frame 49	0,0360873	0,860	0,0373257	0,846	0,853
223656_s_at	**C1orf91**	chromosome 1 open reading frame 91	0,0360873	0,875	0,0327387	0,888	0,881
222248_s_at	**SIRT4**	sirtuin (silent mating type information regulation 2 homolog) 4 (S. cerevisiae)	0,0360873	0,855	0,0503830	0,857	0,856
239222_at	**C9orf9**	chromosome 9 open reading frame 9	0,0361298	0,891	0,0005796	0,721	0,806
213590_at	**SLC16A5**	solute carrier family 16 (monocarboxylic acid transporters), member 5	0,0361416	0,818	0,0422334	0,747	0,782
1557970_s_at	**RPS6KA2**	ribosomal protein S6 kinase, 90kDa, polypeptide 2	0,0361845	0,811	0,0446694	0,655	0,733
209063_x_at	**LOC388345**	NA	0,0363159	0,841	0,0503310	0,901	0,871
225559_at	**C3orf19**	chromosome 3 open reading frame 19	0,0364762	0,872	0,0599565	0,866	0,869
213976_at	**CIZ1**	CDKN1A interacting zinc finger protein 1	0,0364762	0,849	0,0039168	0,676	0,763
1558702_at	**TEX10**	testis expressed sequence 10	0,0364762	0,692	0,0003110	0,528	0,610
230256_at	**C1orf104**	chromosome 1 open reading frame 104	0,0365124	0,616	0,0435275	0,689	0,653
209772_s_at	**CD24**	CD24 antigen (small cell lung carcinoma cluster 4 antigen)	0,0365240	0,480	0,0436930	0,325	0,402
222401_s_at	**TMEM50A**	transmembrane protein 50A	0,0365636	0,796	0,0161759	0,687	0,741
224339_s_at	**ANGPTL1**	angiopoietin-like 1	0,0366057	0,567	0,0412277	0,576	0,571
235198_at	**OSTM1**	osteopetrosis associated transmembrane protein 1	0,0366057	0,707	0,0049926	0,607	0,657
233613_x_at	**REXO2**	REX2, RNA exonuclease 2 homolog (S. cerevisiae)	0,0366863	0,871	0,0171081	0,902	0,886
203723_at	**ITPKB**	inositol 1,4,5-trisphosphate 3-kinase B	0,0368670	0,688	0,0153815	0,619	0,653
208523_x_at	**HIST1H2BI**	histone 1, H2bi	0,0369123	0,781	0,0413784	0,788	0,785
229393_at	**L3MBTL3**	l(3)mbt-like 3 (Drosophila)	0,0369543	0,696	0,0155782	0,676	0,686
222903_s_at	**CPEB1**	cytoplasmic polyadenylation element binding protein 1	0,0369656	0,822	0,0120187	0,456	0,639
222528_s_at	**SLC25A37**	solute carrier family 25, member 37	0,0370615	0,500	0,0534086	0,680	0,590
230232_at	**KIAA0746**	NA	0,0370766	0,900	0,0492237	0,593	0,746
225588_s_at	**LOC92305**	NA	0,0370872	0,787	0,0070029	0,733	0,760
217700_at	**MGC40499**	NA	0,0370872	0,808	0,0233160	0,777	0,792
223177_at	**NT5C2L1**	5'-nucleotidase, cytosolic II-like 1	0,0370872	0,754	0,0008967	0,552	0,653
206174_s_at	**PPP6C**	protein phosphatase 6, catalytic subunit	0,0370872	0,861	0,0128347	0,819	0,840
214455_at	**HIST1H2BC**	histone 1, H2bc	0,0370974	0,647	0,0315858	0,633	0,640
1561425_a_at	**ZNF568**	zinc finger protein 568	0,0370974	0,839	0,0054042	0,764	0,801
219838_at	**TTC23**	tetratricopeptide repeat domain 23	0,0371075	0,871	0,0019882	0,789	0,830
225728_at	**ARGBP2**	NA	0,0371401	0,579	0,0448942	0,409	0,494
223378_at	**GLIS2**	GLIS family zinc finger 2	0,0371635	0,781	0,0054947	0,777	0,779
217962_at	**NOLA3**	nucleolar protein family A, member 3 (H/ACA small nucleolar RNPs)	0,0371635	0,811	0,0265811	0,850	0,831
212503_s_at	**KIAA0934**	KIAA0934	0,0371974	0,797	0,0424296	0,758	0,778
209407_s_at	**DEAF1**	deformed epidermal autoregulatory factor 1 (Drosophila)	0,0372493	0,741	0,0339959	0,632	0,687
213340_s_at	**KIAA0495**	NA	0,0372961	0,798	0,0100129	0,816	0,807

230289_at	**EPB41L1**	erythrocyte membrane protein band 4.1-like 1	0,0373125	0,869	0,0000000	0,416	0,642
232470_at	**SNF1LK**	SNF1-like kinase	0,0373125	0,877	0,0034050	0,790	0,834
200976_s_at	**TAX1BP1**	Tax1 (human T-cell leukemia virus type I) binding protein 1	0,0373605	0,811	0,0068209	0,801	0,806
208796_s_at	**CCNG1**	cyclin G1	0,0373989	0,686	0,0492606	0,699	0,692
34406_at	**PACS2**	phosphofurin acidic cluster sorting protein 2	0,0373989	0,853	0,0013547	0,706	0,779
1557856_at	**ABHD4**	abhydrolase domain containing 4	0,0374217	0,904	0,0246764	0,598	0,751
203899_s_at	**RCP9**	NA	0,0374506	0,835	0,0514587	0,900	0,867
218970_s_at	**CUTC**	cutC copper transporter homolog (E.coli)	0,0374853	0,769	0,0021152	0,688	0,728
217941_s_at	**ERBB2IP**	erbb2 interacting protein	0,0375154	0,794	0,0253269	0,872	0,833
235552_at	**KIAA1627**	NA	0,0375194	0,818	0,0017413	0,742	0,780
226007_at	**HBLD1**	HESB like domain containing 1	0,0376162	0,756	0,0110385	0,762	0,759
218707_at	**ZNF444**	zinc finger protein 444	0,0376347	0,909	0,0272470	0,791	0,850
214661_s_at	**C4orf9**	chromosome 4 open reading frame 9	0,0376593	0,855	0,0268023	0,850	0,852
238326_at	**LOC440836**	NA	0,0377167	0,725	0,0428038	0,861	0,793
231017_at	**STK11**	serine/threonine kinase 11 (Peutz-Jeghers syndrome)	0,0377473	0,802	0,0014688	0,737	0,770
229067_at	**LOC440608**	NA	0,0378028	0,670	0,0093779	0,676	0,673
203436_at	**RPP30**	ribonuclease P/MRP 30kDa subunit	0,0378218	0,882	0,0181166	0,872	0,877
238568_s_at	**NPC1**	Niemann-Pick disease, type C1	0,0378550	0,659	0,0382085	0,617	0,638
1555632_at	**MGC17330**	NA	0,0378604	0,904	0,0397010	0,631	0,768
1563498_s_at	**LOC283130**	NA	0,0379197	0,720	0,0207061	0,758	0,739
235695_at	**INPP4A**	inositol polyphosphate-4-phosphatase, type I, 107kDa	0,0380307	0,843	0,0233593	0,814	0,828
1553971_a_at	**GATS**	NA	0,0381244	0,775	0,0418991	0,831	0,803
203955_at	**KIAA0649**	KIAA0649	0,0381398	0,818	0,0321504	0,768	0,793
208852_s_at	**CANX**	calnexin	0,0381471	0,845	0,0148522	0,904	0,874
227675_at	**LRSAM1**	leucine rich repeat and sterile alpha motif containing 1	0,0382055	0,857	0,0013125	0,737	0,797
219238_at	**PIGV**	phosphatidylinositol glycan, class V	0,0382980	0,793	0,0247089	0,781	0,787
234192_s_at	**GKAP1**	G kinase anchoring protein 1	0,0383724	0,832	0,0222909	0,859	0,845
232373_at	**NOXA1**	NADPH oxidase activator 1	0,0383783	0,890	0,0443117	0,809	0,850
221798_x_at	**RPS2**	ribosomal protein S2	0,0384306	0,957	0,0233739	0,746	0,851
212055_at	**C18orf10**	chromosome 18 open reading frame 10	0,0384503	0,800	0,0277997	0,781	0,790
227692_at	**GNAI1**	guanine nucleotide binding protein (G protein), alpha inhibiting activity polypeptide 1	0,0384503	0,718	0,0019614	0,676	0,697
224703_at	**WDR22**	WD repeat domain 22	0,0384503	0,844	0,0238741	0,773	0,809
228435_at	**FEM1A**	fem-1 homolog a (C.elegans)	0,0385984	0,663	0,0481600	0,795	0,729
1559315_s_at	**LOC144481**	NA	0,0386319	0,637	0,0024132	0,678	0,657
241680_at	**MBD3**	methyl-CpG binding domain protein 3	0,0387011	0,786	0,0007599	0,800	0,793
204984_at	**GPC4**	glypican 4	0,0387275	0,531	0,0260360	0,584	0,557
214251_s_at	**NUMA1**	nuclear mitotic apparatus protein 1	0,0387345	0,737	0,0017874	0,717	0,727
218439_s_at	**COMMD10**	COMM domain containing 10	0,0388388	0,819	0,0483929	0,884	0,852
1569748_at	**PRKCZ**	protein kinase C, zeta	0,0389034	0,891	0,0163183	0,874	0,883
1553449_at	**FLJ36701**	NA	0,0389039	0,816	0,0367418	0,730	0,773
225789_at	**CENTG3**	centaurin, gamma 3	0,0389065	0,873	0,0173761	0,857	0,865
212215_at	**PREPL**	prolyl endopeptidase-like	0,0389065	0,797	0,0115116	0,759	0,778
219940_s_at	**FLJ11305**	NA	0,0389174	0,794	0,0070042	0,779	0,786
242002_at	**TCBA1**	T-cell lymphoma breakpoint associated target 1	0,0389288	0,640	0,0373662	0,506	0,573
243924_at	**LOC440524**	NA	0,0389408	0,819	0,0520047	0,805	0,812
213394_at	**MAPKBP1**	mitogen activated protein kinase binding protein 1	0,0389556	0,841	0,0123461	0,828	0,835
214179_s_at	**NFE2L1**	nuclear factor (erythroid-derived 2)-like 1	0,0389556	0,827	0,0097077	0,739	0,783
229897_at	**ZNF641**	zinc finger protein 641	0,0389556	0,835	0,0577081	0,789	0,812
200049_at	**MYST2**	MYST histone acetyltransferase 2	0,0389665	0,875	0,0232089	0,790	0,833
1556180_at	**LOC255458**	NA	0,0389819	0,746	0,0404417	0,727	0,736
1557359_at	**LOC285758**	NA	0,0390494	0,357	0,0335091	0,402	0,380
214736_s_at	**ADD1**	adducin 1 (alpha)	0,0390498	0,847	0,0338189	0,798	0,823
215000_s_at	**FEZ2**	fasciculation and elongation protein zeta 2 (zygin II)	0,0390740	0,806	0,0035777	0,706	0,756
221754_s_at	**CORO1B**	coronin, actin binding protein, 1B	0,0391155	0,782	0,0451794	0,700	0,741
228953_at	**KIAA1971**	NA	0,0391487	0,837	0,0137403	0,853	0,845
211552_s_at	**ALDH4A1**	aldehyde dehydrogenase 4 family, member A1	0,0392188	0,659	0,0033010	0,533	0,596
239749_at	**FAF1**	Fas (TNFRSF6) associated factor 1	0,0392291	0,827	0,0472001	0,880	0,854
205279_s_at	**GLRB**	glycine receptor, beta	0,0392329	0,704	0,0401584	0,685	0,694
220476_s_at	**C1orf183**	chromosome 1 open reading frame 183	0,0392833	0,843	0,0344502	0,775	0,809
235853_at	**MGC33365**	NA	0,0392833	0,902	0,0254197	0,683	0,793
233297_s_at	**FLJ20449**	NA	0,0393603	0,798	0,0539847	0,894	0,846
219334_s_at	**FLJ22833**	NA	0,0393720	0,775	0,0045600	0,611	0,693
239837_at	**ADAM11**	ADAM metallopeptidase domain 11	0,0394239	0,862	0,0348545	0,847	0,855
219417_s_at	**FLJ20014**	NA	0,0395571	0,863	0,0012971	0,798	0,831
219394_at	**PGS1**	NA	0,0396134	0,836	0,0310497	0,866	0,851
209678_s_at	**PRKCI**	protein kinase C, iota	0,0396358	0,726	0,0214711	0,765	0,746
230205_at	**ZNF561**	zinc finger protein 561	0,0396358	0,720	0,0168036	0,726	0,723
1555882_at	**SPIN3**	spindlin family, member 3	0,0397608	0,795	0,0528049	0,739	0,767
225257_at	**MGC20255**	NA	0,0397701	0,783	0,0242775	0,779	0,781

209001_s_at	**ANAPC13**	anaphase promoting complex subunit 13	0,0397845	0,725	0,0537199	0,812	0,769
1556787_s_at	**PDE5A**	phosphodiesterase 5A, cGMP-specific	0,0398455	0,849	0,0098548	0,686	0,767
210963_s_at	**GYG2**	glycogenin 2	0,0398568	0,671	0,0557026	0,707	0,689
229758_at	**TIGD5**	tigger transposable element derived 5	0,0399063	0,878	0,0371848	0,894	0,886
1554522_at	**CNNM2**	cyclin M2	0,0399090	0,790	0,0236544	0,611	0,700
1554062_at	**XG**	Xg blood group (pseudoautosomal boundary-divided on the X chromosome)	0,0399466	0,682	0,0412030	0,652	0,667
216486_x_at	**ZNF79**	zinc finger protein 79 (pT7)	0,0400260	0,788	0,0066741	0,778	0,783
218056_at	**BFAR**	bifunctional apoptosis regulator	0,0400515	0,905	0,0345174	0,872	0,888
203996_s_at	**C21orf2**	chromosome 21 open reading frame 2	0,0401362	0,882	0,0261493	0,802	0,842
228237_at	**PAPPA2**	pappalysin 2	0,0401511	0,783	0,0206033	0,727	0,755
239260_at	**CORIN**	corin, serine peptidase	0,0401829	0,320	0,0343908	0,541	0,431
224750_at	**RNF185**	ring finger protein 185	0,0402136	0,859	0,0048596	0,801	0,830
235135_at	**ILK**	integrin-linked kinase	0,0402176	0,806	0,0033511	0,828	0,817
201908_at	**DVL3**	dishevelled, dsh homolog 3 (Drosophila)	0,0402216	0,711	0,0232869	0,581	0,646
238708_at	**BC036928**	NA	0,0402773	0,837	0,0404799	0,852	0,844
226845_s_at	**MYEOV2**	myeloma overexpressed 2	0,0402773	0,894	0,0206710	0,851	0,873
228262_at	**FLJ14503**	NA	0,0402809	0,722	0,0160790	0,653	0,687
204074_s_at	**GlyBP**	NA	0,0402809	0,807	0,0067670	0,783	0,795
202738_s_at	**PHKB**	phosphorylase kinase, beta	0,0402974	0,809	0,0557692	0,781	0,795
222392_x_at	**PERP**	PERP, TP53 apoptosis effector	0,0403133	0,763	0,0382995	0,770	0,766
226255_at	**ZBTB33**	zinc finger and BTB domain containing 33	0,0403633	0,830	0,0525062	0,830	0,830
202308_at	**SREBF1**	sterol regulatory element binding transcription factor 1	0,0403882	0,482	0,0463718	0,876	0,679
212683_at	**KIAA0446**	NA	0,0404809	0,824	0,0601894	0,875	0,849
235263_at	**LOC441254**	NA	0,0404828	0,848	0,0012157	0,753	0,800
214545_s_at	**PROSC**	proline synthetase co-transcribed homolog (bacterial)	0,0405546	0,759	0,0450786	0,765	0,762
206907_at	**TNFSF9**	tumor necrosis factor (ligand) superfamily, member 9	0,0405917	0,679	0,0190123	0,619	0,649
222457_s_at	**EPLIN**	NA	0,0405940	0,757	0,0078105	0,779	0,768
217282_at	**MAN1A2**	mannosidase, alpha, class 1A, member 2	0,0405940	0,922	0,0077859	0,843	0,883
217800_s_at	**NDFIP1**	Nedd4 family interacting protein 1	0,0405940	0,847	0,0007133	0,630	0,738
231319_x_at	**KIF9**	kinesin family member 9	0,0406932	0,812	0,0401584	0,883	0,847
228421_s_at	**EFEMP1**	EGF-containing fibulin-like extracellular matrix protein 1	0,0407475	0,856	0,0567150	0,863	0,860
230058_at	**SDCCAG3**	serologically defined colon cancer antigen 3	0,0408659	0,812	0,0554210	0,845	0,828
214665_s_at	**CHP**	NA	0,0408966	0,837	0,0078445	0,817	0,827
214674_at	**USP19**	ubiquitin specific peptidase 19	0,0409289	0,819	0,0347795	0,833	0,826
204876_at	**ZNF646**	zinc finger protein 646	0,0409482	0,825	0,0081129	0,812	0,819
236983_at	**TMC5**	transmembrane channel-like 5	0,0409765	0,916	0,0238747	0,831	0,873
218347_at	**RSAFD1**	radical S-adenosyl methionine and flavodoxin domains 1	0,0409895	0,827	0,0344242	0,847	0,837
225345_s_at	**FBXO32**	F-box protein 32	0,0409997	0,630	0,0541012	0,496	0,563
213235_at	**LOC400506**	NA	0,0410023	0,868	0,0375107	0,836	0,852
222736_s_at	**TMEM38B**	transmembrane protein 38B	0,0410441	0,681	0,0281222	0,729	0,705
1558755_x_at	**ZNF440L**	NA	0,0410441	0,805	0,0270732	0,828	0,817
209411_s_at	**GGA3**	golgi associated, gamma adaptin ear containing, ARF binding protein 3	0,0410962	0,829	0,0016655	0,760	0,795
225050_at	**ZNF512**	zinc finger protein 512	0,0411709	0,737	0,0438346	0,733	0,735
201771_at	**SCAMP3**	secretory carrier membrane protein 3	0,0412955	0,875	0,0418258	0,837	0,856
222573_s_at	**SAV1**	salvador homolog 1 (Drosophila)	0,0413256	0,695	0,0158370	0,391	0,543
51774_s_at	**UBE2D4**	ubiquitin-conjugating enzyme E2D 4 (putative)	0,0413275	0,894	0,0000313	0,571	0,733
244011_at	**PPM1K**	protein phosphatase 1K (PP2C domain containing)	0,0414048	0,886	0,0232773	0,427	0,656
228500_at	**THAP8**	THAP domain containing 8	0,0415768	0,730	0,0013263	0,608	0,669
240639_at	**FLJ12584**	NA	0,0416105	0,853	0,0014977	0,463	0,658
219253_at	**FAM11B**	family with sequence similarity 11, member B	0,0416833	0,844	0,0113898	0,824	0,834
214692_s_at	**JRK**	jerky homolog (mouse)	0,0417082	0,850	0,0455347	0,868	0,859
222474_s_at	**TOMM22**	translocase of outer mitochondrial membrane 22 homolog (yeast)	0,0417489	0,753	0,0445268	0,628	0,690
224762_at	**TDE2L**	tumor differentially expressed 2-like	0,0418846	0,752	0,0263774	0,583	0,667
218152_at	**HMG20A**	high-mobility group 20A	0,0418858	0,729	0,0126407	0,834	0,782
203466_at	**MPV17**	MpV17 transgene, murine homolog, glomerulosclerosis	0,0418947	0,764	0,0243770	0,774	0,769
200793_s_at	**ACO2**	aconitase 2, mitochondrial	0,0419945	0,850	0,0318599	0,828	0,839
201611_s_at	**ICMT**	isoprenylcysteine carboxyl methyltransferase	0,0420409	0,888	0,0341366	0,813	0,850
205761_s_at	**DUS4L**	dihydrouridine synthase 4-like (S. cerevisiae)	0,0420959	0,741	0,0274738	0,728	0,735
228889_at	**C14orf128**	chromosome 14 open reading frame 128	0,0421381	0,784	0,0084845	0,734	0,759
212564_at	**KCTD2**	potassium channel tetramerisation domain containing 2	0,0421469	0,891	0,0565337	0,878	0,885
202098_s_at	**HRMT1L1**	HMT1 hnRNP methyltransferase-like 1 (S. cerevisiae)	0,0421529	0,862	0,0121899	0,721	0,791
204497_at	**ADCY9**	adenylate cyclase 9	0,0421901	0,814	0,0365056	0,780	0,797
219925_at	**ZMYM6**	zinc finger, MYM-type 6	0,0422025	0,733	0,0101959	0,866	0,800
1569076_a_at	**FLJ16287**	NA	0,0422166	0,847	0,0044091	0,849	0,848
209857_s_at	**SPHK2**	sphingosine kinase 2	0,0423095	0,892	0,0004401	0,733	0,812
243807_at	**NCOA7**	nuclear receptor coactivator 7	0,0423547	0,834	0,0440193	0,756	0,795

202457_s_at	**PPP3CA**	protein phosphatase 3 (formerly 2B), catalytic subunit, alpha isoform (calcineurin A alpha)	0,0423741	0,733	0,0379790	0,813	0,773
227667_at	**CUEDC1**	CUE domain containing 1	0,0424082	0,864	0,0038475	0,746	0,805
209620_s_at	**ABCB7**	ATP-binding cassette, sub-family B (MDR/TAP), member 7	0,0424208	0,822	0,0144139	0,802	0,812
225096_at	**HSA272196**	NA	0,0424438	0,771	0,0584362	0,896	0,834
227113_at	**ADHFE1**	alcohol dehydrogenase, iron containing, 1	0,0425155	0,781	0,0023227	0,650	0,715
202330_s_at	**UNG**	uracil-DNA glycosylase	0,0425494	0,754	0,0276152	0,777	0,765
241795_at	**RHEB**	Ras homolog enriched in brain	0,0426775	0,891	0,0082989	0,819	0,855
219649_at	**ALG6**	asparagine-linked glycosylation 6 homolog (yeast, alpha-1,3-glucosyltransferase)	0,0427038	0,853	0,0567854	0,848	0,850
201815_s_at	**TBC1D5**	TBC1 domain family, member 5	0,0427426	0,791	0,0367224	0,701	0,746
235421_at	**MAP3K8**	mitogen-activated protein kinase kinase kinase 8	0,0428052	0,724	0,0095592	0,574	0,649
1558809_s_at	**LOC284408**	NA	0,0428224	0,796	0,0545379	0,769	0,782
223314_at	**TSPAN14**	tetraspanin 14	0,0428491	0,787	0,0086107	0,676	0,731
204518_s_at	**PPIC**	peptidylprolyl isomerase C (cyclophilin C)	0,0428603	0,845	0,0312959	0,858	0,851
219843_at	**IPP**	intracisternal A particle-promoted polypeptide	0,0428629	0,758	0,0209258	0,836	0,797
1555973_at	**FLJ39051**	NA	0,0428902	0,825	0,0060776	0,681	0,753
240011_at	**TTBK2**	tau tubulin kinase 2	0,0429026	0,838	0,0341527	0,644	0,741
215543_s_at	**LARGE**	like-glycosyltransferase	0,0429044	0,785	0,0256776	0,750	0,767
219530_at	**FLJ21816**	NA	0,0429098	0,795	0,0164081	0,762	0,779
218159_at	**C20orf116**	chromosome 20 open reading frame 116	0,0430258	0,849	0,0344139	0,867	0,858
210658_s_at	**GGA2**	golgi associated, gamma adaptin ear containing, ARF binding protein 2	0,0431863	0,843	0,0215414	0,761	0,802
224791_at	**DDEF1**	development and differentiation enhancing factor 1	0,0432396	0,873	0,0039891	0,694	0,784
216594_x_at	**AKR1C1**	c("aldo-keto reductase family 1, member C1 (dihydrodiol dehydrogenase 1", " 20-alpha (3-alpha)-hydroxysteroid dehydrogenase)")	0,0432583	0,425	0,0304220	0,327	0,376
205387_s_at	**CGB**	chorionic gonadotropin, beta polypeptide	0,0432592	0,849	0,0230733	0,838	0,844
213244_at	**SCAMP4**	secretory carrier membrane protein 4	0,0432608	0,755	0,0000000	0,497	0,626
209782_s_at	**DBP**	D site of albumin promoter (albumin D-box) binding protein	0,0433451	0,472	0,0011371	0,326	0,399
219405_at	**TRIM68**	tripartite motif-containing 68	0,0433451	0,843	0,0422931	0,866	0,854
235628_x_at	**LOC389323**	NA	0,0433824	0,807	0,0123904	0,724	0,765
224458_at	**C9orf125**	chromosome 9 open reading frame 125	0,0435149	0,852	0,0012157	0,619	0,735
204199_at	**RALGPS1**	Ral GEF with PH domain and SH3 binding motif 1	0,0435165	0,873	0,0116132	0,829	0,851
243796_at	**HEXIM2**	hexamthylene bis-acetamide inducible 2	0,0435648	0,912	0,0126580	0,709	0,810
222408_s_at	**YPEL5**	yippee-like 5 (Drosophila)	0,0435741	0,902	0,0452815	0,900	0,901
211591_s_at	**PDE4A**	phosphodiesterase 4A, cAMP-specific (phosphodiesterase E2 dunce homolog, Drosophila)	0,0440230	0,869	0,0269623	0,817	0,843
220316_at	**NPAS3**	neuronal PAS domain protein 3	0,0440741	0,905	0,0247572	0,770	0,837
203952_at	**ATF6**	activating transcription factor 6	0,0441374	0,897	0,0209779	0,646	0,771
244689_at	**LOC150383**	NA	0,0441374	0,772	0,0365940	0,773	0,772
212554_at	**CAP2**	CAP, adenylate cyclase-associated protein, 2 (yeast)	0,0443301	0,700	0,0111060	0,581	0,640
214852_x_at	**VPS13A**	vacuolar protein sorting 13A (yeast)	0,0443451	0,873	0,0083879	0,672	0,772
228365_at	**CPNE8**	copine VIII	0,0443521	0,617	0,0018123	0,531	0,574
221056_x_at	**EPS15L1**	epidermal growth factor receptor pathway substrate 15-like 1	0,0443900	0,863	0,0007902	0,704	0,784
229742_at	**LOC145853**	NA	0,0443900	0,802	0,0078105	0,713	0,757
221287_at	**RNASEL**	ribonuclease L (2',5'-oligoisoadenylate synthetase-dependent)	0,0445259	0,763	0,0197052	0,829	0,796
218537_at	**HCFC1R1**	host cell factor C1 regulator 1 (XPO1 dependant)	0,0446012	0,596	0,0019663	0,488	0,542
218494_s_at	**SLC2A4RG**	SLC2A4 regulator	0,0447687	0,809	0,0018035	0,681	0,745
242436_at	**PLEKHH2**	pleckstrin homology domain containing, family H (with MyTH4 domain) member 2	0,0448664	0,895	0,0519788	0,788	0,841
201178_at	**FBXO7**	F-box protein 7	0,0449584	0,867	0,0028602	0,812	0,840
1007_s_at	**DDR1**	discoidin domain receptor family, member 1	0,0449604	0,768	0,0318821	0,595	0,681
205095_s_at	**ATP6V0A1**	ATPase, H+ transporting, lysosomal V0 subunit a isoform 1	0,0450884	0,790	0,0000517	0,597	0,693
237026_at	**SORBS1**	sorbin and SH3 domain containing 1	0,0451435	0,901	0,0538869	0,812	0,856
221771_s_at	**HSMPP8**	NA	0,0452551	0,705	0,0065095	0,693	0,699
218724_s_at	**TGIF2**	TGFB-induced factor 2 (TALE family homeobox)	0,0452605	0,674	0,0375226	0,664	0,669
1555270_a_at	**WFS1**	Wolfram syndrome 1 (wolframin)	0,0452898	0,833	0,0019161	0,683	0,758
201380_at	**CRTAP**	cartilage associated protein	0,0453335	0,759	0,0187365	0,806	0,783
201717_at	**MRPL49**	mitochondrial ribosomal protein L49	0,0453531	0,855	0,0542122	0,867	0,861
202814_s_at	**HEXIM1**	hexamethylene bis-acetamide inducible 1	0,0453686	0,883	0,0554851	0,888	0,885
240269_at	**MGC16169**	NA	0,0454231	0,910	0,0380489	0,680	0,795
213279_at	**DHRS1**	dehydrogenase/reductase (SDR family) member 1	0,0454350	0,792	0,0511957	0,786	0,789
228813_at	**HDAC4**	histone deacetylase 4	0,0454363	0,638	0,0191943	0,658	0,648
203781_at	**MRPL33**	mitochondrial ribosomal protein L33	0,0454833	0,866	0,0278242	0,884	0,875

228305_at	**ZNF565**	zinc finger protein 565	0,0454869	0,808	0,0170865	0,813	0,810
235054_at	**NUDT16**	nudix (nucleoside diphosphate linked moiety X)-type motif 16	0,0455227	0,841	0,0044184	0,801	0,821
212054_x_at	**KIAA0676**	NA	0,0455510	0,881	0,0148603	0,786	0,834
237152_at	**MGC33657**	NA	0,0455510	0,903	0,0245279	0,777	0,840
213361_at	**TDRD7**	tudor domain containing 7	0,0456236	0,860	0,0487909	0,856	0,858
228366_at	**PPA2**	NA	0,0456373	0,794	0,0572316	0,927	0,860
203627_at	**IGF1R**	insulin-like growth factor 1 receptor	0,0456408	0,726	0,0216820	0,583	0,655
217984_at	**RNASET2**	ribonuclease T2	0,0457576	0,833	0,0449292	0,862	0,848
203695_s_at	**DFNA5**	deafness, autosomal dominant 5	0,0457596	0,749	0,0535448	0,644	0,697
227516_at	**SF3A1**	splicing factor 3a, subunit 1, 120kDa	0,0457596	0,888	0,0539310	0,756	0,822
228096_at	**C1orf151**	chromosome 1 open reading frame 151	0,0457859	0,823	0,0037277	0,679	0,751
38671_at	**PLXND1**	plexin D1	0,0458658	0,793	0,0126063	0,757	0,775
1553991_s_at	**FLJ20674**	NA	0,0458962	0,809	0,0066339	0,511	0,660
224560_at	**TIMP2**	TIMP metallopeptidase inhibitor 2	0,0459207	0,821	0,0009756	0,692	0,756
205618_at	**PRRG1**	proline rich Gla (G-carboxyglutamic acid) 1	0,0459516	0,755	0,0026107	0,724	0,740
236328_at	**ZNF285**	zinc finger protein 285	0,0459916	0,748	0,0055115	0,663	0,706
225043_at	**SLC15A4**	solute carrier family 15, member 4	0,0460860	0,818	0,0083638	0,811	0,815
219373_at	**DPM3**	dolichyl-phosphate mannosyltransferase polypeptide 3	0,0460904	0,769	0,0510138	0,691	0,730
231480_at	**SLC6A19**	solute carrier family 6 (neutral amino acid transporter), member 19	0,0461281	0,882	0,0305070	0,853	0,868
237050_at	**FARP2**	FERM, RhoGEF and pleckstrin domain protein 2	0,0461458	0,934	0,0344674	0,709	0,821
233348_at	**KIAA1423**	KIAA1423	0,0462876	0,793	0,0100444	0,766	0,780
227285_at	**C1orf51**	chromosome 1 open reading frame 51	0,0462979	0,770	0,0144381	0,598	0,684
214126_at	**MCART1**	mitochondrial carrier triple repeat 1	0,0463514	0,716	0,0004756	0,712	0,714
227431_at	**RSBN1L**	round spermatid basic protein 1-like	0,0465174	0,809	0,0398188	0,818	0,814
222452_s_at	**GPBP1L1**	GC-rich promoter binding protein 1-like 1	0,0465576	0,865	0,0006119	0,816	0,840
221218_s_at	**TPK1**	thiamin pyrophosphokinase 1	0,0465587	0,806	0,0168675	0,647	0,726
1555793_a_at	**ZNF545**	zinc finger protein 545	0,0465587	0,837	0,0588103	0,768	0,802
227461_at	**STN2**	NA	0,0465806	0,231	0,0319679	0,304	0,267
209703_x_at	**DKFZP586A0522**	NA	0,0466093	0,762	0,0576780	0,778	0,770
208317_at	**XYLB**	xylulokinase homolog (H. influenzae)	0,0466314	0,885	0,0540989	0,882	0,884
235133_at	**ZDHHC14**	zinc finger, DHHC-type containing 14	0,0466875	0,832	0,0475075	0,754	0,793
218377_s_at	**C21orf6**	chromosome 21 open reading frame 6	0,0466912	0,854	0,0045679	0,796	0,825
202754_at	**R3HDM1**	R3H domain containing 1	0,0466912	0,872	0,0406195	0,900	0,886
244389_at	**TLK1**	tousled-like kinase 1	0,0467614	0,802	0,0522337	0,919	0,861
224159_x_at	**TRIM4**	tripartite motif-containing 4	0,0467660	0,814	0,0354772	0,740	0,777
205451_at	**MLLT7**	c("myeloid/lymphoid or mixed-lineage leukemia (trithorax homolog, Drosophila)", " translocated to, 7")	0,0467751	0,828	0,0243910	0,763	0,795
226604_at	**SMILE**	NA	0,0468097	0,828	0,0544445	0,804	0,816
204037_at	**EDG2**	endothelial differentiation, lysophosphatidic acid G-protein-coupled receptor, 2	0,0468962	0,667	0,0318744	0,596	0,631
210908_s_at	**PFDN5**	prefoldin 5	0,0469909	0,783	0,0582163	0,873	0,828
204800_s_at	**FLJ13639**	NA	0,0470312	0,743	0,0128700	0,750	0,746
233123_at	**SLC40A1**	solute carrier family 40 (iron-regulated transporter), member 1	0,0470670	0,851	0,0449463	0,135	0,493
232323_s_at	**TTC17**	tetratricopeptide repeat domain 17	0,0471303	0,869	0,0258463	0,765	0,817
202158_s_at	**CUGBP2**	CUG triplet repeat, RNA binding protein 2	0,0471389	0,725	0,0154509	0,883	0,804
244071_at	**LOC345630**	NA	0,0473357	0,726	0,0249006	0,732	0,729
205252_at	**ZNF174**	zinc finger protein 174	0,0473357	0,784	0,0005687	0,747	0,765
222873_s_at	**EHMT1**	euchromatic histone-lysine N-methyltransferase 1	0,0474833	0,800	0,0264527	0,860	0,830
238210_at	**RYK**	RYK receptor-like tyrosine kinase	0,0475547	0,880	0,0275936	0,818	0,849
217485_x_at	**PMS2L1**	postmeiotic segregation increased 2-like 1	0,0475999	0,886	0,0208905	0,861	0,873
227139_s_at	**HPS3**	Hermansky-Pudlak syndrome 3	0,0476417	0,854	0,0325412	0,778	0,816
218338_at	**PHC1**	polyhomeotic-like 1 (Drosophila)	0,0476561	0,739	0,0519739	0,786	0,763
225897_at	**MARCKS**	myristoylated alanine-rich protein kinase C substrate	0,0476916	0,774	0,0232089	0,655	0,715
212933_x_at	**RPL13**	ribosomal protein L13	0,0476916	0,902	0,0229335	0,768	0,835
200817_x_at	**RPS10**	ribosomal protein S10	0,0477133	0,925	0,0086768	0,938	0,932
212645_x_at	**BRE**	brain and reproductive organ-expressed (TNFRSF1A modulator)	0,0477973	0,821	0,0148522	0,789	0,805
202020_s_at	**LANCL1**	LanC lantibiotic synthetase component C-like 1 (bacterial)	0,0477988	0,805	0,0474450	0,843	0,824
225606_at	**BCL2L11**	BCL2-like 11 (apoptosis facilitator)	0,0479251	0,299	0,0453474	0,423	0,361
227013_at	**LATS2**	LATS, large tumor suppressor, homolog 2 (Drosophila)	0,0479656	0,828	0,0214208	0,598	0,713
221064_s_at	**C16orf28**	chromosome 16 open reading frame 28	0,0479966	0,790	0,0250728	0,772	0,781
228356_at	**ANKRD11**	ankyrin repeat domain 11	0,0480319	0,902	0,0351728	0,823	0,863
240126_x_at	**FALZ**	fetal Alzheimer antigen	0,0480319	0,942	0,0251854	0,783	0,863
221006_s_at	**SNX27**	sorting nexin family member 27	0,0481561	0,781	0,0284280	0,763	0,772
214889_at	**DKFZP564J102**	NA	0,0481780	0,516	0,0339893	0,547	0,531
219730_at	**MED18**	mediator of RNA polymerase II transcription, subunit 18 homolog (yeast)	0,0482018	0,701	0,0396632	0,639	0,670

213812_s_at	**CAMKK2**	calcium/calmodulin-dependent protein kinase kinase 2, beta	0,0482545	0,813	0,0083297	0,811	0,812
230736_at	**LOC387647**	NA	0,0482560	0,795	0,0232089	0,656	0,726
204556_s_at	**DZIP1**	DAZ interacting protein 1	0,0483288	0,793	0,0265994	0,690	0,741
231874_at	**MGC39518**	NA	0,0483294	0,583	0,0099975	0,687	0,635
241618_at	**PACS1**	phosphofurin acidic cluster sorting protein 1	0,0483548	0,851	0,0008152	0,707	0,779
214991_s_at	**PIGO**	phosphatidylinositol glycan, class O	0,0483548	0,834	0,0157279	0,836	0,835
205610_at	**MYOM1**	myomesin 1 (skelemin) 185kDa	0,0484352	0,723	0,0055320	0,719	0,721
212160_at	**XPOT**	exportin, tRNA (nuclear export receptor for tRNAs)	0,0484737	0,813	0,0359214	0,721	0,767
213110_s_at	**COL4A5**	collagen, type IV, alpha 5 (Alport syndrome)	0,0484775	0,727	0,0476080	0,413	0,570
232965_at	**LOC400684**	NA	0,0484853	0,756	0,0012460	0,589	0,672
218784_s_at	**C6orf64**	chromosome 6 open reading frame 64	0,0485108	0,775	0,0174822	0,791	0,783
200022_at	**RPL18**	ribosomal protein L18	0,0485108	0,904	0,0463153	0,913	0,909
224162_s_at	**FBXO31**	F-box protein 31	0,0485343	0,844	0,0023061	0,769	0,807
218452_at	**SMARCAL1**	SWI/SNF related, matrix associated, actin dependent regulator of chromatin, subfamily a-like 1	0,0485634	0,867	0,0075737	0,808	0,837
225407_at	**MBP**	myelin basic protein	0,0486592	0,747	0,0261162	0,337	0,542
201214_s_at	**PPP1R7**	protein phosphatase 1, regulatory subunit 7	0,0486605	0,778	0,0060299	0,833	0,805
1553538_s_at	**LOC440551**	NA	0,0487070	0,766	0,0000000	0,691	0,729
239020_at	**FUT10**	fucosyltransferase 10 (alpha (1,3) fucosyltransferase)	0,0487240	0,840	0,0317851	0,675	0,757
241869_at	**APOL6**	apolipoprotein L, 6	0,0488464	0,393	0,0267294	0,302	0,347
244462_at	**ZNF224**	zinc finger protein 224	0,0488551	0,819	0,0033122	0,679	0,749
226685_at	**SNTB2**	syntrophin, beta 2 (dystrophin-associated protein A1, 59kDa, basic component 2)	0,0490242	0,757	0,0539166	0,821	0,789
221812_at	**FBXO42**	F-box protein 42	0,0491148	0,875	0,0315066	0,854	0,865
239712_at	**C9orf93**	chromosome 9 open reading frame 93	0,0491179	0,922	0,0218582	0,852	0,887
205212_s_at	**CENTB1**	centaurin, beta 1	0,0491251	0,789	0,0232153	0,793	0,791
226954_at	**UBE2R2**	ubiquitin-conjugating enzyme E2R 2	0,0491829	0,847	0,0408231	0,797	0,822
206854_s_at	**MAP3K7**	mitogen-activated protein kinase kinase kinase 7	0,0491967	0,854	0,0015840	0,721	0,788
1564387_at	**KIAA1117**	KIAA1117	0,0492215	0,937	0,0562302	0,835	0,886
1553815_a_at	**MGC17403**	NA	0,0492494	0,716	0,0586038	0,800	0,758
204476_s_at	**PC**	pyruvate carboxylase	0,0492535	0,733	0,0046394	0,576	0,654
1558378_a_at	**C14orf78**	chromosome 14 open reading frame 78	0,0493401	0,819	0,0237812	0,375	0,597
227432_s_at	**INSR**	insulin receptor	0,0493585	0,712	0,0006924	0,728	0,720
1559952_x_at	**LOC441340**	NA	0,0493711	0,818	0,0322325	0,848	0,833
202249_s_at	**WDR42A**	WD repeat domain 42A	0,0494271	0,834	0,0345016	0,754	0,794
204570_at	**COX7A1**	cytochrome c oxidase subunit VIIa polypeptide 1 (muscle)	0,0494651	0,861	0,0260360	0,743	0,802
222621_at	**DNAJC1**	DnaJ (Hsp40) homolog, subfamily C, member 1	0,0494859	0,824	0,0469181	0,748	0,786
222713_s_at	**FANCF**	Fanconi anemia, complementation group F	0,0494859	0,800	0,0514450	0,815	0,808
206683_at	**ZNF165**	zinc finger protein 165	0,0494859	0,847	0,0134988	0,788	0,817
233936_s_at	**ZNF403**	zinc finger protein 403	0,0494859	0,854	0,0217361	0,866	0,860
1554241_at	**COCH**	coagulation factor C homolog, cochlin (Limulus polyphemus)	0,0495189	0,723	0,0369936	0,778	0,750
204485_s_at	**TOM1L1**	target of myb1-like 1 (chicken)	0,0495189	0,727	0,0320386	0,816	0,771
229676_at	**PAPD1**	PAP associated domain containing 1	0,0495415	0,673	0,0489770	0,808	0,740
1565149_at	**DNCH2**	dynein, cytoplasmic, heavy polypeptide 2	0,0495578	0,715	0,0540921	0,746	0,730
206481_s_at	**LDB2**	LIM domain binding 2	0,0495578	0,691	0,0178502	0,421	0,556
222883_at	**C1orf163**	chromosome 1 open reading frame 163	0,0495596	0,743	0,0542469	0,834	0,788
218312_s_at	**ZNF447**	zinc finger protein 447	0,0495993	0,797	0,0081949	0,690	0,743
223404_s_at	**C1orf25**	chromosome 1 open reading frame 25	0,0496202	0,763	0,0347045	0,736	0,749
224990_at	**LOC201895**	NA	0,0496511	0,837	0,0469600	0,659	0,748
223442_at	**NICN1**	nicolin 1	0,0497297	0,742	0,0002773	0,647	0,695
212793_at	**DAAM2**	dishevelled associated activator of morphogenesis 2	0,0497424	0,666	0,0204257	0,544	0,605
227603_at	**HKR1**	GLI-Kruppel family member HKR1	0,0497731	0,726	0,0453452	0,824	0,775
219135_s_at	**FLJ12681**	NA	0,0498362	0,823	0,0235591	0,695	0,759
224877_s_at	**MRPS5**	mitochondrial ribosomal protein S5	0,0498569	0,783	0,0049413	0,818	0,801
230364_at	**CHPT1**	choline phosphotransferase 1	0,0498844	0,815	0,0496693	0,720	0,768
222169_x_at	**SH2D3A**	SH2 domain containing 3A	0,0498844	0,881	0,0014751	0,762	0,821
228885_at	**MAMDC2**	MAM domain containing 2	0,0499355	0,422	0,0419263	0,431	0,427
219041_s_at	**REPIN1**	replication initiator 1	0,0499518	0,713	0,0466452	0,888	0,800
229518_at	**FAM46B**	family with sequence similarity 46, member B	0,0499631	0,537	0,0320467	0,586	0,562
222272_x_at	**SCIN**	scinderin	0,0499952	0,882	0,0269623	0,220	0,551
227319_at	**C16orf44**	chromosome 16 open reading frame 44	0,0500409	0,828	0,0140354	0,863	0,846
225334_at	**C10orf32**	chromosome 10 open reading frame 32	0,0500792	0,804	0,0299495	0,761	0,782
202708_s_at	**HIST2H2BE**	histone 2, H2be	0,0500792	0,718	0,0287719	0,679	0,699
224764_at	**ARHGAP21**	Rho GTPase activating protein 21	0,0501755	0,787	0,0399471	0,743	0,765
219340_s_at	**CLN8**	ceroid-lipofuscinosis, neuronal 8 (epilepsy, progressive with mental retardation)	0,0501967	0,807	0,0214208	0,477	0,642
215123_at	**LOC348162**	NA	0,0501967	0,704	0,0503083	0,598	0,651
216163_at	**HBLD2**	HESB like domain containing 2	0,0502302	0,859	0,0447095	0,850	0,855
235422_at	**LOC440461**	NA	0,0502401	0,784	0,0522064	0,774	0,779

212841_s_at	**PPFIBP2**	PTPRF interacting protein, binding protein 2 (liprin beta 2)	0,0502442	0,718	0,0341657	0,663	0,691
241858_at	**FPGT**	fucose-1-phosphate guanylyltransferase	0,0502536	0,816	0,0171692	0,799	0,808
203562_at	**FEZ1**	fasciculation and elongation protein zeta 1 (zygin I)	0,0502543	0,700	0,0244185	0,679	0,689
212147_at	**EST1B**	NA	0,0502771	0,891	0,0024650	0,842	0,867
229685_at	**TBC1D14**	TBC1 domain family, member 14	0,0502771	0,819	0,0123074	0,714	0,767
1558896_at	**C1orf69**	chromosome 1 open reading frame 69	0,0505462	0,776	0,0222592	0,701	0,738
227778_at	**KIAA1833**	NA	0,0505942	0,857	0,0003594	0,693	0,775
209107_x_at	**NCOA1**	nuclear receptor coactivator 1	0,0506077	0,852	0,0323624	0,713	0,783
229003_x_at	**FAM69B**	family with sequence similarity 69, member B	0,0506224	0,914	0,0486956	0,906	0,910
224614_at	**DNCLI2**	dynein, cytoplasmic, light intermediate polypeptide 2	0,0506536	0,887	0,0564752	0,801	0,844
228190_at	**ATG4C**	ATG4 autophagy related 4 homolog C (S. cerevisiae)	0,0507455	0,718	0,0527692	0,801	0,759
223125_s_at	**C1orf21**	chromosome 1 open reading frame 21	0,0508122	0,705	0,0517838	0,908	0,807
220182_at	**SLC25A23**	c("solute carrier family 25 (mitochondrial carrier", " phosphate carrier), member 23")	0,0508633	0,733	0,0025430	0,554	0,644
226240_at	**MGC21874**	NA	0,0509465	0,880	0,0096306	0,858	0,869
236846_at	**LOC284757**	NA	0,0509512	0,547	0,0293741	0,495	0,521
209346_s_at	**PI4KII**	NA	0,0511323	0,746	0,0319679	0,809	0,778
214755_at	**UAP1L1**	UDP-N-acteylglucosamine pyrophosphorylase 1-like 1	0,0511576	0,701	0,0007019	0,497	0,599
203752_s_at	**JUND**	jun D proto-oncogene	0,0511742	0,853	0,0379063	0,715	0,784
234228_at	**NFIA**	nuclear factor I/A	0,0512619	0,909	0,0382123	0,567	0,738
225721_at	**SYNPO2**	synaptopodin 2	0,0512634	0,583	0,0174320	0,423	0,503
225458_at	**DKFZP564I1171**	NA	0,0513298	0,826	0,0046860	0,698	0,762
218552_at	**ECHDC2**	enoyl Coenzyme A hydratase domain containing 2	0,0513941	0,823	0,0430996	0,755	0,789
200991_s_at	**SNX17**	sorting nexin 17	0,0514180	0,895	0,0432350	0,835	0,865
222011_s_at	**ACAT2**	acetyl-Coenzyme A acetyltransferase 2 (acetoacetyl Coenzyme A thiolase)	0,0514192	0,766	0,0027369	0,480	0,623
1553960_at	**C20orf161**	chromosome 20 open reading frame 161	0,0516962	0,792	0,0301493	0,582	0,687
210589_s_at	**GBA**	c("glucosidase, beta", " acid (includes glucosylceramidase)")	0,0517154	0,774	0,0037277	0,644	0,709
227316_at	**CSRP2BP**	CSRP2 binding protein	0,0517464	0,888	0,0398454	0,807	0,847
229962_at	**FLJ34306**	NA	0,0517464	0,817	0,0242338	0,774	0,796
212244_at	**GRINL1A**	glutamate receptor, ionotropic, N-methyl D-aspartate-like 1A	0,0517931	0,822	0,0153941	0,788	0,805
205238_at	**CXorf34**	chromosome X open reading frame 34	0,0518085	0,647	0,0004756	0,598	0,623
213119_at	**SLC36A1**	solute carrier family 36 (proton/amino acid symporter), member 1	0,0518752	0,712	0,0428038	0,780	0,746
224280_s_at	**FAM54B**	family with sequence similarity 54, member B	0,0519585	0,896	0,0573546	0,855	0,876
241372_at	**ZC3H6**	zinc finger CCCH-type containing 6	0,0519585	0,778	0,0073273	0,759	0,768
243026_x_at	**BIRC4**	baculoviral IAP repeat-containing 4	0,0519909	0,813	0,0048246	0,736	0,775
223199_at	**MKNK2**	MAP kinase interacting serine/threonine kinase 2	0,0520658	0,675	0,0002773	0,497	0,586
225437_s_at	**C7orf27**	chromosome 7 open reading frame 27	0,0522577	0,858	0,0091542	0,756	0,807
214887_at	**CG018**	NA	0,0522753	0,920	0,0488809	0,414	0,667
212229_s_at	**FBXO21**	F-box protein 21	0,0523208	0,842	0,0142126	0,862	0,852
224902_at	**PDPR**	NA	0,0523629	0,785	0,0354872	0,806	0,796
1552664_at	**FLCN**	folliculin	0,0525628	0,662	0,0418108	0,757	0,709
214565_s_at	**SMR3A**	submaxillary gland androgen regulated protein 3 homolog A (mouse)	0,0525628	0,918	0,0217408	0,908	0,913
220387_s_at	**HHLA3**	HERV-H LTR-associating 3	0,0525753	0,655	0,0204257	0,594	0,624
221111_at	**IL26**	interleukin 26	0,0526302	0,184	0,0178367	0,158	0,171
223960_s_at	**C16orf5**	chromosome 16 open reading frame 5	0,0527692	0,867	0,0002773	0,681	0,774
210570_x_at	**MAPK9**	mitogen-activated protein kinase 9	0,0527692	0,886	0,0021023	0,836	0,861
204024_at	**C8orf1**	chromosome 8 open reading frame 1	0,0528060	0,791	0,0256177	0,924	0,857
210610_at	**CEACAM1**	carcinoembryonic antigen-related cell adhesion molecule 1 (biliary glycoprotein)	0,0528060	0,865	0,0597366	0,890	0,877
219586_at	**WDR59**	WD repeat domain 59	0,0528060	0,845	0,0295567	0,774	0,810
218077_s_at	**ZDHHC3**	zinc finger, DHHC-type containing 3	0,0528963	0,821	0,0247341	0,769	0,795
211863_x_at	**HFE**	hemochromatosis	0,0529263	0,810	0,0028602	0,846	0,828
209332_s_at	**MAX**	MYC associated factor X	0,0529375	0,888	0,0418108	0,861	0,875
219923_at	**TRIM45**	tripartite motif-containing 45	0,0529612	0,857	0,0267961	0,706	0,781
228044_at	**C13orf21**	chromosome 13 open reading frame 21	0,0530524	0,838	0,0112118	0,855	0,846
235697_at	**ZNF544**	zinc finger protein 544	0,0530795	0,824	0,0429917	0,830	0,827
216241_s_at	**TCEA1**	transcription elongation factor A (SII), 1	0,0531226	0,802	0,0070029	0,737	0,770
203421_at	**TP53I11**	tumor protein p53 inducible protein 11	0,0531231	0,800	0,0002160	0,378	0,589
201418_s_at	**SOX4**	SRY (sex determining region Y)-box 4	0,0532067	0,607	0,0332986	0,403	0,505
202371_at	**TCEAL4**	transcription elongation factor A (SII)-like 4	0,0532173	0,820	0,0236144	0,885	0,852
209494_s_at	**ZNF278**	zinc finger protein 278	0,0532525	0,826	0,0371740	0,664	0,745
203341_at	**CEBPZ**	CCAAT/enhancer binding protein zeta	0,0533449	0,873	0,0245690	0,598	0,736
235684_s_at	**SESN3**	sestrin 3	0,0533769	0,729	0,0418689	0,451	0,590
231423_s_at	**ANKRD16**	ankyrin repeat domain 16	0,0534120	0,892	0,0158747	0,782	0,837
201347_x_at	**GRHPR**	glyoxylate reductase/hydroxypyruvate reductase	0,0534545	0,871	0,0113371	0,864	0,868

203659_s_at	**RFP2**	ret finger protein 2	0,0534545	0,904	0,0398208	0,722	0,813
211448_s_at	**RGS6**	regulator of G-protein signalling 6	0,0534545	0,926	0,0144847	0,778	0,852
208610_s_at	**SRRM2**	serine/arginine repetitive matrix 2	0,0534545	0,714	0,0328367	0,672	0,693
218525_s_at	**HIF1AN**	hypoxia-inducible factor 1, alpha subunit inhibitor	0,0534998	0,894	0,0338219	0,877	0,885
224804_s_at	**C15orf17**	chromosome 15 open reading frame 17	0,0535224	0,868	0,0338742	0,766	0,817
1565595_at	**SLC6A16**	solute carrier family 6, member 16	0,0535390	0,872	0,0334464	0,874	0,873
213689_x_at	**FAM69A**	family with sequence similarity 69, member A	0,0536231	0,673	0,0413340	0,716	0,695
223592_s_at	**RNF135**	ring finger protein 135	0,0536329	0,817	0,0227970	0,821	0,819
206110_at	**HIST1H3H**	histone 1, H3h	0,0536411	0,803	0,0045186	0,715	0,759
218361_at	**GOLPH3L**	golgi phosphoprotein 3-like	0,0536918	0,871	0,0277243	0,769	0,820
203038_at	**PTPRK**	protein tyrosine phosphatase, receptor type, K	0,0536918	0,722	0,0547471	0,697	0,710
230663_at	**FMNL2**	formin-like 2	0,0537140	0,919	0,0378889	0,480	0,700
233724_at	**ARNT**	aryl hydrocarbon receptor nuclear translocator	0,0537999	0,909	0,0395334	0,617	0,763
237878_at	**MAP2K2**	mitogen-activated protein kinase kinase 2	0,0538651	0,886	0,0095671	0,521	0,704
220183_s_at	**NUDT6**	nudix (nucleoside diphosphate linked moiety X)-type motif 6	0,0539215	0,785	0,0241266	0,752	0,768
226590_at	**PFN4**	profilin family, member 4	0,0539215	0,743	0,0008312	0,538	0,640
235344_at	**PPM1A**	protein phosphatase 1A (formerly 2C), magnesium-dependent, alpha isoform	0,0539239	0,799	0,0323812	0,857	0,828
223283_s_at	**SDCCAG33**	serologically defined colon cancer antigen 33	0,0539641	0,731	0,0233158	0,621	0,676
206259_at	**PROC**	protein C (inactivator of coagulation factors Va and VIIIa)	0,0539962	0,837	0,0513316	0,694	0,766
203338_at	**PPP2R5E**	protein phosphatase 2, regulatory subunit B (B56), epsilon isoform	0,0540011	0,839	0,0472037	0,725	0,782
229546_at	**FAM84A**	family with sequence similarity 84, member A	0,0541189	0,380	0,0365558	0,855	0,618
204056_s_at	**MVK**	mevalonate kinase (mevalonic aciduria)	0,0541296	0,825	0,0033081	0,784	0,804
205620_at	**F10**	coagulation factor X	0,0541744	0,833	0,0456838	0,846	0,839
202756_s_at	**GPC1**	glypican 1	0,0541744	0,753	0,0063528	0,576	0,665
228259_s_at	**TIGA1**	NA	0,0541744	0,778	0,0129047	0,642	0,710
218844_at	**FLJ20920**	NA	0,0541855	0,670	0,0059597	0,634	0,652
225735_at	**KIAA1223**	NA	0,0541855	0,811	0,0171393	0,774	0,792
212508_at	**MOAP1**	modulator of apoptosis 1	0,0542750	0,773	0,0007188	0,791	0,782
206782_s_at	**DNAJC4**	DnaJ (Hsp40) homolog, subfamily C, member 4	0,0542830	0,743	0,0090677	0,742	0,742
209153_s_at	**TCF3**	transcription factor 3 (E2A immunoglobulin enhancer binding factors E12/E47)	0,0545419	0,806	0,0187881	0,877	0,842
214284_s_at	**FGF18**	fibroblast growth factor 18	0,0545920	0,924	0,0522337	0,849	0,887
212039_x_at	**RPL3**	ribosomal protein L3	0,0546037	0,911	0,0453032	0,932	0,921
227781_x_at	**FAM57B**	family with sequence similarity 57, member B	0,0546090	0,833	0,0277977	0,794	0,813
202260_s_at	**STXBP1**	syntaxin binding protein 1	0,0546168	0,844	0,0467125	0,828	0,836
206128_at	**ADRA2C**	adrenergic, alpha-2C-, receptor	0,0546193	0,758	0,0424829	0,683	0,720
209868_s_at	**RBMS1**	RNA binding motif, single stranded interacting protein 1	0,0547242	0,741	0,0444022	0,741	0,741
217734_s_at	**WDR6**	WD repeat domain 6	0,0547242	0,770	0,0372399	0,796	0,783
225876_at	**NPAL3**	NIPA-like domain containing 3	0,0547341	0,766	0,0529298	0,596	0,681
213810_s_at	**C6orf166**	chromosome 6 open reading frame 166	0,0547635	0,800	0,0330219	0,860	0,830
204875_s_at	**GMDS**	GDP-mannose 4,6-dehydratase	0,0547635	0,645	0,0051512	0,750	0,697
207124_s_at	**GNB5**	guanine nucleotide binding protein (G protein), beta 5	0,0547813	0,838	0,0516340	0,922	0,880
203680_at	**PRKAR2B**	protein kinase, cAMP-dependent, regulatory, type II, beta	0,0547813	0,537	0,0530265	0,597	0,567
204274_at	**EBAG9**	estrogen receptor binding site associated, antigen, 9	0,0548104	0,821	0,0334516	0,787	0,804
228816_at	**LOC92270**	NA	0,0548484	0,596	0,0345111	0,540	0,568
238564_at	**KIAA1946**	KIAA1946	0,0550120	0,912	0,0053203	0,567	0,739
225864_at	**FAM84B**	family with sequence similarity 84, member B	0,0550489	0,363	0,0213325	0,482	0,422
211555_s_at	**GUCY1B3**	guanylate cyclase 1, soluble, beta 3	0,0550625	0,402	0,0206710	0,451	0,426
224718_at	**SLC25A29**	solute carrier family 25, member 29	0,0551126	0,815	0,0283205	0,852	0,833
214223_at	**PTP4A3**	protein tyrosine phosphatase type IVA, member 3	0,0551151	0,885	0,0387916	0,816	0,850
233651_s_at	**FLJ14981**	NA	0,0552063	0,799	0,0026431	0,765	0,782
204590_x_at	**VPS33A**	vacuolar protein sorting 33A (yeast)	0,0554606	0,880	0,0303801	0,857	0,869
244697_at	**ZBTB16**	zinc finger and BTB domain containing 16	0,0555185	0,898	0,0166681	0,899	0,899
204388_s_at	**MAOA**	monoamine oxidase A	0,0555359	0,607	0,0373698	0,408	0,507
1568594_s_at	**TRIM52**	tripartite motif-containing 52	0,0556562	0,752	0,0262701	0,766	0,759
220382_s_at	**ARHGAP28**	Rho GTPase activating protein 28	0,0557564	0,686	0,0380364	0,503	0,595
206617_s_at	**RENBP**	renin binding protein	0,0559056	0,604	0,0007019	0,351	0,478
200031_s_at	**RPS11**	ribosomal protein S11	0,0559056	0,941	0,0382123	0,733	0,837
209375_at	**XPC**	xeroderma pigmentosum, complementation group C	0,0559056	0,753	0,0353321	0,698	0,725
221475_s_at	**RPL15**	ribosomal protein L15	0,0559175	0,897	0,0151399	0,762	0,829
237766_at	**ATP9B**	ATPase, Class II, type 9B	0,0559246	0,876	0,0058157	0,838	0,857
226643_s_at	**NUDCD2**	NudC domain containing 2	0,0559770	0,777	0,0396143	0,926	0,851
226439_s_at	**NBEA**	neurobeachin	0,0560933	0,686	0,0390077	0,672	0,679
205376_at	**INPP4B**	inositol polyphosphate-4-phosphatase, type II, 105kDa	0,0562018	0,709	0,0455645	0,623	0,666
204453_at	**ZNF84**	zinc finger protein 84 (HPF2)	0,0563331	0,828	0,0392819	0,676	0,752

241682_at	**KLHL23**	kelch-like 23 (Drosophila)	0,0563593	0,843	0,0216529	0,550	0,696
233380_s_at	**RUFY1**	RUN and FYVE domain containing 1	0,0563657	0,877	0,0100283	0,835	0,856
226583_at	**FLJ40142**	NA	0,0563849	0,871	0,0334031	0,663	0,767
238966_at	**BRUNOL4**	bruno-like 4, RNA binding protein (Drosophila)	0,0564384	0,919	0,0468093	0,857	0,888
202788_at	**MAPKAPK3**	mitogen-activated protein kinase-activated protein kinase 3	0,0566145	0,765	0,0460229	0,801	0,783
218259_at	**MKL2**	MKL/myocardin-like 2	0,0568033	0,798	0,0304320	0,816	0,807
1555830_s_at	**FAM62B**	family with sequence similarity 62 (C2 domain containing) member B	0,0568331	0,592	0,0082989	0,770	0,681
203272_s_at	**TUSC2**	tumor suppressor candidate 2	0,0569556	0,853	0,0204255	0,866	0,859
203825_at	**BRD3**	bromodomain containing 3	0,0570165	0,854	0,0235504	0,874	0,864
208919_s_at	**NADK**	NAD kinase	0,0570211	0,853	0,0075743	0,807	0,830
225403_at	**C9orf23**	chromosome 9 open reading frame 23	0,0570310	0,842	0,0479329	0,889	0,865
223941_at	**FBXO24**	F-box protein 24	0,0570648	0,879	0,0187582	0,878	0,878
205727_at	**TEP1**	telomerase-associated protein 1	0,0570886	0,775	0,0000313	0,612	0,693
218914_at	**C1orf66**	chromosome 1 open reading frame 66	0,0571319	0,767	0,0000313	0,621	0,694
209723_at	**SERPINB9**	serpin peptidase inhibitor, clade B (ovalbumin), member 9	0,0572227	0,588	0,0455824	0,761	0,675
216484_x_at	**HDGF**	hepatoma-derived growth factor (high-mobility group protein 1-like)	0,0572554	0,912	0,0127040	0,812	0,862
1559227_s_at	**VHL**	von Hippel-Lindau tumor suppressor	0,0572969	0,663	0,0004756	0,592	0,627
205145_s_at	**MYL5**	myosin, light polypeptide 5, regulatory	0,0574253	0,770	0,0000000	0,691	0,731
212655_at	**ZCCHC14**	zinc finger, CCHC domain containing 14	0,0575435	0,744	0,0102898	0,784	0,764
203384_s_at	**GOLGA1**	golgi autoantigen, golgin subfamily a, 1	0,0576245	0,881	0,0043096	0,790	0,835
239682_at	**FLJ23861**	NA	0,0576845	0,701	0,0463754	0,729	0,715
240056_at	**NSMAF**	neutral sphingomyelinase (N-SMase) activation associated factor	0,0577784	0,886	0,0192468	0,654	0,770
203959_s_at	**ZBTB40**	zinc finger and BTB domain containing 40	0,0577784	0,875	0,0066543	0,837	0,856
226072_at	**FUK**	fucokinase	0,0578103	0,875	0,0483236	0,875	0,875
206532_at	**SMARCB1**	SWI/SNF related, matrix associated, actin dependent regulator of chromatin, subfamily b, member 1	0,0578141	0,850	0,0010893	0,720	0,785
221904_at	**MGC21688**	NA	0,0578822	0,793	0,0135597	0,680	0,737
214434_at	**HSPA12A**	heat shock 70kDa protein 12A	0,0578995	0,857	0,0012460	0,696	0,777
226655_at	**STX17**	syntaxin 17	0,0580101	0,796	0,0384583	0,832	0,814
226143_at	**RAI1**	retinoic acid induced 1	0,0580168	0,817	0,0066999	0,752	0,784
201984_s_at	**EGFR**	epidermal growth factor receptor (erythroblastic leukemia viral (v-erb-b) oncogene homolog, avian)	0,0580563	0,701	0,0275008	0,595	0,648
237324_s_at	**HKDC1**	hexokinase domain containing 1	0,0581084	0,852	0,0339640	0,866	0,859
239371_at	**FOXK2**	forkhead box K2	0,0581829	0,940	0,0000517	0,783	0,862
201368_at	**ZFP36L2**	zinc finger protein 36, C3H type-like 2	0,0581916	0,712	0,0132356	0,574	0,643
216941_s_at	**TAF1B**	TATA box binding protein (TBP)-associated factor, RNA polymerase I, B, 63kDa	0,0582494	0,817	0,0578948	0,763	0,790
212895_s_at	**ABR**	active BCR-related gene	0,0583210	0,784	0,0137538	0,798	0,791
212638_s_at	**WWP1**	WW domain containing E3 ubiquitin protein ligase 1	0,0584010	0,754	0,0228996	0,805	0,779
207635_s_at	**KCNH1**	potassium voltage-gated channel, subfamily H (eag-related), member 1	0,0584704	0,894	0,0395334	0,734	0,814
222156_x_at	**CCPG1**	cell cycle progression 1	0,0584767	0,694	0,0187907	0,634	0,664
224943_at	**BTBD7**	BTB (POZ) domain containing 7	0,0584784	0,751	0,0495875	0,897	0,824
200903_s_at	**AHCY**	S-adenosylhomocysteine hydrolase	0,0585354	0,875	0,0223195	0,790	0,833
223917_s_at	**SLC39A3**	solute carrier family 39 (zinc transporter), member 3	0,0586060	0,915	0,0448849	0,858	0,886
225184_at	**ARID1B**	AT rich interactive domain 1B (SWI1-like)	0,0586141	0,883	0,0396643	0,936	0,910
226646_at	**KLF2**	Kruppel-like factor 2 (lung)	0,0586223	0,901	0,0070222	0,738	0,820
41644_at	**SASH1**	SAM and SH3 domain containing 1	0,0586318	0,670	0,0236325	0,545	0,608
230730_at	**SGCD**	sarcoglycan, delta (35kDa dystrophin-associated glycoprotein)	0,0587732	0,588	0,0281208	0,784	0,686
223004_s_at	**C3orf1**	chromosome 3 open reading frame 1	0,0589939	0,794	0,0155656	0,793	0,794
202981_x_at	**SIAH1**	seven in absentia homolog 1 (Drosophila)	0,0589939	0,748	0,0497596	0,903	0,825
200620_at	**TMEM59**	transmembrane protein 59	0,0590155	0,881	0,0409808	0,887	0,884
223668_at	**CBLC**	Cas-Br-M (murine) ecotropic retroviral transforming sequence c	0,0591108	0,882	0,0450146	0,825	0,853
215058_at	**MGC24039**	NA	0,0593536	0,748	0,0214906	0,673	0,710
239112_at	**LOC440504**	NA	0,0593718	0,880	0,0479821	0,802	0,841
206006_s_at	**C6orf84**	chromosome 6 open reading frame 84	0,0594662	0,691	0,0465143	0,654	0,673
205426_s_at	**HIP1**	huntingtin interacting protein 1	0,0594831	0,714	0,0119039	0,572	0,643
227639_at	**PIGK**	phosphatidylinositol glycan, class K	0,0595021	0,827	0,0324867	0,775	0,801
204808_s_at	**TMEM5**	transmembrane protein 5	0,0597197	0,833	0,0481216	0,692	0,762
1552276_a_at	**VPS18**	vacuolar protein sorting protein 18	0,0597197	0,820	0,0516913	0,830	0,825
1554272_at	**LRAP**	NA	0,0597260	0,951	0,0437132	0,904	0,927
55616_at	**PERLD1**	per1-like domain containing 1	0,0597473	0,845	0,0045274	0,786	0,816
214608_s_at	**EYA1**	eyes absent homolog 1 (Drosophila)	0,0597708	0,424	0,0525977	0,331	0,377
215668_s_at	**PLXNB1**	plexin B1	0,0598242	0,871	0,0051238	0,588	0,729
235253_at	**RAD1**	RAD1 homolog (S. pombe)	0,0598852	0,756	0,0064520	0,733	0,744
228832_at	**FLJ20021**	NA	0,0598907	0,777	0,0228044	0,814	0,795
227227_at	**LRRC37A**	leucine rich repeat containing 37A	0,0600398	0,661	0,0055136	0,674	0,668

217687_at	**ADCY2**	adenylate cyclase 2 (brain)	0,0600818	0,732	0,0455593	0,446	0,589
236935_at	**PTPN4**	protein tyrosine phosphatase, non-receptor type 4 (megakaryocyte)	0,0600849	0,812	0,0092997	0,770	0,791
203437_at	**TMEM11**	transmembrane protein 11	0,0601496	0,839	0,0150758	0,833	0,836
205594_at	**ZNF652**	zinc finger protein 652	0,0601620	0,496	0,0026617	0,567	0,531
209077_at	**TXN2**	thioredoxin 2	0,0602800	0,882	0,0298720	0,859	0,870
219009_at	**C14orf93**	chromosome 14 open reading frame 93	0,0603567	0,729	0,0322230	0,730	0,729
235846_at	**RAD54B**	RAD54 homolog B (S. cerevisiae)	0,0603573	0,745	0,0176992	0,719	0,732
217254_s_at	**EPO**	erythropoietin	0,0605394	0,880	0,0343309	0,872	0,876
222648_at	**TBC1D16**	TBC1 domain family, member 16	0,0607012	0,801	0,0362847	0,734	0,768
209183_s_at	**C10orf10**	chromosome 10 open reading frame 10	0,0607599	0,593	0,0416947	0,469	0,531
208503_s_at	**GATAD1**	GATA zinc finger domain containing 1	0,0610707	0,803	0,0267816	0,706	0,755
223323_x_at	**TRPM7**	transient receptor potential cation channel, subfamily M, member 7	0,0611967	0,804	0,0119325	0,693	0,748
219114_at	**C3orf18**	chromosome 3 open reading frame 18	0,0615385	0,779	0,0051602	0,761	0,770
204581_at	**CD22**	CD22 antigen	0,0617122	0,856	0,0427489	0,754	0,805
202440_s_at	**ST5**	suppression of tumorigenicity 5	0,0617122	0,619	0,0356572	0,550	0,585
224318_s_at	**FLJ10081**	NA	0,0617189	0,820	0,0199393	0,784	0,802

ProbesetID	Gene Symbol	Gene Description	Adjusted p-value p	FCp
201327_s_at	**CCT6A**	chaperonin containing TCP1, subunit 6A (zeta 1)	0,0000000	1,358
218549_s_at	**FAM82B**	family with sequence similarity 82, member B	0,0000000	1,436
218513_at	**FLJ11184**	NA	0,0000000	1,849
218239_s_at	**GTPBP4**	GTP binding protein 4	0,0000000	1,725
211931_s_at	**HNRPA3**	heterogeneous nuclear ribonucleoprotein A3	0,0000000	1,830
212633_at	**KIAA0776**	KIAA0776	0,0000000	1,500
220239_at	**KLHL7**	kelch-like 7 (Drosophila)	0,0000000	1,742
218163_at	**MCTS1**	malignant T cell amplified sequence 1	0,0000000	1,467
205129_at	**NPM3**	nucleophosmin/nucleoplasmin, 3	0,0000000	1,926
200686_s_at	**SFRS11**	splicing factor, arginine/serine-rich 11	0,0000000	1,327
213654_at	**TAF5L**	TAF5-like RNA polymerase II, p300/CBP-associated factor (PCAF)-associated factor, 65kDa	0,0000000	1,436
200777_s_at	**BZW1**	basic leucine zipper and W2 domains 1	0,0000517	1,318
218334_at	**NIF3L1BP1**	NA	0,0000517	1,390
205084_at	**BCAP29**	B-cell receptor-associated protein 29	0,0002160	1,513
218683_at	**PTBP2**	polypyrimidine tract binding protein 2	0,0002160	1,882
218535_s_at	**RIOK2**	RIO kinase 2 (yeast)	0,0002160	1,444
217834_s_at	**SYNCRIP**	synaptotagmin binding, cytoplasmic RNA interacting protein	0,0002160	1,666
201948_at	**GNL2**	guanine nucleotide binding protein-like 2 (nucleolar)	0,0002614	1,788
221896_s_at	**HIGD1A**	HIG1 domain family, member 1A	0,0002614	1,319
218102_at	**DERA**	2-deoxyribose-5-phosphate aldolase homolog (C. elegans)	0,0002732	1,476
201345_s_at	**UBE2D2**	ubiquitin-conjugating enzyme E2D 2 (UBC4/5 homolog, yeast)	0,0002732	1,371
224725_at	**MIB1**	mindbomb homolog 1 (Drosophila)	0,0002773	1,333
215136_s_at	**EXOSC8**	exosome component 8	0,0003015	1,836
202763_at	**CASP3**	caspase 3, apoptosis-related cysteine peptidase	0,0003110	1,438
224737_x_at	**CCAR1**	cell division cycle and apoptosis regulator 1	0,0003110	1,425
203310_at	**STXBP3**	syntaxin binding protein 3	0,0003661	1,352
210970_s_at	**IBTK**	inhibitor of Bruton agammaglobulinemia tyrosine kinase	0,0004756	1,485
225760_at	**MYSM1**	myb-like, SWIRM and MPN domains 1	0,0004756	1,466
218668_s_at	**RAP2C**	RAP2C, member of RAS oncogene family	0,0004756	1,396
226290_at	**BDP1**	B double prime 1, subunit of RNA polymerase III transcription initiation factor IIIB	0,0004948	1,404
234997_x_at	**LOC388760**	NA	0,0005316	1,499
224714_at	**MKI67IP**	MKI67 (FHA domain) interacting nucleolar phosphoprotein	0,0005337	1,393
224309_s_at	**SUGT1**	SGT1, suppressor of G2 allele of SKP1 (S. cerevisiae)	0,0005337	1,467
218134_s_at	**RBM22**	RNA binding motif protein 22	0,0005504	1,345
223180_s_at	**C18orf55**	chromosome 18 open reading frame 55	0,0005653	1,529
202505_at	**SNRPB2**	small nuclear ribonucleoprotein polypeptide B''	0,0005653	1,267
201682_at	**PMPCB**	peptidase (mitochondrial processing) beta	0,0005784	1,334
209817_at	**PPP3CB**	protein phosphatase 3 (formerly 2B), catalytic subunit, beta isoform (calcineurin A beta)	0,0006153	1,360
202840_at	**TAF15**	TAF15 RNA polymerase II, TATA box binding protein (TBP)-associated factor, 68kDa	0,0006153	1,585
1555325_s_at	**ZNF26**	zinc finger protein 26 (KOX 20)	0,0006153	1,310
212426_s_at	**YWHAQ**	tyrosine 3-monooxygenase/tryptophan 5-monooxygenase activation protein, theta polypeptide	0,0006358	1,234
223480_s_at	**MRPL47**	mitochondrial ribosomal protein L47	0,0006372	1,285
201091_s_at	**CBX3**	chromobox homolog 3 (HP1 gamma homolog, Drosophila)	0,0006629	1,628
212410_at	**EFHA1**	EF-hand domain family, member A1	0,0006629	1,147
204796_at	**EML1**	echinoderm microtubule associated protein like 1	0,0006629	1,563
218147_s_at	**GLT8D1**	glycosyltransferase 8 domain containing 1	0,0006629	1,401
202232_s_at	**hfl-B5**	NA	0,0006629	1,307
200726_at	**PPP1CC**	protein phosphatase 1, catalytic subunit, gamma isoform	0,0007092	1,306
217851_s_at	**C20orf45**	chromosome 20 open reading frame 45	0,0007794	1,499
232075_at	**WDR61**	WD repeat domain 61	0,0007794	1,288

242808_at	**XRRA1**	X-ray radiation resistance associated 1	0,0008168	1,234
212824_at	**FUBP3**	far upstream element (FUSE) binding protein 3	0,0008312	1,278
207624_s_at	**RPGR**	retinitis pigmentosa GTPase regulator	0,0010730	1,364
235391_at	**FAM92A1**	family with sequence similarity 92, member A1	0,0011419	1,238
223133_at	**TMEM14B**	transmembrane protein 14B	0,0011580	1,271
213128_s_at	**UBE3A**	ubiquitin protein ligase E3A (human papilloma virus E6-associated protein, Angelman syndrome)	0,0011580	1,269
205667_at	**WRN**	Werner syndrome	0,0011580	1,365
212672_at	**ATM**	ataxia telangiectasia mutated (includes complementation groups A, C and D)	0,0012096	1,430
200705_s_at	**EEF1B2**	eukaryotic translation elongation factor 1 beta 2	0,0012460	1,183
209096_at	**UBE2V2**	ubiquitin-conjugating enzyme E2 variant 2	0,0012460	1,262
213165_at	**CAP350**	NA	0,0012547	1,324
58780_s_at	**FLJ10357**	NA	0,0012597	1,515
32088_at	**BLZF1**	basic leucine zipper nuclear factor 1 (JEM-1)	0,0013125	1,459
213103_at	**STARD13**	START domain containing 13	0,0013290	1,683
220643_s_at	**FAIM**	Fas apoptotic inhibitory molecule	0,0014108	1,411
202823_at	**TCEB1**	transcription elongation factor B (SIII), polypeptide 1 (15kDa, elongin C)	0,0014382	1,344
217336_at	**LOC158104**	NA	0,0014697	1,368
216305_s_at	**C2orf3**	chromosome 2 open reading frame 3	0,0014728	1,402
206583_at	**ZNF673**	zinc finger protein 673	0,0014814	1,379
213372_at	**PAQR3**	progestin and adipoQ receptor family member III	0,0014945	1,366
201407_s_at	**PPP1CB**	protein phosphatase 1, catalytic subunit, beta isoform	0,0014945	1,528
201519_at	**TOMM70A**	translocase of outer mitochondrial membrane 70 homolog A (yeast)	0,0015248	1,193
219178_at	**QTRTD1**	queuine tRNA-ribosyltransferase domain containing 1	0,0015835	1,392
226395_at	**LOC286170**	NA	0,0016655	1,342
225892_at	**IREB2**	iron-responsive element binding protein 2	0,0019074	1,373
217508_s_at	**C18orf25**	chromosome 18 open reading frame 25	0,0019684	1,361
208975_s_at	**KPNB1**	karyopherin (importin) beta 1	0,0019970	1,493
218568_at	**MULK**	multiple substrate lipid kinase	0,0020083	1,213
202666_s_at	**ACTL6A**	actin-like 6A	0,0021024	1,533
223397_s_at	**CGI-37**	NA	0,0021104	1,423
222500_at	**PPIL1**	peptidylprolyl isomerase (cyclophilin)-like 1	0,0021104	1,502
213151_s_at	**SEPT7p**	septin 7	0,0021656	1,206
212493_s_at	**HYPB**	NA	0,0021912	1,279
211698_at	**CRI1**	CREBBP/EP300 inhibitor 1	0,0021916	1,291
218670_at	**PUS1**	pseudouridylate synthase 1	0,0021916	1,263
202933_s_at	**YES1**	v-yes-1 Yamaguchi sarcoma viral oncogene homolog 1	0,0021977	1,441
1553749_at	**FAM76B**	family with sequence similarity 76, member B	0,0022019	1,511
221970_s_at	**NOL11**	nucleolar protein 11	0,0022032	1,295
235114_x_at	**HOOK3**	hook homolog 3 (Drosophila)	0,0022872	1,381
220768_s_at	**CSNK1G3**	casein kinase 1, gamma 3	0,0023040	1,201
228054_at	**TMEM44**	transmembrane protein 44	0,0023852	1,365
201725_at	**C10orf7**	chromosome 10 open reading frame 7	0,0024132	1,208
206022_at	**NDP**	Norrie disease (pseudoglioma)	0,0024744	3,568
218003_s_at	**FKBP3**	FK506 binding protein 3, 25kDa	0,0025106	1,352
219163_at	**ZNF562**	zinc finger protein 562	0,0025106	1,233
205976_at	**KIAA0971**	KIAA0971	0,0025196	1,218
1568249_at	**RNU71B**	RNA, U71B small nucleolar	0,0025466	1,474
239406_at	**ZNF193**	zinc finger protein 193	0,0025832	1,348
205251_at	**PER2**	period homolog 2 (Drosophila)	0,0026414	1,885
244197_x_at	**CNOT2**	CCR4-NOT transcription complex, subunit 2	0,0026735	1,496
1563532_at	**HMCN2**	hemicentin 2	0,0026867	1,110
201472_at	**VBP1**	von Hippel-Lindau binding protein 1	0,0026882	1,196

224428_s_at	**CDCA7**	cell division cycle associated 7	0,0027369	1,895
201872_s_at	**ABCE1**	ATP-binding cassette, sub-family E (OABP), member 1	0,0027915	1,333
210097_s_at	**NOL7**	nucleolar protein 7, 27kDa	0,0029435	1,321
243643_x_at	**SLC30A6**	solute carrier family 30 (zinc transporter), member 6	0,0030062	1,248
219531_at	**Cep72**	NA	0,0030439	1,357
208838_at	**CAND1**	cullin-associated and neddylation-dissociated 1	0,0031271	1,200
213918_s_at	**NIPBL**	Nipped-B homolog (Drosophila)	0,0031271	1,245
232613_at	**PB1**	NA	0,0031271	1,334
200994_at	**IPO7**	importin 7	0,0031340	1,235
1555573_at	**C10orf93**	chromosome 10 open reading frame 93	0,0031478	1,278
209666_s_at	**CHUK**	conserved helix-loop-helix ubiquitous kinase	0,0031900	1,249
209524_at	**HDGFRP3**	NA	0,0032046	1,362
201795_at	**LBR**	lamin B receptor	0,0032571	1,889
242445_at	**FGD4**	FYVE, RhoGEF and PH domain containing 4	0,0032905	1,304
222745_s_at	**C15orf29**	chromosome 15 open reading frame 29	0,0033274	1,419
200040_at	**KHDRBS1**	KH domain containing, RNA binding, signal transduction associated 1	0,0033274	1,206
1560069_at	**LOC389072**	NA	0,0033400	1,286
241865_at	**GNB1**	guanine nucleotide binding protein (G protein), beta polypeptide 1	0,0034238	1,381
224691_at	**UHMK1**	U2AF homology motif (UHM) kinase 1	0,0034306	1,327
1555864_s_at	**PDHA1**	pyruvate dehydrogenase (lipoamide) alpha 1	0,0034669	1,340
203429_s_at	**C1orf9**	chromosome 1 open reading frame 9	0,0034872	1,342
215596_s_at	**ZNF294**	zinc finger protein 294	0,0035274	1,297
229233_at	**NRG3**	neuregulin 3	0,0036760	1,131
208816_x_at	**ANXA2P2**	annexin A2 pseudogene 2	0,0036833	1,124
231211_s_at	**LOC541469**	NA	0,0037274	1,454
218605_at	**TFB2M**	transcription factor B2, mitochondrial	0,0037765	1,317
224511_s_at	**TXNL5**	thioredoxin-like 5	0,0038049	1,450
236915_at	**LOC441054**	NA	0,0038319	1,937
1568951_at	**LOC387870**	NA	0,0039168	1,495
214140_at	**SLC25A16**	c("solute carrier family 25 (mitochondrial carrier", " Graves disease autoantigen), member 16")	0,0039236	1,393
218096_at	**AGPAT5**	1-acylglycerol-3-phosphate O-acyltransferase 5 (lysophosphatidic acid acyltransferase, epsilon)	0,0039466	1,533
207346_at	**EPIM**	epimorphin	0,0039473	1,232
208511_at	**PTTG3**	pituitary tumor-transforming 3	0,0040058	1,786
239246_at	**FARP1**	FERM, RhoGEF (ARHGEF) and pleckstrin domain protein 1 (chondrocyte-derived)	0,0040217	1,424
218085_at	**CHMP5**	chromatin modifying protein 5	0,0040604	1,196
216348_at	**LOC402057**	NA	0,0040903	1,236
208845_at	**VDAC3**	voltage-dependent anion channel 3	0,0041732	1,301
200056_s_at	**C1D**	NA	0,0042803	1,223
1570561_at	**NA**	NA	0,0042803	5,720
225916_at	**ZNF131**	zinc finger protein 131 (clone pHZ-10)	0,0042803	1,337
204145_at	**FRG1**	FSHD region gene 1	0,0043096	1,348
239971_at	**C16orf45**	chromosome 16 open reading frame 45	0,0043686	1,222
203395_s_at	**HES1**	hairy and enhancer of split 1, (Drosophila)	0,0044184	2,237
242390_at	**WDFY1**	WD repeat and FYVE domain containing 1	0,0044184	1,317
201227_s_at	**NDUFB8**	NADH dehydrogenase (ubiquinone) 1 beta subcomplex, 8, 19kDa	0,0044288	1,286
226385_s_at	**C7orf30**	chromosome 7 open reading frame 30	0,0044292	1,174
220171_x_at	**KIAA1704**	KIAA1704	0,0044292	1,222
208319_s_at	**RBM3**	RNA binding motif (RNP1, RRM) protein 3	0,0044505	1,268
218508_at	**DCP1A**	decapping enzyme	0,0044944	1,220
235007_at	**BBS7**	Bardet-Biedl syndrome 7	0,0045215	1,362
216177_at	**LOC283412**	NA	0,0045215	1,285
211010_s_at	**NCR3**	natural cytotoxicity triggering receptor 3	0,0045215	1,159

219258_at	**FLJ20516**	NA	0,0045274	1,440
231839_at	**PDE12**	2'-PDE	0,0045278	1,180
224906_at	**TMEM16F**	transmembrane protein 16F	0,0045393	1,331
212060_at	**SR140**	NA	0,0045895	1,327
44111_at	**VPS33B**	vacuolar protein sorting 33B (yeast)	0,0046804	1,253
202484_s_at	**MBD2**	methyl-CpG binding domain protein 2	0,0047166	1,297
232521_at	**PCSK7**	proprotein convertase subtilisin/kexin type 7	0,0047295	1,278
235138_at	**PUM2**	pumilio homolog 2 (Drosophila)	0,0047475	1,746
231871_at	**ITR**	NA	0,0047651	1,519
211921_x_at	**PTMA**	prothymosin, alpha (gene sequence 28)	0,0047797	1,545
208549_x_at	**LOC440085**	NA	0,0047991	1,498
1554095_at	**PRR8**	proline rich 8	0,0048596	1,440
242041_at	**CSPP1**	centrosome and spindle pole associated protein 1	0,0049162	1,252
201833_at	**HDAC2**	histone deacetylase 2	0,0049171	1,167
203086_at	**KIF2**	kinesin heavy chain member 2	0,0049483	1,294
204901_at	**BTRC**	beta-transducin repeat containing	0,0049879	1,205
205603_s_at	**DIAPH2**	diaphanous homolog 2 (Drosophila)	0,0050892	1,481
219819_s_at	**MRPS28**	mitochondrial ribosomal protein S28	0,0052829	1,234
223151_at	**MGC2714**	NA	0,0052939	1,236
1556641_at	**FLJ37228**	NA	0,0053203	1,348
232251_at	**LOC152195**	NA	0,0053420	1,120
1569022_a_at	**PIK3C2A**	phosphoinositide-3-kinase, class 2, alpha polypeptide	0,0054445	1,469
225676_s_at	**WDSOF1**	WD repeats and SOF1 domain containing	0,0054697	1,347
201297_s_at	**MOBK1B**	MOB1, Mps One Binder kinase activator-like 1B (yeast)	0,0054823	1,333
229751_s_at	**DKFZP434G1415**	NA	0,0054907	1,479
219002_at	**FLJ21901**	NA	0,0055320	1,333
205525_at	**CALD1**	caldesmon 1	0,0055605	2,941
205788_s_at	**ZC3H11A**	zinc finger CCCH-type containing 11A	0,0056055	1,164
227344_at	**ZNFN1A1**	zinc finger protein, subfamily 1A, 1 (Ikaros)	0,0056214	1,171
204569_at	**ICK**	intestinal cell (MAK-like) kinase	0,0056316	1,379
209654_at	**KIAA0947**	NA	0,0056495	1,158
238634_x_at	**C10orf59**	chromosome 10 open reading frame 59	0,0056864	1,196
1555947_at	**C9orf10**	chromosome 9 open reading frame 10	0,0057143	1,364
225223_at	**SMAD5**	SMAD, mothers against DPP homolog 5 (Drosophila)	0,0057261	1,259
209547_s_at	**SF4**	splicing factor 4	0,0057606	1,243
205613_at	**SYT17**	synaptotagmin XVII	0,0057790	1,203
215203_at	**GOLGA4**	golgi autoantigen, golgin subfamily a, 4	0,0058256	1,440
213365_at	**MGC16943**	NA	0,0058311	1,430
204023_at	**RFC4**	replication factor C (activator 1) 4, 37kDa	0,0058406	1,675
212783_at	**RBBP6**	retinoblastoma binding protein 6	0,0058575	1,253
202673_at	**DPM1**	dolichyl-phosphate mannosyltransferase polypeptide 1, catalytic subunit	0,0058912	1,155
1556806_at	**LOC442133**	NA	0,0059423	1,197
203301_s_at	**DMTF1**	cyclin D binding myb-like transcription factor 1	0,0060062	1,285
233197_at	**LOC158014**	NA	0,0060062	1,280
209662_at	**CETN3**	centrin, EF-hand protein, 3 (CDC31 homolog, yeast)	0,0061114	1,299
1554732_at	**MGC24125**	NA	0,0061258	1,173
220576_at	**PGAP1**	NA	0,0061258	1,238
219240_s_at	**C10orf88**	chromosome 10 open reading frame 88	0,0062183	1,334
229294_at	**JPH3**	junctophilin 3	0,0062291	1,276
1565935_at	**LOC91431**	NA	0,0063430	1,220
213959_s_at	**KIAA1005**	NA	0,0063614	1,521
217965_s_at	**HCNGP**	NA	0,0063934	1,172
225519_at	**PPP4R2**	protein phosphatase 4, regulatory subunit 2	0,0064520	1,260

214772_at	**G2**	NA	0,0064812	1,681
225439_at	**NUDCD1**	NudC domain containing 1	0,0065321	1,380
1556721_at	**FLJ33706**	NA	0,0065496	1,252
227872_at	**POLR3A**	polymerase (RNA) III (DNA directed) polypeptide A, 155kDa	0,0065496	1,250
219885_at	**FLJ10260**	NA	0,0066448	1,584
238614_x_at	**ZNF430**	zinc finger protein 430	0,0066656	1,397
231714_s_at	**AP4B1**	adaptor-related protein complex 4, beta 1 subunit	0,0066741	1,271
224985_at	**CSDE1**	cold shock domain containing E1, RNA-binding	0,0066769	1,305
218256_s_at	**NUP54**	nucleoporin 54kDa	0,0067580	1,249
212168_at	**RBM12**	RNA binding motif protein 12	0,0067670	1,274
1562733_at	**LOC401544**	NA	0,0068030	1,155
244633_at	**PIAS2**	protein inhibitor of activated STAT, 2	0,0068974	1,284
228286_at	**FLJ40869**	NA	0,0069548	1,497
203344_s_at	**RBBP8**	retinoblastoma binding protein 8	0,0069678	1,298
224022_x_at	**WNT16**	wingless-type MMTV integration site family, member 16	0,0071158	1,163
222476_at	**CNOT6**	CCR4-NOT transcription complex, subunit 6	0,0071290	1,308
232336_at	**ZSWIM5**	zinc finger, SWIM-type containing 5	0,0071488	1,245
226762_at	**PURB**	purine-rich element binding protein B	0,0071608	1,423
203253_s_at	**KIAA0433**	NA	0,0072245	1,183
213020_at	**GOSR1**	golgi SNAP receptor complex member 1	0,0072349	1,199
225128_at	**KDELC2**	KDEL (Lys-Asp-Glu-Leu) containing 2	0,0073381	1,358
219200_at	**MGC5297**	NA	0,0073381	1,203
201303_at	**DDX48**	DEAD (Asp-Glu-Ala-Asp) box polypeptide 48	0,0074167	1,181
222930_s_at	**AGMAT**	agmatine ureohydrolase (agmatinase)	0,0074775	1,304
225581_s_at	**MRPL50**	mitochondrial ribosomal protein L50	0,0074775	1,280
206708_at	**HTLF**	human T-cell leukemia virus enhancer factor	0,0074908	1,147
226150_at	**PPAPDC1B**	phosphatidic acid phosphatase type 2 domain containing 1B	0,0075238	1,546
1561676_at	**PRICKLE2**	prickle-like 2 (Drosophila)	0,0075543	1,135
204809_at	**CLPX**	ClpX caseinolytic peptidase X homolog (E. coli)	0,0076011	1,261
225765_at	**TNPO1**	transportin 1	0,0076476	1,329
1559607_s_at	**GBP6**	guanylate binding protein family, member 6	0,0076661	1,161
202144_s_at	**ADSL**	adenylosuccinate lyase	0,0077156	1,233
206632_s_at	**APOBEC3B**	apolipoprotein B mRNA editing enzyme, catalytic polypeptide-like 3B	0,0077683	4,750
201466_s_at	**JUN**	v-jun sarcoma virus 17 oncogene homolog (avian)	0,0077859	1,473
200606_at	**DSP**	desmoplakin	0,0078105	1,729
205401_at	**AGPS**	alkylglycerone phosphate synthase	0,0078445	1,404
223416_at	**SF3B14**	NA	0,0078525	1,166
46167_at	**C1orf175**	chromosome 1 open reading frame 175	0,0078799	1,275
235025_at	**C14orf150**	chromosome 14 open reading frame 150	0,0079218	1,363
219362_at	**MAK10**	MAK10 homolog, amino-acid N-acetyltransferase subunit, (S. cerevisiae)	0,0080323	1,211
228442_at	**NFATC2**	nuclear factor of activated T-cells, cytoplasmic, calcineurin-dependent 2	0,0080323	2,383
228378_at	**DKFZp434N2030**	NA	0,0080823	1,311
1555001_at	**IQCF1**	IQ motif containing F1	0,0081236	1,148
243299_at	**VRK2**	vaccinia related kinase 2	0,0081236	1,419
219243_at	**GIMAP4**	GTPase, IMAP family member 4	0,0081252	1,145
1560485_at	**HIVEP1**	human immunodeficiency virus type I enhancer binding protein 1	0,0081546	1,276
227261_at	**KLF12**	Kruppel-like factor 12	0,0082240	1,458
243099_at	**NFAM1**	NFAT activating protein with ITAM motif 1	0,0082367	1,274
215366_at	**SNX13**	sorting nexin 13	0,0082367	1,177
205406_s_at	**SPA17**	sperm autoantigenic protein 17	0,0082989	1,235
209191_at	**TUBB6**	tubulin, beta 6	0,0082989	1,546
234547_at	**EYA3**	eyes absent homolog 3 (Drosophila)	0,0083297	1,128

214696_at	**MGC14376**	NA	0,0083297	1,487
206887_at	**CCBP2**	chemokine binding protein 2	0,0083344	1,148
229351_at	**C6orf49**	chromosome 6 open reading frame 49	0,0084122	1,196
1561691_at	**LOC285735**	NA	0,0085136	1,145
217266_at	**LOC136321**	NA	0,0085316	1,223
233722_at	**ZNF638**	zinc finger protein 638	0,0086199	1,130
242047_at	**PLEKHK1**	pleckstrin homology domain containing, family K member 1	0,0086399	1,181
218577_at	**LRRC40**	leucine rich repeat containing 40	0,0086437	1,211
202042_at	**HARS**	histidyl-tRNA synthetase	0,0086667	1,233
220017_x_at	**CYP2C9**	cytochrome P450, family 2, subfamily C, polypeptide 9	0,0089249	1,284
1569241_a_at	**LOC114977**	NA	0,0089490	1,243
225470_at	**NUP35**	nucleoporin 35kDa	0,0089547	1,371
218823_s_at	**KCTD9**	potassium channel tetramerisation domain containing 9	0,0089746	1,332
222768_s_at	**CGI-09**	NA	0,0089870	1,240
54632_at	**THADA**	thyroid adenoma associated	0,0089870	1,220
201437_s_at	**EIF4E**	eukaryotic translation initiation factor 4E	0,0090858	1,517
226684_at	**C14orf103**	chromosome 14 open reading frame 103	0,0090992	1,193
223236_at	**DKFZP434K1421**	NA	0,0091555	1,181
230532_at	**CXorf38**	chromosome X open reading frame 38	0,0091656	1,381
1555310_a_at	**PAK6**	p21(CDKN1A)-activated kinase 6	0,0091656	1,155
1553984_s_at	**DTYMK**	deoxythymidylate kinase (thymidylate kinase)	0,0092997	1,583
53071_s_at	**FLJ22222**	NA	0,0093906	1,308
201501_s_at	**GRSF1**	G-rich RNA sequence binding factor 1	0,0094352	1,216
204228_at	**PPIH**	peptidyl prolyl isomerase H (cyclophilin H)	0,0094572	1,250
201342_at	**SNRPC**	small nuclear ribonucleoprotein polypeptide C	0,0094815	1,175
207229_at	**KLRA1**	killer cell lectin-like receptor subfamily A, member 1	0,0095315	1,141
228853_at	**STYX**	serine/threonine/tyrosine interacting protein	0,0095315	1,463
212857_x_at	**LOC342346**	NA	0,0096312	1,121
225367_at	**PGM2**	phosphoglucomutase 2	0,0096991	1,638
201558_at	**RAE1**	RAE1 RNA export 1 homolog (S. pombe)	0,0097077	1,343
227788_at	**USP13**	ubiquitin specific peptidase 13 (isopeptidase T-3)	0,0098469	1,226
200944_s_at	**HMGN1**	high-mobility group nucleosome binding domain 1	0,0098582	1,208
225748_at	**C6orf93**	chromosome 6 open reading frame 93	0,0098725	1,197
222479_s_at	**DNCLI1**	dynein, cytoplasmic, light intermediate polypeptide 1	0,0099027	1,203
222258_s_at	**SH3BP4**	SH3-domain binding protein 4	0,0099628	1,252
208382_s_at	**DMC1**	DMC1 dosage suppressor of mck1 homolog, meiosis-specific homologous recombination (yeast)	0,0099749	1,160
222309_at	**C6orf62**	chromosome 6 open reading frame 62	0,0100146	1,605
204172_at	**CPOX**	coproporphyrinogen oxidase	0,0100348	1,279
203712_at	**KIAA0020**	KIAA0020	0,0100631	1,292
213346_at	**LOC93081**	NA	0,0101975	1,499
238913_at	**CPSF6**	cleavage and polyadenylation specific factor 6, 68kDa	0,0103075	1,263
223413_s_at	**LYAR**	NA	0,0103094	1,319
1569666_s_at	**SLC37A3**	solute carrier family 37 (glycerol-3-phosphate transporter), member 3	0,0104844	1,175
1557615_a_at	**SLIT1**	slit homolog 1 (Drosophila)	0,0105146	1,137
244268_x_at	**LOC441708**	NA	0,0106013	1,162
1565358_at	**RARA**	retinoic acid receptor, alpha	0,0106169	3,024
208878_s_at	**PAK2**	p21 (CDKN1A)-activated kinase 2	0,0106282	1,156
216787_at	**FKBP1B**	FK506 binding protein 1B, 12.6 kDa	0,0106466	1,162
215310_at	**APC**	adenomatosis polyposis coli	0,0106705	1,194
203011_at	**IMPA1**	inositol(myo)-1(or 4)-monophosphatase 1	0,0106705	1,224
232744_x_at	**LOC360030**	NA	0,0106804	1,314
208694_at	**PRKDC**	protein kinase, DNA-activated, catalytic polypeptide	0,0107350	1,287
218170_at	**ISOC1**	isochorismatase domain containing 1	0,0107935	1,265

210406_s_at	**RAB6C**	RAB6C, member RAS oncogene family	0,0108449	1,146
1562063_x_at	**LOC200030**	NA	0,0109688	1,316
223850_at	**DNAJA2**	DnaJ (Hsp40) homolog, subfamily A, member 2	0,0110385	1,203
222825_at	**CGI-77**	NA	0,0110674	1,380
220940_at	**KIAA1641**	KIAA1641	0,0110824	1,596
233681_at	**KRTAP3-3**	keratin associated protein 3-3	0,0111232	1,099
221616_s_at	**TAF9L**	TAF9-like RNA polymerase II, TATA box binding protein (TBP)-associated factor, 31kDa	0,0111762	1,188
227282_at	**PCDH19**	protocadherin 19	0,0111801	1,983
240045_at	**PRDM16**	PR domain containing 16	0,0113755	1,361
238563_at	**ABI1**	abl-interactor 1	0,0114422	1,383
1554047_at	**TXNDC9**	thioredoxin domain containing 9	0,0114590	1,230
227680_at	**ZNF326**	zinc finger protein 326	0,0114611	1,327
225083_at	**C6orf51**	chromosome 6 open reading frame 51	0,0114695	1,219
1564654_at	**COL4A6**	collagen, type IV, alpha 6	0,0115551	1,147
222064_s_at	**MGC2744**	NA	0,0115551	1,197
209045_at	**XPNPEP1**	X-prolyl aminopeptidase (aminopeptidase P) 1, soluble	0,0116223	1,170
225231_at	**CBL**	Cas-Br-M (murine) ecotropic retroviral transforming sequence	0,0116689	1,304
224523_s_at	**MGC4308**	NA	0,0116689	1,359
208154_at	**LOC51336**	NA	0,0116739	1,143
200017_at	**RPS27A**	ribosomal protein S27a	0,0117068	1,084
204391_x_at	**TRIM24**	tripartite motif-containing 24	0,0117892	1,242
215700_x_at	**CPNE6**	copine VI (neuronal)	0,0118503	1,131
228594_at	**FLJ30596**	NA	0,0118503	1,342
216508_x_at	**HMG1L1**	high-mobility group (nonhistone chromosomal) protein 1-like 1	0,0118528	1,602
201699_at	**PSMC6**	proteasome (prosome, macropain) 26S subunit, ATPase, 6	0,0118528	1,297
51176_at	**CRSP8**	cofactor required for Sp1 transcriptional activation, subunit 8, 34kDa	0,0119503	1,195
221514_at	**UTP14A**	UTP14, U3 small nucleolar ribonucleoprotein, homolog A (yeast)	0,0120569	1,233
221570_s_at	**METTL5**	methyltransferase like 5	0,0120578	1,197
1554333_at	**DNAJA4**	DnaJ (Hsp40) homolog, subfamily A, member 4	0,0121342	1,172
218827_s_at	**Cep192**	NA	0,0121372	1,329
200099_s_at	**RPS3A**	ribosomal protein S3A	0,0121372	1,068
1564164_at	**FLJ20054**	NA	0,0121695	1,282
212381_at	**USP24**	ubiquitin specific peptidase 24	0,0123573	1,346
226922_at	**RANBP2**	RAN binding protein 2	0,0123930	1,234
221770_at	**SLC4A10**	solute carrier family 4, sodium bicarbonate transporter-like, member 10	0,0124002	1,228
205255_x_at	**TCF7**	transcription factor 7 (T-cell specific, HMG-box)	0,0125531	1,450
228577_x_at	**ODF2L**	outer dense fiber of sperm tails 2-like	0,0125539	1,425
219212_at	**HSPA14**	heat shock 70kDa protein 14	0,0125968	1,276
209219_at	**RDBP**	RD RNA binding protein	0,0126063	1,167
203714_s_at	**TBCE**	tubulin-specific chaperone e	0,0126063	1,194
230609_at	**ENTH**	NA	0,0126217	1,760
226868_at	**LOC283464**	NA	0,0126580	1,664
204634_at	**NEK4**	NIMA (never in mitosis gene a)-related kinase 4	0,0126580	1,331
216652_s_at	**DR1**	down-regulator of transcription 1, TBP-binding (negative cofactor 2)	0,0127530	1,394
243982_at	**BTBD5**	BTB (POZ) domain containing 5	0,0128010	1,402
217746_s_at	**PDCD6IP**	programmed cell death 6 interacting protein	0,0128936	1,189
235773_at	**ZIK1**	NA	0,0128936	1,206
226465_s_at	**SON**	SON DNA binding protein	0,0129056	1,306
220526_s_at	**MRPL20**	mitochondrial ribosomal protein L20	0,0129761	1,160
204342_at	**SLC25A24**	c("solute carrier family 25 (mitochondrial carrier", " phosphate carrier), member 24")	0,0129761	1,148
1555762_s_at	**MKL1**	megakaryoblastic leukemia (translocation) 1	0,0129867	1,250
227693_at	**WDR20**	WD repeat domain 20	0,0130023	1,256

231003_at	**SLC35B3**	solute carrier family 35, member B3	0,0130717	1,397
200870_at	**STRAP**	serine/threonine kinase receptor associated protein	0,0130717	1,172
207185_at	**SLC10A1**	solute carrier family 10 (sodium/bile acid cotransporter family), member 1	0,0130804	1,133
209803_s_at	**PHLDA2**	pleckstrin homology-like domain, family A, member 2	0,0132252	1,692
222464_s_at	**C10orf119**	chromosome 10 open reading frame 119	0,0132480	1,234
214799_at	**NFASC**	NA	0,0132529	1,358
209313_at	**XAB1**	XPA binding protein 1, GTPase	0,0133500	1,139
221773_at	**ELK3**	ELK3, ETS-domain protein (SRF accessory protein 2)	0,0133743	1,662
201088_at	**KPNA2**	karyopherin alpha 2 (RAG cohort 1, importin alpha 1)	0,0134247	1,525
203078_at	**CUL2**	cullin 2	0,0134639	1,343
203693_s_at	**E2F3**	E2F transcription factor 3	0,0134898	1,275
223040_at	**NAT5**	N-acetyltransferase 5 (ARD1 homolog, S. cerevisiae)	0,0135059	1,234
214766_s_at	**AHCTF1**	AT hook containing transcription factor 1	0,0135425	1,326
1559623_at	**PTD012**	NA	0,0135435	1,160
214801_at	**TOR1AIP2**	torsin A interacting protein 2	0,0135999	1,275
1564281_at	**LOC285708**	NA	0,0136758	1,404
230144_at	**LOC392533**	NA	0,0137403	1,305
1562473_at	**PEX5L**	peroxisomal biogenesis factor 5-like	0,0137806	1,104
213700_s_at	**PKM2**	pyruvate kinase, muscle	0,0137806	1,305
215249_at	**RPL35A**	ribosomal protein L35a	0,0138477	1,132
227926_s_at	**KIAA1245**	KIAA1245	0,0138993	1,196
1556291_at	**DKFZp564N2472**	NA	0,0139548	1,140
208843_s_at	**GORASP2**	golgi reassembly stacking protein 2, 55kDa	0,0140418	1,258
226011_at	**CCDC12**	coiled-coil domain containing 12	0,0140422	1,173
218826_at	**SLC35F2**	solute carrier family 35, member F2	0,0140550	1,591
213044_at	**ROCK1**	Rho-associated, coiled-coil containing protein kinase 1	0,0140582	1,270
219038_at	**MORC4**	MORC family CW-type zinc finger 4	0,0140986	1,344
219353_at	**NHLRC2**	NHL repeat containing 2	0,0141925	1,250
220428_at	**CD207**	CD207 antigen, langerin	0,0141999	1,186
244640_at	**LOC342892**	NA	0,0143377	1,381
1569601_at	**C2orf34**	chromosome 2 open reading frame 34	0,0143910	1,168
1552518_s_at	**MTBP**	Mdm2, transformed 3T3 cell double minute 2, p53 binding protein (mouse) binding protein, 104kDa	0,0144020	1,096
211241_at	**ANXA2P3**	annexin A2 pseudogene 3	0,0145189	1,211
215797_at	**MGC40069**	NA	0,0145926	1,173
213427_at	**RPP40**	ribonuclease P 40kDa subunit	0,0145937	1,287
211623_s_at	**FBL**	fibrillarin	0,0146039	1,309
215890_at	**GM2A**	GM2 ganglioside activator	0,0146256	1,133
233219_at	**MKLN1**	muskelin 1, intracellular mediator containing kelch motifs	0,0146651	1,266
218658_s_at	**ACTR8**	ARP8 actin-related protein 8 homolog (yeast)	0,0148603	1,309
201657_at	**ARL1**	ADP-ribosylation factor-like 1	0,0148603	1,793
212460_at	**C14orf147**	chromosome 14 open reading frame 147	0,0148603	1,922
204471_at	**GAP43**	growth associated protein 43	0,0148603	4,204
200679_x_at	**HMGB1**	high-mobility group box 1	0,0148603	1,662
220486_x_at	**RP13-360B22.2**	NA	0,0148603	1,334
206034_at	**SERPINB8**	serpin peptidase inhibitor, clade B (ovalbumin), member 8	0,0148603	1,783
208821_at	**SNRPB**	small nuclear ribonucleoprotein polypeptides B and B1	0,0148603	1,562
222532_at	**SRPRB**	signal recognition particle receptor, B subunit	0,0148603	1,587
220865_s_at	**TPRT**	trans-prenyltransferase	0,0148603	2,208
217825_s_at	**UBE2J1**	ubiquitin-conjugating enzyme E2, J1 (UBC6 homolog, yeast)	0,0148603	1,668
201523_x_at	**UBE2N**	ubiquitin-conjugating enzyme E2N (UBC13 homolog, yeast)	0,0148603	1,603
205386_s_at	**MDM2**	Mdm2, transformed 3T3 cell double minute 2, p53 binding protein (mouse)	0,0149049	1,188
216387_x_at	**LOC390411**	NA	0,0149122	1,169

211557_x_at	**SLCO2B1**	solute carrier organic anion transporter family, member 2B1	0,0149161	1,432
228745_at	**FLJ13611**	NA	0,0149226	1,293
241360_at	**LOC387816**	NA	0,0149226	1,462
239404_at	**TOP1**	topoisomerase (DNA) I	0,0149226	1,259
206799_at	**SCGB1D2**	secretoglobin, family 1D, member 2	0,0149559	1,157
207394_at	**ZNF137**	zinc finger protein 137 (clone pHZ-30)	0,0150160	1,116
222402_at	**C13orf12**	chromosome 13 open reading frame 12	0,0150745	1,263
210876_at	**ANXA2P1**	annexin A2 pseudogene 1	0,0152271	1,420
202786_at	**STK39**	serine threonine kinase 39 (STE20/SPS1 homolog, yeast)	0,0152528	1,344
221020_s_at	**MFTC**	NA	0,0153509	1,309
235583_at	**ILDR1**	immunoglobulin-like domain containing receptor 1	0,0153591	1,181
233077_at	**FLJ13439**	NA	0,0153616	1,258
244763_at	**MTRF1**	mitochondrial translational release factor 1	0,0153813	1,148
232555_at	**LOC401317**	NA	0,0154016	1,219
232935_at	**LHFP**	lipoma HMGIC fusion partner	0,0154065	1,760
228415_at	**AP1S2**	adaptor-related protein complex 1, sigma 2 subunit	0,0154426	2,059
215160_x_at	**MGC72104**	NA	0,0154426	1,277
202858_at	**U2AF1**	U2(RNU2) small nuclear RNA auxiliary factor 1	0,0154426	1,749
203847_s_at	**AKAP8**	A kinase (PRKA) anchor protein 8	0,0154509	1,239
206875_s_at	**SLK**	STE20-like kinase (yeast)	0,0154509	1,227
226194_at	**C13orf8**	chromosome 13 open reading frame 8	0,0155366	1,144
219774_at	**FLJ10996**	NA	0,0156318	1,295
218882_s_at	**WDR3**	WD repeat domain 3	0,0156882	1,211
1559820_at	**ATG10**	ATG10 autophagy related 10 homolog (S. cerevisiae)	0,0157634	1,118
228029_at	**KIAA1982**	NA	0,0157634	1,342
215380_s_at	**C7orf24**	chromosome 7 open reading frame 24	0,0157889	1,321
209486_at	**SAS10**	NA	0,0157902	1,417
219678_x_at	**DCLRE1C**	DNA cross-link repair 1C (PSO2 homolog, S. cerevisiae)	0,0157926	1,242
221019_s_at	**COLEC12**	collectin sub-family member 12	0,0158156	2,152
225199_at	**PRO0149**	NA	0,0158156	1,272
226817_at	**DSC2**	desmocollin 2	0,0158375	1,633
212105_s_at	**DHX9**	DEAH (Asp-Glu-Ala-His) box polypeptide 9	0,0158825	2,123
223486_at	**HSPC135**	NA	0,0158825	1,215
227990_at	**SLU7**	NA	0,0159196	1,201
217382_at	**LOC390363**	NA	0,0159414	1,121
227861_at	**MGC33214**	NA	0,0160249	1,225
219230_at	**FLJ10970**	NA	0,0161005	2,368
228019_s_at	**MRPS18C**	mitochondrial ribosomal protein S18C	0,0161187	1,183
227080_at	**ZNF697**	zinc finger protein 697	0,0161187	1,385
219094_at	**ARMC8**	armadillo repeat containing 8	0,0161347	1,348
229012_at	**C9orf24**	chromosome 9 open reading frame 24	0,0161546	1,099
228238_at	**RNU47**	RNA, U47 small nuclear	0,0161812	1,462
218326_s_at	**LGR4**	leucine-rich repeat-containing G protein-coupled receptor 4	0,0162097	2,548
218117_at	**RBX1**	ring-box 1	0,0162553	1,279
206940_s_at	**POU4F1**	POU domain, class 4, transcription factor 1	0,0163406	1,157
235875_at	**SLC1A4**	solute carrier family 1 (glutamate/neutral amino acid transporter), member 4	0,0163915	1,140
230614_at	**CYP19A1**	cytochrome P450, family 19, subfamily A, polypeptide 1	0,0164331	1,171
209778_at	**TRIP11**	thyroid hormone receptor interactor 11	0,0164663	1,208
209570_s_at	**D4S234E**	NA	0,0164939	1,236
206523_at	**PSCD3**	pleckstrin homology, Sec7 and coiled-coil domains 3	0,0166681	1,394
231154_x_at	**TINAG**	tubulointerstitial nephritis antigen	0,0166812	1,198
226980_at	**DEPDC1B**	DEP domain containing 1B	0,0167664	2,206
241379_at	**C2orf13**	chromosome 2 open reading frame 13	0,0167913	1,207

214943_s_at	**RBM34**	RNA binding motif protein 34	0,0167915	1,370
202899_s_at	**SFRS3**	splicing factor, arginine/serine-rich 3	0,0167915	2,147
224608_s_at	**VPS25**	vacuolar protein sorting 25 (yeast)	0,0167915	1,188
233656_s_at	**VPS54**	vacuolar protein sorting 54 (yeast)	0,0167915	1,421
234660_s_at	**KIAA1008**	KIAA1008	0,0167953	1,609
1564399_a_at	**ARMCX4**	armadillo repeat containing, X-linked 4	0,0169534	1,197
228355_s_at	**mimitin**	NA	0,0169534	1,225
1560031_at	**FRMD4A**	FERM domain containing 4A	0,0169580	1,783
1561238_at	**PXMP3**	peroxisomal membrane protein 3, 35kDa (Zellweger syndrome)	0,0170232	1,124
226965_at	**FLJ34969**	NA	0,0170496	1,262
237018_at	**AKAP13**	A kinase (PRKA) anchor protein 13	0,0170596	1,225
200876_s_at	**PSMB1**	proteasome (prosome, macropain) subunit, beta type, 1	0,0171063	1,124
210766_s_at	**CSE1L**	CSE1 chromosome segregation 1-like (yeast)	0,0171707	1,408
235178_x_at	**ESCO2**	establishment of cohesion 1 homolog 2 (S. cerevisiae)	0,0171966	1,270
219459_at	**POLR3B**	polymerase (RNA) III (DNA directed) polypeptide B	0,0171966	1,262
1557053_s_at	**TAX1BP3**	Tax1 (human T-cell leukemia virus type I) binding protein 3	0,0171966	1,212
208682_s_at	**MAGED2**	melanoma antigen family D, 2	0,0173264	1,263
209294_x_at	**TNFRSF10B**	tumor necrosis factor receptor superfamily, member 10b	0,0173264	1,384
1553464_at	**FLJ40288**	NA	0,0173286	1,195
206403_at	**ZNF536**	zinc finger protein 536	0,0173435	1,145
202483_s_at	**RANBP1**	RAN binding protein 1	0,0173494	1,972
219484_at	**HCFC2**	host cell factor C2	0,0173532	1,164
210248_at	**WNT7A**	wingless-type MMTV integration site family, member 7A	0,0174380	1,140
200089_s_at	**RPL4**	ribosomal protein L4	0,0174388	1,111
1554980_a_at	**ATF3**	activating transcription factor 3	0,0174468	1,290
242472_x_at	**FNBP4**	formin binding protein 4	0,0174468	1,142
207035_at	**SLC30A3**	solute carrier family 30 (zinc transporter), member 3	0,0174545	1,244
207334_s_at	**TGFBR2**	transforming growth factor, beta receptor II (70/80kDa)	0,0175002	1,698
235117_at	**ASB3**	ankyrin repeat and SOCS box-containing 3	0,0175218	1,697
1556924_at	**ALS2CR10**	amyotrophic lateral sclerosis 2 (juvenile) chromosome region, candidate 10	0,0175888	1,140
206995_x_at	**SCARF1**	scavenger receptor class F, member 1	0,0176219	1,141
232474_at	**PLXNA2**	plexin A2	0,0176714	1,201
232395_x_at	**AGBL3**	ATP/GTP binding protein-like 3	0,0177049	1,151
200052_s_at	**ILF2**	interleukin enhancer binding factor 2, 45kDa	0,0177244	1,639
231608_at	**LOC402059**	NA	0,0177731	1,193
227894_at	**LOC197336**	NA	0,0178244	1,209
212787_at	**YLPM1**	YLP motif containing 1	0,0178502	1,073
219092_s_at	**C9orf12**	chromosome 9 open reading frame 12	0,0178618	1,211
224654_at	**ZNF596**	zinc finger protein 596	0,0179175	1,237
218339_at	**MRPL22**	mitochondrial ribosomal protein L22	0,0179208	1,158
1555086_at	**STAT5B**	signal transducer and activator of transcription 5B	0,0179623	1,191
209595_at	**GTF2F2**	general transcription factor IIF, polypeptide 2, 30kDa	0,0180010	1,243
244192_x_at	**USP4**	ubiquitin specific peptidase 4 (proto-oncogene)	0,0180879	1,147
217915_s_at	**C15orf15**	chromosome 15 open reading frame 15	0,0181179	1,337
212116_at	**RFP**	ret finger protein	0,0181179	1,211
213302_at	**PFAS**	phosphoribosylformylglycinamidine synthase (FGAR amidotransferase)	0,0181674	1,309
212859_x_at	**MT1E**	metallothionein 1E (functional)	0,0182939	1,887
225324_at	**C20orf155**	chromosome 20 open reading frame 155	0,0183053	1,459
220018_at	**CBLL1**	Cas-Br-M (murine) ecotropic retroviral transforming sequence-like 1	0,0183053	1,277
1560060_s_at	**VPS37C**	vacuolar protein sorting 37C (yeast)	0,0183053	1,418
235970_at	**MLR1**	NA	0,0183525	1,239
1554509_a_at	**C10orf97**	chromosome 10 open reading frame 97	0,0183676	1,320

216559_x_at	**LOC120364**	NA	0,0183676	1,363
240271_at	**MTMR3**	myotubularin related protein 3	0,0184008	1,179
204459_at	**CSTF2**	cleavage stimulation factor, 3' pre-RNA, subunit 2, 64kDa	0,0184266	1,225
216228_s_at	**WDHD1**	WD repeat and HMG-box DNA binding protein 1	0,0184832	1,698
202614_at	**SLC30A9**	solute carrier family 30 (zinc transporter), member 9	0,0184955	1,185
229436_x_at	**CXorf53**	chromosome X open reading frame 53	0,0185098	1,250
223687_s_at	**LY6K**	lymphocyte antigen 6 complex, locus K	0,0185378	2,735
211750_x_at	**TUBA6**	NA	0,0186171	1,288
225514_at	**CIDEB**	cell death-inducing DFFA-like effector b	0,0186676	1,514
224177_s_at	**CXorf26**	chromosome X open reading frame 26	0,0186676	1,666
228774_at	**C9orf81**	chromosome 9 open reading frame 81	0,0187041	1,264
209717_at	**EVI5**	ecotropic viral integration site 5	0,0187230	1,332
203144_s_at	**KIAA0040**	KIAA0040	0,0187636	1,304
216212_s_at	**DKC1**	dyskeratosis congenita 1, dyskerin	0,0187907	1,505
203032_s_at	**FH**	fumarate hydratase	0,0187907	2,059
223071_at	**IER3IP1**	immediate early response 3 interacting protein 1	0,0187907	1,353
200776_s_at	**LOC151579**	NA	0,0187907	1,801
228176_at	**C9orf47**	chromosome 9 open reading frame 47	0,0188125	3,674
1553181_at	**DDX31**	DEAD (Asp-Glu-Ala-Asp) box polypeptide 31	0,0188125	1,143
205300_s_at	**U1SNRNPBP**	NA	0,0188125	1,173
231065_at	**PDE6D**	phosphodiesterase 6D, cGMP-specific, rod, delta	0,0188412	1,153
212474_at	**KIAA0241**	NA	0,0188573	1,218
227856_at	**FLJ39370**	NA	0,0188985	1,479
223608_at	**EFCAB2**	EF-hand calcium binding domain 2	0,0189088	1,505
1554029_a_at	**KIAA0372**	KIAA0372	0,0189088	2,089
236132_at	**CREB3**	cAMP responsive element binding protein 3	0,0189120	1,326
222021_x_at	**SDHAL2**	succinate dehydrogenase complex, subunit A, flavoprotein-like 2	0,0190123	1,144
1564520_s_at	**SKB1**	SKB1 homolog (S. pombe)	0,0190392	1,268
227766_at	**LIG4**	ligase IV, DNA, ATP-dependent	0,0190419	1,204
241384_x_at	**MGC40579**	NA	0,0190511	1,322
202401_s_at	**SRF**	serum response factor (c-fos serum response element-binding transcription factor)	0,0190511	1,279
226635_at	**LOC401504**	NA	0,0191336	1,175
201603_at	**PPP1R12A**	protein phosphatase 1, regulatory (inhibitor) subunit 12A	0,0191612	1,262
217814_at	**GK001**	NA	0,0191995	1,815
203347_s_at	**MTF2**	metal response element binding transcription factor 2	0,0191995	1,461
207130_at	**PRKCBP1**	protein kinase C binding protein 1	0,0191995	1,241
200828_s_at	**ZNF207**	zinc finger protein 207	0,0191995	1,583
212010_s_at	**H41**	NA	0,0191998	1,116
232889_at	**LOC389281**	NA	0,0191998	1,757
225652_at	**STK25**	serine/threonine kinase 25 (STE20 homolog, yeast)	0,0193559	1,278
243618_s_at	**LOC152485**	NA	0,0193570	1,437
220948_s_at	**ATP1A1**	ATPase, Na+/K+ transporting, alpha 1 polypeptide	0,0194107	1,325
235471_at	**C10orf72**	chromosome 10 open reading frame 72	0,0194107	1,356
219399_at	**LIN7C**	lin-7 homolog C (C. elegans)	0,0194107	1,433
226276_at	**MGC23909**	NA	0,0194107	1,318
209514_s_at	**RAB27A**	RAB27A, member RAS oncogene family	0,0194107	2,311
210790_s_at	**SARA1**	SAR1a gene homolog 1 (S. cerevisiae)	0,0194107	1,885
1554115_at	**LOC133874**	NA	0,0194186	1,147
216323_x_at	**H2-ALPHA**	NA	0,0194259	1,415
211184_s_at	**USH1C**	Usher syndrome 1C (autosomal recessive, severe)	0,0194259	1,089
212502_at	**C10orf22**	chromosome 10 open reading frame 22	0,0194280	1,293
218231_at	**NAGK**	N-acetylglucosamine kinase	0,0194280	1,273
208708_x_at	**EIF5**	eukaryotic translation initiation factor 5	0,0194495	1,374

206881_s_at	**LILRA3**	leukocyte immunoglobulin-like receptor, subfamily A (without TM domain), member 3	0,0194857	1,241
232801_at	**LOC375748**	NA	0,0194857	1,099
212003_at	**C1orf144**	chromosome 1 open reading frame 144	0,0195015	2,029
1560871_a_at	**FLJ42986**	NA	0,0195546	1,314
233175_at	**ZNF272**	zinc finger protein 272	0,0196507	1,106
210059_s_at	**MAPK13**	mitogen-activated protein kinase 13	0,0196578	1,312
202306_at	**POLR2G**	polymerase (RNA) II (DNA directed) polypeptide G	0,0196649	1,151
235033_at	**NPEPL1**	aminopeptidase-like 1	0,0196713	1,320
217092_x_at	**LOC388401**	NA	0,0196913	1,075
217019_at	**RPS4X**	ribosomal protein S4, X-linked	0,0197052	1,185
221682_s_at	**PCDHGB6**	protocadherin gamma subfamily B, 6	0,0197378	1,130
203102_s_at	**MGAT2**	mannosyl (alpha-1,6-)-glycoprotein beta-1,2-N-acetylglucosaminyltransferase	0,0198526	1,325
232126_at	**COQ2**	coenzyme Q2 homolog, prenyltransferase (yeast)	0,0198611	1,132
229514_at	**C14orf118**	chromosome 14 open reading frame 118	0,0199198	1,250
225847_at	**AADACL1**	arylacetamide deacetylase-like 1	0,0200268	2,679
226118_at	**ADCY3**	adenylate cyclase 3	0,0200268	1,759
1553219_a_at	**AMMECR1**	Alport syndrome, mental retardation, midface hypoplasia and elliptocytosis chromosomal region, gene 1	0,0200268	2,015
218782_s_at	**ATAD2**	ATPase family, AAA domain containing 2	0,0200268	2,982
207508_at	**ATP5G3**	ATP synthase, H+ transporting, mitochondrial F0 complex, subunit c (subunit 9) isoform 3	0,0200268	1,260
209430_at	**BTAF1**	BTAF1 RNA polymerase II, B-TFIID transcription factor-associated, 170kDa (Mot1 homolog, S. cerevisiae)	0,0200268	1,402
225604_s_at	**C9orf19**	chromosome 9 open reading frame 19	0,0200268	1,840
223513_at	**CENPJ**	centromere protein J	0,0200268	2,093
202462_s_at	**DDX46**	DEAD (Asp-Glu-Ala-Asp) box polypeptide 46	0,0200268	1,581
201386_s_at	**DHX15**	DEAH (Asp-Glu-Ala-His) box polypeptide 15	0,0200268	1,404
209214_s_at	**EWSR1**	Ewing sarcoma breakpoint region 1	0,0200268	1,497
1565717_s_at	**FUS**	c("fusion (involved in t(12", "16) in malignant liposarcoma)")	0,0200268	4,672
219015_s_at	**GLT28D1**	glycosyltransferase 28 domain containing 1	0,0200268	1,819
218343_s_at	**GTF3C3**	general transcription factor IIIC, polypeptide 3, 102kDa	0,0200268	1,189
219122_s_at	**ICF45**	NA	0,0200268	1,357
218219_s_at	**LANCL2**	LanC lantibiotic synthetase component C-like 2 (bacterial)	0,0200268	1,685
227320_at	**LOC144347**	NA	0,0200268	3,633
217693_x_at	**LOC388335**	NA	0,0200268	1,388
229640_x_at	**LOC440128**	NA	0,0200268	1,393
213461_at	**NUDT21**	nudix (nucleoside diphosphate linked moiety X)-type motif 21	0,0200268	1,840
201192_s_at	**PITPNA**	phosphatidylinositol transfer protein, alpha	0,0200268	1,397
218681_s_at	**SDF2L1**	stromal cell-derived factor 2-like 1	0,0200268	2,491
223209_s_at	**SELS**	NA	0,0200268	1,497
200890_s_at	**SSR1**	signal sequence receptor, alpha (translocon-associated protein alpha)	0,0200268	1,962
205812_s_at	**SULT1C2**	sulfotransferase family, cytosolic, 1C, member 2	0,0200268	1,753
209404_s_at	**TMED7**	transmembrane emp24 protein transport domain containing 7	0,0200268	1,453
201351_s_at	**YME1L1**	YME1-like 1 (S. cerevisiae)	0,0200268	1,171
209989_at	**ZNF268**	zinc finger protein 268	0,0200268	1,340
241472_at	**DMXL1**	Dmx-like 1	0,0200533	1,127
1554737_at	**FBN2**	fibrillin 2 (congenital contractural arachnodactyly)	0,0200604	1,152
218422_s_at	**C13orf10**	chromosome 13 open reading frame 10	0,0201534	1,193
225049_at	**BLOC1S2**	biogenesis of lysosome-related organelles complex-1, subunit 2	0,0201750	1,265
235300_x_at	**RCHY1**	ring finger and CHY zinc finger domain containing 1	0,0202146	1,324
226801_s_at	**DOCK9**	dedicator of cytokinesis 9	0,0202284	1,137
201530_x_at	**EIF4A1**	eukaryotic translation initiation factor 4A, isoform 1	0,0202832	1,533
209136_s_at	**USP10**	ubiquitin specific peptidase 10	0,0203136	1,459
204240_s_at	**SMC2L1**	SMC2 structural maintenance of chromosomes 2-like 1 (yeast)	0,0204137	2,071

227375_at	**ANKRD13C**	ankyrin repeat domain 13C	0,0204257	1,436
223101_s_at	**ARPC5L**	actin related protein 2/3 complex, subunit 5-like	0,0204257	1,478
238795_at	**C10orf18**	chromosome 10 open reading frame 18	0,0204257	1,989
243106_at	**CLEC12A**	C-type lectin domain family 12, member A	0,0204257	1,101
218333_at	**DERL2**	Der1-like domain family, member 2	0,0204257	1,320
214263_x_at	**DOK4**	docking protein 4	0,0204257	1,213
202852_s_at	**FLJ11506**	NA	0,0204257	1,585
1560599_a_at	**FLJ14640**	NA	0,0204257	1,549
212789_at	**hCAP-D3**	NA	0,0204257	1,528
200942_s_at	**HSBP1**	heat shock factor binding protein 1	0,0204257	1,458
224744_at	**IMPAD1**	inositol monophosphatase domain containing 1	0,0204257	1,473
204709_s_at	**KIF23**	kinesin family member 23	0,0204257	4,079
235096_at	**LEO1**	Leo1, Paf1/RNA polymerase II complex component, homolog (S. cerevisiae)	0,0204257	1,385
210732_s_at	**LGALS8**	lectin, galactoside-binding, soluble, 8 (galectin 8)	0,0204257	1,863
219588_s_at	**LUZP5**	leucine zipper protein 5	0,0204257	2,657
216237_s_at	**MCM5**	MCM5 minichromosome maintenance deficient 5, cell division cycle 46 (S. cerevisiae)	0,0204257	2,858
1552312_a_at	**MFAP3**	microfibrillar-associated protein 3	0,0204257	1,707
209208_at	**MPDU1**	mannose-P-dolichol utilization defect 1	0,0204257	1,765
223996_s_at	**MRPL30**	mitochondrial ribosomal protein L30	0,0204257	1,619
201521_s_at	**NCBP2**	nuclear cap binding protein subunit 2, 20kDa	0,0204257	1,577
218036_x_at	**NMD3**	NMD3 homolog (S. cerevisiae)	0,0204257	1,475
202352_s_at	**PSMD12**	proteasome (prosome, macropain) 26S subunit, non-ATPase, 12	0,0204257	1,421
226089_at	**RABL3**	RAB, member of RAS oncogene family-like 3	0,0204257	1,709
208801_at	**SRP72**	signal recognition particle 72kDa	0,0204257	1,263
221847_at	**LOC441653**	NA	0,0204270	1,144
236535_at	**SMC6L1**	SMC6 structural maintenance of chromosomes 6-like 1 (yeast)	0,0204281	1,292
223021_x_at	**C6orf55**	chromosome 6 open reading frame 55	0,0204600	1,387
224377_s_at	**RAB18**	RAB18, member RAS oncogene family	0,0205907	1,238
1556255_a_at	**LOC400756**	NA	0,0206635	1,121
213505_s_at	**SFRS14**	splicing factor, arginine/serine-rich 14	0,0206635	1,184
1563513_at	**SYTL4**	synaptotagmin-like 4 (granuphilin-a)	0,0206635	1,160
221510_s_at	**GLS**	glutaminase	0,0206636	1,420
200875_s_at	**NOL5A**	nucleolar protein 5A (56kDa with KKE/D repeat)	0,0207523	1,336
218884_s_at	**FLJ13220**	NA	0,0207763	1,563
217196_s_at	**CAMSAP1L1**	calmodulin regulated spectrin-associated protein 1-like 1	0,0207940	1,182
218039_at	**NUSAP1**	nucleolar and spindle associated protein 1	0,0208455	6,013
201304_at	**NDUFA5**	NADH dehydrogenase (ubiquinone) 1 alpha subcomplex, 5, 13kDa	0,0208800	1,265
205024_s_at	**RAD51**	RAD51 homolog (RecA homolog, E. coli) (S. cerevisiae)	0,0208800	2,455
207386_at	**CYP7B1**	cytochrome P450, family 7, subfamily B, polypeptide 1	0,0209355	1,228
33323_r_at	**SFN**	stratifin	0,0209601	1,345
205329_s_at	**SNX4**	sorting nexin 4	0,0209779	1,513
208757_at	**TMED9**	transmembrane emp24 protein transport domain containing 9	0,0209779	2,224
217144_at	**UBB**	ubiquitin B	0,0209779	1,395
238131_at	**PHC2**	polyhomeotic-like 2 (Drosophila)	0,0209830	1,161
233800_at	**AP2B1**	adaptor-related protein complex 2, beta 1 subunit	0,0210402	1,286
203023_at	**HSPC111**	NA	0,0210576	1,392
214529_at	**TSHB**	thyroid stimulating hormone, beta	0,0210759	1,104
202924_s_at	**PLAGL2**	pleiomorphic adenoma gene-like 2	0,0211377	1,164
1566249_at	**KIAA1102**	NA	0,0211957	1,189
239782_at	**RBP1**	retinol binding protein 1, cellular	0,0212750	1,170
217725_x_at	**SERBP1**	SERPINE1 mRNA binding protein 1	0,0213413	1,504
239738_at	**DACH2**	dachshund homolog 2 (Drosophila)	0,0214208	1,081
217755_at	**HN1**	hematological and neurological expressed 1	0,0214208	1,709

224334_s_at	**MRPL51**	mitochondrial ribosomal protein L51	0,0214208	1,258
202348_s_at	**TOR1A**	torsin family 1, member A (torsin A)	0,0214208	1,214
218838_s_at	**FLJ12788**	NA	0,0214247	1,141
211297_s_at	**CDK7**	cyclin-dependent kinase 7 (MO15 homolog, Xenopus laevis, cdk-activating kinase)	0,0214277	1,307
218401_s_at	**ZNF281**	zinc finger protein 281	0,0214489	1,466
1569073_x_at	**SMARCA4**	SWI/SNF related, matrix associated, actin dependent regulator of chromatin, subfamily a, member 4	0,0214631	1,210
203418_at	**CCNA2**	cyclin A2	0,0214687	6,372
201695_s_at	**NP**	nucleoside phosphorylase	0,0214687	3,082
223076_s_at	**NSUN2**	NOL1/NOP2/Sun domain family, member 2	0,0214687	1,356
202558_s_at	**STCH**	stress 70 protein chaperone, microsome-associated, 60kDa	0,0214687	1,663
225982_at	**UBTF**	upstream binding transcription factor, RNA polymerase I	0,0214687	1,325
241946_at	**ZDHHC21**	zinc finger, DHHC-type containing 21	0,0214687	1,741
207855_s_at	**CLCC1**	chloride channel CLIC-like 1	0,0214906	1,217
236738_at	**LOC401097**	NA	0,0214997	7,421
53912_at	**SNX11**	sorting nexin 11	0,0214997	1,158
218478_s_at	**ZCCHC8**	zinc finger, CCHC domain containing 8	0,0216169	1,310
219623_at	**ACTR5**	ARP5 actin-related protein 5 homolog (yeast)	0,0216395	1,109
219148_at	**PBK**	PDZ binding kinase	0,0216410	7,299
235039_x_at	**LIN9**	lin-9 homolog (C. elegans)	0,0216653	1,263
222448_s_at	**CMPK**	cytidylate kinase	0,0216668	1,162
201057_s_at	**GOLGB1**	golgi autoantigen, golgin subfamily b, macrogolgin (with transmembrane signal), 1	0,0216790	1,163
241696_at	**C9orf39**	chromosome 9 open reading frame 39	0,0216908	1,218
223320_s_at	**ABCB10**	ATP-binding cassette, sub-family B (MDR/TAP), member 10	0,0216910	1,449
206445_s_at	**HRMT1L2**	HMT1 hnRNP methyltransferase-like 2 (S. cerevisiae)	0,0216910	2,424
219711_at	**ZNF586**	zinc finger protein 586	0,0216910	1,189
218768_at	**NUP107**	nucleoporin 107kDa	0,0217142	1,261
225910_at	**HELZ**	helicase with zinc finger	0,0217403	1,232
204201_s_at	**PTPN13**	protein tyrosine phosphatase, non-receptor type 13 (APO-1/CD95 (Fas)-associated phosphatase)	0,0217403	1,429
202602_s_at	**HTATSF1**	HIV TAT specific factor 1	0,0217408	1,193
1554730_at	**MCTP1**	NA	0,0217408	1,114
206473_at	**MBTPS2**	membrane-bound transcription factor peptidase, site 2	0,0217465	1,486
1569677_a_at	**C8orf45**	chromosome 8 open reading frame 45	0,0217866	1,080
223341_s_at	**SCOC**	short coiled-coil protein	0,0218148	1,405
205020_s_at	**ARL4**	ADP-ribosylation factor-like 4	0,0218338	1,583
218726_at	**DKFZp762E1312**	NA	0,0218338	4,593
203105_s_at	**DNM1L**	dynamin 1-like	0,0218338	1,430
218193_s_at	**GOLT1B**	golgi transport 1 homolog B (S. cerevisiae)	0,0218338	1,846
201784_s_at	**SMAP**	NA	0,0218338	1,229
201946_s_at	**CCT2**	chaperonin containing TCP1, subunit 2 (beta)	0,0218822	1,775
202543_s_at	**GMFB**	glia maturation factor, beta	0,0218822	1,969
201277_s_at	**HNRPAB**	heterogeneous nuclear ribonucleoprotein A/B	0,0218822	1,537
212355_at	**KIAA0323**	KIAA0323	0,0218822	1,373
1554768_a_at	**MAD2L1**	MAD2 mitotic arrest deficient-like 1 (yeast)	0,0218822	4,617
225208_s_at	**MGC2560**	NA	0,0218822	1,302
224330_s_at	**MRPL27**	mitochondrial ribosomal protein L27	0,0218822	1,311
214697_s_at	**ROD1**	ROD1 regulator of differentiation 1 (S. pombe)	0,0218822	1,321
205520_at	**STRN**	striatin, calmodulin binding protein	0,0218822	1,409
218235_s_at	**UTP11L**	UTP11-like, U3 small nucleolar ribonucleoprotein, (yeast)	0,0218822	1,281
222703_s_at	**YRDC**	yrdC domain containing (E.coli)	0,0218822	1,457
223191_at	**C14orf112**	chromosome 14 open reading frame 112	0,0218952	1,287
205909_at	**POLE2**	polymerase (DNA directed), epsilon 2 (p59 subunit)	0,0218952	2,680
202779_s_at	**UBE2S**	ubiquitin-conjugating enzyme E2S	0,0218952	2,048

210048_at	**NAPG**	N-ethylmaleimide-sensitive factor attachment protein, gamma	0,0219297	1,727
222386_s_at	**COPZ1**	coatomer protein complex, subunit zeta 1	0,0219392	1,342
225501_at	**PHF6**	PHD finger protein 6	0,0219456	1,165
225110_at	**FLJ10826**	NA	0,0219563	1,357
219297_at	**WDR44**	WD repeat domain 44	0,0219923	1,187
213743_at	**CCNT2**	cyclin T2	0,0220373	1,213
223035_s_at	**FARSLB**	phenylalanine-tRNA synthetase-like, beta subunit	0,0220762	1,244
236769_at	**LOC158402**	NA	0,0220762	1,377
218442_at	**TTC4**	tetratricopeptide repeat domain 4	0,0220762	1,176
236889_at	**VPS37B**	vacuolar protein sorting 37B (yeast)	0,0220762	1,109
208394_x_at	**ESM1**	endothelial cell-specific molecule 1	0,0220778	4,369
223247_at	**TRG20**	NA	0,0220778	1,537
219713_at	**CARKL**	carbohydrate kinase-like	0,0220861	1,228
216088_s_at	**PSMA7**	proteasome (prosome, macropain) subunit, alpha type, 7	0,0220873	1,362
1561206_at	**KLHL8**	kelch-like 8 (Drosophila)	0,0220949	1,125
207956_x_at	**APRIN**	androgen-induced proliferation inhibitor	0,0221084	1,147
1554344_s_at	**AQP12A**	aquaporin 12A	0,0221125	1,088
1567214_a_at	**PNN**	pinin, desmosome associated protein	0,0221539	1,264
235644_at	**FLJ32745**	NA	0,0221822	1,276
212315_s_at	**NUP210**	nucleoporin 210kDa	0,0221822	1,227
231852_at	**THEX1**	three prime histone mRNA exonuclease 1	0,0221859	1,533
211801_x_at	**MFN1**	mitofusin 1	0,0222037	1,682
213989_x_at	**C21orf18**	chromosome 21 open reading frame 18	0,0222056	1,231
34260_at	**KIAA0683**	NA	0,0222056	1,345
202918_s_at	**PREI3**	preimplantation protein 3	0,0222056	1,366
222310_at	**SFRS15**	splicing factor, arginine/serine-rich 15	0,0222056	1,550
210995_s_at	**TRIM23**	tripartite motif-containing 23	0,0222056	1,485
201222_s_at	**RAD23B**	RAD23 homolog B (S. cerevisiae)	0,0222483	1,493
202502_at	**ACADM**	acyl-Coenzyme A dehydrogenase, C-4 to C-12 straight chain	0,0222631	1,186
232902_s_at	**RARSL**	arginyl-tRNA synthetase-like	0,0222641	1,230
224441_s_at	**USP45**	ubiquitin specific peptidase 45	0,0222909	1,216
244738_at	**BRWD3**	bromodomain and WD repeat domain containing 3	0,0223146	1,153
222164_at	**FGFR1**	fibroblast growth factor receptor 1 (fms-related tyrosine kinase 2, Pfeiffer syndrome)	0,0223592	1,314
206902_s_at	**ENDOGL1**	endonuclease G-like 1	0,0223679	1,288
204444_at	**KIF11**	kinesin family member 11	0,0223679	5,673
206316_s_at	**KNTC1**	kinetochore associated 1	0,0223679	1,773
201239_s_at	**SPCS2**	signal peptidase complex subunit 2 homolog (S. cerevisiae)	0,0223679	1,468
224623_at	**THOC3**	THO complex 3	0,0223679	1,326
219985_at	**HS3ST3A1**	heparan sulfate (glucosamine) 3-O-sulfotransferase 3A1	0,0223741	1,689
210156_s_at	**PCMT1**	protein-L-isoaspartate (D-aspartate) O-methyltransferase	0,0223961	1,520
204128_s_at	**RFC3**	replication factor C (activator 1) 3, 38kDa	0,0224096	3,102
233655_s_at	**FAM29A**	family with sequence similarity 29, member A	0,0224157	1,602
212280_x_at	**ATG4B**	ATG4 autophagy related 4 homolog B (S. cerevisiae)	0,0224770	1,159
1553467_at	**FLJ32742**	NA	0,0224770	1,207
242586_at	**FSD1L**	FSD1-like	0,0225278	1,457
208304_at	**CCR3**	chemokine (C-C motif) receptor 3	0,0226156	1,200
1556744_a_at	**LOC440971**	NA	0,0226337	1,654
235296_at	**EIF5A2**	eukaryotic translation initiation factor 5A2	0,0226416	1,450
202690_s_at	**SNRPD1**	small nuclear ribonucleoprotein D1 polypeptide 16kDa	0,0226652	1,568
226752_at	**UNQ1912**	NA	0,0226652	1,301
218150_at	**ARL5**	ADP-ribosylation factor-like 5	0,0226748	1,309
227383_at	**LOC440668**	NA	0,0226748	1,929
217843_s_at	**MED4**	mediator of RNA polymerase II transcription, subunit 4 homolog (yeast)	0,0226748	1,266

1554482_a_at	**SARA2**	SAR1a gene homolog 2 (S. cerevisiae)	0,0226748	1,272
222406_s_at	**PNRC2**	proline-rich nuclear receptor coactivator 2	0,0227628	1,762
201529_s_at	**RPA1**	replication protein A1, 70kDa	0,0227682	1,233
203594_at	**RTCD1**	RNA terminal phosphate cyclase domain 1	0,0228044	1,266
201457_x_at	**BUB3**	BUB3 budding uninhibited by benzimidazoles 3 homolog (yeast)	0,0228373	1,622
205338_s_at	**DCT**	dopachrome tautomerase (dopachrome delta-isomerase, tyrosine-related protein 2)	0,0228373	1,210
1552417_a_at	**NEDD1**	neural precursor cell expressed, developmentally down-regulated 1	0,0228373	1,489
210236_at	**PPFIA1**	protein tyrosine phosphatase, receptor type, f polypeptide (PTPRF), interacting protein (liprin), alpha 1	0,0228373	1,302
223225_s_at	**SEH1L**	SEH1-like (S. cerevisiae)	0,0228373	1,656
234140_s_at	**STIM2**	stromal interaction molecule 2	0,0228373	1,487
226319_s_at	**THOC4**	THO complex 4	0,0228687	2,200
215072_x_at	**C10orf137**	chromosome 10 open reading frame 137	0,0229079	1,123
1558015_s_at	**ACTR2**	ARP2 actin-related protein 2 homolog (yeast)	0,0230671	2,786
202095_s_at	**BIRC5**	baculoviral IAP repeat-containing 5 (survivin)	0,0230671	4,256
203213_at	**CDC2**	cell division cycle 2, G1 to S and G2 to M	0,0230671	8,836
222680_s_at	**DTL**	denticleless homolog (Drosophila)	0,0230671	4,771
222039_at	**LOC146909**	NA	0,0230671	3,559
228809_at	**LOC442467**	NA	0,0230671	1,692
213672_at	**MARS**	methionine-tRNA synthetase	0,0230671	1,422
200610_s_at	**NCL**	nucleolin	0,0230671	1,255
1554691_a_at	**PACSIN2**	protein kinase C and casein kinase substrate in neurons 2	0,0230671	1,768
202732_at	**PKIG**	protein kinase (cAMP-dependent, catalytic) inhibitor gamma	0,0230671	1,143
212220_at	**PSME4**	proteasome (prosome, macropain) activator subunit 4	0,0230671	1,948
210813_s_at	**XRCC4**	X-ray repair complementing defective repair in Chinese hamster cells 4	0,0230671	2,153
218739_at	**ABHD5**	abhydrolase domain containing 5	0,0230733	1,543
1552619_a_at	**ANLN**	anillin, actin binding protein (scraps homolog, Drosophila)	0,0230733	5,050
220342_x_at	**C1orf22**	chromosome 1 open reading frame 22	0,0230733	1,468
207165_at	**HMMR**	hyaluronan-mediated motility receptor (RHAMM)	0,0230733	7,814
226631_at	**LOC399818**	NA	0,0230733	1,503
1558292_s_at	**PIGW**	phosphatidylinositol glycan, class W	0,0230733	1,812
218576_s_at	**DUSP12**	dual specificity phosphatase 12	0,0231003	1,170
232383_at	**TFEC**	transcription factor EC	0,0231238	1,323
214831_at	**MED28**	mediator of RNA polymerase II transcription, subunit 28 homolog (yeast)	0,0231727	1,312
203755_at	**BUB1B**	BUB1 budding uninhibited by benzimidazoles 1 homolog beta (yeast)	0,0231790	6,419
202521_at	**CTCF**	CCCTC-binding factor (zinc finger protein)	0,0231842	1,154
232615_at	**LOC440666**	NA	0,0231842	1,827
201629_s_at	**ACP1**	acid phosphatase 1, soluble	0,0231983	1,684
1555797_a_at	**ARPC5**	actin related protein 2/3 complex, subunit 5, 16kDa	0,0232089	1,701
232013_at	**C9orf102**	chromosome 9 open reading frame 102	0,0232089	1,380
213524_s_at	**G0S2**	G0/G1switch 2	0,0232089	1,544
1554026_a_at	**MYO10**	myosin X	0,0232089	1,922
211139_s_at	**NAB1**	NGFI-A binding protein 1 (EGR1 binding protein 1)	0,0232089	1,930
225485_at	**TSGA14**	testis specific, 14	0,0232089	1,749
37079_at	**YDD19**	NA	0,0232089	1,775
218349_s_at	**ZWILCH**	Zwilch, kinetochore associated, homolog (Drosophila)	0,0232089	2,470
206184_at	**CRKL**	v-crk sarcoma virus CT10 oncogene homolog (avian)-like	0,0232153	1,395
243256_at	**MKNK1**	MAP kinase interacting serine/threonine kinase 1	0,0232227	1,254
210145_at	**PLA2G4A**	phospholipase A2, group IVA (cytosolic, calcium-dependent)	0,0232627	2,020
200751_s_at	**HNRPC**	heterogeneous nuclear ribonucleoprotein C (C1/C2)	0,0232773	1,834
209478_at	**STRA13**	stimulated by retinoic acid 13 homolog (mouse)	0,0232773	1,339
202003_s_at	**ACAA2**	acetyl-Coenzyme A acyltransferase 2 (mitochondrial 3-oxoacyl-Coenzyme A thiolase)	0,0232869	1,410

228829_at	**ATF7**	activating transcription factor 7	0,0232869	1,318
207829_s_at	**BNIP1**	BCL2/adenovirus E1B 19kDa interacting protein 1	0,0232869	1,274
208653_s_at	**CD164**	CD164 antigen, sialomucin	0,0232869	1,516
241874_at	**LOC492311**	NA	0,0232869	1,256
222460_s_at	**NIP30**	NA	0,0232869	1,248
201577_at	**NME1**	non-metastatic cells 1, protein (NM23A) expressed in	0,0232869	1,738
219110_at	**NOLA1**	nucleolar protein family A, member 1 (H/ACA small nucleolar RNPs)	0,0232869	1,435
201608_s_at	**PWP1**	PWP1 homolog (S. cerevisiae)	0,0232869	1,371
1555247_a_at	**RAPGEF6**	Rap guanine nucleotide exchange factor (GEF) 6	0,0232869	1,435
219021_at	**RNF121**	ring finger protein 121	0,0232869	1,407
210932_s_at	**RNF6**	ring finger protein (C3H2C3 type) 6	0,0232869	1,911
209080_x_at	**TXNL2**	thioredoxin-like 2	0,0232869	1,438
201483_s_at	**SUPT4H1**	suppressor of Ty 4 homolog 1 (S. cerevisiae)	0,0233020	1,899
208967_s_at	**AK2**	adenylate kinase 2	0,0233593	1,446
1555278_a_at	**CKAP5**	cytoskeleton associated protein 5	0,0233593	1,239
224864_at	**SRA1**	steroid receptor RNA activator 1	0,0233593	1,512
225769_at	**COG6**	component of oligomeric golgi complex 6	0,0233693	1,452
216392_s_at	**SEC23IP**	SEC23 interacting protein	0,0233693	1,403
238856_s_at	**PANK2**	pantothenate kinase 2 (Hallervorden-Spatz syndrome)	0,0233785	1,263
206788_s_at	**CBFB**	core-binding factor, beta subunit	0,0233800	2,046
216483_s_at	**C19orf10**	chromosome 19 open reading frame 10	0,0233875	1,807
201686_x_at	**API5**	apoptosis inhibitor 5	0,0233890	1,456
225694_at	**CRK7**	NA	0,0234030	1,322
209503_s_at	**PSMC5**	proteasome (prosome, macropain) 26S subunit, ATPase, 5	0,0234030	1,264
206098_at	**ZNF482**	zinc finger protein 482	0,0234030	2,324
1555988_a_at	**LOC126536**	NA	0,0234256	1,102
226880_at	**NUCKS1**	nuclear casein kinase and cyclin-dependent kinase substrate 1	0,0234340	1,395
236465_at	**RNF175**	ring finger protein 175	0,0234398	1,159
235125_x_at	**FAM73A**	family with sequence similarity 73, member A	0,0234494	1,235
1554362_at	**BTG4**	B-cell translocation gene 4	0,0234809	1,125
202660_at	**ITPR2**	inositol 1,4,5-triphosphate receptor, type 2	0,0235093	1,772
201701_s_at	**PGRMC2**	progesterone receptor membrane component 2	0,0235219	1,220
228990_at	**C1orf79**	chromosome 1 open reading frame 79	0,0235504	1,499
204419_x_at	**HBG2**	hemoglobin, gamma G	0,0235504	1,126
201896_s_at	**PSRC1**	proline/serine-rich coiled-coil 1	0,0235578	2,057
239826_at	**C18orf45**	chromosome 18 open reading frame 45	0,0236325	1,238
226614_s_at	**C8orf13**	chromosome 8 open reading frame 13	0,0236325	1,421
221781_s_at	**DNAJC10**	DnaJ (Hsp40) homolog, subfamily C, member 10	0,0236325	1,362
218680_x_at	**HYPK**	NA	0,0236325	1,341
211450_s_at	**MSH6**	mutS homolog 6 (E. coli)	0,0236325	1,272
201970_s_at	**NASP**	nuclear autoantigenic sperm protein (histone-binding)	0,0236325	1,897
235609_at	**BRIP1**	BRCA1 interacting protein C-terminal helicase 1	0,0236544	3,604
1554433_a_at	**ZNF146**	zinc finger protein 146	0,0236759	1,974
202542_s_at	**SCYE1**	small inducible cytokine subfamily E, member 1 (endothelial monocyte-activating)	0,0236856	1,174
239372_at	**ZZZ3**	zinc finger, ZZ-type containing 3	0,0237128	1,134
201232_s_at	**PSMD13**	proteasome (prosome, macropain) 26S subunit, non-ATPase, 13	0,0237329	1,403
218252_at	**CKAP2**	cytoskeleton associated protein 2	0,0237471	2,043
214512_s_at	**SUB1**	SUB1 homolog (S. cerevisiae)	0,0237669	1,426
202712_s_at	**CKMT1B**	creatine kinase, mitochondrial 1B	0,0237739	1,109
396_f_at	**EPOR**	erythropoietin receptor	0,0238603	1,182
203978_at	**NUBP1**	nucleotide binding protein 1 (MinD homolog, E. coli)	0,0238634	1,288
209589_s_at	**EPHB2**	EPH receptor B2	0,0238670	1,251
228996_at	**RC3H1**	ring finger and CCCH-type zinc finger domains 1	0,0239347	1,450

208822_s_at	**DAP3**	death associated protein 3	0,0239475	1,133
228454_at	**MLR2**	NA	0,0239475	1,307
227936_at	**TMEM68**	transmembrane protein 68	0,0239475	1,281
214141_x_at	**SFRS7**	splicing factor, arginine/serine-rich 7, 35kDa	0,0240391	1,572
208079_s_at	**STK6**	serine/threonine kinase 6	0,0240391	4,658
216591_s_at	**SDHC**	succinate dehydrogenase complex, subunit C, integral membrane protein, 15kDa	0,0240516	1,810
230288_at	**FGF14**	fibroblast growth factor 14	0,0240687	1,153
205235_s_at	**MPHOSPH1**	M-phase phosphoprotein 1	0,0240687	1,797
239785_at	**DZIP1L**	DAZ interacting protein 1-like	0,0240812	1,318
218014_at	**PCNT1**	pericentrin 1	0,0240886	1,233
214596_at	**CHRM3**	cholinergic receptor, muscarinic 3	0,0241051	1,262
202535_at	**FADD**	Fas (TNFRSF6)-associated via death domain	0,0241340	1,519
221597_s_at	**HSPC171**	NA	0,0241340	1,458
221258_s_at	**KIF18A**	kinesin family member 18A	0,0241673	2,475
226859_at	**GNG10**	guanine nucleotide binding protein (G protein), gamma 10	0,0241750	1,289
225310_at	**RBMX**	RNA binding motif protein, X-linked	0,0241750	1,729
219936_s_at	**GPR87**	G protein-coupled receptor 87	0,0241842	1,081
231911_at	**KIAA1189**	KIAA1189	0,0242232	2,364
1558253_x_at	**ZNF587**	zinc finger protein 587	0,0242484	1,277
230791_at	**NFIB**	nuclear factor I/B	0,0243139	1,224
201202_at	**PCNA**	proliferating cell nuclear antigen	0,0243208	1,480
1555004_a_at	**RBL1**	retinoblastoma-like 1 (p107)	0,0243208	1,205
202819_s_at	**TCEB3**	transcription elongation factor B (SIII), polypeptide 3 (110kDa, elongin A)	0,0243354	1,524
201697_s_at	**DNMT1**	DNA (cytosine-5-)-methyltransferase 1	0,0243604	1,359
210065_s_at	**UPK1B**	uroplakin 1B	0,0243838	1,160
211661_x_at	**PTAFR**	platelet-activating factor receptor	0,0243999	1,366
214790_at	**SENP6**	SUMO1/sentrin specific peptidase 6	0,0244074	1,268
239835_at	**KBTBD8**	kelch repeat and BTB (POZ) domain containing 8	0,0244185	1,554
222393_s_at	**MAK3**	Mak3 homolog (S. cerevisiae)	0,0245332	1,455
201953_at	**CIB1**	calcium and integrin binding 1 (calmyrin)	0,0245690	1,439
226153_s_at	**CNOT6L**	CCR4-NOT transcription complex, subunit 6-like	0,0245690	1,311
209391_at	**DPM2**	dolichyl-phosphate mannosyltransferase polypeptide 2, regulatory subunit	0,0245690	1,190
212720_at	**PAPOLA**	poly(A) polymerase alpha	0,0245690	1,896
204146_at	**RAD51AP1**	RAD51 associated protein 1	0,0245690	3,669
206499_s_at	**RCC1**	regulator of chromosome condensation 1	0,0245690	1,501
204768_s_at	**FEN1**	flap structure-specific endonuclease 1	0,0245889	2,197
204207_s_at	**RNGTT**	RNA guanylyltransferase and 5'-phosphatase	0,0246440	1,685
206055_s_at	**SNRPA1**	small nuclear ribonucleoprotein polypeptide A'	0,0246440	1,533
233011_at	**ANXA1**	annexin A1	0,0246460	2,056
235707_at	**LOC221710**	NA	0,0246764	1,486
200712_s_at	**MAPRE1**	microtubule-associated protein, RP/EB family, member 1	0,0246837	1,350
224867_at	**RPS14**	ribosomal protein S14	0,0246926	1,156
206502_s_at	**INSM1**	insulinoma-associated 1	0,0246987	1,179
228652_at	**FLJ38288**	NA	0,0247089	1,187
219306_at	**KIF15**	kinesin family member 15	0,0247299	2,490
211686_s_at	**RBM13**	RNA binding motif protein 13	0,0247299	1,160
217713_x_at	**DKFZP566N034**	NA	0,0247341	1,194
220547_s_at	**FAM35A**	family with sequence similarity 35, member A	0,0247453	1,256
1552266_at	**ADAM32**	ADAM metallopeptidase domain 32	0,0247572	1,286
224776_at	**AGPAT6**	1-acylglycerol-3-phosphate O-acyltransferase 6 (lysophosphatidic acid acyltransferase, zeta)	0,0247675	1,190
222047_s_at	**ARS2**	NA	0,0247946	1,275
218270_at	**MRPL24**	mitochondrial ribosomal protein L24	0,0247998	1,227

204426_at	**TMED2**	transmembrane emp24 domain trafficking protein 2	0,0248558	2,284
209619_at	**CD74**	CD74 antigen (invariant polypeptide of major histocompatibility complex, class II antigen-associated)	0,0248985	4,305
208721_s_at	**ANAPC5**	anaphase promoting complex subunit 5	0,0250096	1,644
244835_at	**C16orf52**	chromosome 16 open reading frame 52	0,0250275	1,180
1562030_at	**LOC284898**	NA	0,0250275	1,182
201267_s_at	**PSMC3**	proteasome (prosome, macropain) 26S subunit, ATPase, 3	0,0250352	1,358
205436_s_at	**H2AFX**	H2A histone family, member X	0,0250354	1,545
219017_at	**ETNK1**	ethanolamine kinase 1	0,0250358	1,275
210009_s_at	**GOSR2**	golgi SNAP receptor complex member 2	0,0250855	1,467
208766_s_at	**HNRPR**	heterogeneous nuclear ribonucleoprotein R	0,0251072	1,277
202870_s_at	**CDC20**	CDC20 cell division cycle 20 homolog (S. cerevisiae)	0,0251176	4,969
211212_s_at	**ORC5L**	origin recognition complex, subunit 5-like (yeast)	0,0251179	1,551
226335_at	**RPS6KA3**	ribosomal protein S6 kinase, 90kDa, polypeptide 3	0,0251250	1,502
231990_at	**USP15**	ubiquitin specific peptidase 15	0,0251703	1,376
214595_at	**KCNG1**	potassium voltage-gated channel, subfamily G, member 1	0,0251706	1,572
237105_at	**PRKRA**	protein kinase, interferon-inducible double stranded RNA dependent activator	0,0251772	1,223
203358_s_at	**EZH2**	enhancer of zeste homolog 2 (Drosophila)	0,0252185	2,145
205867_at	**PTPN11**	protein tyrosine phosphatase, non-receptor type 11 (Noonan syndrome 1)	0,0252196	1,294
1555399_a_at	**DUSP16**	dual specificity phosphatase 16	0,0252552	1,126
207147_at	**DLX2**	distal-less homeo box 2	0,0252688	1,979
203177_x_at	**TFAM**	transcription factor A, mitochondrial	0,0252750	1,350
225404_at	**LOC113444**	NA	0,0252780	1,156
207904_s_at	**LNPEP**	leucyl/cystinyl aminopeptidase	0,0253083	1,555
225081_s_at	**CDCA7L**	cell division cycle associated 7-like	0,0253149	1,840
201043_s_at	**ANP32A**	acidic (leucine-rich) nuclear phosphoprotein 32 family, member A	0,0253269	3,144
219493_at	**SHCBP1**	SHC SH2-domain binding protein 1	0,0253269	5,803
203743_s_at	**TDG**	thymine-DNA glycosylase	0,0253269	1,349
206631_at	**PTGER2**	prostaglandin E receptor 2 (subtype EP2), 53kDa	0,0253398	3,560
233442_at	**PDZRN3**	PDZ domain containing RING finger 3	0,0253444	1,287
1555039_a_at	**ABCC4**	ATP-binding cassette, sub-family C (CFTR/MRP), member 4	0,0253696	1,529
204812_at	**ZW10**	ZW10, kinetochore associated, homolog (Drosophila)	0,0253870	1,478
220060_s_at	**FLJ20641**	NA	0,0253968	1,480
207749_s_at	**PPP2R3A**	protein phosphatase 2 (formerly 2A), regulatory subunit B'', alpha	0,0254116	1,325
201573_s_at	**ETF1**	eukaryotic translation termination factor 1	0,0254197	1,243
229650_s_at	**MGC2747**	NA	0,0254491	1,388
239238_at	**SMARCC1**	SWI/SNF related, matrix associated, actin dependent regulator of chromatin, subfamily c, member 1	0,0254560	1,267
1552519_at	**ACVR1C**	activin A receptor, type IC	0,0254578	1,238
217778_at	**SLC39A1**	solute carrier family 39 (zinc transporter), member 1	0,0254910	1,186
216349_at	**LOC341651**	NA	0,0255050	1,136
215489_x_at	**HOMER3**	homer homolog 3 (Drosophila)	0,0255380	1,699
201614_s_at	**RUVBL1**	RuvB-like 1 (E. coli)	0,0255380	1,295
204826_at	**CCNF**	cyclin F	0,0255404	1,646
1554339_a_at	**COG3**	component of oligomeric golgi complex 3	0,0255404	1,955
216942_s_at	**CD58**	CD58 antigen, (lymphocyte function-associated antigen 3)	0,0255515	1,357
221685_s_at	**FLJ20364**	NA	0,0255653	2,660
205442_at	**MFAP3L**	microfibrillar-associated protein 3-like	0,0255653	1,861
1569053_at	**AP3M2**	adaptor-related protein complex 3, mu 2 subunit	0,0256553	1,354
223909_s_at	**HDAC8**	histone deacetylase 8	0,0256598	1,123
201535_at	**UBL3**	ubiquitin-like 3	0,0257051	1,297
202166_s_at	**PPP1R2**	protein phosphatase 1, regulatory (inhibitor) subunit 2	0,0257500	1,418
1558816_at	**ZNF664**	zinc finger protein 664	0,0257685	1,226
213007_at	**FLJ10719**	NA	0,0257695	3,384

1564277_a_at	**LOC389757**	NA	0,0257809	1,186
203721_s_at	**WDR50**	WD repeat domain 50	0,0258097	1,222
206979_at	**C8B**	complement component 8, beta polypeptide	0,0258409	1,129
210041_s_at	**PGM3**	phosphoglucomutase 3	0,0258524	1,906
222077_s_at	**RACGAP1**	Rac GTPase activating protein 1	0,0258524	2,853
235345_at	**RUFY2**	RUN and FYVE domain containing 2	0,0258524	1,428
202705_at	**CCNB2**	cyclin B2	0,0258575	4,362
225268_at	**KPNA4**	karyopherin alpha 4 (importin alpha 3)	0,0258869	1,205
212242_at	**TUBA1**	tubulin, alpha 1 (testis specific)	0,0258905	1,880
218119_at	**TIMM23**	translocase of inner mitochondrial membrane 23 homolog (yeast)	0,0259041	1,213
204191_at	**IFNAR1**	interferon (alpha, beta and omega) receptor 1	0,0259369	1,293
211456_x_at	**LOC440737**	NA	0,0259504	1,974
204120_s_at	**ADK**	adenosine kinase	0,0259922	1,376
200984_s_at	**CD59**	CD59 antigen p18-20 (antigen identified by monoclonal antibodies 16.3A5, EJ16, EJ30, EL32 and G344)	0,0259995	1,179
224634_at	**GPATC4**	G patch domain containing 4	0,0260039	1,330
210455_at	**C10orf28**	chromosome 10 open reading frame 28	0,0260339	1,379
215373_x_at	**FLJ12151**	NA	0,0260407	1,177
203208_s_at	**MTFR1**	mitochondrial fission regulator 1	0,0260463	1,153
211434_s_at	**CCRL2**	chemokine (C-C motif) receptor-like 2	0,0261162	1,330
225355_at	**DKFZP761M1511**	NA	0,0261162	2,232
219544_at	**FLJ22624**	NA	0,0261162	2,377
1554606_at	**FLJ36090**	NA	0,0261162	1,464
204195_s_at	**PKNOX1**	PBX/knotted 1 homeobox 1	0,0261162	1,249
217775_s_at	**RDH11**	retinol dehydrogenase 11 (all-trans and 9-cis)	0,0261162	1,269
201420_s_at	**WDR77**	WD repeat domain 77	0,0261162	1,329
219239_s_at	**ZNF654**	zinc finger protein 654	0,0261162	1,820
209055_s_at	**CDC5L**	CDC5 cell division cycle 5-like (S. pombe)	0,0261493	1,380
200841_s_at	**EPRS**	glutamyl-prolyl-tRNA synthetase	0,0261493	1,758
225042_s_at	**C12orf22**	chromosome 12 open reading frame 22	0,0261591	1,160
91617_at	**DGCR8**	DiGeorge syndrome critical region gene 8	0,0261726	1,281
205831_at	**CD2**	CD2 antigen (p50), sheep red blood cell receptor	0,0262108	1,150
238895_at	**PTGES3**	prostaglandin E synthase 3 (cytosolic)	0,0262108	1,165
238220_at	**UTX**	ubiquitously transcribed tetratricopeptide repeat, X chromosome	0,0262205	1,088
219037_at	**CGI-115**	NA	0,0263057	1,306
210639_s_at	**ATG5**	ATG5 autophagy related 5 homolog (S. cerevisiae)	0,0263153	1,277
222149_x_at	**LOC283768**	NA	0,0263287	1,164
203672_x_at	**TPMT**	thiopurine S-methyltransferase	0,0263664	1,168
223274_at	**TCF19**	transcription factor 19 (SC1)	0,0263668	2,551
230263_s_at	**DOCK5**	dedicator of cytokinesis 5	0,0264504	1,408
207298_at	**SLC17A3**	solute carrier family 17 (sodium phosphate), member 3	0,0265021	1,178
238020_at	**SLC26A5**	solute carrier family 26, member 5 (prestin)	0,0265021	1,175
1552953_a_at	**NEUROD2**	neurogenic differentiation 2	0,0265185	1,155
223300_s_at	**FLJ23518**	NA	0,0265452	1,508
1553982_a_at	**RAB7B**	RAB7B, member RAS oncogene family	0,0265457	1,524
202396_at	**TCERG1**	transcription elongation regulator 1	0,0265457	1,455
205292_s_at	**HNRPA2B1**	heterogeneous nuclear ribonucleoprotein A2/B1	0,0265844	1,607
224740_at	**LOC441075**	NA	0,0266313	1,181
209797_at	**TMEM4**	transmembrane protein 4	0,0266313	1,345
220083_x_at	**UCHL5**	ubiquitin carboxyl-terminal hydrolase L5	0,0266313	1,357
230316_at	**LOC200312**	NA	0,0266443	1,844
218212_s_at	**MOCS2**	molybdenum cofactor synthesis 2	0,0266583	1,151
204012_s_at	**LCMT2**	leucine carboxyl methyltransferase 2	0,0266769	1,136
209507_at	**RPA3**	replication protein A3, 14kDa	0,0266769	1,317

211098_x_at	**TMCO1**	transmembrane and coiled-coil domains 1	0,0267056	1,256
212432_at	**GRPEL1**	GrpE-like 1, mitochondrial (E. coli)	0,0267359	1,282
219918_s_at	**ASPM**	asp (abnormal spindle)-like, microcephaly associated (Drosophila)	0,0267722	6,898
201101_s_at	**BCLAF1**	BCL2-associated transcription factor 1	0,0267816	1,885
218542_at	**C10orf3**	chromosome 10 open reading frame 3	0,0267816	4,491
208798_x_at	**GOLGA8A**	golgi autoantigen, golgin subfamily a, 8A	0,0267816	1,573
202293_at	**STAG1**	stromal antigen 1	0,0267816	1,322
208638_at	**ATP6V1C2**	ATPase, H+ transporting, lysosomal 42kDa, V1 subunit C isoform 2	0,0267961	1,370
225687_at	**C20orf129**	chromosome 20 open reading frame 129	0,0267961	6,999
225071_at	**C6orf68**	chromosome 6 open reading frame 68	0,0267961	1,459
210642_at	**CCIN**	calicin	0,0267961	1,518
203968_s_at	**CDC6**	CDC6 cell division cycle 6 homolog (S. cerevisiae)	0,0267961	2,068
222398_s_at	**EFTUD2**	elongation factor Tu GTP binding domain containing 2	0,0267961	1,137
224407_s_at	**MASK**	NA	0,0267961	1,775
222231_s_at	**PRO1855**	NA	0,0267961	1,712
221810_at	**RAB15**	RAB15, member RAS onocogene family	0,0267961	1,301
210568_s_at	**RECQL**	RecQ protein-like (DNA helicase Q1-like)	0,0267961	1,795
201476_s_at	**RRM1**	ribonucleotide reductase M1 polypeptide	0,0267961	2,212
1553910_at	**FLJ32833**	NA	0,0268023	1,104
40225_at	**GAK**	cyclin G associated kinase	0,0268199	1,267
235022_at	**C18orf19**	chromosome 18 open reading frame 19	0,0268331	1,241
1553781_at	**MGC14289**	NA	0,0269623	1,088
208438_s_at	**FGR**	Gardner-Rasheed feline sarcoma viral (v-fgr) oncogene homolog	0,0269629	1,297
235620_x_at	**ZMYM5**	zinc finger, MYM-type 5	0,0269629	1,265
229594_at	**FLJ39441**	NA	0,0269718	1,330
243424_at	**SOX6**	SRY (sex determining region Y)-box 6	0,0269718	1,085
233999_s_at	**FLJ12571**	NA	0,0269841	1,665
235653_s_at	**THAP6**	THAP domain containing 6	0,0269841	1,342
213865_at	**DCBLD2**	discoidin, CUB and LCCL domain containing 2	0,0269940	2,086
217752_s_at	**CNDP2**	CNDP dipeptidase 2 (metallopeptidase M20 family)	0,0270315	1,475
200593_s_at	**HNRPU**	heterogeneous nuclear ribonucleoprotein U (scaffold attachment factor A)	0,0270315	1,451
209032_s_at	**IGSF4**	immunoglobulin superfamily, member 4	0,0270315	1,466
209520_s_at	**NCBP1**	nuclear cap binding protein subunit 1, 80kDa	0,0270315	1,441
209131_s_at	**SNAP23**	synaptosomal-associated protein, 23kDa	0,0270315	2,018
220305_at	**MGC3260**	NA	0,0270347	1,829
242117_at	**MAP3K3**	mitogen-activated protein kinase kinase kinase 3	0,0270560	1,065
1552719_at	**CASC4**	cancer susceptibility candidate 4	0,0270675	1,271
235447_at	**TRUB1**	TruB pseudouridine (psi) synthase homolog 1 (E. coli)	0,0270675	1,521
217850_at	**GNL3**	guanine nucleotide binding protein-like 3 (nucleolar)	0,0270850	1,401
213449_at	**POP1**	processing of precursor 1, ribonuclease P/MRP subunit (S. cerevisiae)	0,0272081	1,544
1563528_at	**LOC91149**	NA	0,0272470	1,220
211855_s_at	**SLC25A14**	solute carrier family 25 (mitochondrial carrier, brain), member 14	0,0272470	1,385
1553113_s_at	**CDK8**	cyclin-dependent kinase 8	0,0273358	1,212
224508_at	**MGC12916**	NA	0,0273486	1,270
201930_at	**MCM6**	MCM6 minichromosome maintenance deficient 6 (MIS5 homolog, S. pombe) (S. cerevisiae)	0,0274052	1,609
219312_s_at	**ZBTB10**	zinc finger and BTB domain containing 10	0,0274089	1,421
235315_at	**TSC22D1**	TSC22 domain family, member 1	0,0274154	1,446
1555225_at	**C1orf43**	chromosome 1 open reading frame 43	0,0274285	1,529
201898_s_at	**UBE2A**	ubiquitin-conjugating enzyme E2A (RAD6 homolog)	0,0274285	1,385
218741_at	**C22orf18**	chromosome 22 open reading frame 18	0,0274382	1,878
213696_s_at	**MED8**	mediator of RNA polymerase II transcription, subunit 8 homolog (yeast)	0,0274382	1,417
1554397_s_at	**UEV3**	NA	0,0274382	1,587

242157_at	**CHD9**	chromodomain helicase DNA binding protein 9	0,0274419	1,396
1553810_a_at	**KIAA1524**	KIAA1524	0,0274535	1,566
209927_s_at	**C1orf77**	chromosome 1 open reading frame 77	0,0274738	1,502
211150_s_at	**DLAT**	dihydrolipoamide S-acetyltransferase (E2 component of pyruvate dehydrogenase complex)	0,0274738	1,316
217294_s_at	**ENO1**	enolase 1, (alpha)	0,0274738	1,926
1555751_a_at	**GEMIN7**	gem (nuclear organelle) associated protein 7	0,0274738	1,242
222660_s_at	**RNF38**	ring finger protein 38	0,0274814	1,302
1553725_s_at	**ZNF644**	zinc finger protein 644	0,0275093	1,947
227255_at	**PDIK1L**	PDLIM1 interacting kinase 1 like	0,0275194	1,335
201955_at	**CCNC**	cyclin C	0,0275336	1,161
233230_s_at	**KIAA1458**	NA	0,0275336	1,765
228195_at	**MGC13057**	NA	0,0275336	1,348
213407_at	**PHLPPL**	PH domain and leucine rich repeat protein phosphatase-like	0,0275336	1,352
221423_s_at	**YIPF5**	Yip1 domain family, member 5	0,0275336	1,565
225309_at	**PHF5A**	PHD finger protein 5A	0,0275489	1,641
217979_at	**TSPAN13**	tetraspanin 13	0,0275489	1,922
209363_s_at	**SURB7**	SRB7 suppressor of RNA polymerase B homolog (yeast)	0,0275547	1,261
211089_s_at	**NEK3**	NIMA (never in mitosis gene a)-related kinase 3	0,0275617	1,554
201196_s_at	**AMD1**	adenosylmethionine decarboxylase 1	0,0275936	1,288
239697_x_at	**FLJ42117**	NA	0,0275936	1,329
222645_s_at	**KCTD5**	potassium channel tetramerisation domain containing 5	0,0276011	1,721
223338_s_at	**ATPIF1**	ATPase inhibitory factor 1	0,0276152	1,134
213226_at	**EXOSC9**	exosome component 9	0,0276179	4,455
205966_at	**TAF13**	TAF13 RNA polymerase II, TATA box binding protein (TBP)-associated factor, 18kDa	0,0276499	1,550
1552899_at	**MGC34034**	NA	0,0276704	1,174
209674_at	**CRY1**	cryptochrome 1 (photolyase-like)	0,0276870	1,486
1557984_s_at	**FLJ21908**	NA	0,0276870	1,755
204822_at	**TTK**	TTK protein kinase	0,0277049	6,858
210005_at	**GART**	phosphoribosylglycinamide formyltransferase, phosphoribosylglycinamide synthetase, phosphoribosylaminoimidazole synthetase	0,0277566	1,457
202070_s_at	**IDH3A**	isocitrate dehydrogenase 3 (NAD+) alpha	0,0277566	1,425
236517_at	**MEGF10**	NA	0,0277771	1,575
202127_at	**PRPF4B**	PRP4 pre-mRNA processing factor 4 homolog B (yeast)	0,0277781	1,286
205324_s_at	**FTSJ1**	FtsJ homolog 1 (E. coli)	0,0277977	1,763
202654_x_at	**MARCH7**	membrane-associated ring finger (C3HC4) 7	0,0277977	1,415
1563473_at	**PPP1R16B**	protein phosphatase 1, regulatory (inhibitor) subunit 16B	0,0277977	1,258
208696_at	**CCT5**	chaperonin containing TCP1, subunit 5 (epsilon)	0,0278055	1,247
1555834_at	**UCHL1**	ubiquitin carboxyl-terminal esterase L1 (ubiquitin thiolesterase)	0,0278548	1,182
243402_at	**MGC35023**	NA	0,0278575	1,137
209567_at	**RRS1**	RRS1 ribosome biogenesis regulator homolog (S. cerevisiae)	0,0279022	1,190
203764_at	**DLG7**	discs, large homolog 7 (Drosophila)	0,0279227	7,275
234304_s_at	**IPO11**	importin 11	0,0279227	1,195
217959_s_at	**TRAPPC4**	trafficking protein particle complex 4	0,0279399	1,314
206770_s_at	**SLC35A3**	solute carrier family 35 (UDP-N-acetylglucosamine (UDP-GlcNAc) transporter), member A3	0,0280031	1,211
202200_s_at	**SRPK1**	SFRS protein kinase 1	0,0280168	1,452
222682_s_at	**C5orf3**	chromosome 5 open reading frame 3	0,0280208	1,172
201994_at	**MORF4L2**	mortality factor 4 like 2	0,0280375	1,176
1554868_s_at	**PCNP**	NA	0,0280679	1,347
213836_s_at	**WIPI49**	NA	0,0281104	1,165
219451_at	**MSRB2**	methionine sulfoxide reductase B2	0,0281121	1,309
1552625_a_at	**TRNT1**	tRNA nucleotidyl transferase, CCA-adding, 1	0,0281121	1,452
201633_s_at	**CYB5-M**	NA	0,0281208	1,420

235283_at	**DDX26**	DEAD/H (Asp-Glu-Ala-Asp/His) box polypeptide 26	0,0281208	1,274
229509_at	**MGC33302**	NA	0,0281208	1,375
206113_s_at	**RAB5A**	RAB5A, member RAS oncogene family	0,0281208	1,874
204893_s_at	**ZFYVE9**	zinc finger, FYVE domain containing 9	0,0281208	1,357
238894_at	**RABGAP1L**	RAB GTPase activating protein 1-like	0,0281510	1,130
241345_at	**ZNF265**	zinc finger protein 265	0,0281764	1,212
1554518_at	**FLJ13273**	NA	0,0282191	1,163
228170_at	**OLIG1**	oligodendrocyte transcription factor 1	0,0282259	1,282
220840_s_at	**C1orf112**	chromosome 1 open reading frame 112	0,0282720	1,533
206474_at	**PCTK2**	PCTAIRE protein kinase 2	0,0282898	1,759
205690_s_at	**G10**	NA	0,0282933	1,166
205497_at	**ZNF175**	zinc finger protein 175	0,0283530	1,260
212449_s_at	**LYPLA1**	lysophospholipase I	0,0283777	1,547
213012_at	**NEDD4**	neural precursor cell expressed, developmentally down-regulated 4	0,0284031	1,526
207125_at	**ZNF225**	zinc finger protein 225	0,0284031	1,250
219787_s_at	**ECT2**	epithelial cell transforming sequence 2 oncogene	0,0284101	2,165
201359_at	**COPB**	coatomer protein complex, subunit beta	0,0284221	1,268
209938_at	**TADA2L**	transcriptional adaptor 2 (ADA2 homolog, yeast)-like	0,0284760	1,259
227674_at	**ZNF585A**	zinc finger protein 585A	0,0284960	1,253
219558_at	**ATP13A3**	ATPase type 13A3	0,0285061	2,378
215253_s_at	**DSCR1**	Down syndrome critical region gene 1	0,0285110	2,130
242889_x_at	**FUT8**	fucosyltransferase 8 (alpha (1,6) fucosyltransferase)	0,0285513	1,161
222983_s_at	**PAIP2**	poly(A) binding protein interacting protein 2	0,0285843	1,174
203579_s_at	**SLC7A6OS**	solute carrier family 7, member 6 opposite strand	0,0285976	1,660
208649_s_at	**VCP**	valosin-containing protein	0,0285976	1,273
201504_s_at	**TSN**	translin	0,0286039	1,405
207828_s_at	**CENPF**	centromere protein F, 350/400ka (mitosin)	0,0286213	4,009
201450_s_at	**TIA1**	TIA1 cytotoxic granule-associated RNA binding protein	0,0286213	1,640
204126_s_at	**CDC45L**	CDC45 cell division cycle 45-like (S. cerevisiae)	0,0286355	2,493
229551_x_at	**ZNF367**	zinc finger protein 367	0,0286419	3,122
1555529_at	**RNH1**	ribonuclease/angiogenin inhibitor 1	0,0287048	1,098
206451_at	**FLJ10560**	NA	0,0287435	1,237
203258_at	**DRAP1**	DR1-associated protein 1 (negative cofactor 2 alpha)	0,0287683	1,451
238983_at	**FLJ14001**	NA	0,0287894	1,110
218503_at	**KIAA1797**	KIAA1797	0,0288076	1,232
205842_s_at	**JAK2**	Janus kinase 2 (a protein tyrosine kinase)	0,0288114	1,354
222567_s_at	**RKHD2**	ring finger and KH domain containing 2	0,0288114	1,524
205046_at	**CENPE**	centromere protein E, 312kDa	0,0288199	4,015
217811_at	**SELT**	NA	0,0288206	1,435
204385_at	**KYNU**	kynureninase (L-kynurenine hydrolase)	0,0288305	2,152
1554883_a_at	**ERCC8**	excision repair cross-complementing rodent repair deficiency, complementation group 8	0,0288735	1,398
221194_s_at	**LOC51136**	NA	0,0288735	1,615
214507_s_at	**EXOSC2**	exosome component 2	0,0288812	1,355
202647_s_at	**NRAS**	neuroblastoma RAS viral (v-ras) oncogene homolog	0,0288812	1,711
1560916_a_at	**DPY19L1**	dpy-19-like 1 (C. elegans)	0,0288962	1,785
218009_s_at	**PRC1**	protein regulator of cytokinesis 1	0,0289272	3,140
212296_at	**PSMD14**	proteasome (prosome, macropain) 26S subunit, non-ATPase, 14	0,0289444	1,184
228131_at	**ERCC1**	excision repair cross-complementing rodent repair deficiency, complementation group 1 (includes overlapping antisense sequence)	0,0289813	1,515
1552792_at	**SOCS4**	suppressor of cytokine signaling 4	0,0289842	1,336
239045_at	**ERN1**	endoplasmic reticulum to nucleus signalling 1	0,0290814	1,123
215207_x_at	**LOC389850**	NA	0,0291136	1,732
1553954_at	**ALG14**	asparagine-linked glycosylation 14 homolog (yeast)	0,0291260	1,162

223586_at	**ARNTL2**	aryl hydrocarbon receptor nuclear translocator-like 2	0,0291260	1,673
205773_at	**CPEB3**	cytoplasmic polyadenylation element binding protein 3	0,0291260	1,237
216913_s_at	**KIAA0690**	KIAA0690	0,0291260	1,198
225062_at	**LOC389831**	NA	0,0291260	1,672
216632_at	**NAV3**	neuron navigator 3	0,0291260	1,094
244614_at	**TFG**	TRK-fused gene	0,0291260	1,182
213212_x_at	**LOC440295**	NA	0,0291489	1,301
218676_s_at	**PCTP**	phosphatidylcholine transfer protein	0,0291666	1,119
209470_s_at	**GPM6A**	glycoprotein M6A	0,0291961	1,155
202278_s_at	**SPTLC1**	serine palmitoyltransferase, long chain base subunit 1	0,0291992	1,336
235812_at	**FLJ38101**	NA	0,0292198	1,229
1552717_s_at	**KIAA0470L**	KIAA0470-like	0,0292621	1,627
222548_s_at	**MAP4K4**	mitogen-activated protein kinase kinase kinase kinase 4	0,0292987	1,366
1554408_a_at	**TK1**	thymidine kinase 1, soluble	0,0293084	3,540
200008_s_at	**GDI2**	GDP dissociation inhibitor 2	0,0293168	1,637
218241_at	**GOLGA5**	golgi autoantigen, golgin subfamily a, 5	0,0293233	1,208
222029_x_at	**HKE2**	HLA class II region expressed gene KE2	0,0293233	1,385
217765_at	**NRBP**	nuclear receptor binding protein	0,0293233	1,634
205157_s_at	**KRT17**	keratin 17	0,0293741	1,884
235411_at	**PGBD1**	piggyBac transposable element derived 1	0,0293741	1,315
213342_at	**YAP1**	Yes-associated protein 1, 65kDa	0,0293805	1,480
204171_at	**RPS6KB1**	ribosomal protein S6 kinase, 70kDa, polypeptide 1	0,0293845	1,149
238524_at	**DENND2D**	DENN/MADD domain containing 2D	0,0294284	1,112
222700_at	**ARL6IP2**	ADP-ribosylation factor-like 6 interacting protein 2	0,0294350	1,271
1563572_at	**LOC152274**	NA	0,0294350	1,069
229367_s_at	**GIMAP6**	GTPase, IMAP family member 6	0,0294511	1,142
218564_at	**RFWD3**	ring finger and WD repeat domain 3	0,0294631	1,420
213170_at	**GPX7**	glutathione peroxidase 7	0,0294633	1,543
243118_at	**C6orf182**	chromosome 6 open reading frame 182	0,0294682	1,195
214700_x_at	**RIF1**	RAP1 interacting factor homolog (yeast)	0,0294682	1,222
58916_at	**KCTD14**	potassium channel tetramerisation domain containing 14	0,0295174	1,139
208381_s_at	**SGPL1**	sphingosine-1-phosphate lyase 1	0,0295567	1,598
1565752_at	**FGD2**	FYVE, RhoGEF and PH domain containing 2	0,0296884	1,086
218598_at	**RINT-1**	NA	0,0297329	1,537
210184_at	**ITGAX**	integrin, alpha X (antigen CD11C (p150), alpha polypeptide)	0,0297902	1,474
225899_x_at	**LOC440395**	NA	0,0297902	1,470
209408_at	**KIF2C**	kinesin family member 2C	0,0298015	4,646
200054_at	**ZNF259**	zinc finger protein 259	0,0298015	1,406
1558014_s_at	**MLSTD2**	male sterility domain containing 2	0,0298091	1,789
239143_x_at	**RNF138**	ring finger protein 138	0,0298140	1,416
225711_at	**ARL6IP6**	ADP-ribosylation-like factor 6 interacting protein 6	0,0298361	1,234
223381_at	**CDCA1**	cell division cycle associated 1	0,0298495	5,974
202797_at	**SACM1L**	SAC1 suppressor of actin mutations 1-like (yeast)	0,0298786	1,148
211190_x_at	**CD84**	CD84 antigen (leukocyte antigen)	0,0298950	1,380
234620_at	**LOC221875**	NA	0,0298984	1,113
201974_s_at	**C7orf28A**	chromosome 7 open reading frame 28A	0,0298987	1,149
236281_x_at	**HTR7**	5-hydroxytryptamine (serotonin) receptor 7 (adenylate cyclase-coupled)	0,0299476	1,487
212815_at	**ASCC3**	activating signal cointegrator 1 complex subunit 3	0,0299495	1,248
242458_at	**RALGPS2**	Ral GEF with PH domain and SH3 binding motif 2	0,0299495	1,432
241988_x_at	**SSH2**	slingshot homolog 2 (Drosophila)	0,0299613	1,156
1558697_a_at	**LKAP**	NA	0,0299632	1,233
227720_at	**ANKRD13B**	ankyrin repeat domain 13B	0,0000000	0,654

226314_at	**D4ST1**	dermatan 4 sulfotransferase 1	0,0000000	0,638
213771_at	**IRF2BP1**	interferon regulatory factor 2 binding protein 1	0,0000000	0,668
35160_at	**LDB1**	LIM domain binding 1	0,0000000	0,713
227937_at	**LOC339344**	NA	0,0000000	0,659
206234_s_at	**MMP17**	matrix metallopeptidase 17 (membrane-inserted)	0,0000000	0,670
44696_at	**TBC1D13**	TBC1 domain family, member 13	0,0000000	0,790
1555881_s_at	**LZTS2**	leucine zipper, putative tumor suppressor 2	0,0000313	0,774
224821_at	**MGC15429**	NA	0,0000313	0,506
237209_s_at	**NFRKB**	nuclear factor related to kappaB binding protein	0,0000313	0,721
227159_at	**LGP1**	NA	0,0000517	0,535
210482_x_at	**MAP2K5**	mitogen-activated protein kinase kinase 5	0,0000517	0,700
209253_at	**SCAM-1**	NA	0,0001600	0,608
221216_s_at	**SCMH1**	sex comb on midleg homolog 1 (Drosophila)	0,0001761	0,770
226849_at	**DENND1A**	DENN/MADD domain containing 1A	0,0002160	0,697
220314_at	**LW-1**	NA	0,0002160	0,743
209557_s_at	**NCDN**	neurochondrin	0,0002160	0,725
212050_at	**WIRE**	NA	0,0002160	0,708
233022_at	**TRPM3**	transient receptor potential cation channel, subfamily M, member 3	0,0002256	0,820
203295_s_at	**ATP1A2**	ATPase, Na+/K+ transporting, alpha 2 (+) polypeptide	0,0002561	0,760
47083_at	**C7orf26**	chromosome 7 open reading frame 26	0,0002711	0,743
235442_at	**FLJ22965**	NA	0,0002711	0,530
217717_s_at	**YWHAB**	tyrosine 3-monooxygenase/tryptophan 5-monooxygenase activation protein, beta polypeptide	0,0002732	0,707
212090_at	**GRINA**	glutamate receptor, ionotropic, N-methyl D-asparate-associated protein 1 (glutamate binding)	0,0002773	0,532
231865_at	**KIAA1602**	NA	0,0002773	0,674
201046_s_at	**RAD23A**	RAD23 homolog A (S. cerevisiae)	0,0003110	0,733
212547_at	**FLJ35348**	NA	0,0003180	0,774
209973_at	**NFKBIL1**	nuclear factor of kappa light polypeptide gene enhancer in B-cells inhibitor-like 1	0,0003180	0,712
218307_at	**RSAD1**	radical S-adenosyl methionine domain containing 1	0,0003403	0,681
231317_at	**TSNARE1**	t-SNARE domain containing 1	0,0003716	0,770
221966_at	**C11ORF4**	NA	0,0003910	0,742
218903_s_at	**MGC2731**	NA	0,0004401	0,677
213529_at	**ZNF688**	zinc finger protein 688	0,0004401	0,755
218251_at	**MID1IP1**	MID1 interacting protein 1 (gastrulation specific G12-like (zebrafish))	0,0004661	0,597
202958_at	**PTPN9**	protein tyrosine phosphatase, non-receptor type 9	0,0004756	0,784
206008_at	**TGM1**	transglutaminase 1 (K polypeptide epidermal type I, protein-glutamine-gamma-glutamyltransferase)	0,0004756	0,820
236258_at	**C20orf151**	chromosome 20 open reading frame 151	0,0005337	0,749
238679_at	**MESDC2**	mesoderm development candidate 2	0,0005337	0,707
210208_x_at	**BAT3**	HLA-B associated transcript 3	0,0005504	0,765
231372_at	**LOC153328**	NA	0,0005607	0,841
226377_at	**NFIC**	nuclear factor I/C (CCAAT-binding transcription factor)	0,0005607	0,553
221005_s_at	**PTDSS2**	phosphatidylserine synthase 2	0,0005607	0,783
221725_at	**WASF2**	WAS protein family, member 2	0,0005784	0,726
218825_at	**EGFL7**	EGF-like-domain, multiple 7	0,0005796	0,583
227093_at	**USP36**	ubiquitin specific peptidase 36	0,0005796	0,760
212218_s_at	**FASN**	fatty acid synthase	0,0005899	0,437
208242_at	**RAX**	retina and anterior neural fold homeobox	0,0006358	0,770
242200_at	**THSD6**	thrombospondin, type I, domain containing 6	0,0006502	0,805
213081_at	**ZNF297**	zinc finger protein 297	0,0006545	0,776
222007_s_at	**FKBP8**	FK506 binding protein 8, 38kDa	0,0006629	0,803
218225_at	**SITPEC**	NA	0,0006629	0,802
225560_at	**POMT2**	protein-O-mannosyltransferase 2	0,0007567	0,739

203271_s_at	**UNC119**	unc-119 homolog (C. elegans)	0,0007683	0,780
224411_at	**PLA2G12B**	phospholipase A2, group XIIB	0,0007794	0,885
220748_s_at	**ZNF580**	zinc finger protein 580	0,0007794	0,562
201360_at	**CST3**	cystatin C (amyloid angiopathy and cerebral hemorrhage)	0,0007902	0,713
226173_at	**OATL1**	ornithine aminotransferase-like 1	0,0008349	0,845
213204_at	**PARC**	NA	0,0008788	0,771
211217_s_at	**KCNQ1**	potassium voltage-gated channel, KQT-like subfamily, member 1	0,0009482	0,873
227325_at	**LOC255783**	NA	0,0009673	0,642
238863_x_at	**COG8**	component of oligomeric golgi complex 8	0,0009703	0,763
215235_at	**SPTAN1**	spectrin, alpha, non-erythrocytic 1 (alpha-fodrin)	0,0010125	0,741
1561820_at	**SCN8A**	sodium channel, voltage gated, type VIII, alpha	0,0010525	0,827
206982_at	**CRYBA1**	crystallin, beta A1	0,0010893	0,842
203933_at	**RAB11FIP3**	RAB11 family interacting protein 3 (class II)	0,0011371	0,742
212369_at	**ZNF384**	zinc finger protein 384	0,0011580	0,850
218862_at	**ASB13**	ankyrin repeat and SOCS box-containing 13	0,0012157	0,748
230033_at	**LOC352909**	NA	0,0012157	0,833
231522_at	**C20orf144**	chromosome 20 open reading frame 144	0,0012162	0,843
221866_at	**TFEB**	transcription factor EB	0,0012162	0,792
204585_s_at	**L1CAM**	L1 cell adhesion molecule	0,0012460	0,812
215678_at	**LOC440792**	NA	0,0012460	0,799
231176_at	**MGC70924**	NA	0,0012460	0,692
212518_at	**PIP5K1C**	phosphatidylinositol-4-phosphate 5-kinase, type I, gamma	0,0012460	0,655
241486_at	**LOC441347**	NA	0,0013125	0,783
1568683_at	**MGC23284**	NA	0,0013910	0,800
229617_x_at	**AP2A1**	adaptor-related protein complex 2, alpha 1 subunit	0,0014226	0,766
235430_at	**C14orf43**	chromosome 14 open reading frame 43	0,0014456	0,791
237840_at	**LOC388948**	NA	0,0014456	0,827
230145_at	**DUS3L**	dihydrouridine synthase 3-like (S. cerevisiae)	0,0014542	0,811
224528_s_at	**KCNIP2**	Kv channel interacting protein 2	0,0014688	0,787
230070_at	**CNIH2**	cornichon homolog 2 (Drosophila)	0,0014697	0,833
222228_s_at	**FLJ20013**	NA	0,0014814	0,747
204144_s_at	**PIGQ**	phosphatidylinositol glycan, class Q	0,0014977	0,705
207612_at	**WNT8B**	wingless-type MMTV integration site family, member 8B	0,0015437	0,810
238680_at	**MGC2477**	NA	0,0015471	0,855
205333_s_at	**RCE1**	RCE1 homolog, prenyl protein peptidase (S. cerevisiae)	0,0015733	0,807
206452_x_at	**PPP2R4**	protein phosphatase 2A, regulatory subunit B' (PR 53)	0,0015885	0,754
222571_at	**ST6GALNAC6**	ST6 (alpha-N-acetyl-neuraminyl-2,3-beta-galactosyl-1,3)-N-acetylgalactosaminide alpha-2,6-sialyltransferase 6	0,0016171	0,810
55065_at	**MARK4**	MAP/microtubule affinity-regulating kinase 4	0,0016499	0,765
218492_s_at	**THAP7**	THAP domain containing 7	0,0016499	0,779
229660_at	**C16orf55**	chromosome 16 open reading frame 55	0,0016517	0,769
204931_at	**TCF21**	transcription factor 21	0,0016655	0,725
240293_at	**LOC283152**	NA	0,0016672	0,749
215792_s_at	**DNAJC11**	DnaJ (Hsp40) homolog, subfamily C, member 11	0,0016959	0,850
216572_at	**FOXL1**	forkhead box L1	0,0017007	0,844
214503_x_at	**GPR135**	G protein-coupled receptor 135	0,0017413	0,887
237719_x_at	**R7BP**	NA	0,0017413	0,852
221792_at	**RAB6B**	RAB6B, member RAS oncogene family	0,0018412	0,807
238683_at	**ZNF524**	zinc finger protein 524	0,0019074	0,785
203950_s_at	**CLCN6**	chloride channel 6	0,0019684	0,596
212739_s_at	**NME4**	non-metastatic cells 4, protein expressed in	0,0020138	0,704
226454_at	**RENT1**	regulator of nonsense transcripts 1	0,0020442	0,648
228757_at	**SCDR10**	NA	0,0020715	0,836
212559_at	**PRKAR1B**	protein kinase, cAMP-dependent, regulatory, type I, beta	0,0020764	0,854

213709_at	**BHLHB9**	basic helix-loop-helix domain containing, class B, 9	0,0021104	0,512
31637_s_at	**NR1D1**	nuclear receptor subfamily 1, group D, member 1	0,0021725	0,589
218767_at	**REXO4**	REX4, RNA exonuclease 4 homolog (S. cerevisiae)	0,0021875	0,827
210244_at	**CAMP**	cathelicidin antimicrobial peptide	0,0022264	0,821
226598_s_at	**GTPBP5**	GTP binding protein 5 (putative)	0,0022728	0,841
240377_at	**LOC339047**	NA	0,0022930	0,732
211884_s_at	**CIITA**	class II, major histocompatibility complex, transactivator	0,0023040	0,831
223166_x_at	**C9orf86**	chromosome 9 open reading frame 86	0,0023128	0,764
204257_at	**FADS3**	fatty acid desaturase 3	0,0023383	0,664
231287_s_at	**HSPC065**	NA	0,0023719	0,848
216637_at	**ITSN1**	intersectin 1 (SH3 domain protein)	0,0023719	0,817
231018_at	**LOC342979**	NA	0,0023942	0,875
219095_at	**PLA2G4B**	phospholipase A2, group IVB (cytosolic)	0,0023942	0,827
243075_at	**DKFZp547K054**	NA	0,0024132	0,777
219548_at	**ZNF16**	zinc finger protein 16 (KOX 9)	0,0024132	0,815
217923_at	**PEF1**	penta-EF-hand domain containing 1	0,0024152	0,745
220233_at	**FBXO17**	F-box protein 17	0,0024213	0,608
1570138_at	**ASIP**	agouti signaling protein, nonagouti homolog (mouse)	0,0025016	0,887
244379_at	**TMEM16M**	transmembrane protein 16M	0,0025104	0,770
215535_s_at	**AGPAT1**	1-acylglycerol-3-phosphate O-acyltransferase 1 (lysophosphatidic acid acyltransferase, alpha)	0,0025194	0,599
220120_s_at	**EPB41L4A**	erythrocyte membrane protein band 4.1 like 4A	0,0025410	0,791
215544_s_at	**UBOX5**	U-box domain containing 5	0,0025410	0,871
218928_s_at	**SLC37A1**	solute carrier family 37 (glycerol-3-phosphate transporter), member 1	0,0025745	0,796
214246_x_at	**CHRNE**	cholinergic receptor, nicotinic, epsilon polypeptide	0,0026107	0,666
239254_at	**FLJ20753**	NA	0,0026617	0,813
215749_s_at	**GORASP1**	golgi reassembly stacking protein 1, 65kDa	0,0026867	0,800
234312_s_at	**ACSS2**	acyl-CoA synthetase short-chain family member 2	0,0027195	0,790
220064_at	**TTC21B**	tetratricopeptide repeat domain 21B	0,0027369	0,821
242269_at	**FLJ42875**	NA	0,0027820	0,799
211099_s_at	**CNGB1**	cyclic nucleotide gated channel beta 1	0,0027836	0,821
207027_at	**HGFAC**	HGF activator	0,0027884	0,853
207576_x_at	**OXT**	oxytocin, prepro- (neurophysin I)	0,0028026	0,857
1560628_at	**FLJ16124**	NA	0,0028201	0,883
228704_s_at	**CLDN23**	claudin 23	0,0028461	0,823
203853_s_at	**GAB2**	GRB2-associated binding protein 2	0,0028572	0,768
1552310_at	**MGC29937**	NA	0,0028631	0,663
237197_at	**LEMD1**	LEM domain containing 1	0,0028661	0,823
213389_at	**PNKP**	polynucleotide kinase 3'-phosphatase	0,0029449	0,782
1553479_at	**FLJ90805**	NA	0,0030197	0,855
231905_at	**C20orf96**	chromosome 20 open reading frame 96	0,0030211	0,812
215732_s_at	**DTX2**	deltex homolog 2 (Drosophila)	0,0031151	0,713
205459_s_at	**NPAS2**	neuronal PAS domain protein 2	0,0031151	0,791
207410_s_at	**TLX2**	T-cell leukemia, homeobox 2	0,0031360	0,861
205124_at	**MEF2B**	MADS box transcription enhancer factor 2, polypeptide B (myocyte enhancer factor 2B)	0,0031614	0,759
227000_at	**Ells1**	NA	0,0031876	0,772
221443_x_at	**PRLH**	prolactin releasing hormone	0,0032003	0,770
221049_s_at	**POLL**	polymerase (DNA directed), lambda	0,0032561	0,815
201155_s_at	**MFN2**	mitofusin 2	0,0032571	0,820
207525_s_at	**GIPC1**	GIPC PDZ domain containing family, member 1	0,0033274	0,762
211141_s_at	**CNOT3**	CCR4-NOT transcription complex, subunit 3	0,0034415	0,683
243202_at	**LOC284611**	NA	0,0034432	0,846
226207_at	**FLJ39378**	NA	0,0034669	0,768

217849_s_at	**CDC42BPB**	CDC42 binding protein kinase beta (DMPK-like)	0,0034939	0,623
223895_s_at	**EPN3**	epsin 3	0,0035493	0,726
215924_at	**DBC1**	deleted in bladder cancer 1	0,0035937	0,906
205575_at	**C1QL1**	complement component 1, q subcomponent-like 1	0,0036169	0,849
228404_at	**IRX2**	iroquois homeobox protein 2	0,0036613	0,825
227902_at	**FLJ38705**	NA	0,0037421	0,753
209462_at	**APLP1**	amyloid beta (A4) precursor-like protein 1	0,0037666	0,677
208215_x_at	**DRD4**	dopamine receptor D4	0,0037702	0,843
205640_at	**ALDH3B1**	aldehyde dehydrogenase 3 family, member B1	0,0037809	0,749
232367_x_at	**ZNF598**	zinc finger protein 598	0,0038327	0,884
233051_at	**SLITRK2**	SLIT and NTRK-like family, member 2	0,0038517	0,801
1555923_a_at	**C10orf114**	chromosome 10 open reading frame 114	0,0038582	0,798
207670_at	**KRTHB5**	keratin, hair, basic, 5	0,0039797	0,870
209002_s_at	**CALCOCO1**	calcium binding and coiled-coil domain 1	0,0040533	0,696
217265_at	**PLLP**	plasma membrane proteolipid (plasmolipin)	0,0040604	0,884
223588_at	**DKFZP564I0422**	NA	0,0040960	0,524
201853_s_at	**CDC25B**	cell division cycle 25B	0,0040966	0,594
232157_at	**SPRY3**	sprouty homolog 3 (Drosophila)	0,0041732	0,823
225476_at	**BAT4**	HLA-B associated transcript 4	0,0041795	0,728
208256_at	**EFNA2**	ephrin-A2	0,0042291	0,836
205494_at	**LOC55565**	NA	0,0042604	0,845
240275_at	**ARMC3**	armadillo repeat containing 3	0,0043077	0,900
206598_at	**INS**	insulin	0,0043096	0,863
237646_x_at	**TNFRSF25**	tumor necrosis factor receptor superfamily, member 25	0,0043096	0,776
224890_s_at	**LOC389541**	NA	0,0043251	0,740
232792_at	**RNF36**	ring finger protein 36	0,0044011	0,736
209079_x_at	**PCDHGA1**	protocadherin gamma subfamily A, 1	0,0044100	0,643
231728_at	**CAPS**	calcyphosine	0,0044184	0,820
214371_at	**TSSK2**	testis-specific serine kinase 2	0,0044184	0,861
220705_s_at	**ADAMTS7**	ADAM metallopeptidase with thrombospondin type 1 motif, 7	0,0044288	0,818
228616_at	**POLRMT**	polymerase (RNA) mitochondrial (DNA directed)	0,0044292	0,831
232639_at	**LOC90288**	NA	0,0044505	0,899
222942_s_at	**TFB1M**	transcription factor B1, mitochondrial	0,0045000	0,678
231677_at	**AIM1**	absent in melanoma 1	0,0045215	0,877
214253_s_at	**DTNB**	dystrobrevin, beta	0,0045676	0,838
213013_at	**MAPK8IP1**	mitogen-activated protein kinase 8 interacting protein 1	0,0045837	0,793
213177_at	**MAPK8IP3**	mitogen-activated protein kinase 8 interacting protein 3	0,0046340	0,790
205433_at	**BCHE**	butyrylcholinesterase	0,0046860	0,903
220125_at	**DNAI1**	dynein, axonemal, intermediate polypeptide 1	0,0047014	0,793
219013_at	**GALNT11**	UDP-N-acetyl-alpha-D-galactosamine:polypeptide N-acetylgalactosaminyltransferase 11 (GalNAc-T11)	0,0047134	0,743
221066_at	**RLN3R1**	relaxin 3 receptor 1	0,0047166	0,838
217032_at	**FOXD4L1**	forkhead box D4-like 1	0,0047300	0,794
207578_s_at	**HTR4**	5-hydroxytryptamine (serotonin) receptor 4	0,0047405	0,834
203965_at	**USP20**	ubiquitin specific peptidase 20	0,0047405	0,807
219413_at	**ACBD4**	acyl-Coenzyme A binding domain containing 4	0,0048394	0,812
234284_at	**GNG8**	guanine nucleotide binding protein (G protein), gamma 8	0,0048644	0,845
227043_at	**LOC126075**	NA	0,0048701	0,833
216699_s_at	**KLK1**	kallikrein 1, renal/pancreas/salivary	0,0049017	0,884
222175_s_at	**PCQAP**	PC2 (positive cofactor 2, multiprotein complex) glutamine/Q-rich-associated protein	0,0049017	0,699
214203_s_at	**PRODH**	proline dehydrogenase (oxidase) 1	0,0049268	0,807
215293_s_at	**FRAG1**	NA	0,0049362	0,802
233644_at	**KATNAL2**	katanin p60 subunit A-like 2	0,0049387	0,845
236689_at	**RNF151**	ring finger protein 151	0,0049737	0,754

208060_at	**PAX7**	paired box gene 7	0,0050003	0,859
242145_at	**TGFBR3**	transforming growth factor, beta receptor III (betaglycan, 300kDa)	0,0050470	0,900
244477_at	**SLC12A3**	solute carrier family 12 (sodium/chloride transporters), member 3	0,0050795	0,754
244728_at	**LOC130063**	NA	0,0050821	0,856
228236_at	**C20orf54**	chromosome 20 open reading frame 54	0,0051123	0,840
226139_at	**DKFZp761B107**	NA	0,0051123	0,773
223485_at	**HAGHL**	hydroxyacylglutathione hydrolase-like	0,0051123	0,831
216036_x_at	**WDTC1**	WD and tetratricopeptide repeats 1	0,0052562	0,825
224500_s_at	**MON1A**	MON1 homolog A (yeast)	0,0052784	0,830
45297_at	**EHD2**	EH-domain containing 2	0,0052828	0,718
232518_at	**PRIC285**	NA	0,0053116	0,801
220074_at	**MUCDHL**	mucin and cadherin-like	0,0053203	0,819
233571_x_at	**C20orf149**	chromosome 20 open reading frame 149	0,0053399	0,689
204936_at	**MAP4K2**	mitogen-activated protein kinase kinase kinase kinase 2	0,0053722	0,741
207494_s_at	**ZNF76**	zinc finger protein 76 (expressed in testis)	0,0053722	0,811
232349_x_at	**IQWD1**	IQ motif and WD repeats 1	0,0053808	0,784
209957_s_at	**NPPA**	natriuretic peptide precursor A	0,0053808	0,789
203051_at	**BAHD1**	bromo adjacent homology domain containing 1	0,0053893	0,788
233545_at	**INPP5D**	inositol polyphosphate-5-phosphatase, 145kDa	0,0053893	0,813
233165_at	**NCKIPSD**	NCK interacting protein with SH3 domain	0,0054106	0,792
215510_at	**ETV2**	ets variant gene 2	0,0055320	0,787
228265_at	**LOC440869**	NA	0,0055359	0,841
215598_at	**TTC12**	tetratricopeptide repeat domain 12	0,0055547	0,802
1563181_a_at	**C22orf1**	chromosome 22 open reading frame 1	0,0055935	0,854
217567_at	**TGM4**	transglutaminase 4 (prostate)	0,0056495	0,875
203237_s_at	**NOTCH3**	Notch homolog 3 (Drosophila)	0,0056903	0,793
226074_at	**PPM1M**	protein phosphatase 1M (PP2C domain containing)	0,0057044	0,747
219878_s_at	**KLF13**	Kruppel-like factor 13	0,0057701	0,645
239215_at	**LOC401284**	NA	0,0057709	0,844
214029_at	**MGC26694**	NA	0,0057709	0,910
225648_at	**STK35**	serine/threonine kinase 35	0,0057829	0,857
219980_at	**FLJ21106**	NA	0,0057969	0,833
243520_x_at	**ADAM30**	ADAM metallopeptidase domain 30	0,0058157	0,928
218110_at	**XAB2**	XPA binding protein 2	0,0058328	0,630
1566126_at	**FABP6**	fatty acid binding protein 6, ileal (gastrotropin)	0,0059423	0,856
212046_x_at	**MAPK3**	mitogen-activated protein kinase 3	0,0059423	0,793
223141_at	**UCK1**	uridine-cytidine kinase 1	0,0059475	0,826
218359_at	**C20orf98**	chromosome 20 open reading frame 98	0,0059541	0,667
214879_x_at	**USF2**	upstream transcription factor 2, c-fos interacting	0,0059766	0,796
235832_at	**NKX6-2**	NK6 transcription factor related, locus 2 (Drosophila)	0,0059862	0,866
220998_s_at	**UNC93B1**	unc-93 homolog B1 (C. elegans)	0,0059879	0,744
241915_at	**LOC123876**	NA	0,0059955	0,827
227716_at	**UBXD5**	UBX domain containing 5	0,0060030	0,816
219776_s_at	**FLJ11125**	NA	0,0060075	0,810
229037_at	**LOC400368**	NA	0,0060299	0,867
244692_at	**FLJ39501**	NA	0,0060776	0,841
1553385_at	**FLJ14816**	NA	0,0061003	0,804
1558976_x_at	**LOC124402**	NA	0,0061210	0,853
240884_at	**CCDC14**	coiled-coil domain containing 14	0,0061258	0,879
206561_s_at	**AKR1B10**	aldo-keto reductase family 1, member B10 (aldose reductase)	0,0061497	0,553
222238_s_at	**POLM**	polymerase (DNA directed), mu	0,0061497	0,664
216671_x_at	**MUC8**	mucin 8, tracheobronchial	0,0061649	0,829
224792_at	**TNKS1BP1**	tankyrase 1 binding protein 1, 182kDa	0,0061755	0,738

238074_at	**WDR27**	WD repeat domain 27	0,0061755	0,824
238209_at	**LOC349236**	NA	0,0061962	0,848
202650_s_at	**KIAA0195**	NA	0,0062133	0,800
229930_at	**LOC150371**	NA	0,0062291	0,846
226286_at	**RBED1**	RNA binding motif and ELMO domain 1	0,0062930	0,784
1553486_a_at	**FLJ39647**	NA	0,0063060	0,893
244830_at	**RASIP1**	Ras interacting protein 1	0,0063477	0,827
231196_x_at	**C10orf51**	chromosome 10 open reading frame 51	0,0064066	0,847
222107_x_at	**LZTS1**	leucine zipper, putative tumor suppressor 1	0,0064297	0,830
211848_s_at	**CEACAM7**	carcinoembryonic antigen-related cell adhesion molecule 7	0,0064313	0,846
224686_x_at	**LOC388397**	NA	0,0064520	0,638
207757_at	**ZFP2**	zinc finger protein 2 homolog (mouse)	0,0064759	0,768
1556187_at	**ZNF555**	zinc finger protein 555	0,0064828	0,820
228741_s_at	**HCN3**	hyperpolarization activated cyclic nucleotide-gated potassium channel 3	0,0065061	0,873
237284_at	**DNAJB8**	DnaJ (Hsp40) homolog, subfamily B, member 8	0,0065285	0,830
209420_s_at	**SMPD1**	sphingomyelin phosphodiesterase 1, acid lysosomal (acid sphingomyelinase)	0,0065742	0,757
203448_s_at	**TERF1**	telomeric repeat binding factor (NIMA-interacting) 1	0,0065775	0,765
218410_s_at	**MGC4692**	NA	0,0066177	0,850
225272_at	**SAT2**	spermidine/spermine N1-acetyltransferase 2	0,0066286	0,640
221655_x_at	**EPS8L1**	EPS8-like 1	0,0066458	0,810
222986_s_at	**SCOTIN**	NA	0,0066769	0,764
221254_s_at	**PITPNM3**	PITPNM family member 3	0,0066999	0,826
209768_s_at	**GP1BB**	glycoprotein Ib (platelet), beta polypeptide	0,0067852	0,779
231667_at	**SLC39A5**	solute carrier family 39 (metal ion transporter), member 5	0,0067968	0,791
218745_x_at	**FLJ20422**	NA	0,0068030	0,842
224372_at	**PCBD2**	6-pyruvoyl-tetrahydropterin synthase/dimerization cofactor of hepatocyte nuclear factor 1 alpha (TCF1) 2	0,0068030	0,886
213185_at	**KIAA0556**	NA	0,0068874	0,832
207562_at	**DGKQ**	diacylglycerol kinase, theta 110kDa	0,0069869	0,826
205276_s_at	**GTPBP1**	GTP binding protein 1	0,0070222	0,900
1568867_x_at	**KIAA0553**	NA	0,0070222	0,922
236245_at	**ODF3L1**	outer dense fiber of sperm tails 3-like 1	0,0070496	0,840
202592_at	**BLOC1S1**	biogenesis of lysosome-related organelles complex-1, subunit 1	0,0070502	0,775
1564690_at	**C9orf41**	chromosome 9 open reading frame 41	0,0070552	0,915
215812_s_at	**FLJ43855**	NA	0,0070552	0,750
221298_s_at	**SLC22A8**	solute carrier family 22 (organic anion transporter), member 8	0,0070952	0,810
209441_at	**RHOBTB2**	Rho-related BTB domain containing 2	0,0071158	0,794
220289_s_at	**AIM1L**	absent in melanoma 1-like	0,0071290	0,872
208435_s_at	**AQP6**	aquaporin 6, kidney specific	0,0071290	0,852
210977_s_at	**HSF4**	heat shock transcription factor 4	0,0071518	0,824
206746_at	**BFSP1**	beaded filament structural protein 1, filensin	0,0071608	0,670
1553823_a_at	**RTP1**	NA	0,0071841	0,890
214412_at	**H2AFB3**	H2A histone family, member B3	0,0072520	0,883
228755_at	**PERQ1**	PERQ amino acid rich, with GYF domain 1	0,0072801	0,843
231242_at	**BHLHB3**	basic helix-loop-helix domain containing, class B, 3	0,0072859	0,818
232185_at	**C20orf132**	chromosome 20 open reading frame 132	0,0072959	0,838
206054_at	**KNG1**	kininogen 1	0,0073273	0,811
223179_at	**YPEL3**	yippee-like 3 (Drosophila)	0,0073766	0,578
218555_at	**ANAPC2**	anaphase promoting complex subunit 2	0,0073827	0,823
220804_s_at	**TP73**	tumor protein p73	0,0073888	0,805
209925_at	**OCLN**	occludin	0,0074256	0,707
1558077_s_at	**MDH1B**	malate dehydrogenase 1B, NAD (soluble)	0,0074298	0,802
223967_at	**ANGPTL6**	angiopoietin-like 6	0,0074887	0,833

202689_at	**RBM15B**	RNA binding motif protein 15B	0,0074908	0,739
206013_s_at	**ACTL6B**	actin-like 6B	0,0075158	0,859
224907_s_at	**SH3GLB2**	SH3-domain GRB2-like endophilin B2	0,0075478	0,770
1554880_at	**DKFZP434K028**	NA	0,0075753	0,877
229862_x_at	**ZNF499**	zinc finger protein 499	0,0076353	0,853
221259_s_at	**TEX11**	testis expressed sequence 11	0,0076786	0,880
222445_at	**SLC39A9**	solute carrier family 39 (zinc transporter), member 9	0,0077022	0,818
224706_at	**KIAA2013**	KIAA2013	0,0077831	0,821
216059_at	**PAX3**	paired box gene 3 (Waardenburg syndrome 1)	0,0077831	0,818
1552389_at	**C8orf47**	chromosome 8 open reading frame 47	0,0078103	0,860
226674_at	**TMEM58**	transmembrane protein 58	0,0078132	0,757
232391_at	**GIOT-1**	NA	0,0078245	0,816
231246_at	**CHST10**	carbohydrate sulfotransferase 10	0,0078289	0,866
218942_at	**PIP5K2C**	phosphatidylinositol-4-phosphate 5-kinase, type II, gamma	0,0078516	0,830
226307_at	**TORC2**	NA	0,0078808	0,824
221028_s_at	**MGC11335**	NA	0,0079005	0,773
238242_at	**SBNO1**	sno, strawberry notch homolog 1 (Drosophila)	0,0079438	0,766
1560475_at	**LOC401020**	NA	0,0079810	0,841
207077_at	**ELA2B**	NA	0,0079970	0,842
1557782_s_at	**C21orf49**	chromosome 21 open reading frame 49	0,0080641	0,846
219717_at	**FLJ20280**	NA	0,0080815	0,679
211486_s_at	**KCNQ2**	potassium voltage-gated channel, KQT-like subfamily, member 2	0,0080815	0,816
222144_at	**KIF17**	kinesin family member 17	0,0080815	0,866
234414_at	**FAM22F**	family with sequence similarity 22, member F	0,0081005	0,858
225247_at	**C19orf6**	chromosome 19 open reading frame 6	0,0081129	0,703
206428_s_at	**ZNF143**	zinc finger protein 143 (clone pHZ-1)	0,0081621	0,824
233167_at	**SELO**	NA	0,0081645	0,586
228126_x_at	**CTXN1**	cortexin 1	0,0081686	0,721
241172_at	**GRIA2**	glutamate receptor, ionotropic, AMPA 2	0,0081777	0,914
212571_at	**CHD8**	chromodomain helicase DNA binding protein 8	0,0082072	0,860
220217_x_at	**SPANXC**	SPANX family, member C	0,0082933	0,843
52741_at	**C14orf172**	chromosome 14 open reading frame 172	0,0082989	0,861
233887_at	**GPR126**	G protein-coupled receptor 126	0,0082989	0,833
222217_s_at	**SLC27A3**	solute carrier family 27 (fatty acid transporter), member 3	0,0082989	0,594
223226_x_at	**SSBP4**	single stranded DNA binding protein 4	0,0083273	0,804
220579_at	**FLJ14100**	NA	0,0083389	0,878
244209_at	**FLJ41350**	NA	0,0083995	0,864
205720_at	**POMC**	proopiomelanocortin (adrenocorticotropin/ beta-lipotropin/ alpha-melanocyte stimulating hormone/ beta-melanocyte stimulating hormone/ beta-endorphin)	0,0084599	0,787
238571_at	**HOXA13**	homeo box A13	0,0084725	0,904
220588_at	**BCAS4**	breast carcinoma amplified sequence 4	0,0085136	0,787
227672_at	**LOC286073**	NA	0,0085136	0,848
227694_at	**LOC90529**	NA	0,0085136	0,753
225196_s_at	**MRPS26**	mitochondrial ribosomal protein S26	0,0085136	0,859
201404_x_at	**PSMB2**	proteasome (prosome, macropain) subunit, beta type, 2	0,0085316	0,773
202492_at	**ATG9A**	ATG9 autophagy related 9 homolog A (S. cerevisiae)	0,0085470	0,707
1559916_a_at	**CHST12**	carbohydrate (chondroitin 4) sulfotransferase 12	0,0085554	0,813
1562938_at	**LOC150763**	NA	0,0085554	0,824
205980_s_at	**PRR5**	NA	0,0085759	0,859
211510_s_at	**CRHR2**	corticotropin releasing hormone receptor 2	0,0086667	0,865
243258_at	**KIAA0664**	NA	0,0086939	0,815
206136_at	**FZD5**	frizzled homolog 5 (Drosophila)	0,0087234	0,750
226852_at	**MTA3**	metastasis associated 1 family, member 3	0,0087234	0,763

232019_at	**ZNF694**	zinc finger protein 694	0,0087234	0,885
201885_s_at	**CYB5R3**	cytochrome b5 reductase 3	0,0087781	0,890
223459_s_at	**C1orf56**	chromosome 1 open reading frame 56	0,0088153	0,628
1552564_at	**NUDT9P1**	nudix (nucleoside diphosphate linked moiety X)-type motif 9 pseudogene 1	0,0088295	0,814
238027_at	**LOC202051**	NA	0,0088348	0,745
220894_x_at	**PRDM12**	PR domain containing 12	0,0088433	0,816
205458_at	**MC1R**	melanocortin 1 receptor (alpha melanocyte stimulating hormone receptor)	0,0088623	0,860
214892_x_at	**C19orf29**	chromosome 19 open reading frame 29	0,0089870	0,858
1570329_at	**ZNF395**	zinc finger protein 395	0,0089942	0,887
218346_s_at	**SESN1**	sestrin 1	0,0090677	0,648
207194_s_at	**ICAM4**	intercellular adhesion molecule 4, Landsteiner-Wiener blood group	0,0091542	0,866
205670_at	**GAL3ST1**	galactose-3-O-sulfotransferase 1	0,0091656	0,878
227392_at	**NISCH**	nischarin	0,0091837	0,842
217669_s_at	**AKAP6**	A kinase (PRKA) anchor protein 6	0,0092280	0,881
224659_at	**SEPN1**	selenoprotein N, 1	0,0092280	0,714
243770_at	**ZNF483**	zinc finger protein 483	0,0092455	0,853
237563_s_at	**LOC440731**	NA	0,0092881	0,786
219726_at	**NLGN3**	neuroligin 3	0,0092881	0,817
219680_at	**NOD9**	NA	0,0093404	0,833
232416_at	**BRUNOL5**	bruno-like 5, RNA binding protein (Drosophila)	0,0093608	0,918
219533_at	**CDKN1C**	cyclin-dependent kinase inhibitor 1C (p57, Kip2)	0,0093779	0,866
220371_s_at	**SLC12A9**	solute carrier family 12 (potassium/chloride transporters), member 9	0,0094698	0,835
216193_at	**LOC440366**	NA	0,0095106	0,912
208142_at	**FAM12A**	family with sequence similarity 12, member A	0,0095137	0,855
216167_at	**LRRN5**	leucine rich repeat neuronal 5	0,0095137	0,858
205056_s_at	**GPR162**	G protein-coupled receptor 162	0,0095315	0,860
235063_at	**FLJ25067**	NA	0,0095844	0,875
234695_x_at	**FAM22B**	family with sequence similarity 22, member B	0,0096300	0,825
227411_at	**WTIP**	Wilms tumor 1 interacting protein	0,0096312	0,787
212892_at	**ZNF282**	zinc finger protein 282	0,0096769	0,750
225145_at	**NCOA5**	nuclear receptor coactivator 5	0,0096994	0,818
242505_at	**LOC440962**	NA	0,0097403	0,839
239636_at	**MCF2L**	MCF.2 cell line derived transforming sequence-like	0,0097403	0,892
225473_at	**C20orf117**	chromosome 20 open reading frame 117	0,0097686	0,728
206072_at	**UCN**	urocortin	0,0097721	0,705
208890_s_at	**PLXNB2**	plexin B2	0,0097846	0,812
212437_at	**CENPB**	centromere protein B, 80kDa	0,0097881	0,674
209148_at	**RXRB**	retinoid X receptor, beta	0,0097913	0,862
219665_at	**NUDT18**	nudix (nucleoside diphosphate linked moiety X)-type motif 18	0,0097916	0,772
241426_at	**KIAA1712**	KIAA1712	0,0097998	0,930
1557262_at	**AP1G2**	adaptor-related protein complex 1, gamma 2 subunit	0,0098022	0,857
225317_at	**ACBD6**	acyl-Coenzyme A binding domain containing 6	0,0098819	0,850
1555074_a_at	**KCNH5**	potassium voltage-gated channel, subfamily H (eag-related), member 5	0,0098819	0,899
212728_at	**DLG3**	discs, large homolog 3 (neuroendocrine-dlg, Drosophila)	0,0099128	0,785
206998_x_at	**PRB3**	proline-rich protein BstNI subfamily 3	0,0099333	0,870
213674_x_at	**IGHD**	immunoglobulin heavy constant delta	0,0099350	0,880
226597_at	**C19orf32**	chromosome 19 open reading frame 32	0,0099561	0,768
226071_at	**TSRC1**	thrombospondin repeat containing 1	0,0099561	0,308
202590_s_at	**PDK2**	pyruvate dehydrogenase kinase, isoenzyme 2	0,0099626	0,632
241886_x_at	**LOC440115**	NA	0,0099975	0,887
226121_at	**MGC23280**	NA	0,0099975	0,773
235953_at	**ZNF610**	zinc finger protein 610	0,0099975	0,631

210158_at	**ERCC4**	excision repair cross-complementing rodent repair deficiency, complementation group 4	0,0100128	0,781
236482_at	**EVER2**	epidermodysplasia verruciformis 2	0,0100348	0,710
211979_at	**GPR107**	G protein-coupled receptor 107	0,0100439	0,848
204874_x_at	**BAIAP3**	BAI1-associated protein 3	0,0100453	0,857
211728_s_at	**HYAL3**	hyaluronoglucosaminidase 3	0,0100633	0,793
233586_s_at	**KLK12**	kallikrein 12	0,0100880	0,897
240311_at	**NANOS3**	nanos homolog 3 (Drosophila)	0,0100896	0,833
217177_s_at	**PTPRB**	protein tyrosine phosphatase, receptor type, B	0,0101219	0,864
205989_s_at	**MOG**	myelin oligodendrocyte glycoprotein	0,0101527	0,824
205824_at	**HSPB2**	heat shock 27kDa protein 2	0,0102271	0,694
1553618_at	**TRIM43**	tripartite motif-containing 43	0,0103029	0,932
238196_at	**LOC285095**	NA	0,0103078	0,894
1570364_at	**FLJ38281**	NA	0,0103462	0,808
214751_at	**ZNF468**	NA	0,0103463	0,719
229922_at	**KIAA1893**	NA	0,0103614	0,842
238400_at	**FLJ35220**	NA	0,0103659	0,873
215840_at	**FLJ46675**	NA	0,0103719	0,899
220674_at	**FLJ22814**	NA	0,0104599	0,859
211753_s_at	**RLN1**	relaxin 1	0,0104744	0,873
1561025_at	**CDKAL1**	CDK5 regulatory subunit associated protein 1-like 1	0,0105771	0,873
218660_at	**DYSF**	dysferlin, limb girdle muscular dystrophy 2B (autosomal recessive)	0,0105970	0,544
208110_x_at	**MED25**	mediator of RNA polymerase II transcription, subunit 25 homolog (yeast)	0,0105970	0,835
214643_x_at	**BIN1**	bridging integrator 1	0,0106551	0,836
217969_at	**C11orf2**	chromosome 11 open reading frame2	0,0106565	0,759
235510_at	**USHBP1**	Usher syndrome 1C binding protein 1	0,0106705	0,718
239057_at	**LMOD2**	leiomodin 2 (cardiac)	0,0107210	0,850
204706_at	**INPP5E**	inositol polyphosphate-5-phosphatase, 72 kDa	0,0107742	0,799
236709_at	**LOC400411**	NA	0,0107935	0,806
240792_at	**NCOR2**	nuclear receptor co-repressor 2	0,0108116	0,882
214017_s_at	**DHX34**	DEAH (Asp-Glu-Ala-His) box polypeptide 34	0,0108185	0,851
207019_s_at	**AKAP4**	A kinase (PRKA) anchor protein 4	0,0108962	0,864
207163_s_at	**AKT1**	v-akt murine thymoma viral oncogene homolog 1	0,0109442	0,749
1559788_at	**C20orf142**	chromosome 20 open reading frame 142	0,0109483	0,880
237236_x_at	**LOC387643**	NA	0,0109536	0,855
220753_s_at	**CRYL1**	crystallin, lambda 1	0,0110932	0,825
221566_s_at	**NOL3**	nucleolar protein 3 (apoptosis repressor with CARD domain)	0,0110932	0,753
229884_s_at	**MRPL2**	mitochondrial ribosomal protein L2	0,0111209	0,725
217961_at	**FLJ20551**	NA	0,0111363	0,825
224059_s_at	**NUMBL**	numb homolog (Drosophila)-like	0,0111603	0,848
220116_at	**KCNN2**	potassium intermediate/small conductance calcium-activated channel, subfamily N, member 2	0,0111947	0,854
230164_at	**ZNF621**	zinc finger protein 621	0,0112092	0,730
222125_s_at	**PH-4**	NA	0,0112156	0,761
212431_at	**KIAA0194**	NA	0,0112390	0,845
232066_x_at	**MGC21675**	NA	0,0112468	0,823
200696_s_at	**GSN**	gelsolin (amyloidosis, Finnish type)	0,0112699	0,685
242386_x_at	**LOC283387**	NA	0,0112932	0,873
202571_s_at	**DLGAP4**	discs, large (Drosophila) homolog-associated protein 4	0,0113360	0,735
242520_s_at	**MGC33556**	NA	0,0113371	0,863
205007_s_at	**CIB2**	calcium and integrin binding family member 2	0,0113969	0,845
200601_at	**ACTN4**	actinin, alpha 4	0,0114343	0,726
213380_x_at	**MSTP9**	macrophage stimulating, pseudogene 9	0,0114590	0,817
205738_s_at	**FABP3**	fatty acid binding protein 3, muscle and heart (mammary-derived growth inhibitor)	0,0114715	0,618

227544_at	**C14orf83**	chromosome 14 open reading frame 83	0,0114988	0,802
220921_at	**SPANXA1**	sperm protein associated with the nucleus, X-linked, family member A1	0,0115116	0,861
226842_at	**LOC90110**	NA	0,0115551	0,820
57715_at	**FAM26B**	family with sequence similarity 26, member B	0,0115975	0,732
217681_at	**WNT7B**	wingless-type MMTV integration site family, member 7B	0,0116584	0,863
1552496_a_at	**COBL**	cordon-bleu homolog (mouse)	0,0116689	0,859
205614_x_at	**MST1**	macrophage stimulating 1 (hepatocyte growth factor-like)	0,0116689	0,847
234895_at	**CTLA4**	cytotoxic T-lymphocyte-associated protein 4	0,0117892	0,888
1555282_a_at	**PPARGC1B**	peroxisome proliferative activated receptor, gamma, coactivator 1, beta	0,0118424	0,808
231487_at	**COX8C**	cytochrome c oxidase subunit 8C	0,0118494	0,882
214893_x_at	**HCN2**	hyperpolarization activated cyclic nucleotide-gated potassium channel 2	0,0119348	0,841
1562317_at	**LOC121006**	NA	0,0119473	0,881
208700_s_at	**TKT**	transketolase (Wernicke-Korsakoff syndrome)	0,0119496	0,694
208224_at	**HOXB1**	homeo box B1	0,0119778	0,864
216330_s_at	**POU6F1**	POU domain, class 6, transcription factor 1	0,0119968	0,818
225808_at	**LOC124512**	NA	0,0120187	0,829
225475_at	**MIER1**	mesoderm induction early response 1 homolog (Xenopus laevis)	0,0120461	0,736
233840_at	**LOC284017**	NA	0,0120741	0,853
213609_s_at	**SEZ6L**	seizure related 6 homolog (mouse)-like	0,0121137	0,899
241919_x_at	**WDR31**	WD repeat domain 31	0,0122413	0,898
216654_at	**TNXB**	tenascin XB	0,0123043	0,866
238275_at	**HAP1**	huntingtin-associated protein 1 (neuroan 1)	0,0123745	0,866
1552566_at	**C10orf87**	chromosome 10 open reading frame 87	0,0123930	0,872
208081_s_at	**ZNF442**	zinc finger protein 442	0,0123930	0,836
206256_at	**CPN1**	carboxypeptidase N, polypeptide 1, 50kD	0,0124139	0,864
1566571_at	**DKFZp761N1114**	NA	0,0124625	0,893
223389_s_at	**ZNF581**	zinc finger protein 581	0,0124769	0,684
1556473_at	**FLJ38379**	NA	0,0125036	0,916
212501_at	**CEBPB**	CCAAT/enhancer binding protein (C/EBP), beta	0,0125248	0,757
232040_at	**LOC157860**	NA	0,0125256	0,853
204974_at	**RAB3A**	RAB3A, member RAS oncogene family	0,0125277	0,744
227539_at	**GNA13**	guanine nucleotide binding protein (G protein), alpha 13	0,0125531	0,725
223755_at	**KIRREL2**	kin of IRRE like 2 (Drosophila)	0,0125607	0,864
238182_at	**RFC5**	replication factor C (activator 1) 5, 36.5kDa	0,0125800	0,785
205233_s_at	**PAFAH2**	platelet-activating factor acetylhydrolase 2, 40kDa	0,0126120	0,831
221348_at	**NPPC**	natriuretic peptide precursor C	0,0126165	0,897
223753_s_at	**CFC1**	cripto, FRL-1, cryptic family 1	0,0126939	0,856
208472_at	**ZNFN1A4**	zinc finger protein, subfamily 1A, 4 (Eos)	0,0127040	0,885
209352_s_at	**SIN3B**	SIN3 homolog B, transcription regulator (yeast)	0,0127887	0,774
234742_at	**SIRPB2**	signal-regulatory protein beta 2	0,0127894	0,863
225628_s_at	**MLLT6**	c("myeloid/lymphoid or mixed-lineage leukemia (trithorax homolog, Drosophila)", " translocated to, 6")	0,0128010	0,858
223152_at	**PPP1R12C**	protein phosphatase 1, regulatory (inhibitor) subunit 12C	0,0128077	0,766
241358_at	**WFIKKN2**	WAP, follistatin/kazal, immunoglobulin, kunitz and netrin domain containing 2	0,0128077	0,829
232275_s_at	**HS6ST3**	heparan sulfate 6-O-sulfotransferase 3	0,0128347	0,916
233955_x_at	**CXXC5**	CXXC finger 5	0,0128623	0,795
228747_at	**SEC61A2**	Sec61 alpha 2 subunit (S. cerevisiae)	0,0128623	0,796
213611_at	**AQP5**	aquaporin 5	0,0128796	0,822
1560201_at	**FLJ39963**	NA	0,0128804	0,817
224548_at	**HES7**	hairy and enhancer of split 7 (Drosophila)	0,0128936	0,849
219488_at	**A4GALT**	alpha 1,4-galactosyltransferase (globotriaosylceramide synthase)	0,0128981	0,847
240541_at	**LOC388383**	NA	0,0129032	0,799

243206_at	**LOC90799**	NA	0,0129032	0,893
221868_at	**KIAA1155**	NA	0,0129039	0,879
1552631_a_at	**MAP3K6**	mitogen-activated protein kinase kinase kinase 6	0,0129279	0,838
206850_at	**RRP22**	NA	0,0129279	0,866
208556_at	**GPR31**	G protein-coupled receptor 31	0,0129633	0,858
213157_s_at	**KIAA0523**	NA	0,0129633	0,838
232087_at	**CXorf23**	chromosome X open reading frame 23	0,0129640	0,771
219116_s_at	**C13orf17**	chromosome 13 open reading frame 17	0,0130023	0,864
209043_at	**PAPSS1**	3'-phosphoadenosine 5'-phosphosulfate synthase 1	0,0130023	0,790
206153_at	**CYP4F11**	cytochrome P450, family 4, subfamily F, polypeptide 11	0,0131002	0,874
1559390_a_at	**LOC149643**	NA	0,0131144	0,911
212319_at	**RUTBC1**	RUN and TBC1 domain containing 1	0,0131219	0,799
235972_at	**RW1**	NA	0,0131810	0,852
211897_s_at	**CRHR1**	corticotropin releasing hormone receptor 1	0,0132007	0,841
220584_at	**FLJ22184**	NA	0,0133043	0,856
213569_at	**VASH1**	vasohibin 1	0,0133293	0,860
202391_at	**BASP1**	brain abundant, membrane attached signal protein 1	0,0133500	0,653
235574_at	**GBP4**	guanylate binding protein 4	0,0134283	0,923
208547_at	**HIST1H2BB**	histone 1, H2bb	0,0134428	0,880
238136_at	**LOC284296**	NA	0,0134898	0,859
232999_at	**FLJ35848**	NA	0,0134993	0,880
227515_at	**STAMBP**	STAM binding protein	0,0135059	0,784
235569_at	**VPS37D**	vacuolar protein sorting 37D (yeast)	0,0135089	0,798
231568_at	**LOC255313**	NA	0,0136130	0,854
233374_at	**HIC2**	hypermethylated in cancer 2	0,0136165	0,893
242697_at	**ZNF540**	zinc finger protein 540	0,0136181	0,834
221818_at	**KIAA1698**	NA	0,0136233	0,850
1561948_at	**FLJ13448**	NA	0,0136412	0,892
202175_at	**CHPF**	NA	0,0136519	0,674
1561554_at	**MYOCD**	myocardin	0,0136907	0,867
212212_s_at	**DKFZP586J0619**	NA	0,0136918	0,807
221835_at	**DTX3**	deltex 3 homolog (Drosophila)	0,0137403	0,884
234008_s_at	**FLJ21736**	NA	0,0137403	0,845
1556711_at	**FLJ40919**	NA	0,0137601	0,906
203193_at	**ESRRA**	estrogen-related receptor alpha	0,0138290	0,824
1553834_at	**FLJ35880**	NA	0,0138342	0,905
237380_at	**FBXO46**	F-box protein 46	0,0138727	0,839
227456_s_at	**C6orf136**	chromosome 6 open reading frame 136	0,0138837	0,794
230455_at	**PPP1R9B**	protein phosphatase 1, regulatory subunit 9B, spinophilin	0,0139671	0,844
215632_at	**NEUROG2**	neurogenin 2	0,0139805	0,828
210600_s_at	**GRK4**	G protein-coupled receptor kinase 4	0,0140161	0,882
234291_s_at	**SLC6A20**	solute carrier family 6 (proline IMINO transporter), member 20	0,0140418	0,870
213403_at	**MGC11332**	NA	0,0140485	0,763
205918_at	**SLC4A3**	solute carrier family 4, anion exchanger, member 3	0,0140598	0,776
236496_at	**DEGS2**	degenerative spermatocyte homolog 2, lipid desaturase (Drosophila)	0,0140707	0,846
223954_x_at	**APBA2BP**	amyloid beta (A4) precursor protein-binding, family A, member 2 binding protein	0,0141376	0,873
214477_at	**MLLT1**	c("myeloid/lymphoid or mixed-lineage leukemia (trithorax homolog, Drosophila)", " translocated to, 1")	0,0141396	0,863
204356_at	**LIMK1**	LIM domain kinase 1	0,0141412	0,881
1316_at	**THRA**	thyroid hormone receptor, alpha (erythroblastic leukemia viral (v-erb-a) oncogene homolog, avian)	0,0141428	0,874
241115_at	**KIAA1467**	NA	0,0141540	0,837
211830_s_at	**CACNA1I**	calcium channel, voltage-dependent, alpha 1I subunit	0,0142881	0,865
207907_at	**TNFSF14**	tumor necrosis factor (ligand) superfamily, member 14	0,0143027	0,814

224539_s_at	**PCDHAC2**	protocadherin alpha subfamily C, 2	0,0143196	0,827
1560316_s_at	**GLCCI1**	glucocorticoid induced transcript 1	0,0143262	0,681
31826_at	**KIAA0674**	KIAA0674	0,0143265	0,905
238594_x_at	**DUSP8**	dual specificity phosphatase 8	0,0144330	0,837
222896_at	**TMEM38A**	transmembrane protein 38A	0,0144330	0,787
204343_at	**ABCA3**	ATP-binding cassette, sub-family A (ABC1), member 3	0,0144355	0,726
213181_s_at	**MOCS1**	molybdenum cofactor synthesis 1	0,0144355	0,632
213052_at	**PRKAR2A**	protein kinase, cAMP-dependent, regulatory, type II, alpha	0,0144535	0,832
229209_at	**LOC283596**	NA	0,0145822	0,907
223820_at	**RBP5**	retinol binding protein 5, cellular	0,0145937	0,846
217622_at	**C22orf3**	chromosome 22 open reading frame 3	0,0145964	0,856
241213_x_at	**PTPRN2**	protein tyrosine phosphatase, receptor type, N polypeptide 2	0,0145964	0,774
244489_at	**THBS3**	thrombospondin 3	0,0145964	0,834
221317_x_at	**PCDHB6**	protocadherin beta 6	0,0146039	0,776
229162_s_at	**ABTB1**	ankyrin repeat and BTB (POZ) domain containing 1	0,0146825	0,846
229113_s_at	**C1orf86**	chromosome 1 open reading frame 86	0,0147483	0,784
204941_s_at	**ALDH3B2**	aldehyde dehydrogenase 3 family, member B2	0,0148448	0,876
202645_s_at	**MEN1**	multiple endocrine neoplasia I	0,0148522	0,795
243821_at	**MRPS31**	mitochondrial ribosomal protein S31	0,0148709	0,905
210958_s_at	**MAST4**	microtubule associated serine/threonine kinase family member 4	0,0148856	0,684
202310_s_at	**COL1A1**	collagen, type I, alpha 1	0,0148986	0,879
240348_at	**SLC6A11**	solute carrier family 6 (neurotransmitter transporter, GABA), member 11	0,0149226	0,887
208258_s_at	**GAS2L1**	growth arrest-specific 2 like 1	0,0149689	0,715
202210_x_at	**GSK3A**	glycogen synthase kinase 3 alpha	0,0150102	0,862
200859_x_at	**FLNA**	filamin A, alpha (actin binding protein 280)	0,0150243	0,786
206846_s_at	**HDAC6**	histone deacetylase 6	0,0151528	0,797
225500_x_at	**SR-A1**	NA	0,0151528	0,786
207339_s_at	**LTB**	lymphotoxin beta (TNF superfamily, member 3)	0,0151847	0,840
206531_at	**DPF1**	D4, zinc and double PHD fingers family 1	0,0152006	0,806
235055_x_at	**MUC4**	mucin 4, tracheobronchial	0,0152013	0,823
240522_at	**BAIAP2L1**	BAI1-associated protein 2-like 1	0,0152232	0,875
227848_at	**PEBP4**	NA	0,0152271	0,733
227407_at	**FLJ90013**	NA	0,0152352	0,852
242988_at	**DCST2**	DC-STAMP domain containing 2	0,0152526	0,919
217661_x_at	**SIX5**	sine oculis homeobox homolog 5 (Drosophila)	0,0153800	0,862
218648_at	**TORC3**	NA	0,0154065	0,723
206709_x_at	**GPT**	glutamic-pyruvate transaminase (alanine aminotransferase)	0,0154190	0,909
205377_s_at	**ACHE**	acetylcholinesterase (YT blood group)	0,0154280	0,801
211142_x_at	**HLA-DOA**	major histocompatibility complex, class II, DO alpha	0,0154509	0,917
1567358_at	**NAV2**	neuron navigator 2	0,0155533	0,881
227825_at	**C9orf90**	chromosome 9 open reading frame 90	0,0155782	0,863
224073_at	**FLJ20464**	NA	0,0155782	0,829
219579_at	**RAB3IL1**	RAB3A interacting protein (rabin3)-like 1	0,0155941	0,767
214142_at	**ZG16**	NA	0,0156142	0,834
214740_at	**POLR2J2**	NA	0,0156486	0,876
230378_at	**SCGB3A1**	secretoglobin, family 3A, member 1	0,0157368	0,778
1562698_x_at	**LOC339988**	NA	0,0157401	0,878
241467_at	**OSBPL5**	oxysterol binding protein-like 5	0,0157790	0,850
221772_s_at	**PPP2R2D**	protein phosphatase 2, regulatory subunit B, delta isoform	0,0157790	0,905
202913_at	**ARHGEF11**	Rho guanine nucleotide exchange factor (GEF) 11	0,0157902	0,740
1566242_at	**CUTL1**	cut-like 1, CCAAT displacement protein (Drosophila)	0,0157926	0,890
218742_at	**NARFL**	nuclear prelamin A recognition factor-like	0,0157926	0,895
237004_at	**WDTC2**	WD and tetratricopeptide repeats 2	0,0157926	0,882

226168_at	**ZFAND2B**	zinc finger, AN1-type domain 2B	0,0157926	0,857
220412_x_at	**KCNK7**	potassium channel, subfamily K, member 7	0,0158156	0,897
227023_at	**GLI4**	GLI-Kruppel family member GLI4	0,0158408	0,833
224886_at	**STUB1**	STIP1 homology and U-box containing protein 1	0,0158725	0,838
228668_x_at	**FLJ36031**	NA	0,0159239	0,861
218848_at	**WDR58**	WD repeat domain 58	0,0159414	0,699
220545_s_at	**TSKS**	NA	0,0159458	0,897
209959_at	**NR4A3**	nuclear receptor subfamily 4, group A, member 3	0,0161347	0,484
227281_at	**SLC29A4**	solute carrier family 29 (nucleoside transporters), member 4	0,0161347	0,806
231072_at	**MIDN**	midnolin	0,0161377	0,887
220531_at	**FLJ14126**	NA	0,0161484	0,867
236275_at	**KIAA1862**	NA	0,0162155	0,869
212439_at	**RNF123**	ring finger protein 123	0,0162614	0,918
205760_s_at	**OGG1**	8-oxoguanine DNA glycosylase	0,0163183	0,669
205611_at	**TNFSF12**	tumor necrosis factor (ligand) superfamily, member 12	0,0163183	0,759
1555198_x_at	**C21orf58**	chromosome 21 open reading frame 58	0,0163544	0,845
211877_s_at	**PCDHGA11**	protocadherin gamma subfamily A, 11	0,0164475	0,904
218714_at	**MGC3121**	NA	0,0164939	0,848
38269_at	**PRKD2**	protein kinase D2	0,0164939	0,814
209202_s_at	**EXTL3**	exostoses (multiple)-like 3	0,0165010	0,820
203254_s_at	**TLN1**	talin 1	0,0165121	0,572
202588_at	**AK1**	adenylate kinase 1	0,0165161	0,828
244240_at	**LOC440112**	NA	0,0165255	0,857
240626_at	**C8orf15**	chromosome 8 open reading frame 15	0,0165400	0,897
235243_at	**CLIPR-59**	NA	0,0165556	0,666
228671_at	**RP13-15M17.2**	NA	0,0165702	0,803
239076_at	**LOC441220**	NA	0,0165922	0,842
204432_at	**SOX12**	SRY (sex determining region Y)-box 12	0,0166427	0,683
211780_x_at	**DCTN1**	dynactin 1 (p150, glued homolog, Drosophila)	0,0166681	0,737
218412_s_at	**GTF2IRD1**	GTF2I repeat domain containing 1	0,0166681	0,739
240841_at	**INSM2**	insulinoma-associated 2	0,0166681	0,848
240069_at	**LOC441335**	NA	0,0166681	0,723
218208_at	**PQLC1**	PQ loop repeat containing 1	0,0166681	0,791
208505_s_at	**FUT2**	fucosyltransferase 2 (secretor status included)	0,0166721	0,882
219900_s_at	**ZNF446**	zinc finger protein 446	0,0166812	0,894
213218_at	**ZNF187**	zinc finger protein 187	0,0167866	0,770
239793_at	**XRCC3**	X-ray repair complementing defective repair in Chinese hamster cells 3	0,0167913	0,787
227248_at	**FLJ21019**	NA	0,0167915	0,837
225335_at	**ZNF496**	zinc finger protein 496	0,0168064	0,760
232545_at	**LRRC29**	leucine rich repeat containing 29	0,0169404	0,848
208479_at	**KCNA1**	potassium voltage-gated channel, shaker-related subfamily, member 1 (episodic ataxia with myokymia)	0,0170421	0,859
229986_at	**LOC377064**	NA	0,0170496	0,779
207292_s_at	**MAPK7**	mitogen-activated protein kinase 7	0,0170596	0,846
1560108_at	**NYREN18**	NA	0,0170830	0,896
204919_at	**PRR4**	proline rich 4 (lacrimal)	0,0170830	0,826
227323_at	**COX4NB**	COX4 neighbor	0,0171242	0,859
208277_at	**PITX3**	paired-like homeodomain transcription factor 3	0,0171532	0,811
1570244_at	**KIAA1409**	KIAA1409	0,0171571	0,924
221274_s_at	**LMAN2L**	lectin, mannose-binding 2-like	0,0171692	0,805
214625_s_at	**MINK1**	misshapen-like kinase 1 (zebrafish)	0,0171707	0,748
223353_at	**HCCA2**	NA	0,0171966	0,829
234370_at	**VSIG1**	V-set and immunoglobulin domain containing 1	0,0171966	0,884
228470_at	**RNF187**	ring finger protein 187	0,0172180	0,833

206262_at	**ADH1C**	alcohol dehydrogenase 1C (class I), gamma polypeptide	0,0172274	0,857
243978_at	**C20orf175**	chromosome 20 open reading frame 175	0,0172274	0,874
205707_at	**IL17R**	interleukin 17 receptor	0,0172688	0,812
204436_at	**pp9099**	NA	0,0172860	0,733
1563074_at	**LOC255654**	NA	0,0172968	0,861
206334_at	**LIPF**	lipase, gastric	0,0173084	0,874
217658_at	**THAP3**	THAP domain containing, apoptosis associated protein 3	0,0173291	0,876
222154_s_at	**DNAPTP6**	NA	0,0173368	0,790
210037_s_at	**NOS2A**	nitric oxide synthase 2A (inducible, hepatocytes)	0,0173442	0,889
213473_at	**BRAP**	BRCA1 associated protein	0,0173871	0,793
220280_s_at	**ANKMY1**	ankyrin repeat and MYND domain containing 1	0,0174352	0,877
228888_at	**STAC2**	SH3 and cysteine rich domain 2	0,0174352	0,812
233524_at	**C9orf44**	chromosome 9 open reading frame 44	0,0174388	0,788
206759_at	**FCER2**	Fc fragment of IgE, low affinity II, receptor for (CD23A)	0,0174388	0,793
202473_x_at	**HCFC1**	host cell factor C1 (VP16-accessory protein)	0,0174388	0,815
1562283_at	**LOC440806**	NA	0,0174388	0,889
228226_s_at	**MGC33584**	NA	0,0174388	0,816
241147_at	**SEC15L1**	SEC15-like 1 (S. cerevisiae)	0,0174401	0,906
231002_s_at	**NUP88**	nucleoporin 88kDa	0,0174468	0,797
244861_at	**ZNF527**	zinc finger protein 527	0,0174468	0,849
219968_at	**ZNF589**	zinc finger protein 589	0,0174468	0,819
244109_at	**LOC401551**	NA	0,0174500	0,879
221299_at	**GPR173**	G-protein coupled receptor 173	0,0174963	0,860
214767_s_at	**HSPB6**	heat shock protein, alpha-crystallin-related, B6	0,0174963	0,257
1556156_at	**ESRRB**	estrogen-related receptor beta	0,0175002	0,882
212699_at	**SCAMP5**	secretory carrier membrane protein 5	0,0175274	0,835
233157_x_at	**FLJ32926**	NA	0,0175881	0,812
243346_at	**LMOD3**	leiomodin 3 (fetal)	0,0175881	0,871
228609_at	**LOC440897**	NA	0,0175881	0,875
211175_at	**GPR45**	G protein-coupled receptor 45	0,0176219	0,891
237450_at	**LOC389332**	NA	0,0176219	0,860
230648_at	**LOC283663**	NA	0,0176526	0,828
243700_x_at	**FAM47A**	family with sequence similarity 47, member A	0,0177143	0,900
218320_s_at	**NDUFB11**	NADH dehydrogenase (ubiquinone) 1 beta subcomplex, 11, 17.3kDa	0,0177471	0,844
217270_s_at	**DYRK1B**	dual-specificity tyrosine-(Y)-phosphorylation regulated kinase 1B	0,0177578	0,842
221240_s_at	**B3GNT4**	UDP-GlcNAc:betaGal beta-1,3-N-acetylglucosaminyltransferase 4	0,0177635	0,817
204243_at	**RLF**	rearranged L-myc fusion	0,0177635	0,790
231015_at	**KLF15**	Kruppel-like factor 15	0,0178131	0,770
211837_s_at	**PTCRA**	pre T-cell antigen receptor alpha	0,0178131	0,854
223867_at	**TEKT3**	tektin 3	0,0178244	0,814
221267_s_at	**C19orf27**	chromosome 19 open reading frame 27	0,0178502	0,737
1561462_at	**HEPHL1**	hephaestin-like 1	0,0178502	0,873
236218_at	**PHOSPHO1**	phosphatase, orphan 1	0,0179175	0,812
207123_s_at	**MATN4**	matrilin 4	0,0179208	0,863
238923_at	**SPOP**	speckle-type POZ protein	0,0179337	0,815
1555557_a_at	**TNK2**	tyrosine kinase, non-receptor, 2	0,0180010	0,864
221239_s_at	**FCRL2**	Fc receptor-like 2	0,0180152	0,874
229728_at	**LOC388291**	NA	0,0180494	0,827
1562914_a_at	**FLJ25328**	NA	0,0180746	0,872
211870_s_at	**PCDHA3**	protocadherin alpha 3	0,0180879	0,833
225370_at	**PYGO2**	pygopus homolog 2 (Drosophila)	0,0181179	0,690
230428_at	**THEM5**	thioesterase superfamily member 5	0,0181179	0,784
226578_s_at	**DUSP1**	dual specificity phosphatase 1	0,0181182	0,882

210130_s_at	**TM7SF2**	transmembrane 7 superfamily member 2	0,0181780	0,724
227389_x_at	**IRF2BP2**	interferon regulatory factor 2 binding protein 2	0,0181920	0,872
227582_at	**KARCA1**	NA	0,0181995	0,719
1569430_at	**C9orf115**	chromosome 9 open reading frame 115	0,0182642	0,855
220364_at	**FLJ11235**	NA	0,0182642	0,854
206263_at	**FMO4**	flavin containing monooxygenase 4	0,0182642	0,599
215834_x_at	**SCARB1**	scavenger receptor class B, member 1	0,0183053	0,823
211002_s_at	**TRIM29**	tripartite motif-containing 29	0,0183525	0,814
1556229_at	**TRPV1**	transient receptor potential cation channel, subfamily V, member 1	0,0183525	0,900
241346_at	**ARHGAP30**	Rho GTPase activating protein 30	0,0184008	0,779
225918_at	**LOC146346**	NA	0,0184033	0,766
1555740_a_at	**MRAP**	melanocortin 2 receptor accessory protein	0,0184048	0,915
230514_s_at	**LYZL2**	lysozyme-like 2	0,0184266	0,874
224452_s_at	**MGC12966**	NA	0,0184512	0,836
208831_x_at	**SUPT6H**	suppressor of Ty 6 homolog (S. cerevisiae)	0,0185098	0,785
243168_at	**PDZK1**	PDZ domain containing 1	0,0186029	0,889
217728_at	**S100A6**	S100 calcium binding protein A6 (calcyclin)	0,0186073	0,877
213681_at	**CYHR1**	cysteine/histidine-rich 1	0,0186171	0,884
225251_at	**RAB24**	RAB24, member RAS oncogene family	0,0186171	0,814
205605_at	**HOXD9**	homeo box D9	0,0186293	0,851
1552630_a_at	**SRCAP**	NA	0,0186862	0,774
1561720_at	**RECQL5**	RecQ protein-like 5	0,0187196	0,915
221301_at	**C6orf27**	chromosome 6 open reading frame 27	0,0187230	0,818
220573_at	**KLK14**	kallikrein 14	0,0187636	0,818
212731_at	**ANKRD46**	ankyrin repeat domain 46	0,0187881	0,759
233144_s_at	**RASAL1**	RAS protein activator like 1 (GAP1 like)	0,0187907	0,850
232986_at	**ZNF233**	zinc finger protein 233	0,0188125	0,813
240211_at	**APCDD1**	adenomatosis polyposis coli down-regulated 1	0,0188236	0,913
205002_at	**AHDC1**	AT hook, DNA binding motif, containing 1	0,0188412	0,703
218752_at	**ZMAT5**	zinc finger, matrin type 5	0,0188412	0,821
206286_s_at	**TDGF1**	teratocarcinoma-derived growth factor 1	0,0188616	0,819
219994_at	**APBB1IP**	amyloid beta (A4) precursor protein-binding, family B, member 1 interacting protein	0,0188712	0,698
210652_s_at	**C1orf34**	chromosome 1 open reading frame 34	0,0188716	0,894
244757_at	**CYP2R1**	cytochrome P450, family 2, subfamily R, polypeptide 1	0,0188785	0,923
231617_at	**MGC35206**	NA	0,0188785	0,886
221562_s_at	**SIRT3**	sirtuin (silent mating type information regulation 2 homolog) 3 (S. cerevisiae)	0,0188785	0,746
217530_at	**SLC34A1**	solute carrier family 34 (sodium phosphate), member 1	0,0188899	0,917
213965_s_at	**CHD5**	chromodomain helicase DNA binding protein 5	0,0189088	0,858
1553936_a_at	**MGC2848**	NA	0,0189120	0,873
218301_at	**RNPEPL1**	arginyl aminopeptidase (aminopeptidase B)-like 1	0,0189120	0,797
203244_at	**PEX5**	peroxisomal biogenesis factor 5	0,0189153	0,787
1557618_at	**LOC285768**	NA	0,0189237	0,897
203602_s_at	**ZBTB17**	zinc finger and BTB domain containing 17	0,0189237	0,876
202074_s_at	**OPTN**	optineurin	0,0190706	0,833
218164_at	**SPATA20**	spermatogenesis associated 20	0,0191212	0,650
210402_at	**KCNJ1**	potassium inwardly-rectifying channel, subfamily J, member 1	0,0191301	0,891
216267_s_at	**PL6**	NA	0,0191336	0,811
231474_at	**ECAT1**	NA	0,0191368	0,858
211685_s_at	**NCALD**	neurocalcin delta	0,0191439	0,711
229646_at	**FLJ10159**	NA	0,0191544	0,879
207962_at	**CAPN11**	calpain 11	0,0191561	0,870
228064_at	**LOC388886**	NA	0,0191998	0,827
224537_at	**PCDHGC5**	protocadherin gamma subfamily C, 5	0,0192519	0,839

214784_x_at	**XPO6**	exportin 6	0,0192816	0,884
215273_s_at	**TADA3L**	transcriptional adaptor 3 (NGG1 homolog, yeast)-like	0,0193138	0,827
1554657_a_at	**C20orf26**	chromosome 20 open reading frame 26	0,0193179	0,857
220166_at	**CNNM1**	cyclin M1	0,0193820	0,920
242180_at	**TSPAN16**	tetraspanin 16	0,0194107	0,864
202841_x_at	**OGFR**	opioid growth factor receptor	0,0194262	0,853
233076_at	**C10orf39**	chromosome 10 open reading frame 39	0,0194743	0,759
232741_at	**GLUD1**	glutamate dehydrogenase 1	0,0194857	0,934
1553361_x_at	**FBXL18**	F-box and leucine-rich repeat protein 18	0,0195054	0,868
208880_s_at	**C20orf14**	chromosome 20 open reading frame 14	0,0195367	0,853
236847_at	**C19orf18**	chromosome 19 open reading frame 18	0,0196507	0,835
222864_s_at	**ZNF219**	zinc finger protein 219	0,0196578	0,843
234943_at	**CSNK1E**	casein kinase 1, epsilon	0,0196713	0,881
233615_at	**CGA**	glycoprotein hormones, alpha polypeptide	0,0196913	0,877
204503_at	**EVPL**	envoplakin	0,0197317	0,875
235490_at	**MGC10744**	NA	0,0197317	0,904
227284_at	**LOC90321**	NA	0,0197769	0,808
220709_at	**ZNF556**	zinc finger protein 556	0,0197809	0,801
203600_s_at	**C4orf8**	chromosome 4 open reading frame 8	0,0197938	0,836
226171_at	**FLJ20209**	NA	0,0198076	0,746
206646_at	**GLI1**	glioma-associated oncogene homolog 1 (zinc finger protein)	0,0198125	0,862
1553663_a_at	**NPB**	NA	0,0198220	0,834
230668_at	**C20orf58**	chromosome 20 open reading frame 58	0,0198353	0,808
229764_at	**FAM79B**	family with sequence similarity 79, member B	0,0198525	0,739
232937_at	**LRCH1**	leucine-rich repeats and calponin homology (CH) domain containing 1	0,0199893	0,865
216916_s_at	**DLGAP2**	discs, large (Drosophila) homolog-associated protein 2	0,0200233	0,852
225322_s_at	**FLJ22175**	NA	0,0200233	0,786
204650_s_at	**APBB3**	amyloid beta (A4) precursor protein-binding, family B, member 3	0,0200268	0,753
201162_at	**IGFBP7**	insulin-like growth factor binding protein 7	0,0200268	0,892
244747_at	**NPNT**	NA	0,0200268	0,889
1559504_at	**FLJ35409**	NA	0,0200625	0,873
228681_x_at	**DAPK3**	death-associated protein kinase 3	0,0200758	0,880
227385_at	**PPAPDC2**	phosphatidic acid phosphatase type 2 domain containing 2	0,0201213	0,835
236721_at	**ALKBH**	alkB, alkylation repair homolog (E. coli)	0,0201233	0,832
206362_x_at	**MAP3K10**	mitogen-activated protein kinase kinase kinase 10	0,0201250	0,871
206802_at	**PAX5**	paired box gene 5 (B-cell lineage specific activator)	0,0201253	0,872
223973_at	**C19orf30**	chromosome 19 open reading frame 30	0,0201316	0,878
220827_at	**CACNA1C**	calcium channel, voltage-dependent, L type, alpha 1C subunit	0,0202146	0,868
220061_at	**FLJ20581**	NA	0,0202284	0,626
1559559_at	**C9orf79**	chromosome 9 open reading frame 79	0,0202378	0,869
227806_at	**MGC17624**	NA	0,0202637	0,744
221278_at	**HOXB8**	homeo box B8	0,0202767	0,849
1552730_at	**DHH**	desert hedgehog homolog (Drosophila)	0,0202901	0,874
206484_s_at	**XPNPEP2**		0,0203136	0,849
219070_s_at	**MOSPD3**	motile sperm domain containing 3	0,0203324	0,853
239998_at	**C10orf53**	chromosome 10 open reading frame 53	0,0203401	0,857
206393_at	**TNNI2**	troponin I, skeletal, fast	0,0204079	0,806
221585_at	**CACNG4**	calcium channel, voltage-dependent, gamma subunit 4	0,0204257	0,781
206640_x_at	**GAGE7B**	G antigen 7B	0,0204257	0,845
220561_at	**IGF2AS**	insulin-like growth factor 2 antisense	0,0204257	0,827
226522_at	**PODN**	podocan	0,0204257	0,567
239523_at	**TUSC5**	tumor suppressor candidate 5	0,0204257	0,816
232328_at	**ZNF552**	zinc finger protein 552	0,0204257	0,773

215022_x_at	**ZNF11B**	zinc finger protein 11B	0,0204560	0,741
237603_at	**C1orf100**	chromosome 1 open reading frame 100	0,0204627	0,855
208455_at	**PVRL1**	c("poliovirus receptor-related 1 (herpesvirus entry mediator C", " nectin)")	0,0205966	0,889
242169_at	**BHMT2**	betaine-homocysteine methyltransferase 2	0,0206042	0,893
1561266_at	**FLJ34208**	NA	0,0206355	0,892
206518_s_at	**RGS9**	regulator of G-protein signalling 9	0,0206635	0,837
223635_s_at	**SSBP3**	single stranded DNA binding protein 3	0,0206738	0,673
35179_at	**B3GAT3**	beta-1,3-glucuronyltransferase 3 (glucuronosyltransferase I)	0,0207061	0,768
212682_s_at	**BC002942**	NA	0,0207455	0,780
236039_at	**LYPD5**	LY6/PLAUR domain containing 5	0,0207523	0,881
243116_at	**PIP5KL1**	phosphatidylinositol-4-phosphate 5-kinase-like 1	0,0207523	0,773
1552321_a_at	**NYD-SP28**	NA	0,0209154	0,911
227192_at	**LOC112476**	NA	0,0209894	0,800
235897_at	**COPZ2**	coatomer protein complex, subunit zeta 2	0,0210103	0,841
33768_at	**DMWD**	dystrophia myotonica-containing WD repeat motif	0,0210412	0,890
227298_at	**TRAM2**	translocation associated membrane protein 2	0,0210576	0,742
210810_s_at	**SLC6A5**	solute carrier family 6 (neurotransmitter transporter, glycine), member 5	0,0210759	0,894
1564373_a_at	**LOC283887**	NA	0,0211258	0,909
237801_at	**LOC400951**	NA	0,0211349	0,858
220501_at	**ACTL7A**	actin-like 7A	0,0211377	0,860
1556872_s_at	**IQSEC3**	IQ motif and Sec7 domain 3	0,0211377	0,834
229320_at	**MGC42367**	NA	0,0211423	0,748
207801_s_at	**RNF10**	ring finger protein 10	0,0211640	0,769
221790_s_at	**LDLRAP1**	low density lipoprotein receptor adaptor protein 1	0,0211667	0,759
1560834_a_at	**NCRMS**	NA	0,0212164	0,898
236112_at	**LOC285548**	NA	0,0213468	0,914
210030_at	**HLRC1**	HEAT-like (PBS lyase) repeat containing 1	0,0213925	0,879
213752_at	**FLJ43806**	NA	0,0214631	0,797
210560_at	**GBX2**	gastrulation brain homeo box 2	0,0214687	0,867
243956_at	**SUSD3**	sushi domain containing 3	0,0214687	0,872
238931_at	**LOC284009**	NA	0,0214933	0,660
220186_s_at	**PCLKC**	NA	0,0215414	0,900
203482_at	**C10orf6**	chromosome 10 open reading frame 6	0,0215451	0,873
219692_at	**KREMEN2**	kringle containing transmembrane protein 2	0,0215823	0,884
1569246_a_at	**LOC203076**	NA	0,0216344	0,865
204707_s_at	**MAPK4**	mitogen-activated protein kinase 4	0,0216352	0,842
235439_at	**RBMS2**	RNA binding motif, single stranded interacting protein 2	0,0216352	0,779
225985_at	**PRKAA1**	protein kinase, AMP-activated, alpha 1 catalytic subunit	0,0216410	0,792
233362_at	**ZNF341**	zinc finger protein 341	0,0216869	0,799
211545_at	**GHRHR**	growth hormone releasing hormone receptor	0,0216908	0,894
226972_s_at	**NAG6**	NA	0,0216908	0,681
229976_at	**C9orf18**	chromosome 9 open reading frame 18	0,0217031	0,907
1562559_at	**C9orf89**	chromosome 9 open reading frame 89	0,0217142	0,826
207170_s_at	**LETMD1**	LETM1 domain containing 1	0,0217142	0,680
1562638_at	**LOC339874**	NA	0,0217142	0,905
242071_x_at	**ITGA8**	integrin, alpha 8	0,0217275	0,719
222203_s_at	**RDH14**	retinol dehydrogenase 14 (all-trans and 9-cis)	0,0217361	0,881
222082_at	**ZBTB7A**	zinc finger and BTB domain containing 7A	0,0217408	0,825
226409_at	**TBC1D20**	TBC1 domain family, member 20	0,0217465	0,871
1553607_at	**C21orf109**	chromosome 21 open reading frame 109	0,0217685	0,909
224211_at	**FOXP3**	forkhead box P3	0,0217719	0,906
228886_at	**LRRC27**	leucine rich repeat containing 27	0,0217762	0,783
205200_at	**CLEC3B**	C-type lectin domain family 3, member B	0,0217874	0,527

229411_at	**PNCK**	pregnancy upregulated non-ubiquitously expressed CaM kinase	0,0218312	0,892
227307_at	**TSPAN18**	tetraspanin 18	0,0218338	0,460
243123_at	**PLD3**	phospholipase D family, member 3	0,0218539	0,877
1561614_at	**SLC8A1**	solute carrier family 8 (sodium/calcium exchanger), member 1	0,0218590	0,844
1552714_at	**CREG2**	cellular repressor of E1A-stimulated genes 2	0,0218822	0,914
213125_at	**OLFML2B**	olfactomedin-like 2B	0,0218822	0,477
212329_at	**SCAP**	NA	0,0218822	0,710
213885_at	**TRIM3**	tripartite motif-containing 3	0,0218826	0,805
224245_at	**INGX**	inhibitor of growth family, X-linked, pseudogene	0,0219131	0,862
203831_at	**KIAA1002**	NA	0,0219329	0,835
218637_at	**IMPACT**	NA	0,0219392	0,822
238678_at	**LOC402483**	NA	0,0219898	0,777
1560253_at	**LHX9**	LIM homeobox 9	0,0220233	0,862
228575_at	**FNDC6**	fibronectin type III domain containing 6	0,0220475	0,529
235455_at	**C1orf117**	chromosome 1 open reading frame 117	0,0220762	0,848
207632_at	**MUSK**	muscle, skeletal, receptor tyrosine kinase	0,0220762	0,872
227726_at	**RNF166**	ring finger protein 166	0,0220762	0,770
1568408_x_at	**MGC27165**	NA	0,0220778	0,825
236311_at	**LOH12CR2**	loss of heterozygosity, 12, chromosomal region 2	0,0220990	0,690
232923_at	**MICAL3**	microtubule associated monoxygenase, calponin and LIM domain containing 3	0,0221862	0,851
213991_s_at	**HS3ST1**	heparan sulfate (glucosamine) 3-O-sulfotransferase 1	0,0222483	0,855
234381_at	**IGLC2**	immunoglobulin lambda constant 2 (Kern-Oz- marker)	0,0222506	0,892
231466_at	**NYD-SP18**	NA	0,0222506	0,865
214640_at	**UNC93A**	unc-93 homolog A (C. elegans)	0,0222638	0,853
220679_s_at	**CDH7**	cadherin 7, type 2	0,0222909	0,899
222905_s_at	**FLJ10922**	NA	0,0222909	0,838
215980_s_at	**IGHMBP2**	immunoglobulin mu binding protein 2	0,0222909	0,877
226510_at	**C14orf125**	chromosome 14 open reading frame 125	0,0222913	0,808
235058_at	**ATPBD1B**	ATP binding domain 1 family, member B	0,0223115	0,720
219223_at	**C9orf7**	chromosome 9 open reading frame 7	0,0223200	0,849
1561508_at	**MRPL38**	mitochondrial ribosomal protein L38	0,0223841	0,896
210618_at	**RAP1GA1**	RAP1, GTPase activating protein 1	0,0224157	0,856
230124_at	**TMEM30B**	transmembrane protein 30B	0,0224173	0,841
212403_at	**UBE3B**	ubiquitin protein ligase E3B	0,0225169	0,830
228896_at	**LOC389108**	NA	0,0225927	0,816
227470_at	**ZNF553**	zinc finger protein 553	0,0226341	0,893
219779_at	**ZFHX4**	zinc finger homeodomain 4	0,0226652	0,635
207502_at	**GUCA2B**	guanylate cyclase activator 2B (uroguanylin)	0,0226748	0,862
203977_at	**TAZ**	c("tafazzin (cardiomyopathy, dilated 3A (X-linked)", " endocardial fibroelastosis 2", " Barth syndrome)")	0,0226925	0,833
201161_s_at	**CSDA**	cold shock domain protein A	0,0227316	0,796
205198_s_at	**ATP7A**	ATPase, Cu++ transporting, alpha polypeptide (Menkes syndrome)	0,0227363	0,789
202111_at	**SLC4A2**	solute carrier family 4, anion exchanger, member 2 (erythrocyte membrane protein band 3-like 1)	0,0227363	0,677
1557224_at	**MLSTD1**	male sterility domain containing 1	0,0227628	0,604
214555_at	**SSTR5**	somatostatin receptor 5	0,0227628	0,801
221889_at	**KCTD13**	potassium channel tetramerisation domain containing 13	0,0228044	0,791
242684_at	**ZNF425**	zinc finger protein 425	0,0228243	0,858
205971_s_at	**CTRB1**	chymotrypsinogen B1	0,0228373	0,805
208790_s_at	**PTRF**	polymerase I and transcript release factor	0,0228562	0,645
223833_at	**WDR55**	WD repeat domain 55	0,0228875	0,829
1553214_a_at	**CCDC7**	coiled-coil domain containing 7	0,0229245	0,929
212494_at	**TENC1**	tensin like C1 domain containing phosphatase (tensin 2)	0,0229335	0,735
226796_at	**LOC116236**	NA	0,0230015	0,709

241009_at	**FOXN4**	forkhead box N4	0,0230044	0,888
210413_x_at	**SERPINB4**	serpin peptidase inhibitor, clade B (ovalbumin), member 4	0,0230550	0,880
1561635_at	**FLJ32810**	NA	0,0230671	0,887
243899_at	**LOC51326**	NA	0,0230671	0,821
1556881_at	**LOC440856**	NA	0,0230701	0,878
212865_s_at	**COL14A1**	collagen, type XIV, alpha 1 (undulin)	0,0230733	0,777
209117_at	**WBP2**	WW domain binding protein 2	0,0230733	0,476
238422_at	**LOC151534**	NA	0,0230770	0,813
213043_s_at	**THRAP4**	thyroid hormone receptor associated protein 4	0,0230776	0,809
202652_at	**APBB1**	amyloid beta (A4) precursor protein-binding, family B, member 1 (Fe65)	0,0231123	0,813
228137_s_at	**PPP2R2C**	protein phosphatase 2 (formerly 2A), regulatory subunit B (PR 52), gamma isoform	0,0231123	0,839
225861_at	**MGC15416**	NA	0,0231131	0,788
218215_s_at	**NR1H2**	nuclear receptor subfamily 1, group H, member 2	0,0231131	0,700
241380_at	**FLJ41603**	NA	0,0231238	0,855
204450_x_at	**APOA1**	apolipoprotein A-I	0,0231239	0,866
242212_at	**RGS16**	regulator of G-protein signalling 16	0,0231239	0,900
225689_at	**C3orf39**	NA	0,0232089	0,633
225117_at	**LOC284058**	NA	0,0232089	0,815
218607_s_at	**SDAD1**	SDA1 domain containing 1	0,0232089	0,666
1557119_a_at	**ZNF575**	zinc finger protein 575	0,0232275	0,831
232983_s_at	**DELGEF**	NA	0,0232378	0,820
222526_at	**GATAD2A**	GATA zinc finger domain containing 2A	0,0232378	0,854
227706_at	**SPIRE2**	spire homolog 2 (Drosophila)	0,0232378	0,852
1552906_at	**FMR1NB**	fragile X mental retardation 1 neighbor	0,0232588	0,919
233504_at	**C9orf84**	chromosome 9 open reading frame 84	0,0232633	0,886
218661_at	**FLJ14154**	NA	0,0232773	0,776
203546_at	**IPO13**	importin 13	0,0232773	0,749
218749_s_at	**SLC24A6**	solute carrier family 24 (sodium/potassium/calcium exchanger), member 6	0,0233593	0,783
215735_s_at	**TSC2**	tuberous sclerosis 2	0,0233593	0,622
207511_s_at	**C2orf24**	chromosome 2 open reading frame 24	0,0233724	0,778
227219_x_at	**MAP1LC3A**	microtubule-associated protein 1 light chain 3 alpha	0,0233822	0,747
236556_s_at	**LONRF1**	LON peptidase N-terminal domain and ring finger 1	0,0233835	0,880
232936_at	**KCNA7**	potassium voltage-gated channel, shaker-related subfamily, member 7	0,0233933	0,903
235604_x_at	**ZNF493**	zinc finger protein 493	0,0234019	0,703
209741_x_at	**ZNF291**	zinc finger protein 291	0,0234030	0,834
215673_at	**KIAA1655**	NA	0,0234104	0,894
205223_at	**DEPDC5**	DEP domain containing 5	0,0234119	0,880
219213_at	**JAM2**	junctional adhesion molecule 2	0,0234119	0,535
209044_x_at	**SF3B4**	splicing factor 3b, subunit 4, 49kDa	0,0234119	0,843
214823_at	**ZNF204**	zinc finger protein 204	0,0234256	0,699
217451_at	**COX5A**	cytochrome c oxidase subunit Va	0,0234371	0,877
1562606_a_at	**LOC440028**	NA	0,0234442	0,808
221759_at	**G6PC3**	glucose 6 phosphatase, catalytic, 3	0,0235219	0,793
212441_at	**KIAA0232**	NA	0,0235343	0,886
1555646_at	**Dbpht2**	DNA binding protein with his-thr domain	0,0235363	0,921
241277_x_at	**LOC116412**	NA	0,0236033	0,830
228302_x_at	**CAMK2N1**	calcium/calmodulin-dependent protein kinase II inhibitor 1	0,0236325	0,492
225312_at	**COMMD6**	COMM domain containing 6	0,0236325	0,860
235145_at	**ZBTB7B**	zinc finger and BTB domain containing 7B	0,0236325	0,819
1553071_a_at	**MYOZ3**	myozenin 3	0,0236405	0,851
216842_x_at	**RBMY2FP**	RNA binding motif protein, Y-linked, family 2, member F pseudogene	0,0236544	0,796

1557185_at	**TPCN1**	two pore segment channel 1	0,0236544	0,888
1558238_at	**TNRC18**	trinucleotide repeat containing 18	0,0236974	0,875
208559_at	**IPF1**	insulin promoter factor 1, homeodomain transcription factor	0,0237029	0,878
61297_at	**CASKIN2**	CASK interacting protein 2	0,0237128	0,732
208133_at	**RFC1**	replication factor C (activator 1) 1, 145kDa	0,0237197	0,871
219765_at	**ZNF329**	zinc finger protein 329	0,0237215	0,830
1562301_at	**C8orf34**	chromosome 8 open reading frame 34	0,0237395	0,803
1555052_a_at	**SYT9**	synaptotagmin IX	0,0237590	0,882
205869_at	**PRSS1**	protease, serine, 1 (trypsin 1)	0,0237613	0,866
1555339_at	**RAP1A**	RAP1A, member of RAS oncogene family	0,0237613	0,910
229142_s_at	**BBS1**	Bardet-Biedl syndrome 1	0,0237669	0,864
1557295_a_at	**CRIM1**	cysteine rich transmembrane BMP regulator 1 (chordin-like)	0,0237812	0,813
210879_s_at	**RAB11FIP5**	RAB11 family interacting protein 5 (class I)	0,0237812	0,794
217069_at	**MLL4**	NA	0,0238401	0,821
203190_at	**NDUFS8**	NADH dehydrogenase (ubiquinone) Fe-S protein 8, 23kDa (NADH-coenzyme Q reductase)	0,0238523	0,812
223186_at	**Kua**	NA	0,0238632	0,795
225701_at	**AKNA**	AT-hook transcription factor	0,0238634	0,696
218702_at	**SARS2**	seryl-tRNA synthetase 2	0,0238741	0,844
205489_at	**CRYM**	crystallin, mu	0,0239300	0,776
243986_at	**LOC144766**	NA	0,0239475	0,890
202959_at	**MUT**	methylmalonyl Coenzyme A mutase	0,0239475	0,806
208504_x_at	**PCDHB11**	protocadherin beta 11	0,0239625	0,834
229186_s_at	**ZFP64**	zinc finger protein 64 homolog (mouse)	0,0240298	0,843
229326_at	**TNFSF13**	tumor necrosis factor (ligand) superfamily, member 13	0,0240617	0,831
205993_s_at	**TBX2**	T-box 2	0,0240886	0,714
212630_at	**SEC6L1**	SEC6-like 1 (S. cerevisiae)	0,0241303	0,916
217806_s_at	**POLDIP2**	polymerase (DNA-directed), delta interacting protein 2	0,0241566	0,864
215369_at	**LOC442436**	NA	0,0241879	0,843
212864_at	**CDS2**	CDP-diacylglycerol synthase (phosphatidate cytidylyltransferase) 2	0,0242010	0,868
214679_x_at	**GNA11**	guanine nucleotide binding protein (G protein), alpha 11 (Gq class)	0,0242010	0,768
231743_at	**WNT3**	wingless-type MMTV integration site family, member 3	0,0242787	0,893
1555483_x_at	**FBLIM1**	filamin binding LIM protein 1	0,0242827	0,781
203286_at	**RNF44**	ring finger protein 44	0,0242847	0,769
237464_at	**IMAA**	NA	0,0243515	0,872
224814_at	**DPP7**	dipeptidylpeptidase 7	0,0243815	0,745
240744_at	**CPA5**	carboxypeptidase A5	0,0243838	0,859
216264_s_at	**LAMB2**	laminin, beta 2 (laminin S)	0,0243853	0,752
215699_x_at	**SFI1**	Sfi1 homolog, spindle assembly associated (yeast)	0,0243853	0,893
219475_at	**OKL38**	NA	0,0243910	0,814
215585_at	**KIAA0174**	KIAA0174	0,0244430	0,905
240871_at	**C9orf128**	chromosome 9 open reading frame 128	0,0244751	0,771
201285_at	**MKRN1**	makorin, ring finger protein, 1	0,0244849	0,830
229140_at	**ZNF579**	zinc finger protein 579	0,0244849	0,845
219687_at	**HHAT**	hedgehog acyltransferase	0,0245282	0,805
220385_at	**JPH2**	junctophilin 2	0,0245346	0,864
212152_x_at	**ARID1A**	AT rich interactive domain 1A (SWI- like)	0,0245549	0,848
232950_s_at	**PITPNM2**	phosphatidylinositol transfer protein, membrane-associated 2	0,0245558	0,851
211891_s_at	**ARHGEF4**	Rho guanine nucleotide exchange factor (GEF) 4	0,0246129	0,852
1561967_at	**FLNB**	filamin B, beta (actin binding protein 278)	0,0246764	0,896
218636_s_at	**MAN1B1**	mannosidase, alpha, class 1B, member 1	0,0246764	0,801
237256_at	**FBXL12**	F-box and leucine-rich repeat protein 12	0,0246926	0,797
235896_s_at	**SMCR7**	Smith-Magenis syndrome chromosome region, candidate 7	0,0246926	0,778
241478_at	**MICAL-L2**	NA	0,0246987	0,856

224024_at	**KIAA1181**	NA	0,0247341	0,926
221024_s_at	**SLC2A10**	solute carrier family 2 (facilitated glucose transporter), member 10	0,0248467	0,775
1555257_a_at	**MYO3B**	myosin IIIB	0,0249511	0,890
1553812_at	**TLE6**	transducin-like enhancer of split 6 (E(sp1) homolog, Drosophila)	0,0249746	0,819
208566_at	**KCNJ12**	potassium inwardly-rectifying channel, subfamily J, member 12	0,0250050	0,890
206821_x_at	**HRBL**	HIV-1 Rev binding protein-like	0,0250254	0,891
244718_at	**LOC339483**	NA	0,0250275	0,829
1556684_at	**PARP2**	poly (ADP-ribose) polymerase family, member 2	0,0250275	0,789
230500_at	**PDE7A**	phosphodiesterase 7A	0,0250350	0,925
241704_x_at	**ZFPL**	NA	0,0250514	0,878
240048_at	**CATSPER2**	cation channel, sperm associated 2	0,0250565	0,891
208497_x_at	**NEUROG1**	neurogenin 1	0,0250611	0,893
205477_s_at	**AMBP**	alpha-1-microglobulin/bikunin precursor	0,0250715	0,856
241149_at	**KLHDC4**	kelch domain containing 4	0,0250715	0,803
212285_s_at	**AGRN**	agrin	0,0251117	0,713
235292_at	**LOC441069**	NA	0,0251137	0,704
233453_at	**DKFZp761A052**	NA	0,0251307	0,903
210193_at	**MOBP**	myelin-associated oligodendrocyte basic protein	0,0251913	0,902
209872_s_at	**PKP3**	plakophilin 3	0,0251913	0,854
224932_at	**C22orf16**	chromosome 22 open reading frame 16	0,0251955	0,685
208173_at	**IFNB1**	interferon, beta 1, fibroblast	0,0252836	0,813
205160_at	**PEX11A**	peroxisomal biogenesis factor 11A	0,0253269	0,743
228929_at	**DNASE1**	deoxyribonuclease I	0,0253542	0,896
218770_s_at	**TMEM39B**	transmembrane protein 39B	0,0253696	0,832
1555914_a_at	**FLJ20433**	NA	0,0253730	0,878
215264_at	**EMX1**	empty spiracles homolog 1 (Drosophila)	0,0253918	0,897
205025_at	**HKR3**	GLI-Kruppel family member HKR3	0,0254197	0,710
218475_at	**HTF9C**	NA	0,0254285	0,746
202273_at	**PDGFRB**	platelet-derived growth factor receptor, beta polypeptide	0,0254511	0,748
1553511_at	**GJC1**	gap junction protein, chi 1, 31.9kDa (connexin 31.9)	0,0254530	0,879
208348_s_at	**CBLB**	Cas-Br-M (murine) ecotropic retroviral transforming sequence b	0,0254673	0,896
235783_at	**C1orf33**	chromosome 1 open reading frame 33	0,0255275	0,628
227024_s_at	**MRPL55**	mitochondrial ribosomal protein L55	0,0255380	0,818
239182_at	**LOC401022**	NA	0,0255404	0,938
237654_at	**C14orf50**	chromosome 14 open reading frame 50	0,0255515	0,800
240340_at	**SPATA3**	spermatogenesis associated 3	0,0255690	0,835
240021_at	**LOC388173**	NA	0,0256071	0,843
204737_s_at	**MYH7**	myosin, heavy polypeptide 7, cardiac muscle, beta	0,0256177	0,890
215625_at	**LOC441014**	NA	0,0256322	0,855
202023_at	**EFNA1**	ephrin-A1	0,0256933	0,826
207137_at	**NFKBIL2**	nuclear factor of kappa light polypeptide gene enhancer in B-cells inhibitor-like 2	0,0257183	0,817
224980_at	**LEMD2**	LEM domain containing 2	0,0257500	0,767
213486_at	**DKFZP761N09121**	NA	0,0257685	0,686
1552538_a_at	**C6orf102**	chromosome 6 open reading frame 102	0,0257806	0,882
1554643_at	**RGS11**	regulator of G-protein signalling 11	0,0257809	0,875
204099_at	**SMARCD3**	SWI/SNF related, matrix associated, actin dependent regulator of chromatin, subfamily d, member 3	0,0257809	0,772
222998_at	**MAF1**	MAF1 homolog (S. cerevisiae)	0,0258072	0,825
1564705_at	**GLS2**	glutaminase 2 (liver, mitochondrial)	0,0258463	0,922
1559152_at	**C6orf85**	chromosome 6 open reading frame 85	0,0259631	0,899
208603_s_at	**MAPK8IP2**	mitogen-activated protein kinase 8 interacting protein 2	0,0260236	0,880
220403_s_at	**P53AIP1**	NA	0,0260531	0,852
214080_x_at	**PRKCSH**	protein kinase C substrate 80K-H	0,0260531	0,880
221783_at	**WIZ**	NA	0,0260531	0,841

204660_at	**GFER**	growth factor, augmenter of liver regeneration (ERV1 homolog, S. cerevisiae)	0,0261162	0,817
226716_at	**KIAA1205**	KIAA1205	0,0261162	0,757
234451_at	**LOC391241**	NA	0,0261162	0,844
219855_at	**NUDT11**	nudix (nucleoside diphosphate linked moiety X)-type motif 11	0,0261453	0,790
235839_at	**LOC440835**	NA	0,0261493	0,923
238080_at	**Beta4GalNAc-T4**	NA	0,0261535	0,835
204664_at	**ALPP**	alkaline phosphatase, placental (Regan isozyme)	0,0262108	0,880
203002_at	**AMOTL2**	angiomotin like 2	0,0262108	0,782
236744_at	**PHPT1**	phosphohistidine phosphatase 1	0,0262108	0,797
218744_s_at	**PACSIN3**	protein kinase C and casein kinase substrate in neurons 3	0,0262205	0,749
204776_at	**THBS4**	thrombospondin 4	0,0262205	0,860
203759_at	**ST3GAL4**	ST3 beta-galactoside alpha-2,3-sialyltransferase 4	0,0262852	0,768
237229_at	**FLJ13798**	NA	0,0262915	0,887
208105_at	**GIPR**	gastric inhibitory polypeptide receptor	0,0263287	0,893
1552993_at	**DYDC1**	DPY30 domain containing 1	0,0263664	0,850
239763_at	**PRDM11**	PR domain containing 11	0,0264123	0,830
202500_at	**DNAJB2**	DnaJ (Hsp40) homolog, subfamily B, member 2	0,0264217	0,827
231276_at	**PDE3B**	phosphodiesterase 3B, cGMP-inhibited	0,0264261	0,851
227158_at	**C14orf126**	chromosome 14 open reading frame 126	0,0264454	0,831
227829_at	**GYLTL1B**	glycosyltransferase-like 1B	0,0265193	0,883
243120_at	**LOC440116**	NA	0,0265280	0,863
1556067_a_at	**JMJD3**	jumonji domain containing 3	0,0265350	0,780
1560488_at	**FLJ45224**	NA	0,0266069	0,895
217470_at	**LOC389248**	NA	0,0266069	0,924
1554907_a_at	**HYDIN**	hydrocephalus inducing	0,0266210	0,851
1556555_at	**LOC441126**	NA	0,0266443	0,817
210100_s_at	**ABCA2**	ATP-binding cassette, sub-family A (ABC1), member 2	0,0266583	0,705
231338_at	**NUT**	NA	0,0266909	0,851
1561928_s_at	**C3orf16**	chromosome 3 open reading frame 16	0,0267359	0,864
65635_at	**FLJ21865**	NA	0,0267961	0,890
1557717_at	**LOC338862**	NA	0,0267961	0,937
233026_s_at	**PDZK3**	PDZ domain containing 3	0,0267961	0,777
233811_at	**RIN2**	Ras and Rab interactor 2	0,0267961	0,882
218086_at	**NPDC1**	neural proliferation, differentiation and control, 1	0,0267978	0,605
204166_at	**KIAA0963**	KIAA0963	0,0268054	0,754
240553_at	**TSC**	NA	0,0268317	0,889
238930_at	**FLJ40125**	NA	0,0268331	0,835
222695_s_at	**AXIN2**	axin 2 (conductin, axil)	0,0268416	0,906
230765_at	**KIAA1239**	NA	0,0268669	0,887
206629_at	**ADAMTSL2**	ADAMTS-like 2	0,0268908	0,821
213676_at	**LOC441151**	NA	0,0269718	0,821
229356_x_at	**INO80**	NA	0,0269841	0,868
207570_at	**SHOX**	short stature homeobox	0,0269841	0,774
237693_at	**RIMS2**	regulating synaptic membrane exocytosis 2	0,0269888	0,901
236427_at	**LOC441776**	NA	0,0269940	0,898
207036_x_at	**GRIN2D**	glutamate receptor, ionotropic, N-methyl D-aspartate 2D	0,0270149	0,872
215541_s_at	**DIAPH1**	diaphanous homolog 1 (Drosophila)	0,0270315	0,778
213622_at	**COL9A2**	collagen, type IX, alpha 2	0,0270554	0,855
230893_at	**DNAJA5**	NA	0,0270732	0,711
226075_at	**SPSB1**	splA/ryanodine receptor domain and SOCS box containing 1	0,0270732	0,737
214623_at	**BCR**	breakpoint cluster region	0,0270738	0,828
1565339_at	**DNAH10**	dynein, axonemal, heavy polypeptide 10	0,0270914	0,932
209831_x_at	**DNASE2**	deoxyribonuclease II, lysosomal	0,0270951	0,805

206883_x_at	**GP9**	glycoprotein IX (platelet)	0,0271294	0,858
236813_at	**C10orf83**	chromosome 10 open reading frame 83	0,0271764	0,816
214571_at	**FGF3**	fibroblast growth factor 3 (murine mammary tumor virus integration site (v-int-2) oncogene homolog)	0,0271764	0,857
218634_at	**PHLDA3**	pleckstrin homology-like domain, family A, member 3	0,0272205	0,733
218952_at	**PCSK1N**	proprotein convertase subtilisin/kexin type 1 inhibitor	0,0272633	0,777
1569788_at	**ST8SIA1**	ST8 alpha-N-acetyl-neuraminide alpha-2,8-sialyltransferase 1	0,0272732	0,857
235286_at	**CKLF**	chemokine-like factor	0,0272739	0,749
242543_at	**LOC284948**	NA	0,0273372	0,875
232258_at	**TCTE3**	t-complex-associated-testis-expressed 3	0,0273386	0,904
225848_at	**FLJ31413**	NA	0,0273486	0,772
1553030_a_at	**SUOX**	sulfite oxidase	0,0274154	0,841
1553517_at	**FERD3L**	Fer3-like (Drosophila)	0,0274288	0,889
230833_at	**ACRBP**	acrosin binding protein	0,0274476	0,735
235383_at	**MYO7B**	myosin VIIB	0,0274738	0,876
207109_at	**POU2F3**	POU domain, class 2, transcription factor 3	0,0274738	0,896
241899_at	**SLC22A4**	solute carrier family 22 (organic cation transporter), member 4	0,0274738	0,931
201746_at	**TP53**	tumor protein p53 (Li-Fraumeni syndrome)	0,0274738	0,750
209350_s_at	**GPS2**	G protein pathway suppressor 2	0,0275191	0,774
221902_at	**GPR153**	G protein-coupled receptor 153	0,0275336	0,742
218279_s_at	**HIST2H2AA**	histone 2, H2aa	0,0275702	0,866
203108_at	**GPRC5A**	G protein-coupled receptor, family C, group 5, member A	0,0275936	0,351
234307_s_at	**KIF26A**	kinesin family member 26A	0,0275936	0,896
210683_at	**NRTN**	neurturin	0,0275936	0,849
227321_at	**MGC2463**	NA	0,0276314	0,862
219058_x_at	**TINAGL1**	tubulointerstitial nephritis antigen-like 1	0,0276403	0,599
211838_x_at	**PCDHA5**	protocadherin alpha 5	0,0276417	0,849
209497_s_at	**RBM30**	RNA binding motif protein 30	0,0276898	0,786
1566851_at	**TRIM42**	tripartite motif-containing 42	0,0277598	0,896
218383_at	**C14orf94**	chromosome 14 open reading frame 94	0,0277781	0,800
220136_s_at	**CRYBA2**	crystallin, beta A2	0,0277781	0,827
216670_at	**KLK13**	kallikrein 13	0,0277977	0,783
1555063_at	**USP6**	ubiquitin specific peptidase 6 (Tre-2 oncogene)	0,0277977	0,884
210212_x_at	**MTCP1**	mature T-cell proliferation 1	0,0278055	0,852
203858_s_at	**COX10**	COX10 homolog, cytochrome c oxidase assembly protein, heme A: farnesyltransferase (yeast)	0,0278187	0,908
226956_at	**LOC400924**	NA	0,0278434	0,830
205666_at	**FMO1**	flavin containing monooxygenase 1	0,0279103	0,907
231921_at	**FLJ13096**	NA	0,0279190	0,778
213210_at	**TAF6L**	TAF6-like RNA polymerase II, p300/CBP-associated factor (PCAF)-associated factor, 65kDa	0,0279227	0,815
229570_at	**LAMA5**	laminin, alpha 5	0,0279502	0,800
206517_at	**CDH16**	cadherin 16, KSP-cadherin	0,0280031	0,820
209243_s_at	**PEG3**	paternally expressed 3	0,0280097	0,775
216381_x_at	**AKR7A3**	aldo-keto reductase family 7, member A3 (aflatoxin aldehyde reductase)	0,0280168	0,865
227739_at	**LOC389822**	NA	0,0280194	0,896
221347_at	**CHRM5**	cholinergic receptor, muscarinic 5	0,0280303	0,901
243121_x_at	**LOC91664**	NA	0,0280303	0,888
226533_at	**HINT3**	histidine triad nucleotide binding protein 3	0,0280366	0,468
232052_at	**LOC401051**	NA	0,0280532	0,856
234376_at	**MYCN**	v-myc myelocytomatosis viral related oncogene, neuroblastoma derived (avian)	0,0280543	0,899
223801_s_at	**APOL4**	apolipoprotein L, 4	0,0281131	0,802
231139_at	**ALOX5**	arachidonate 5-lipoxygenase	0,0281208	0,888
225616_at	**LOC283377**	NA	0,0281208	0,855

1558578_a_at	**SLC13A4**	solute carrier family 13 (sodium/sulfate symporters), member 4	0,0281208	0,906
205125_at	**PLCD1**	phospholipase C, delta 1	0,0281258	0,713
205079_s_at	**MPDZ**	multiple PDZ domain protein	0,0281510	0,741
243421_at	**TACR1**	tachykinin receptor 1	0,0282011	0,940
234810_at	**ZFP1**	zinc finger protein 1 homolog (mouse)	0,0282011	0,821
237335_at	**ZP1**	zona pellucida glycoprotein 1 (sperm receptor)	0,0282011	0,826
204765_at	**ARHGEF5**	Rho guanine nucleotide exchange factor (GEF) 5	0,0282624	0,835
206851_at	**RNASE3**	ribonuclease, RNase A family, 3 (eosinophil cationic protein)	0,0282624	0,872
209877_at	**SNCG**	synuclein, gamma (breast cancer-specific protein 1)	0,0282795	0,844
202481_at	**DHRS3**	dehydrogenase/reductase (SDR family) member 3	0,0282933	0,404
220587_s_at	**GBL**	NA	0,0283103	0,633
223694_at	**TRIM7**	tripartite motif-containing 7	0,0283159	0,858
232265_at	**ATXN7L1**	ataxin 7-like 1	0,0283247	0,861
243387_at	**MESP1**	NA	0,0283592	0,880
217732_s_at	**ITM2B**	integral membrane protein 2B	0,0283660	0,848
207159_x_at	**MECT1**	mucoepidermoid carcinoma translocated 1	0,0283998	0,890
206016_at	**CCDC22**	coiled-coil domain containing 22	0,0284118	0,771
222052_at	**FLJ41131**	NA	0,0284118	0,808
205448_s_at	**MAP3K12**	mitogen-activated protein kinase kinase kinase 12	0,0284222	0,760
1561990_at	**LOC157931**	NA	0,0284666	0,905
1555491_a_at	**FLJ11286**	NA	0,0284809	0,774
206399_x_at	**CACNA1A**	calcium channel, voltage-dependent, P/Q type, alpha 1A subunit	0,0284873	0,879
218144_s_at	**C14orf173**	chromosome 14 open reading frame 173	0,0285426	0,842
220163_s_at	**HR**	hairless homolog (mouse)	0,0285452	0,913
230714_s_at	**ZNF286**	zinc finger protein 286	0,0285496	0,850
206142_at	**ZNF135**	zinc finger protein 135 (clone pHZ-17)	0,0285902	0,736
209215_at	**TETRAN**	NA	0,0286001	0,816
207424_at	**MYF5**	myogenic factor 5	0,0286213	0,902
206738_at	**APOC4**	apolipoprotein C-IV	0,0286354	0,870
208327_at	**CYP2A13**	cytochrome P450, family 2, subfamily A, polypeptide 13	0,0286355	0,793
244326_at	**FLJ11078**	NA	0,0286355	0,908
230435_at	**FLJ30851**	NA	0,0286355	0,674
202745_at	**USP8**	ubiquitin specific peptidase 8	0,0286355	0,900
35265_at	**FXR2**	fragile X mental retardation, autosomal homolog 2	0,0286419	0,842
34408_at	**RTN2**	reticulon 2	0,0286425	0,751
1557380_at	**KIAA1975**	NA	0,0287633	0,865
1556883_a_at	**LOC401528**	NA	0,0288310	0,876
224904_at	**LOC400541**	NA	0,0288735	0,801
216055_at	**PDGFB**	platelet-derived growth factor beta polypeptide (simian sarcoma viral (v-sis) oncogene homolog)	0,0288744	0,864
209675_s_at	**HNRPUL1**	heterogeneous nuclear ribonucleoprotein U-like 1	0,0288842	0,741
229566_at	**LOC440449**	NA	0,0289234	0,676
233106_at	**C14orf82**	chromosome 14 open reading frame 82	0,0289578	0,778
207091_at	**P2RX7**	purinergic receptor P2X, ligand-gated ion channel, 7	0,0289578	0,612
219584_at	**PLA1A**	phospholipase A1 member A	0,0289675	0,771
225886_at	**DDX5**	DEAD (Asp-Glu-Ala-Asp) box polypeptide 5	0,0289813	0,743
223607_x_at	**ZSWIM1**	zinc finger, SWIM-type containing 1	0,0290307	0,906
1557765_at	**LOC340109**	NA	0,0290393	0,908
1555251_a_at	**OTOF**	otoferlin	0,0290393	0,899
211033_s_at	**PEX7**	peroxisomal biogenesis factor 7	0,0290493	0,892
211975_at	**ZNF289**	zinc finger protein 289, ID1 regulated	0,0290603	0,874
215824_at	**NUDT7**	nudix (nucleoside diphosphate linked moiety X)-type motif 7	0,0291260	0,902
205352_at	**SERPINI1**	serpin peptidase inhibitor, clade I (neuroserpin), member 1	0,0291260	0,563
219172_at	**UBTD1**	ubiquitin domain containing 1	0,0291260	0,674

1559827_at	**LOC401074**	NA	0,0291266	0,793
234281_at	**ESPNP**	espin pseudogene	0,0291463	0,866
207809_s_at	**ATP6AP1**	ATPase, H+ transporting, lysosomal accessory protein 1	0,0291877	0,777
228027_at	**GPRASP2**	G protein-coupled receptor associated sorting protein 2	0,0291882	0,711
1552340_at	**SP7**	Sp7 transcription factor	0,0292094	0,885
1553634_a_at	**FLJ40852**	NA	0,0292198	0,756
206871_at	**ELA2**	elastase 2, neutrophil	0,0292250	0,836
203685_at	**BCL2**	B-cell CLL/lymphoma 2	0,0292987	0,432
203081_at	**CTNNBIP1**	catenin, beta interacting protein 1	0,0292987	0,846
232678_at	**SLC2A9**	solute carrier family 2 (facilitated glucose transporter), member 9	0,0292987	0,895
233961_at	**GPR51**	G protein-coupled receptor 51	0,0293084	0,899
227006_at	**PPP1R14A**	protein phosphatase 1, regulatory (inhibitor) subunit 14A	0,0293084	0,517
221141_x_at	**EPN1**	epsin 1	0,0293284	0,837
227393_at	**TMEM16J**	transmembrane protein 16J	0,0293856	0,877
241759_at	**MGC5566**	NA	0,0293878	0,839
214311_at	**ZFPL1**	zinc finger protein-like 1	0,0294284	0,794
1554655_a_at	**LOC388394**	NA	0,0294436	0,874
207559_s_at	**ZNF261**	zinc finger protein 261	0,0294608	0,893
209419_at	**C22orf19**	chromosome 22 open reading frame 19	0,0294682	0,886
1563475_s_at	**MGC50559**	NA	0,0294682	0,863
202549_at	**VAPB**	VAMP (vesicle-associated membrane protein)-associated protein B and C	0,0294682	0,845
218760_at	**COQ6**	coenzyme Q6 homolog (yeast)	0,0295076	0,868
234891_at	**DKFZP547L112**	NA	0,0295076	0,860
236463_at	**LOC161931**	NA	0,0295170	0,761
203683_s_at	**VEGFB**	vascular endothelial growth factor B	0,0295170	0,427
233918_at	**LOC149069**	NA	0,0295174	0,900
220956_s_at	**EGLN2**	egl nine homolog 2 (C. elegans)	0,0295559	0,883
230576_at	**BLOC1S3**	biogenesis of lysosome-related organelles complex-1, subunit 3	0,0296832	0,870
236661_at	**LOC440956**	NA	0,0296850	0,877
207705_s_at	**KIAA0980**	NA	0,0296884	0,707
239027_at	**DOCK8**	dedicator of cytokinesis 8	0,0296967	0,828
1553970_s_at	**CEL**	carboxyl ester lipase (bile salt-stimulated lipase)	0,0296998	0,818
217609_at	**LOC283345**	NA	0,0297233	0,697
1565698_at	**HECTD2**	HECT domain containing 2	0,0298015	0,897
201715_s_at	**ACIN1**	apoptotic chromatin condensation inducer 1	0,0298140	0,745
233848_x_at	**ZNF221**	zinc finger protein 221	0,0298589	0,885
221034_s_at	**TEX13B**	testis expressed sequence 13B	0,0298630	0,861
232255_at	**LOC401321**	NA	0,0298950	0,878
210632_s_at	**SGCA**	sarcoglycan, alpha (50kDa dystrophin-associated glycoprotein)	0,0298950	0,760
220513_at	**C6orf148**	chromosome 6 open reading frame 148	0,0299340	0,866
1553778_at	**WBSCR27**	Williams Beuren syndrome chromosome region 27	0,0299495	0,831
213363_at	**CA5BL**	carbonic anhydrase VB-like	0,0299632	0,766
214302_x_at	**GJA12**	gap junction protein, alpha 12, 47kDa	0,0299632	0,895
207607_at	**ASCL2**	achaete-scute complex-like 2 (Drosophila)	0,0299753	0,899
232771_at	**NRK**	Nik related kinase	0,0299766	0,916

Supplementary Table 4A, Garcia-Gomez et al.

Transcription factor binding sites (TFBS) as putative promotors of gene expression									
**699 genes up-regulated in d/pMSCs cocultured with the MM.1S cell line versus non-cocultured MSCs**									
**Tool**	**Gene locus / TFBS location**	**TF family**	**TF**	**TransFac ID or Jaspar ID**	**Gene Hits**	**TFBS Hits**	**Total Genes**	**Adjusted *p*-value**	**TF found in our list**
TransFind	from -800 to +200	REL	RELA	MA0107.1	18	-	581	0.001493	RELB
oPOSSUM	from -2000 to +0	REL	RELA	MA0107.1	157	227	581	1.579e-08	RELB
oPOSSUM	from -2000 to +0	HMG	Sox9	MA0077.1	233	496	581	0.000001	-
oPOSSUM	from -2000 to +0	FORKHEAD	FOXI1	MA0042.1	197	410	581	0.000089	FOXO3 A
oPOSSUM	from -2000 to +0	ETS	ELK1	MA0028.1	319	784	581	0.000001	-
TransFind	from -800 to +200	ETS	GABPA	MA0062.1	15	-	584	0.001225	-
oPOSSUM	from -2000 to +0	PAIRED	Pax6	MA0069.1	28	30	581	0.000175	-
TransFind	from -800 to +200	PAIRED	Pax5	MA0014.1	22	-	577	0.000034	-
TransFind	from -800 to +200	ZN-FINGER	PLAG1	MA0163.1	20	-	579	0.000496	-
TransFind	from -800 to +200	ZN-FINGER	Klf4	MA0039.2	16	-	583	0.057747	Klf6, Klf7, Klf9, Klf10
oPOSSUM	from -2000 to +0	ZN-FINGER	Gfi	MA0038.1	253	652	581	0.001453	-
**1250 genes up-regulated exclusively in pMSCs co-cultured with the MM.1S cell line versus MM-MSCs non-cocultured pMSCs**									
**Tool**	**Gene locus / TFBS location**	**TF family**	**TF**	**TransFac ID or Jaspar ID**	**Gene Hits**	**TFBS Hits**	**Total Genes**	**Adjusted *p*-value**	**TF found in our list**
oPOSSUM	from -2000 to +0	ETS	GABPA	MA0062.1	29	493	1582	0.001123	GABPA
TransFind	from -800 to +200	ETS	GABPA	MA0062.1	87	-	2558	0.000001	GABPA
oPOSSUM	from -2000 to +0	ETS	ELK4	MA0076.1	85	392	1582	0.000593	ELK4
TransFind	from -800 to +200	ETS	ELK4	MA0076.1	89	-	2556	0.000001	ELK4
oPOSSUM	from -2000 to +0	ZN-FINGER	MIZF	MA0131.1	67	137	1582	0.04443	-
TransFind	from -800 to +200	ZN-FINGER	MIZF	MA0131.1	65	-	2580	0.000001	-
TransFind	from -800 to +200	NFYCCAAT-binding	NFYA	MA0060.1	63	-	2582	0.000001	NFYA

Supplementary Table 4B, Garcia-Gomez et al.

TF with the highest number of TFBS and gene hits		Target genes in Lists I & II
List I	Elk1	ADAM12, ADAMTS6, ANGPTL4, APAF1, CCL20, CXCR4, DR6, EREG, ETS1, EXT1, FGF2, FN1, GDF5, HAS2, HBEGF, HGF, IL16, KLF10, LIF, MMP9, NRP1, NRG1, PECAM1, SEMA4C, TNC, VEGF, WNT5A
	Gfi1	ADAMTS6, AQP9, BSF3, CXCR4, ETS1, FGF2, FN1, GDF5, HBEGF, IL16, IL6, ITGA2, KLF7, KLF9, KLF10, MMP13, MMP19, NRG1, NRP1, SEMA4C, SPP1, TLR8, TNC, VCAM1, VEGF, WNT5A, WNT5B
List II	GABPA	CCR3, FGF14, HES1, IRE1, KLF12, NDP, NRG3, TCF7, WNT16
	Elk4	CCR3, HES1, IRE1, NFATC2, NRG3, TCF7
